# Supplementary figures and images for: Analysis of microRNA expression profiles in exosomes derived from acute myeloid leukemia by p62 knockdown and effect on angiogenesis (part 1 of 2)
Source: PeerJ. 2022 Jul 22;10:e13498. doi: 10.7717/peerj.13498 (PMC9310811; doi:10.7717/peerj.13498)

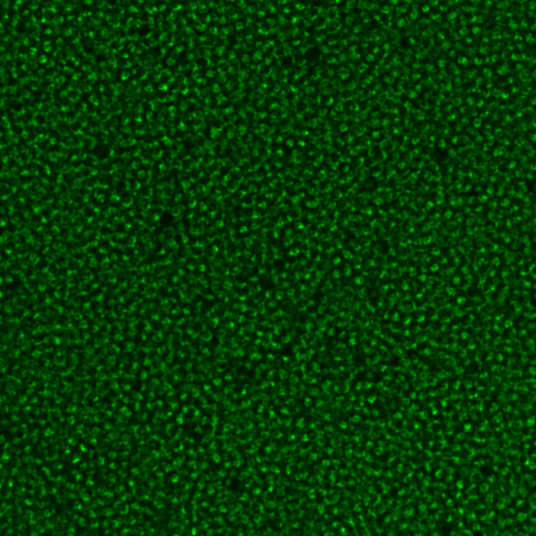

Supplement: Supplemental Information 1 [file peerj-10-13498-s001.zip › 20180925 P62-transfection/con-1-200.tif]

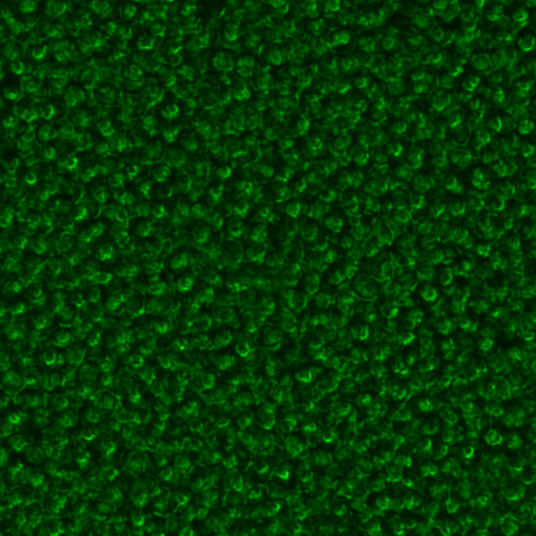

Supplement: Supplemental Information 1 [file peerj-10-13498-s001.zip › 20180925 P62-transfection/con-1-400.tif]

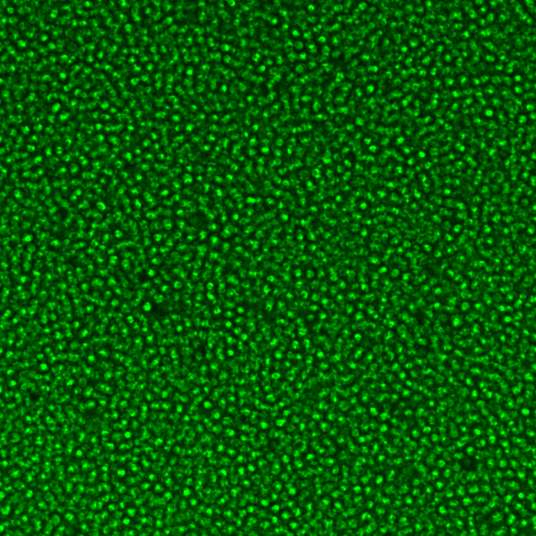

Supplement: Supplemental Information 1 [file peerj-10-13498-s001.zip › 20180925 P62-transfection/con-2-200.tif]

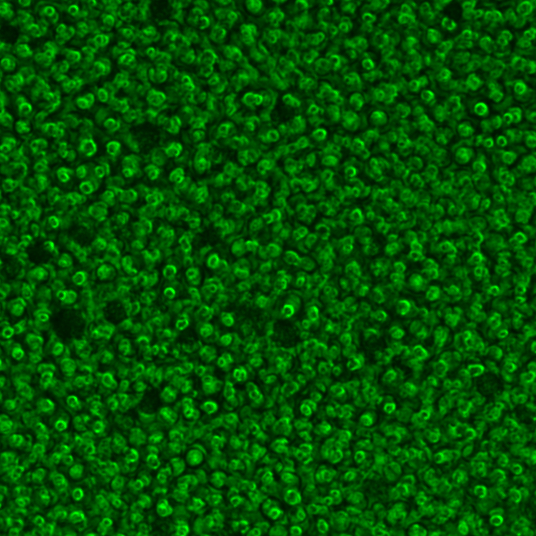

Supplement: Supplemental Information 1 [file peerj-10-13498-s001.zip › 20180925 P62-transfection/con-2-400.tif]

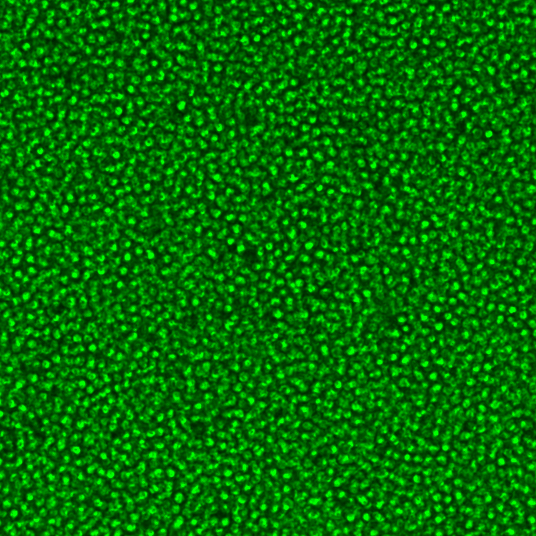

Supplement: Supplemental Information 1 [file peerj-10-13498-s001.zip › 20180925 P62-transfection/con-3-200.tif]

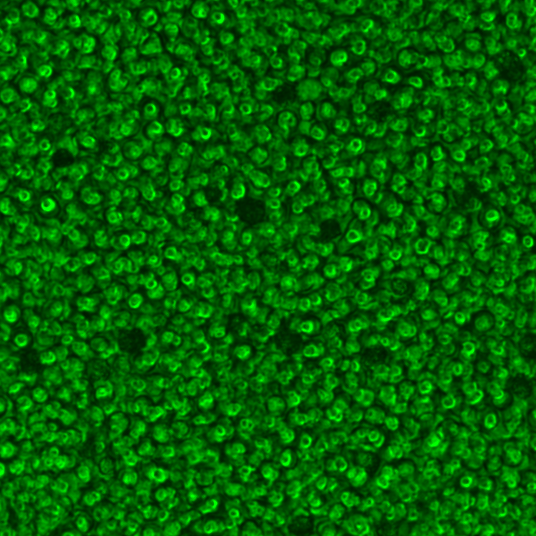

Supplement: Supplemental Information 1 [file peerj-10-13498-s001.zip › 20180925 P62-transfection/con-3-400.tif]

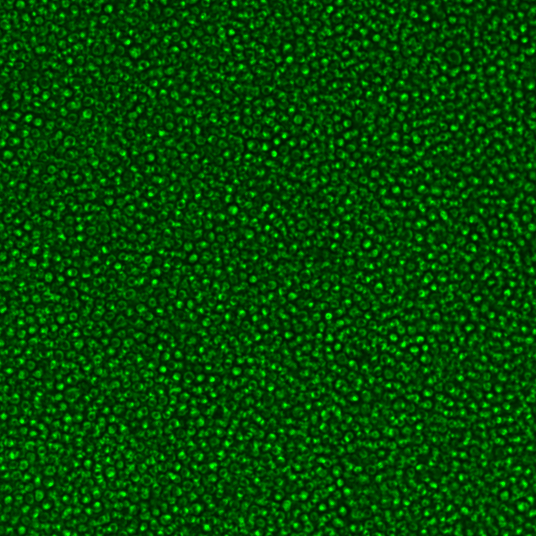

Supplement: Supplemental Information 1 [file peerj-10-13498-s001.zip › 20180925 P62-transfection/p62-1-200.tif]

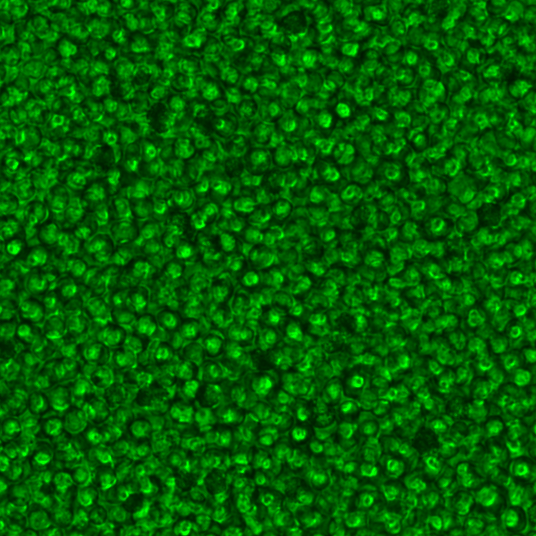

Supplement: Supplemental Information 1 [file peerj-10-13498-s001.zip › 20180925 P62-transfection/p62-1-400.tif]

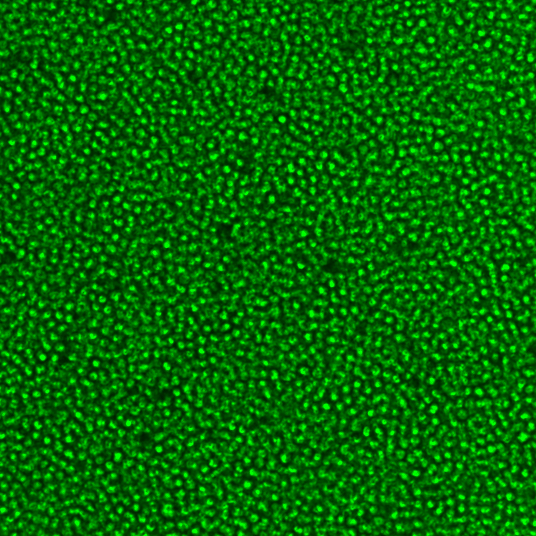

Supplement: Supplemental Information 1 [file peerj-10-13498-s001.zip › 20180925 P62-transfection/p62-2-200.tif]

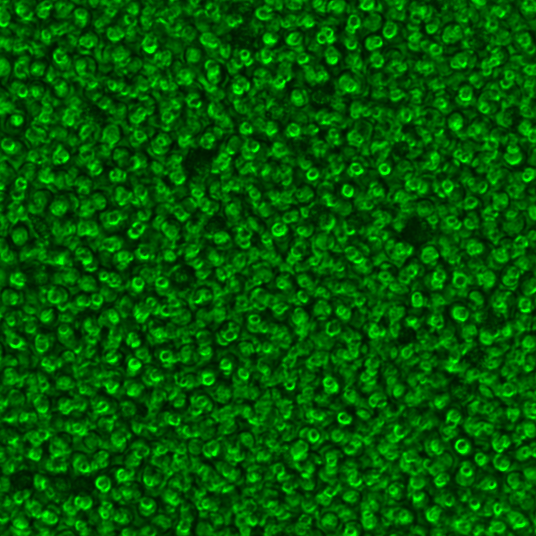

Supplement: Supplemental Information 1 [file peerj-10-13498-s001.zip › 20180925 P62-transfection/p62-2-400.tif]

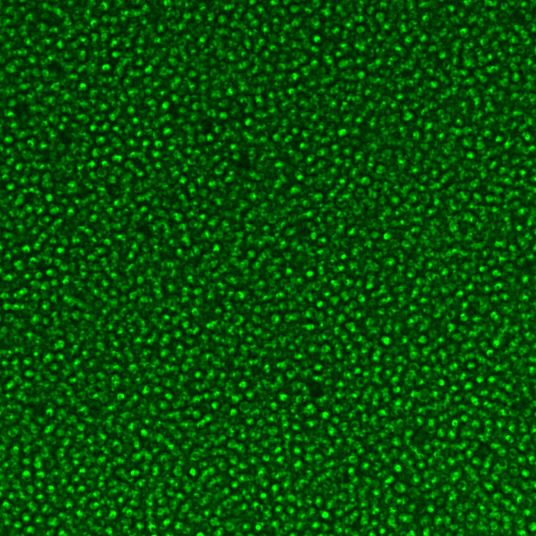

Supplement: Supplemental Information 1 [file peerj-10-13498-s001.zip › 20180925 P62-transfection/p62-3-200.tif]

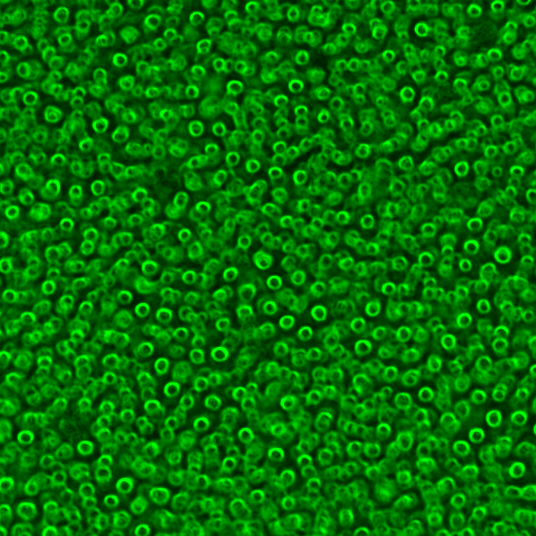

Supplement: Supplemental Information 1 [file peerj-10-13498-s001.zip › 20180925 P62-transfection/p62-3-400.tif]

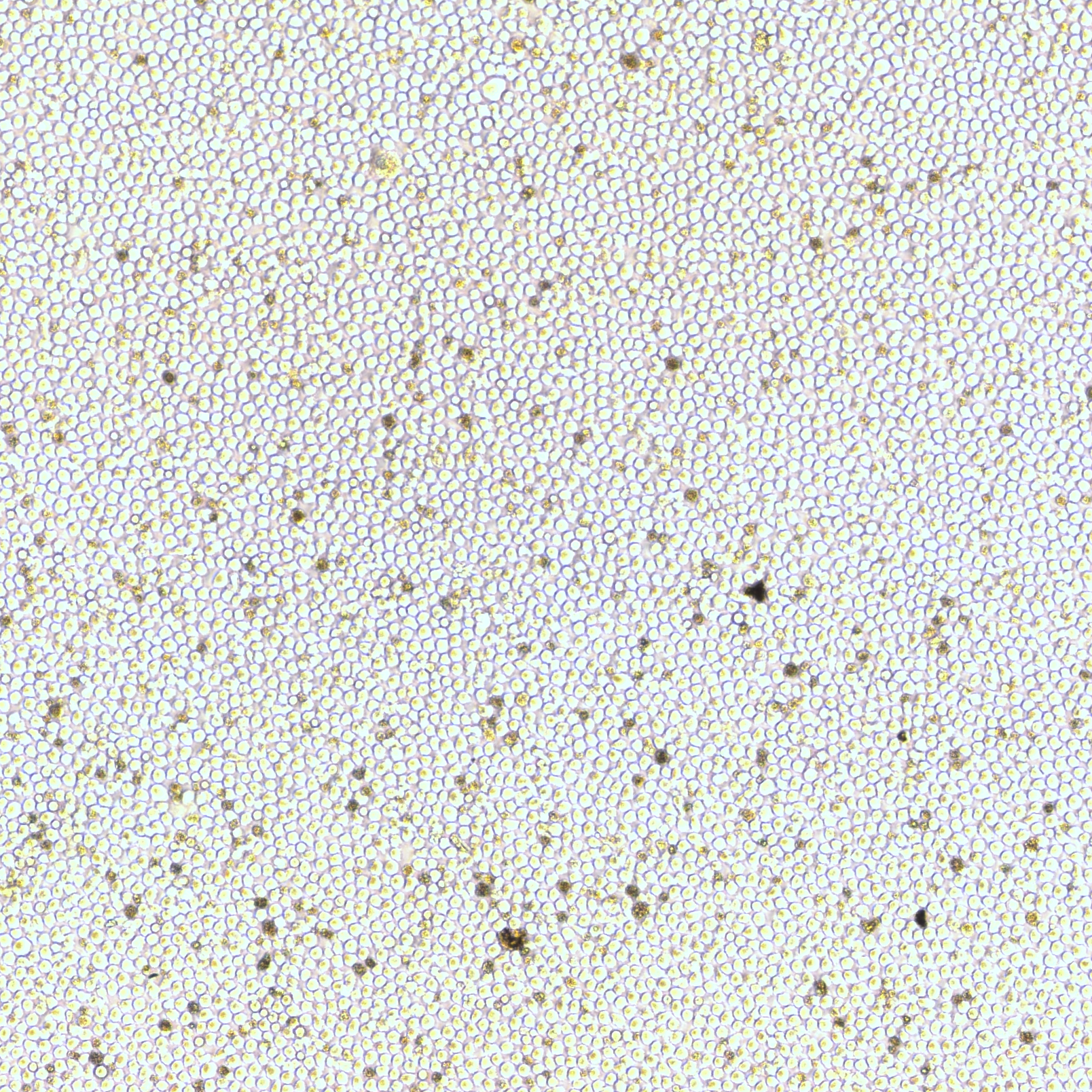

Supplement: Supplemental Information 2 [file peerj-10-13498-s002.zip › lentiviral transfection 1/con-3.tif]

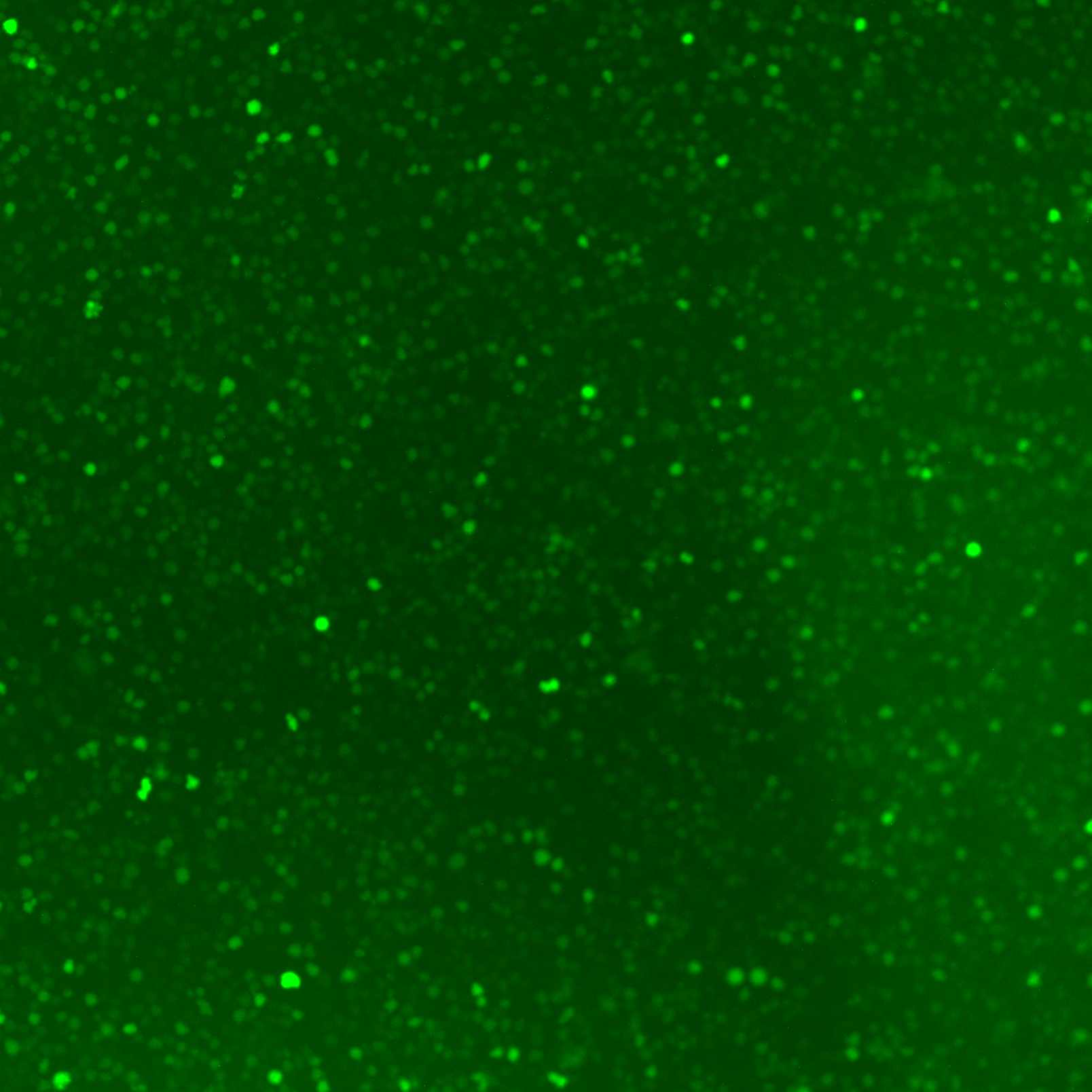

Supplement: Supplemental Information 2 [file peerj-10-13498-s002.zip › lentiviral transfection 1/con01.tif]

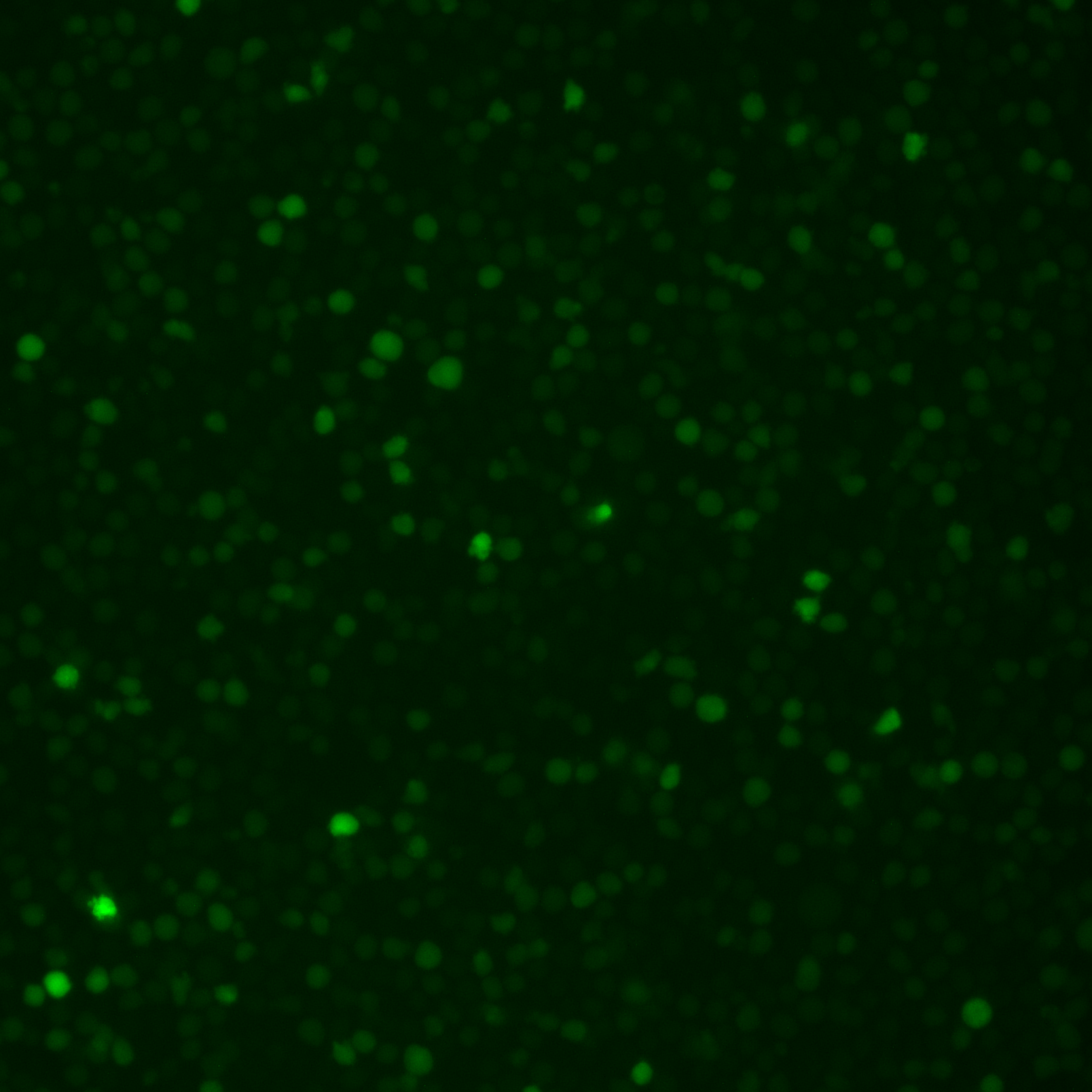

Supplement: Supplemental Information 2 [file peerj-10-13498-s002.zip › lentiviral transfection 1/con02.tif]

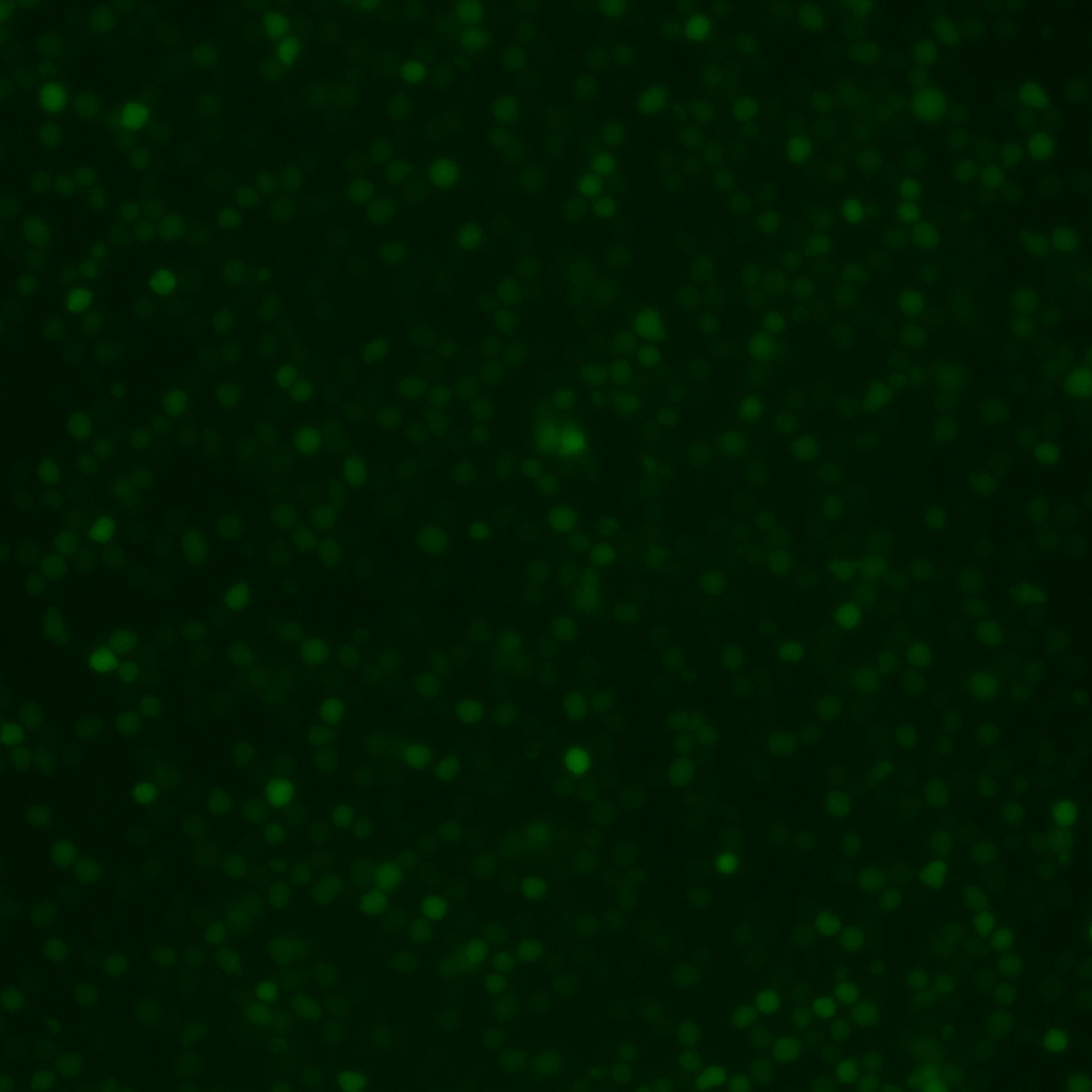

Supplement: Supplemental Information 2 [file peerj-10-13498-s002.zip › lentiviral transfection 1/P62-04-400.tif]

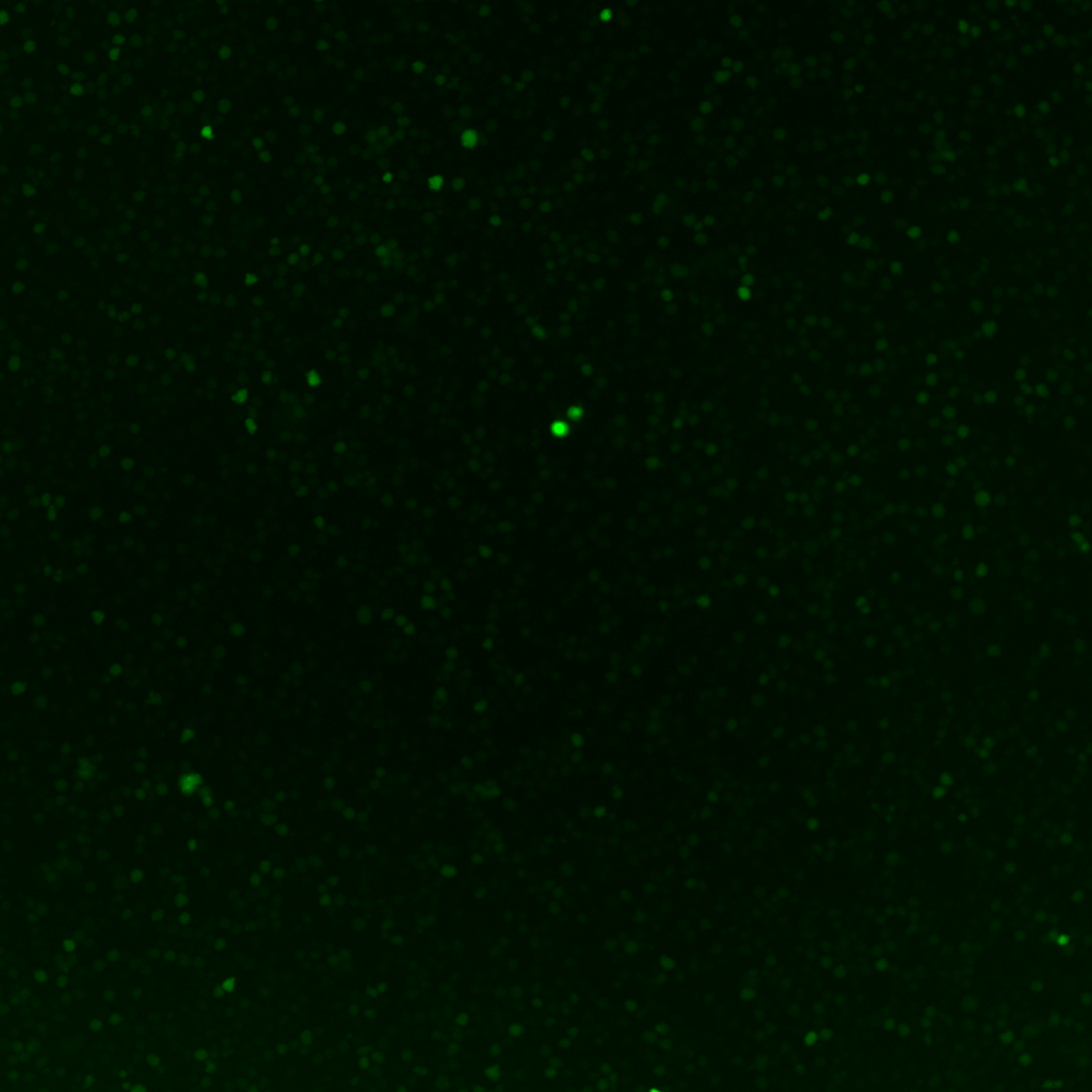

Supplement: Supplemental Information 2 [file peerj-10-13498-s002.zip › lentiviral transfection 1/P62-1.tif]

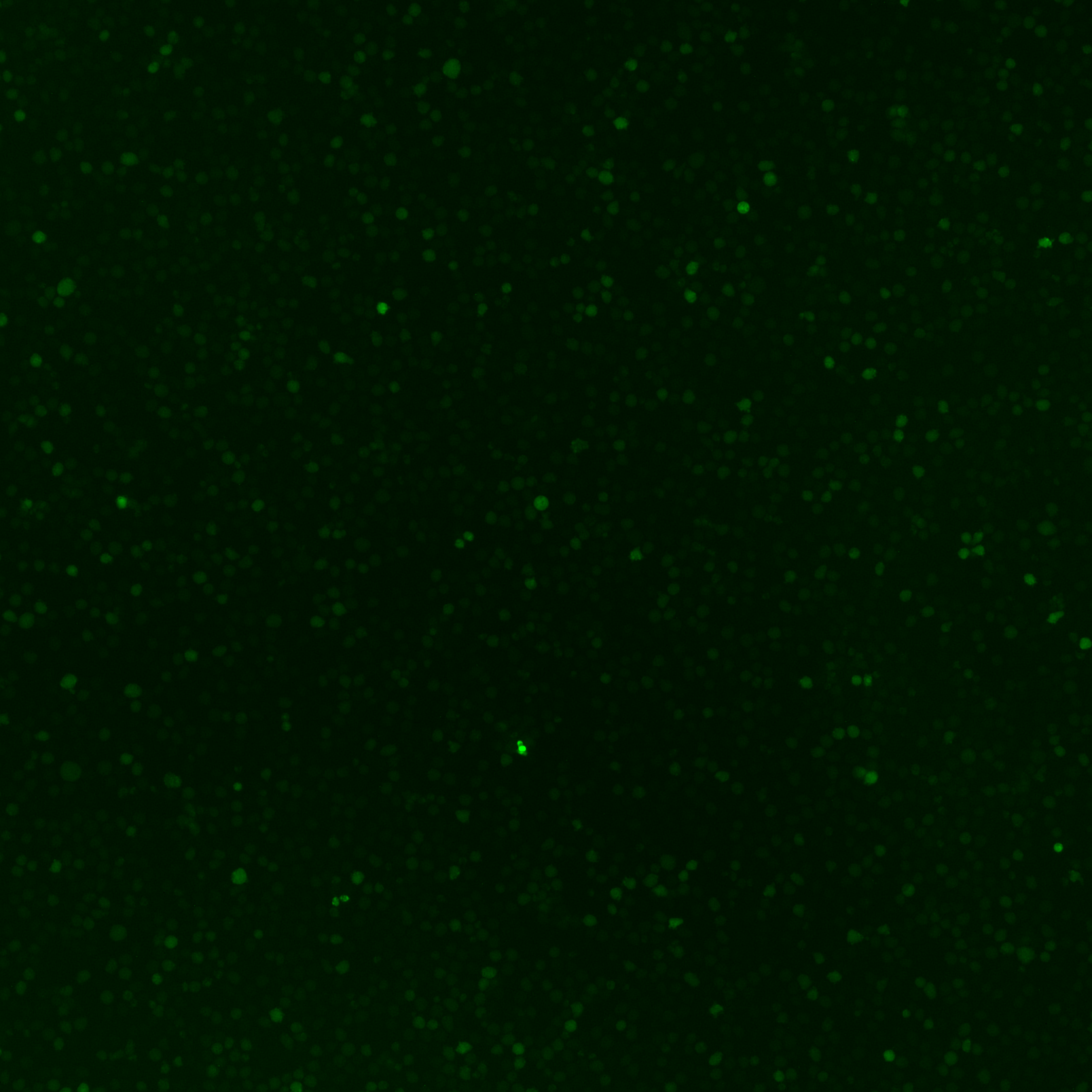

Supplement: Supplemental Information 2 [file peerj-10-13498-s002.zip › lentiviral transfection 1/p62-2.tif]

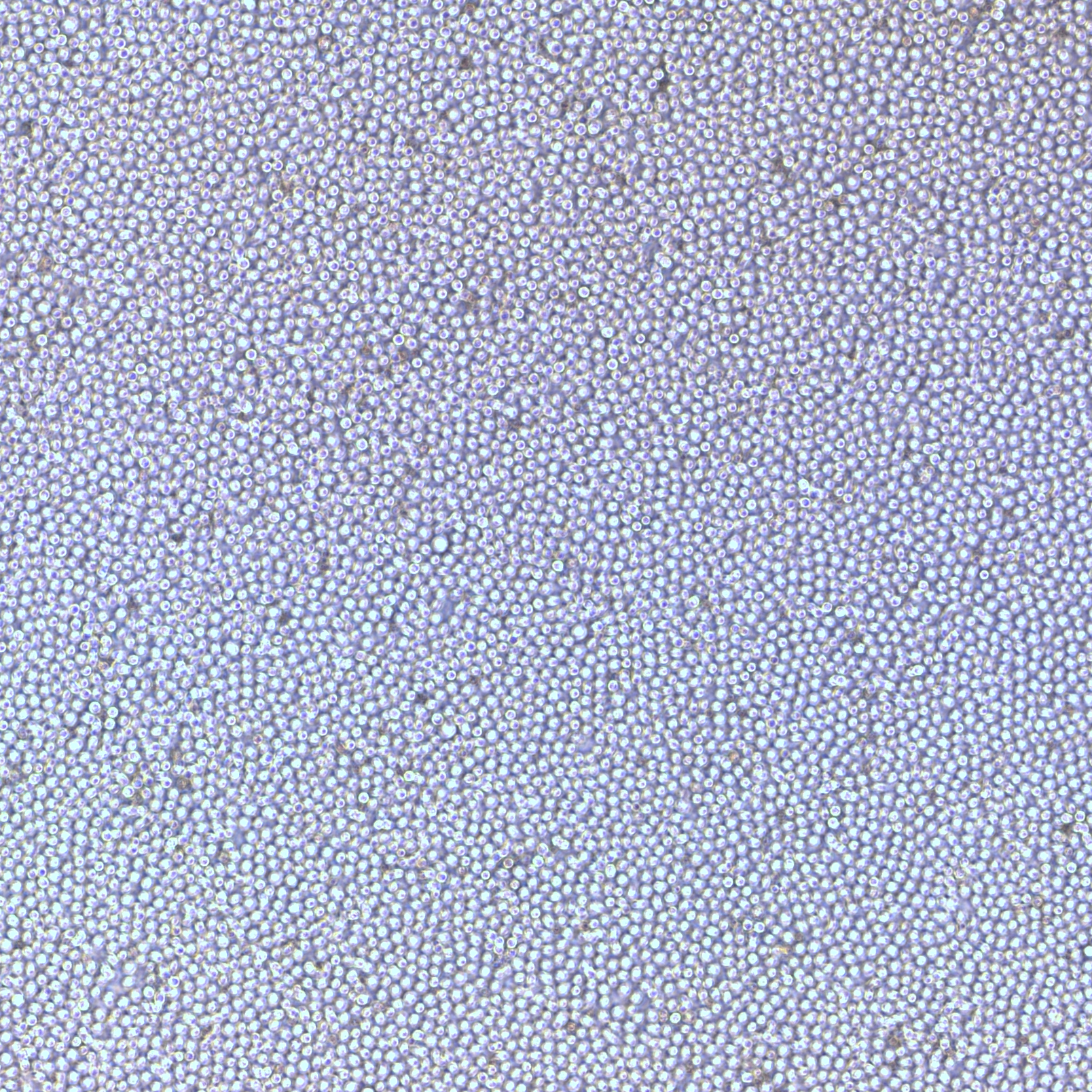

Supplement: Supplemental Information 2 [file peerj-10-13498-s002.zip › lentiviral transfection 1/p62-3.tif]

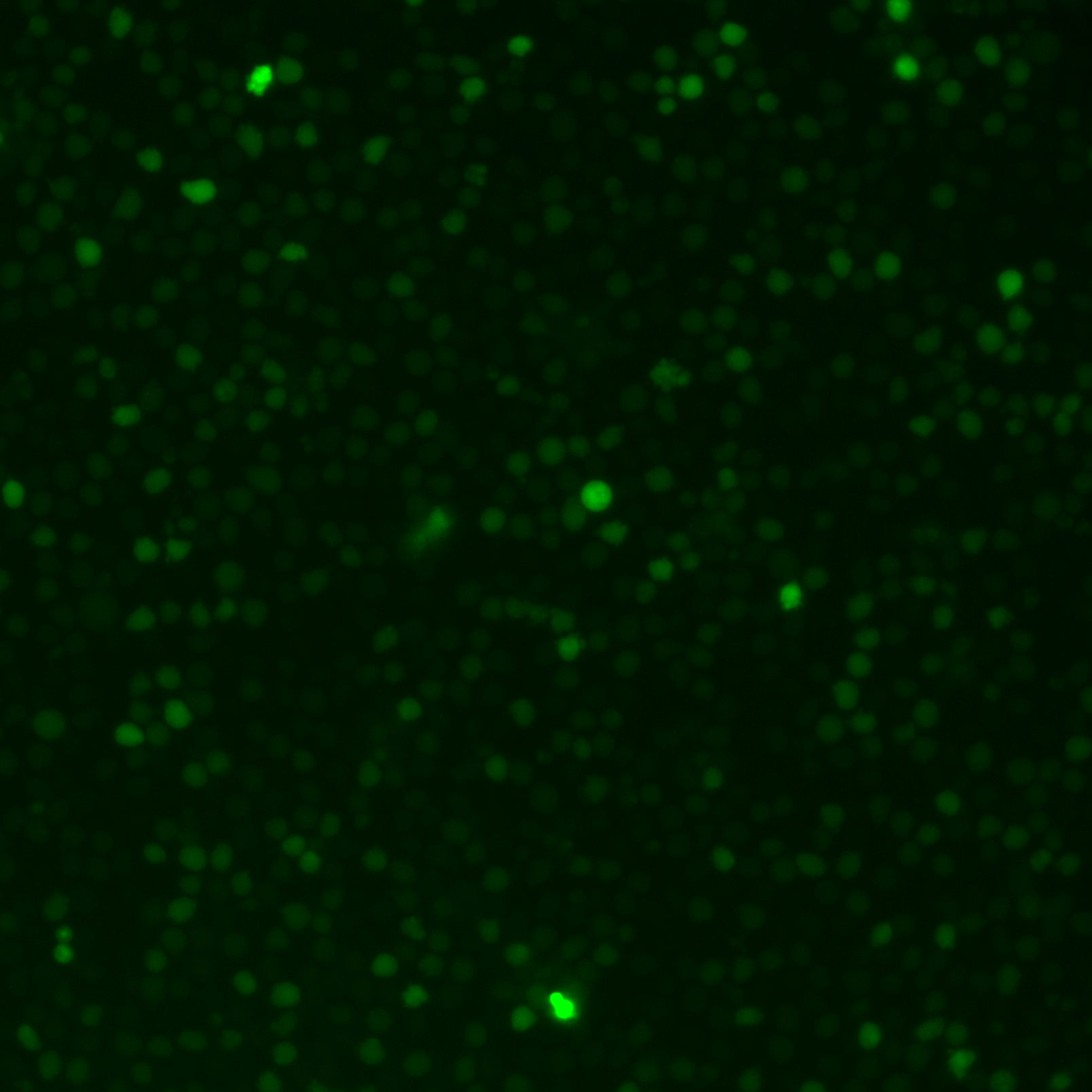

Supplement: Supplemental Information 2 [file peerj-10-13498-s002.zip › lentiviral transfection 1/p62-400.tif]

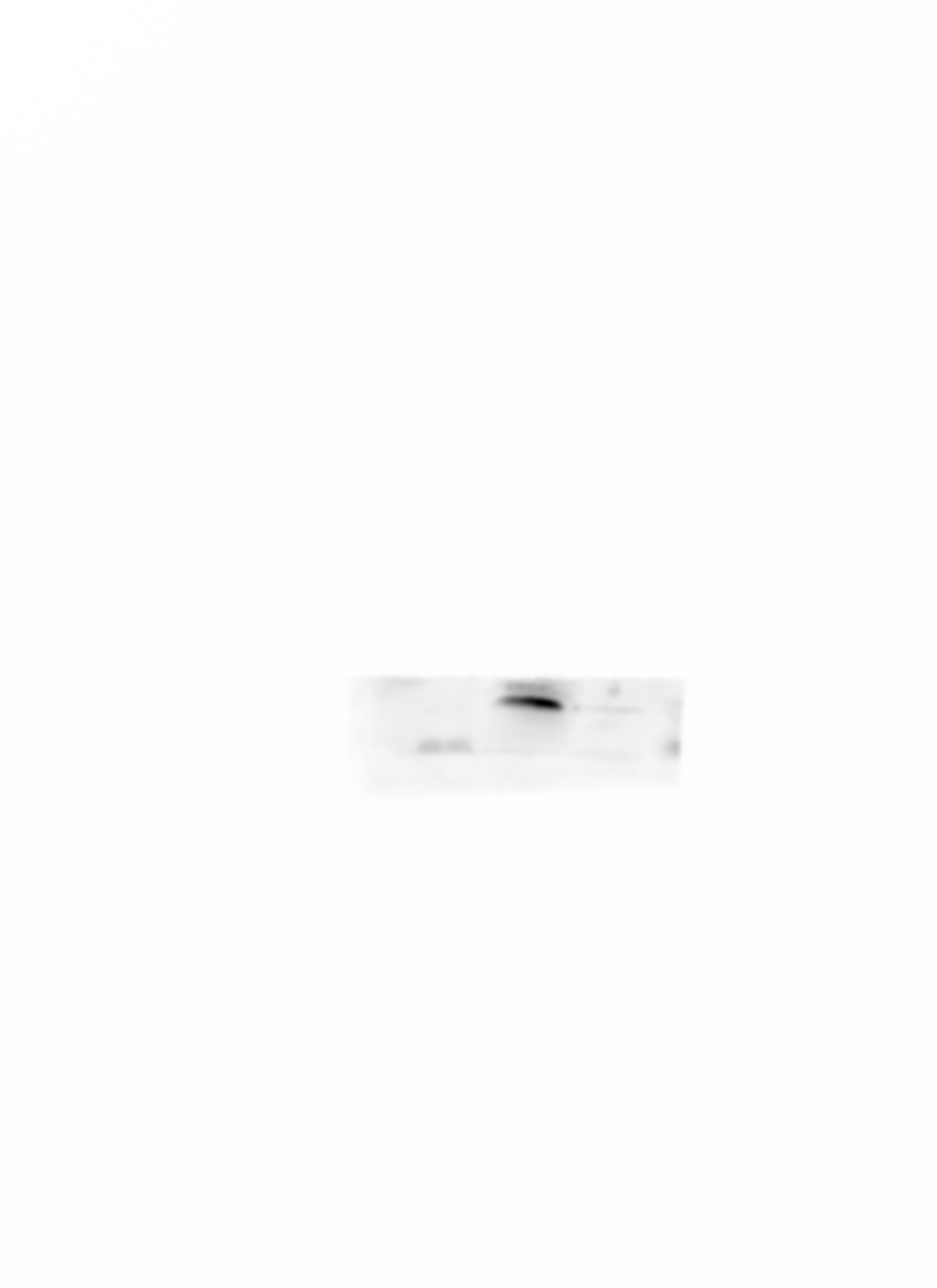

Supplement: Supplemental Information 3 [file peerj-10-13498-s003.zip › 2. WB/0411 P62 2019.04.11_11.15.25_Ch.tif]

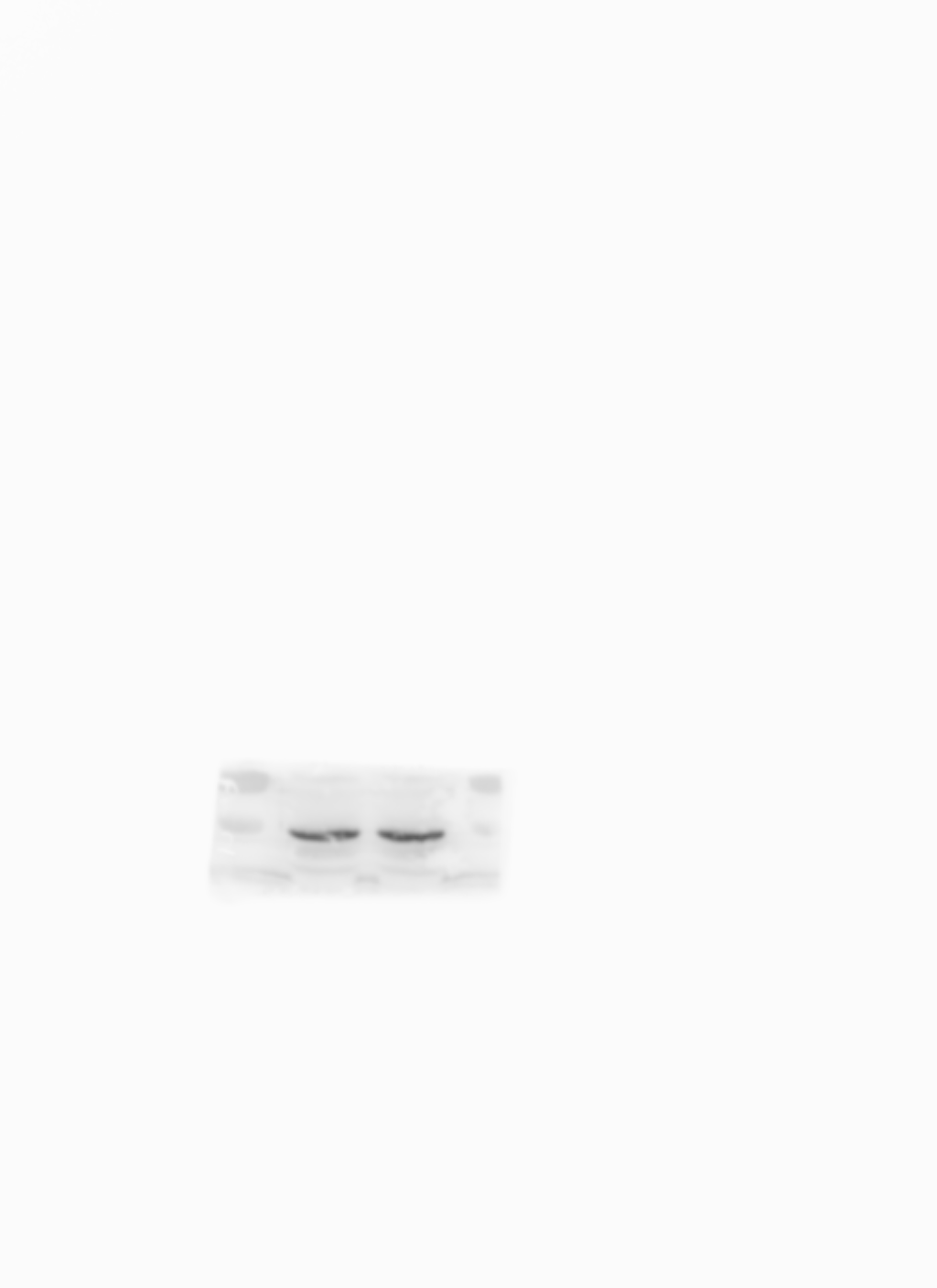

Supplement: Supplemental Information 3 [file peerj-10-13498-s003.zip › 2. WB/0411 P62' 2019.04.11_11.12.27_Ch.tif]

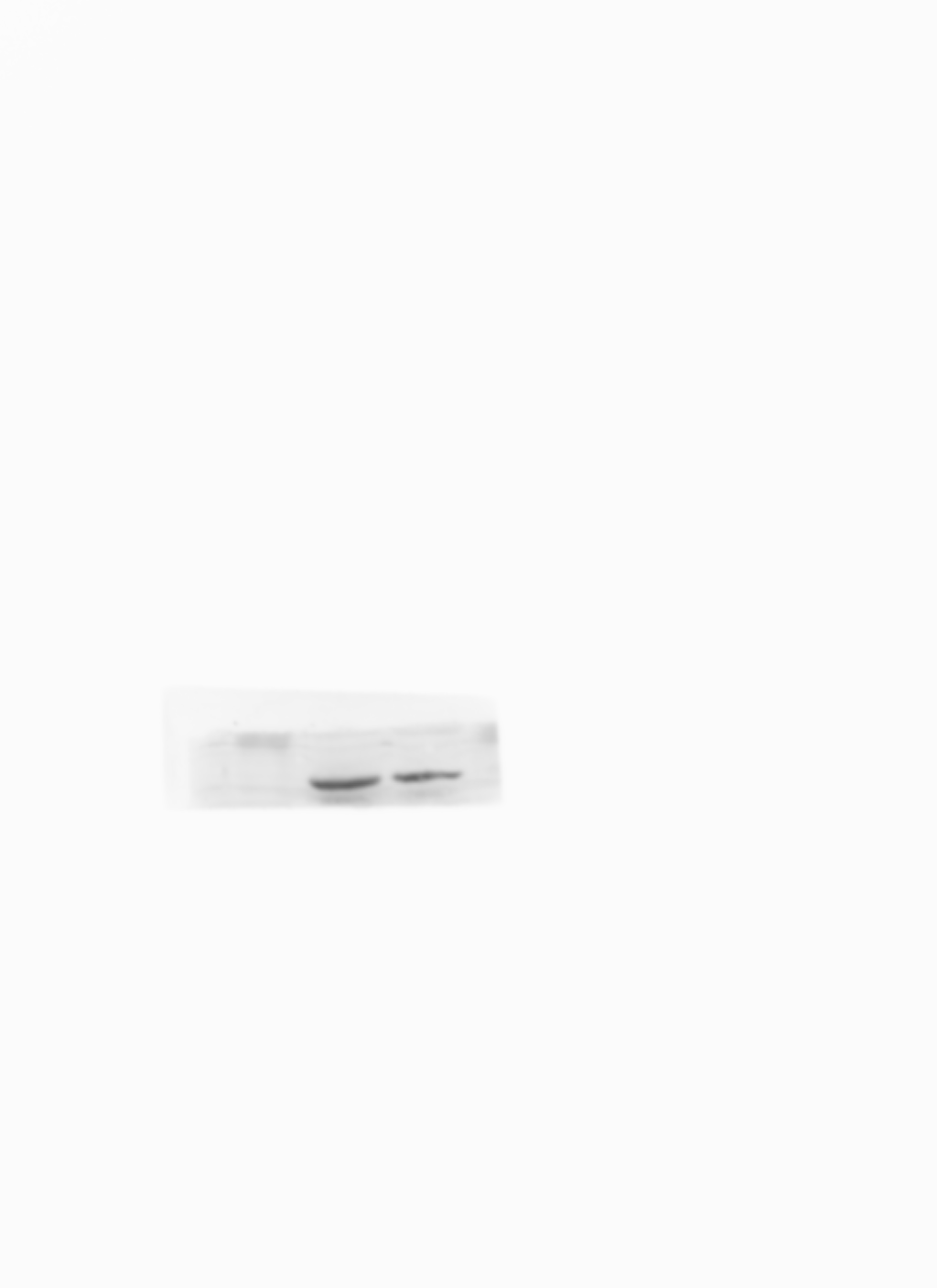

Supplement: Supplemental Information 3 [file peerj-10-13498-s003.zip › 2. WB/0411 P62-1 2019.04.11_11.18.17_Ch.tif]

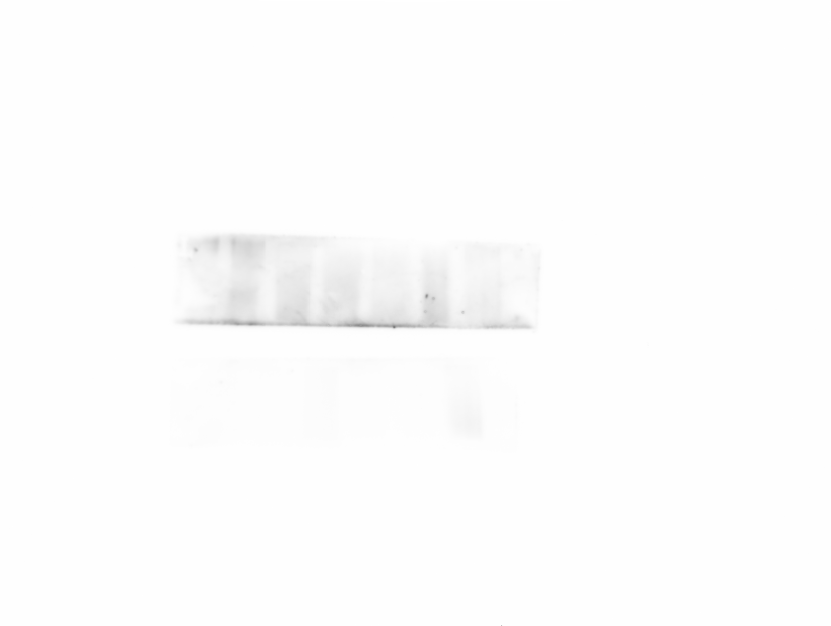

Supplement: Supplemental Information 4 [file peerj-10-13498-s004.zip › 2.Exosome/181115wb/2018-1115-144436.tif]

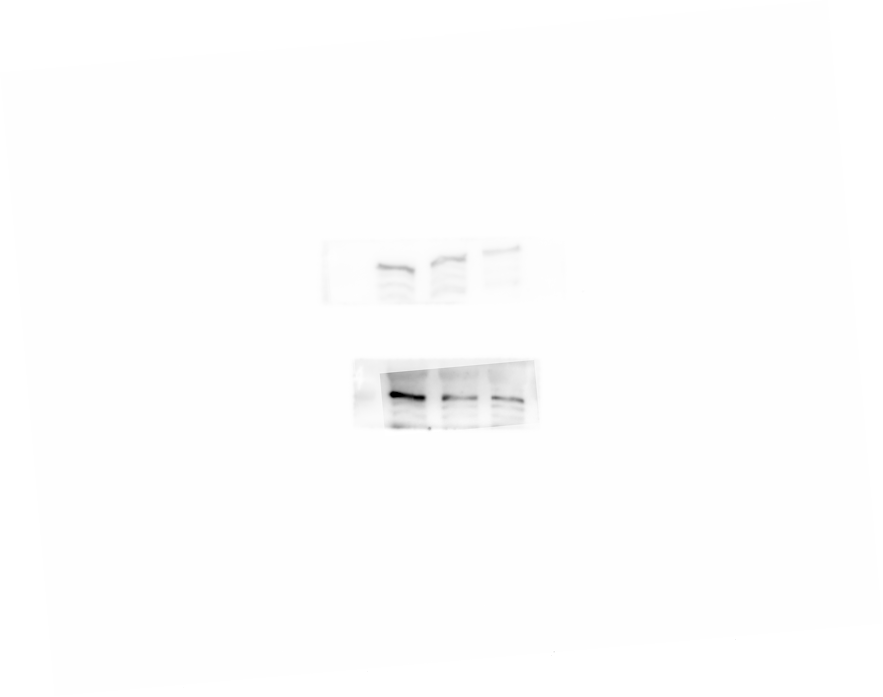

Supplement: Supplemental Information 4 [file peerj-10-13498-s004.zip › 2.Exosome/181115wb/CA.tif]

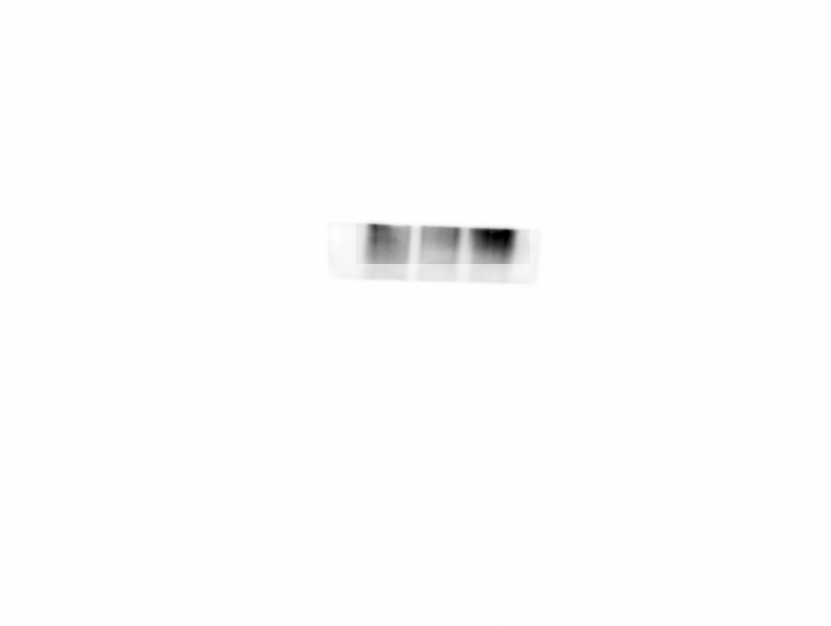

Supplement: Supplemental Information 4 [file peerj-10-13498-s004.zip › 2.Exosome/181115wb/CD63-2.tif]

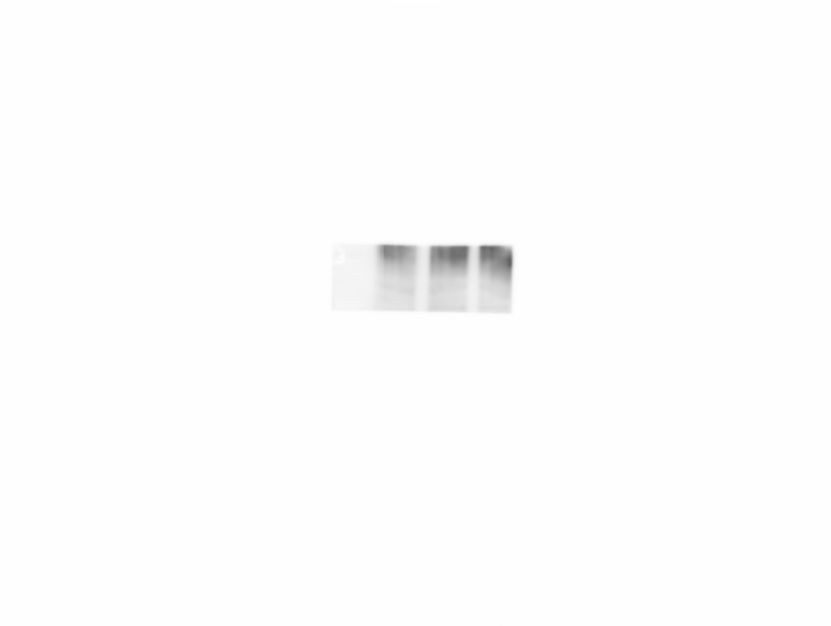

Supplement: Supplemental Information 4 [file peerj-10-13498-s004.zip › 2.Exosome/181115wb/CD63.tif]

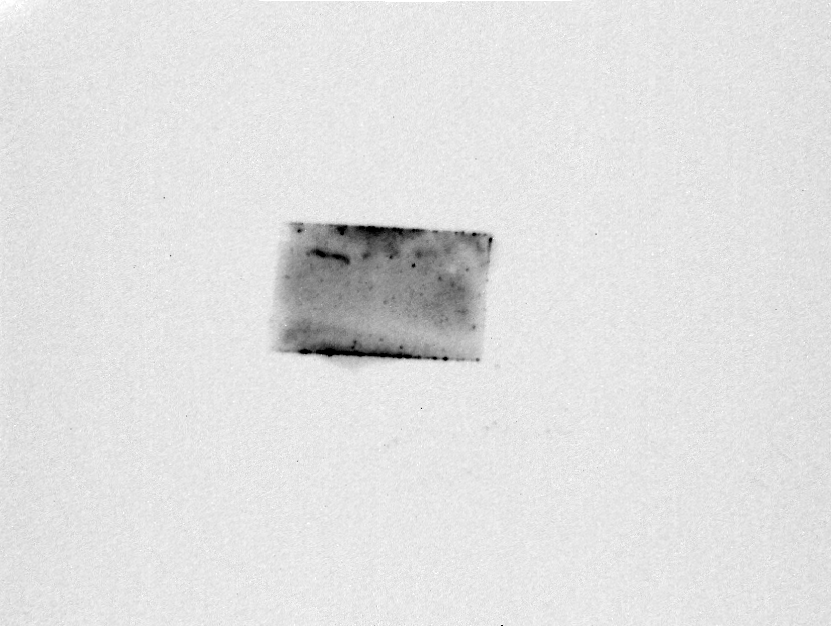

Supplement: Supplemental Information 4 [file peerj-10-13498-s004.zip › 2.Exosome/181115wb/CD9-2.tif]

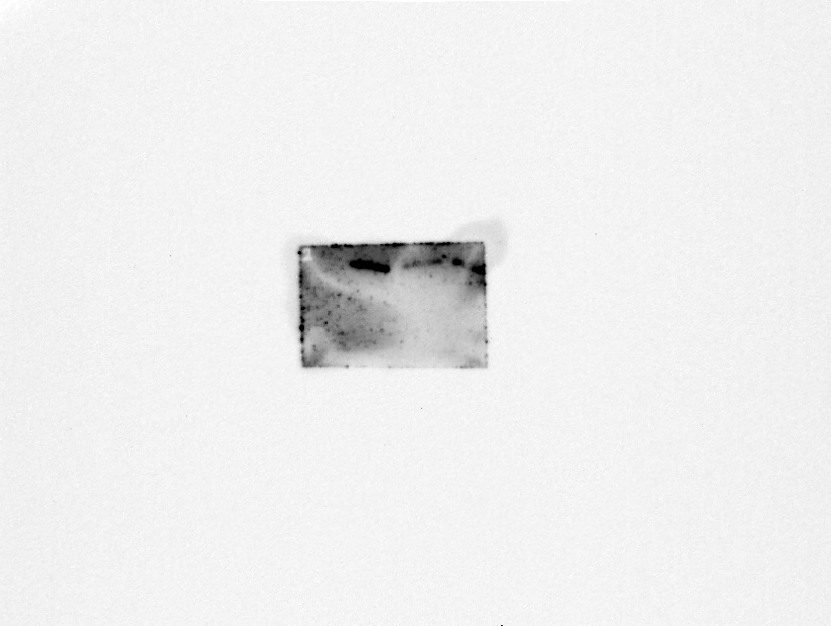

Supplement: Supplemental Information 4 [file peerj-10-13498-s004.zip › 2.Exosome/181115wb/CD9.tif]

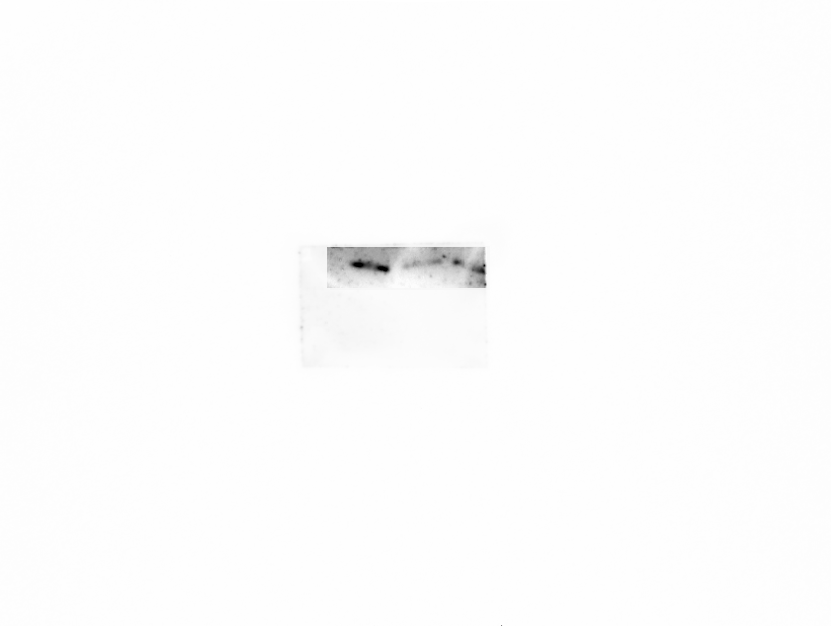

Supplement: Supplemental Information 4 [file peerj-10-13498-s004.zip › 2.Exosome/181115wb/CD9ps.tif]

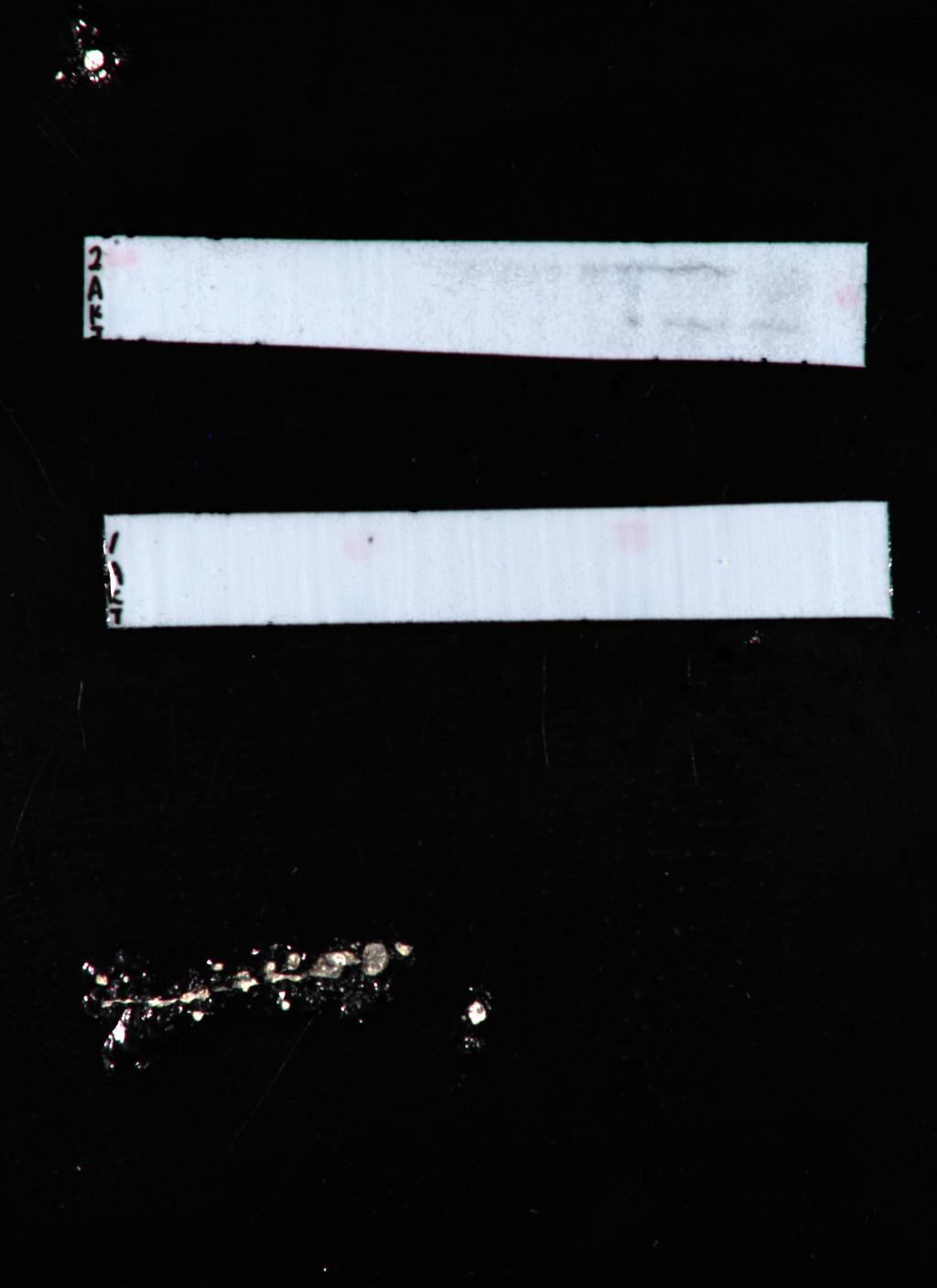

Supplement: Supplemental Information 4 [file peerj-10-13498-s004.zip › 2.Exosome/181202wb/12.2akt 2018.12.02_19.02.32_Ch/12.2akt 2018.12.02_19.02.32_Ch+Marker.jpg]

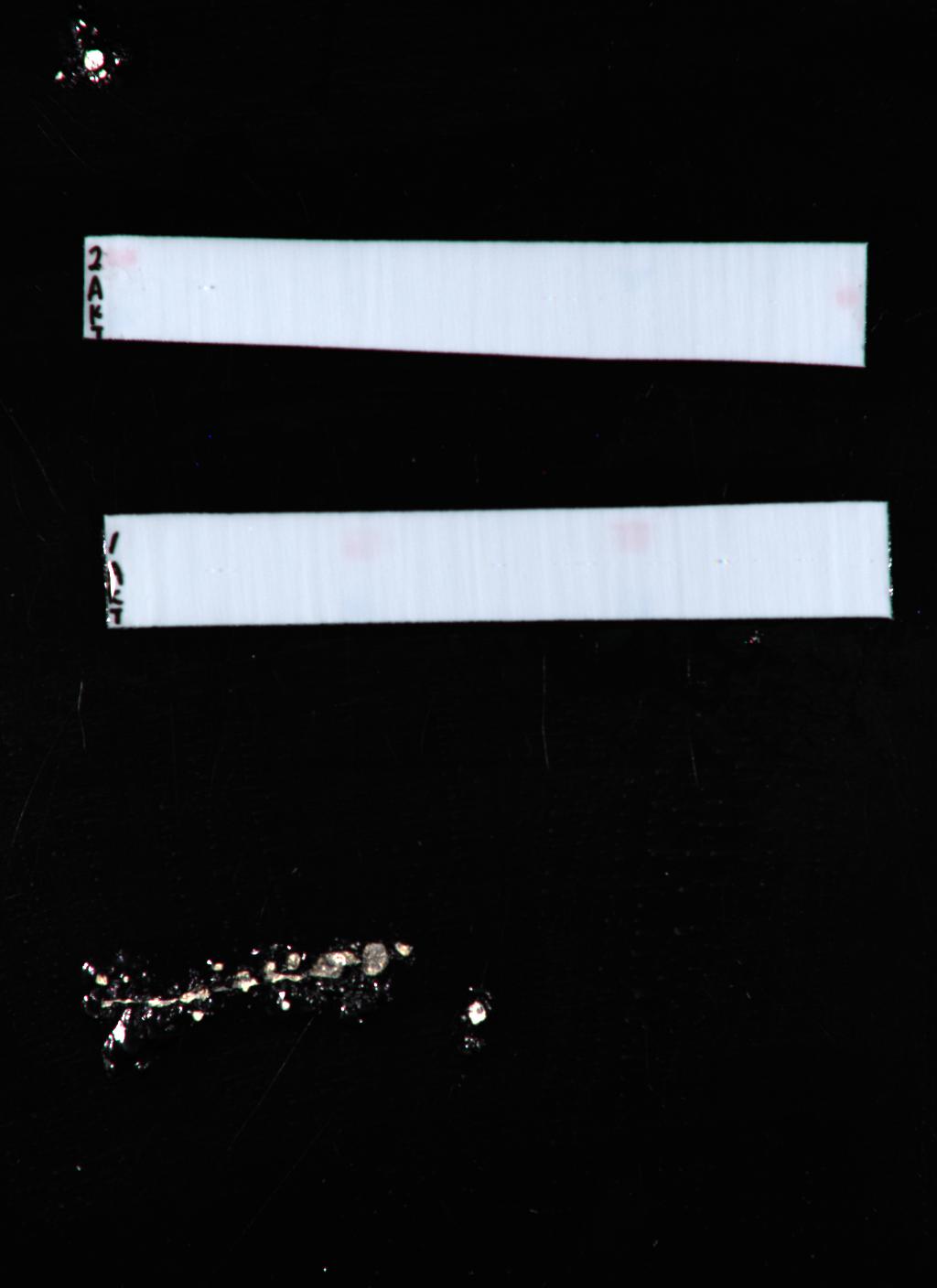

Supplement: Supplemental Information 4 [file peerj-10-13498-s004.zip › 2.Exosome/181202wb/12.2akt 2018.12.02_19.02.32_Ch/12.2akt 2018.12.02_19.02.32_Ch-Marker.jpg]

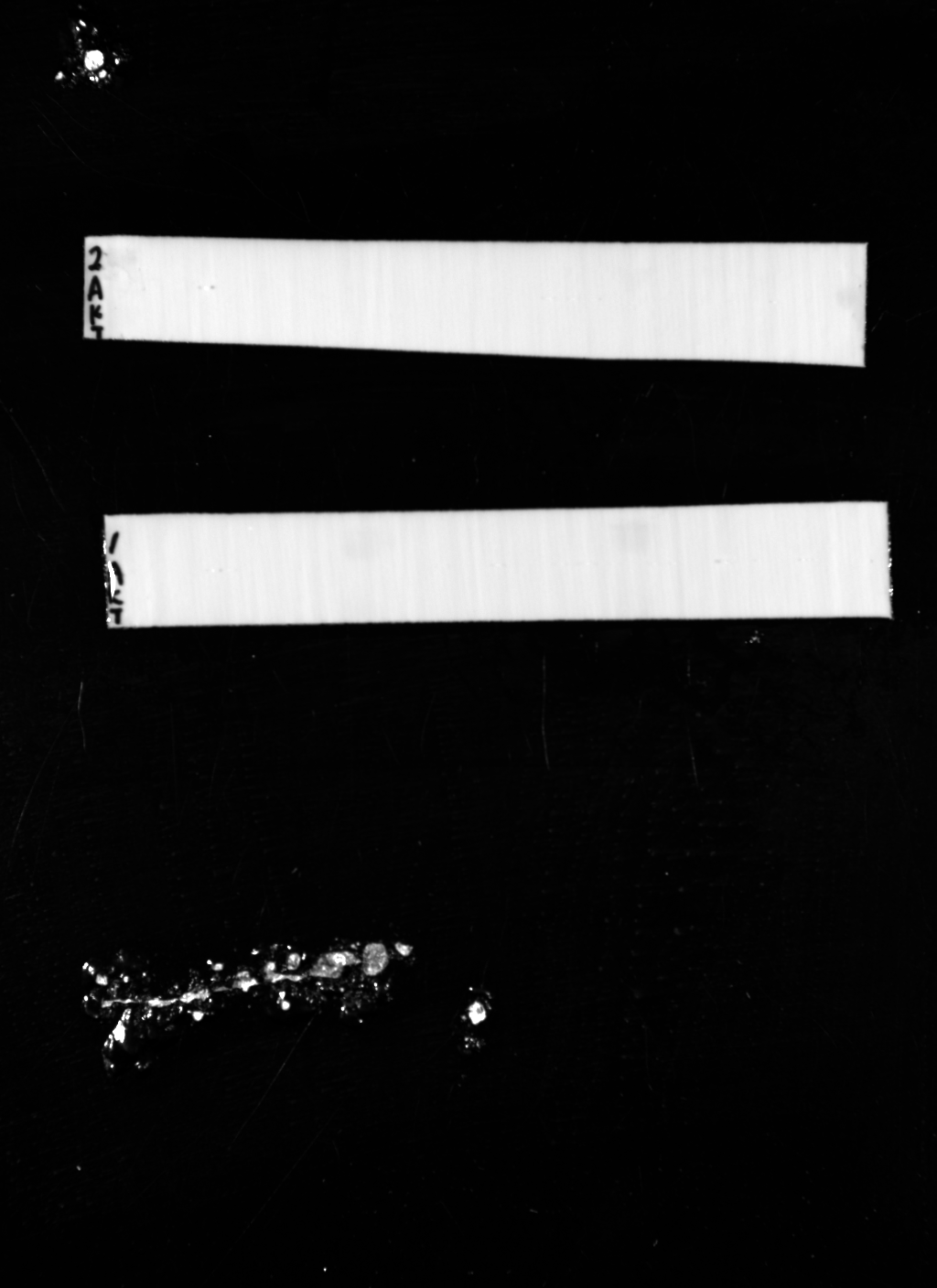

Supplement: Supplemental Information 4 [file peerj-10-13498-s004.zip › 2.Exosome/181202wb/12.2akt 2018.12.02_19.02.32_Ch/12.2akt 2018.12.02_19.02.32_Ch-Marker.tif]

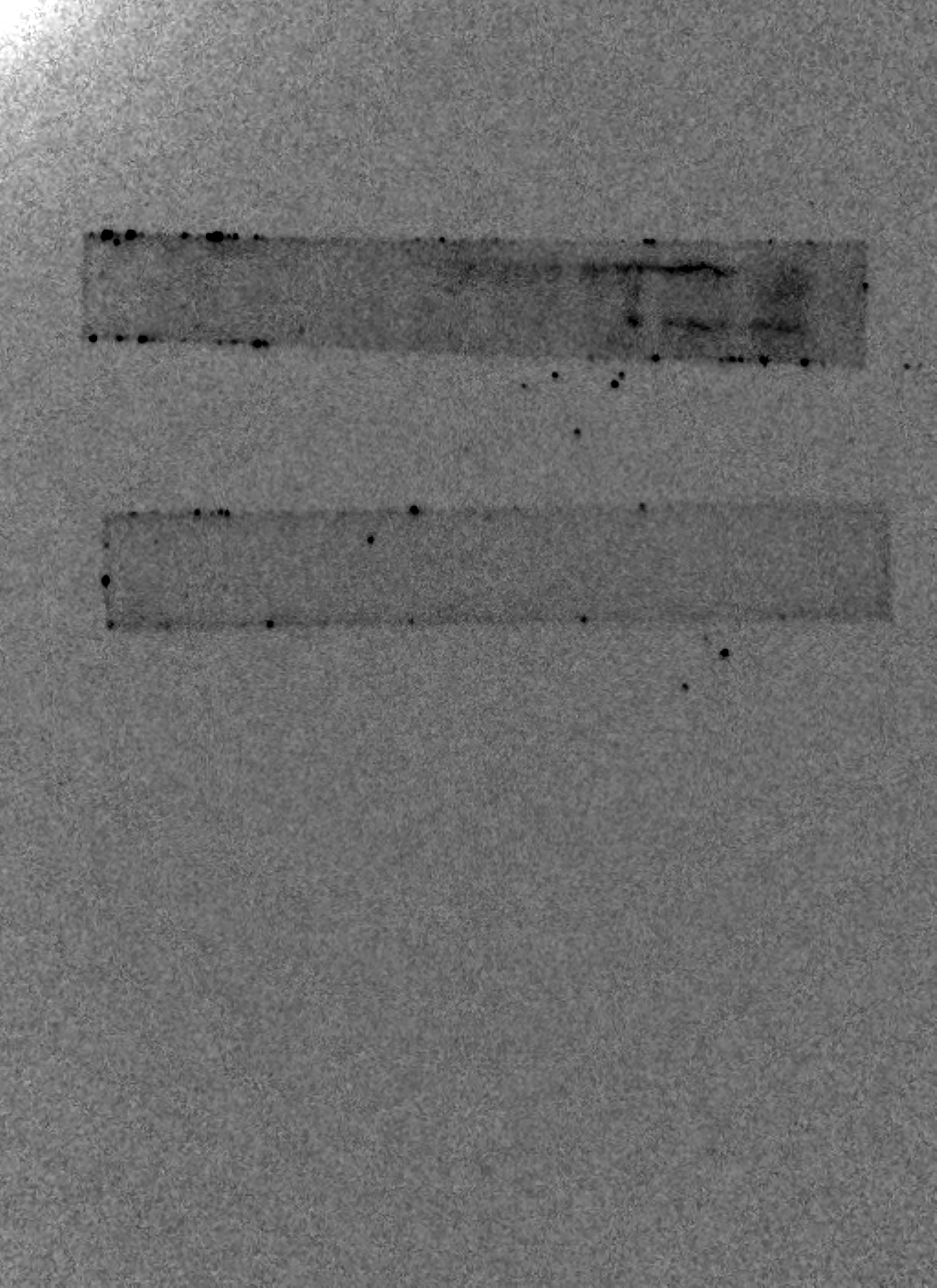

Supplement: Supplemental Information 4 [file peerj-10-13498-s004.zip › 2.Exosome/181202wb/12.2akt 2018.12.02_19.02.32_Ch/12.2akt 2018.12.02_19.02.32_Ch.tif]

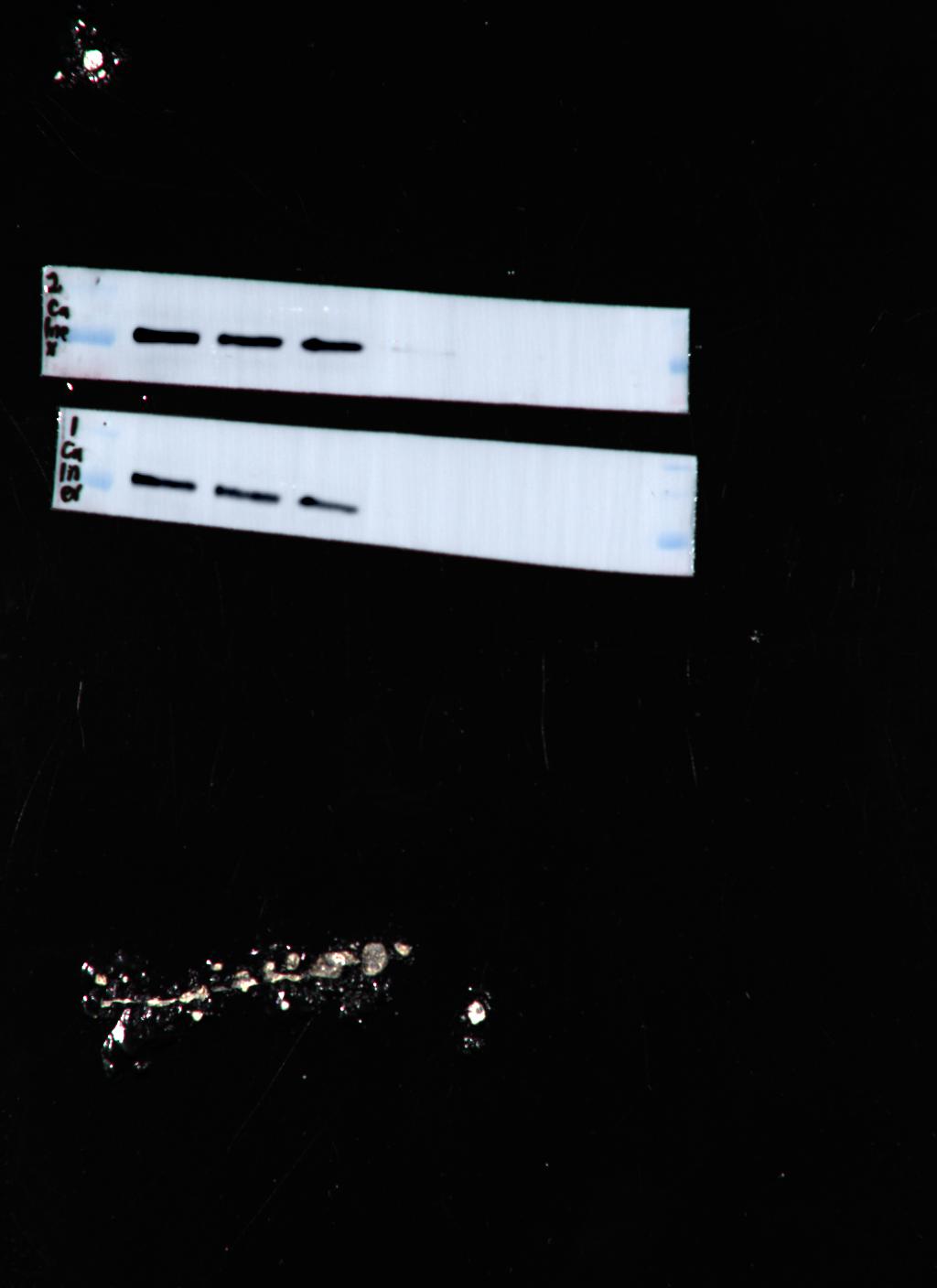

Supplement: Supplemental Information 4 [file peerj-10-13498-s004.zip › 2.Exosome/181202wb/12.2calnex 2018.12.02_18.40.20_Ch/12.2calnex 2018.12.02_18.40.20_Ch+Marker.jpg]

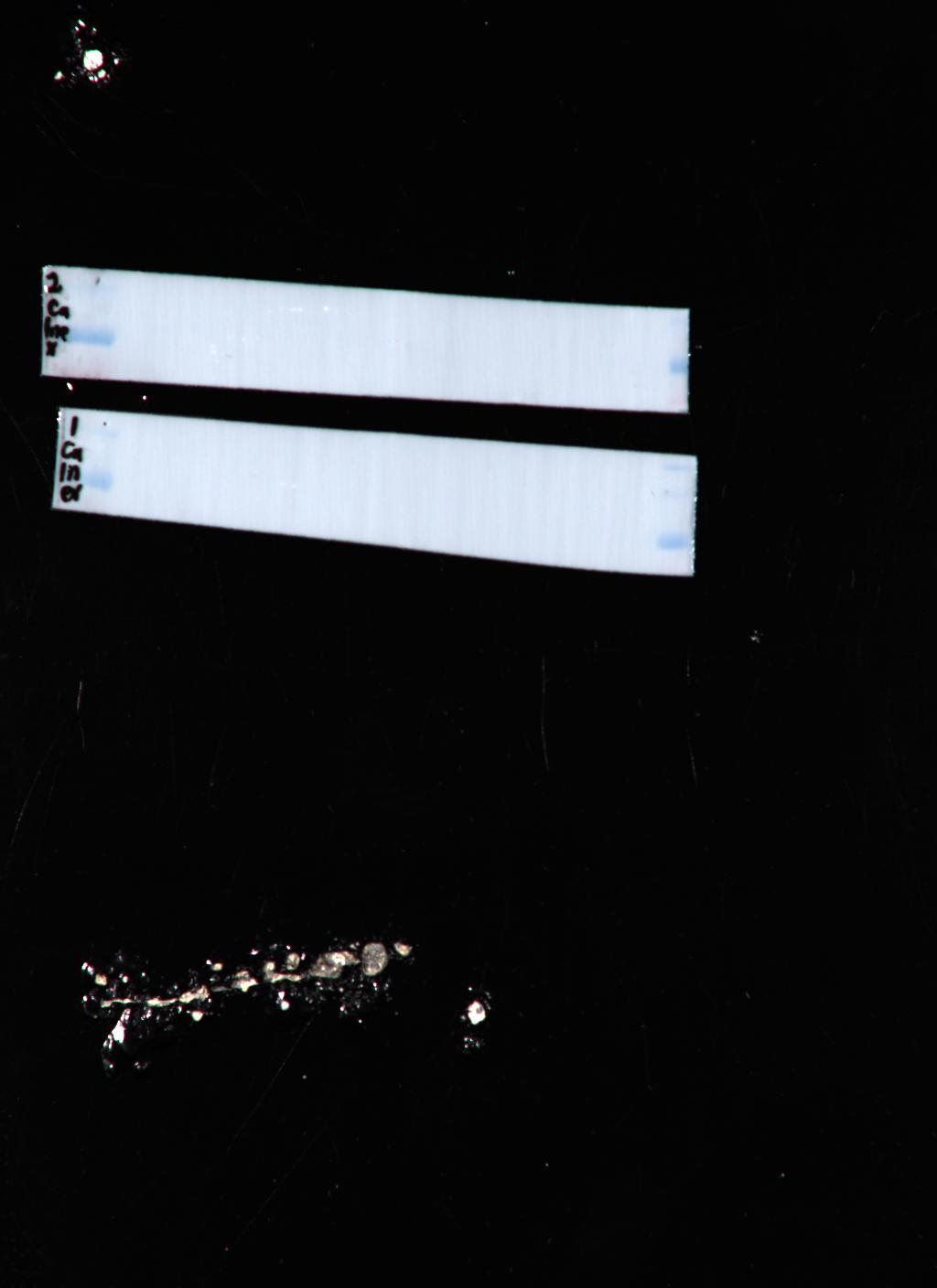

Supplement: Supplemental Information 4 [file peerj-10-13498-s004.zip › 2.Exosome/181202wb/12.2calnex 2018.12.02_18.40.20_Ch/12.2calnex 2018.12.02_18.40.20_Ch-Marker.jpg]

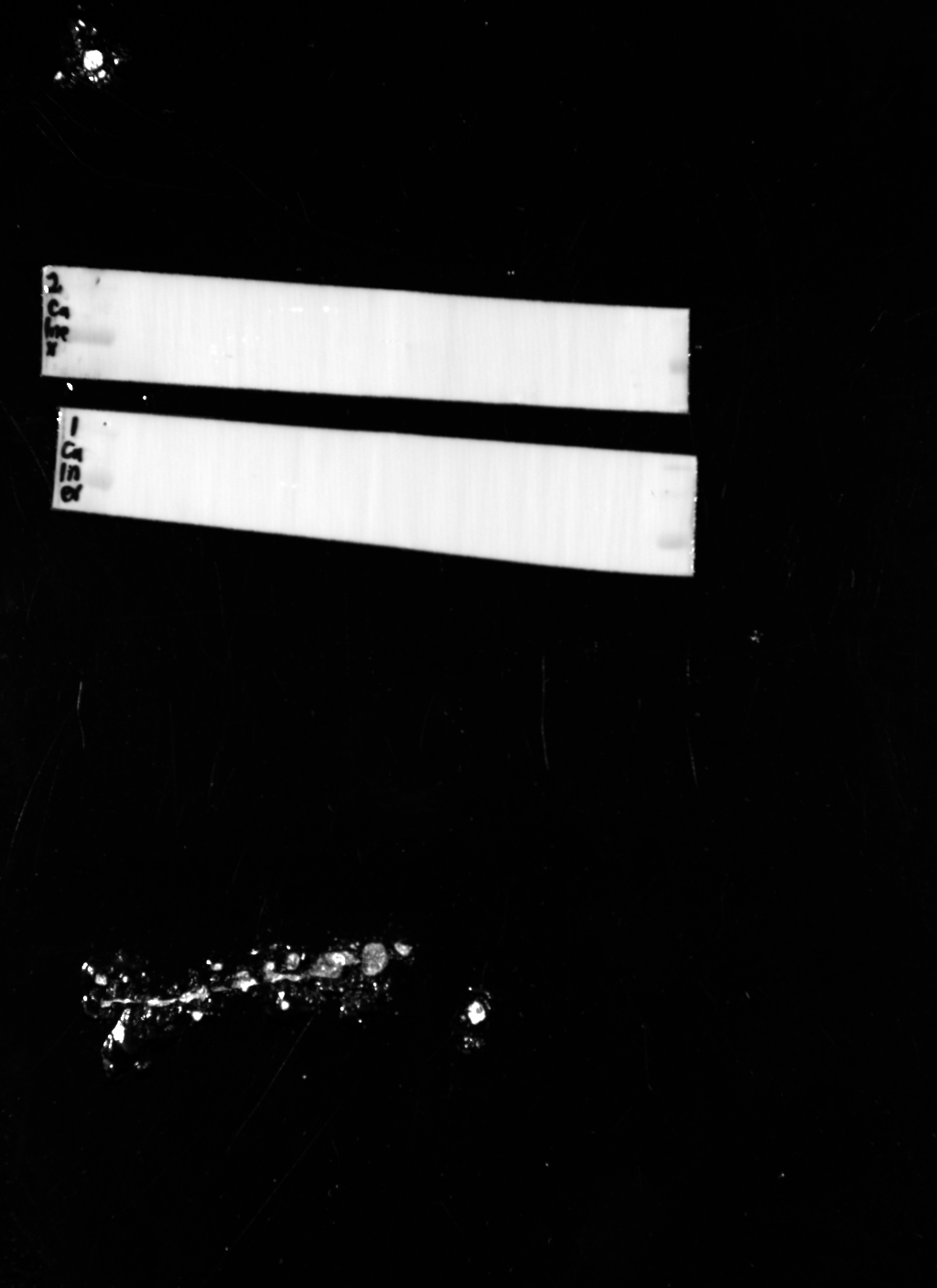

Supplement: Supplemental Information 4 [file peerj-10-13498-s004.zip › 2.Exosome/181202wb/12.2calnex 2018.12.02_18.40.20_Ch/12.2calnex 2018.12.02_18.40.20_Ch-Marker.tif]

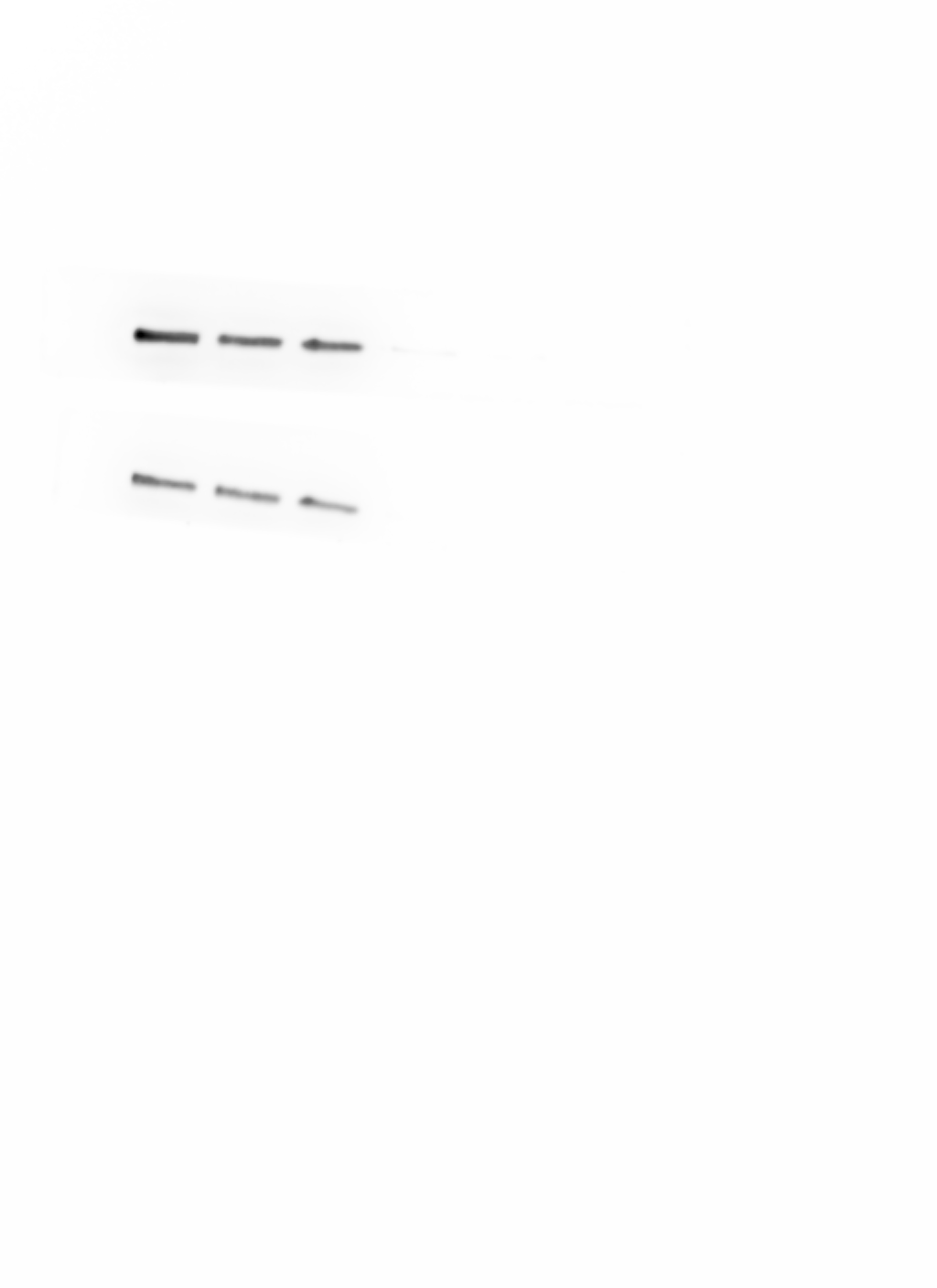

Supplement: Supplemental Information 4 [file peerj-10-13498-s004.zip › 2.Exosome/181202wb/12.2calnex 2018.12.02_18.40.20_Ch/12.2calnex 2018.12.02_18.40.20_Ch.tif]

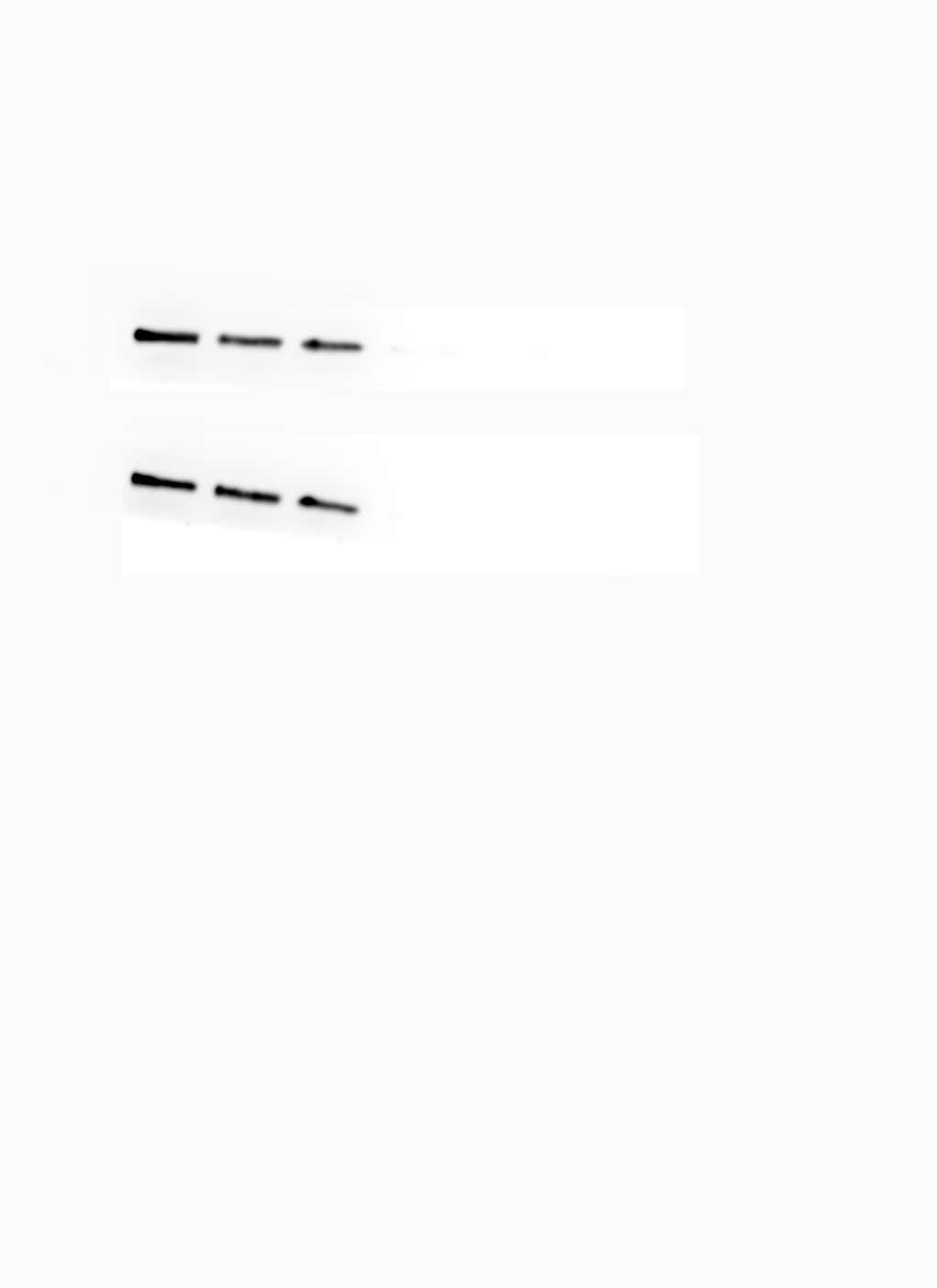

Supplement: Supplemental Information 4 [file peerj-10-13498-s004.zip › 2.Exosome/181202wb/12.2calnex 2018.12.02_18.40.20_Ch/12.2calnex ps.tif]

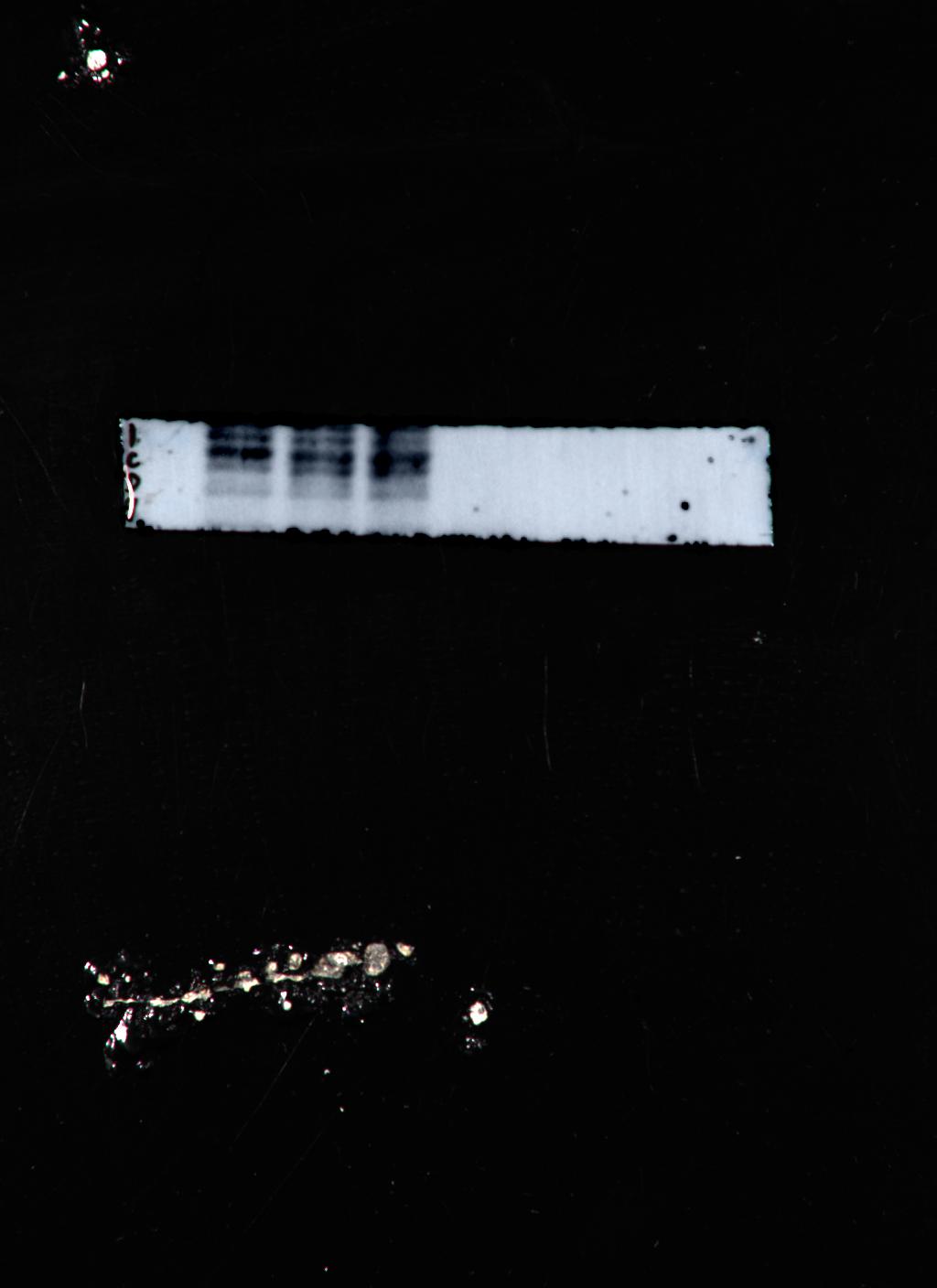

Supplement: Supplemental Information 4 [file peerj-10-13498-s004.zip › 2.Exosome/181202wb/12.2cd9-1 2018.12.02_18.52.36_Ch/12.2cd9-1 2018.12.02_18.52.36_Ch+Marker.jpg]

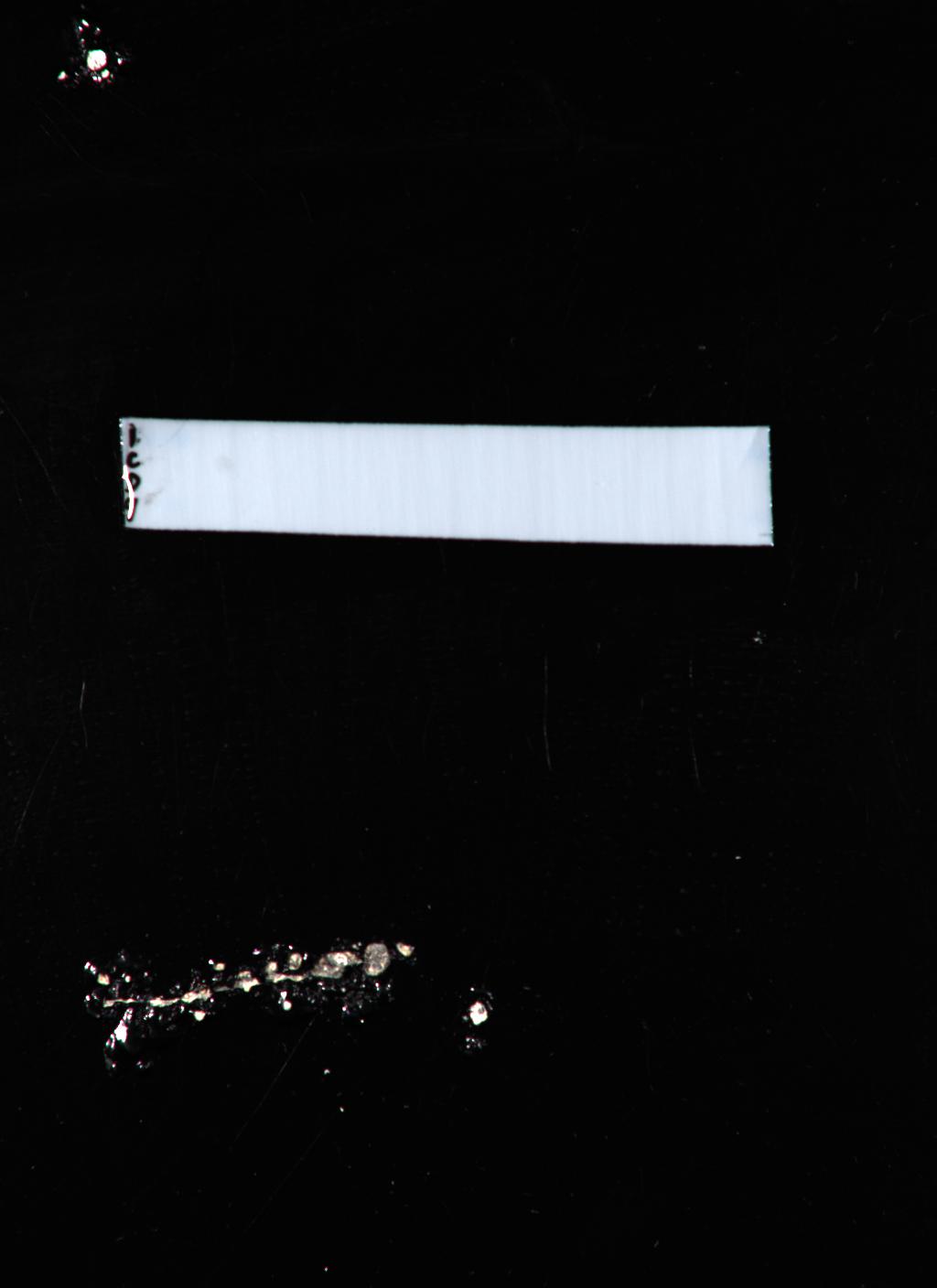

Supplement: Supplemental Information 4 [file peerj-10-13498-s004.zip › 2.Exosome/181202wb/12.2cd9-1 2018.12.02_18.52.36_Ch/12.2cd9-1 2018.12.02_18.52.36_Ch-Marker.jpg]

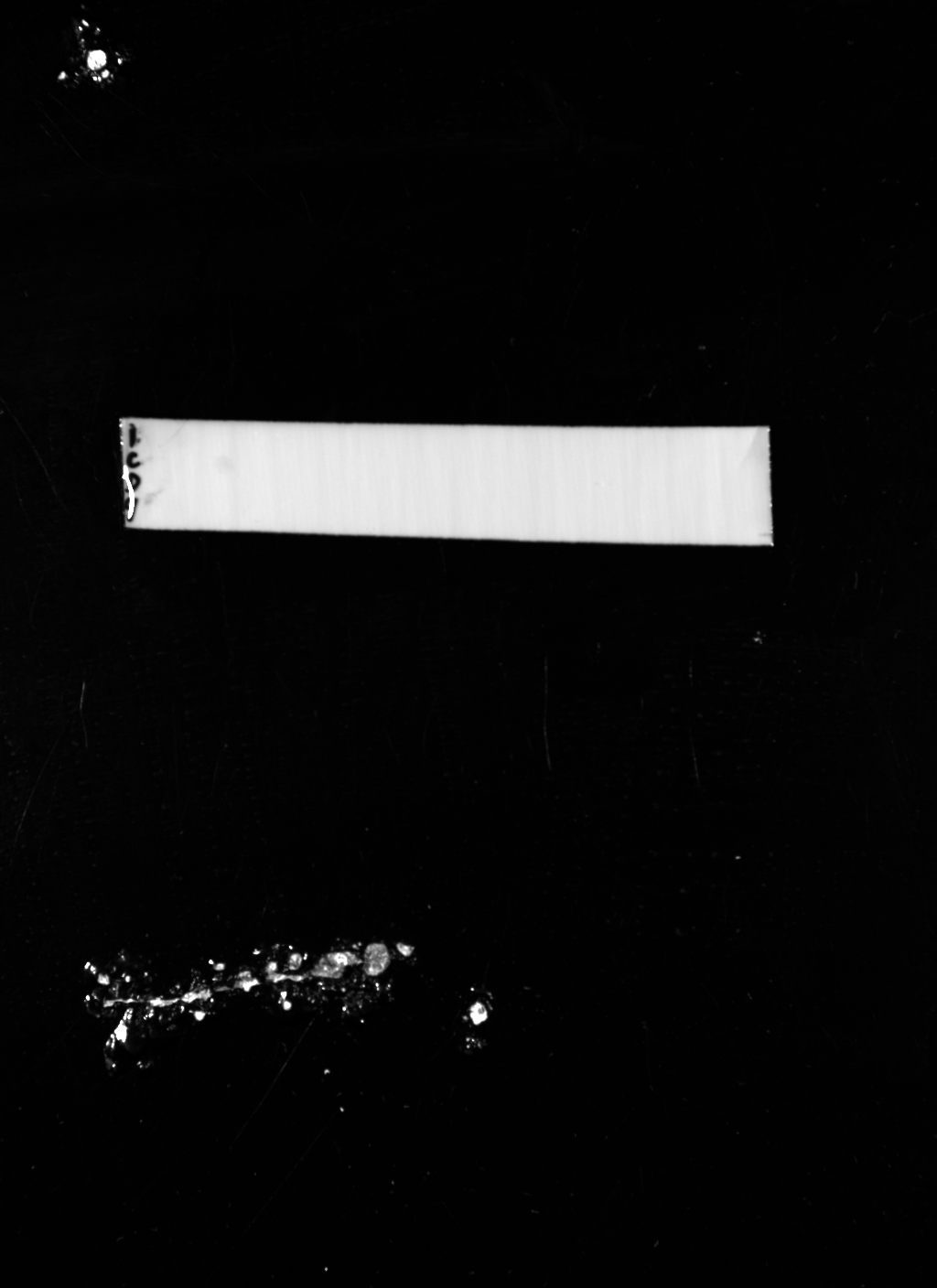

Supplement: Supplemental Information 4 [file peerj-10-13498-s004.zip › 2.Exosome/181202wb/12.2cd9-1 2018.12.02_18.52.36_Ch/12.2cd9-1 2018.12.02_18.52.36_Ch-Marker.tif]

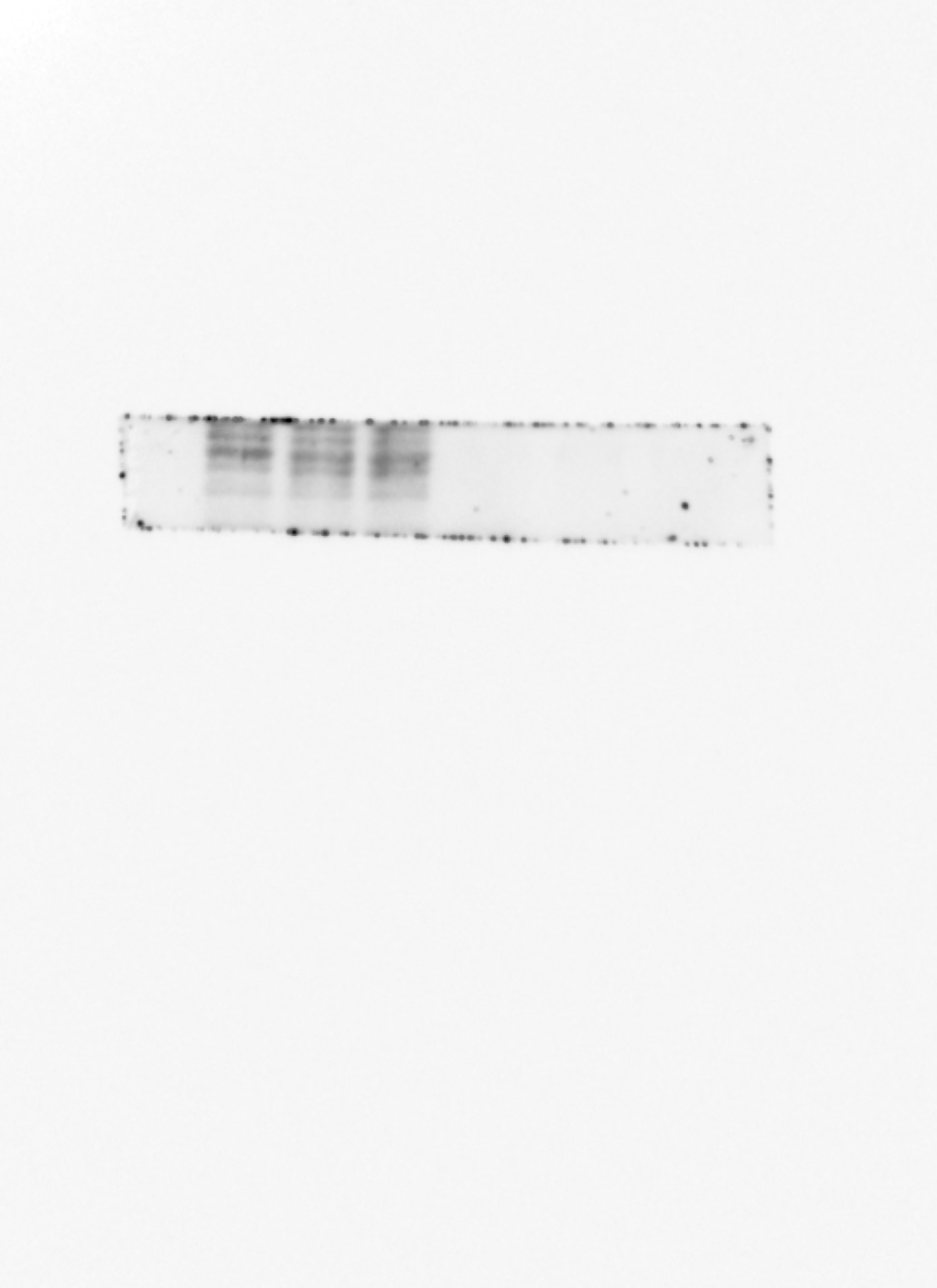

Supplement: Supplemental Information 4 [file peerj-10-13498-s004.zip › 2.Exosome/181202wb/12.2cd9-1 2018.12.02_18.52.36_Ch/12.2cd9-1 2018.12.02_18.52.36_Ch.tif]

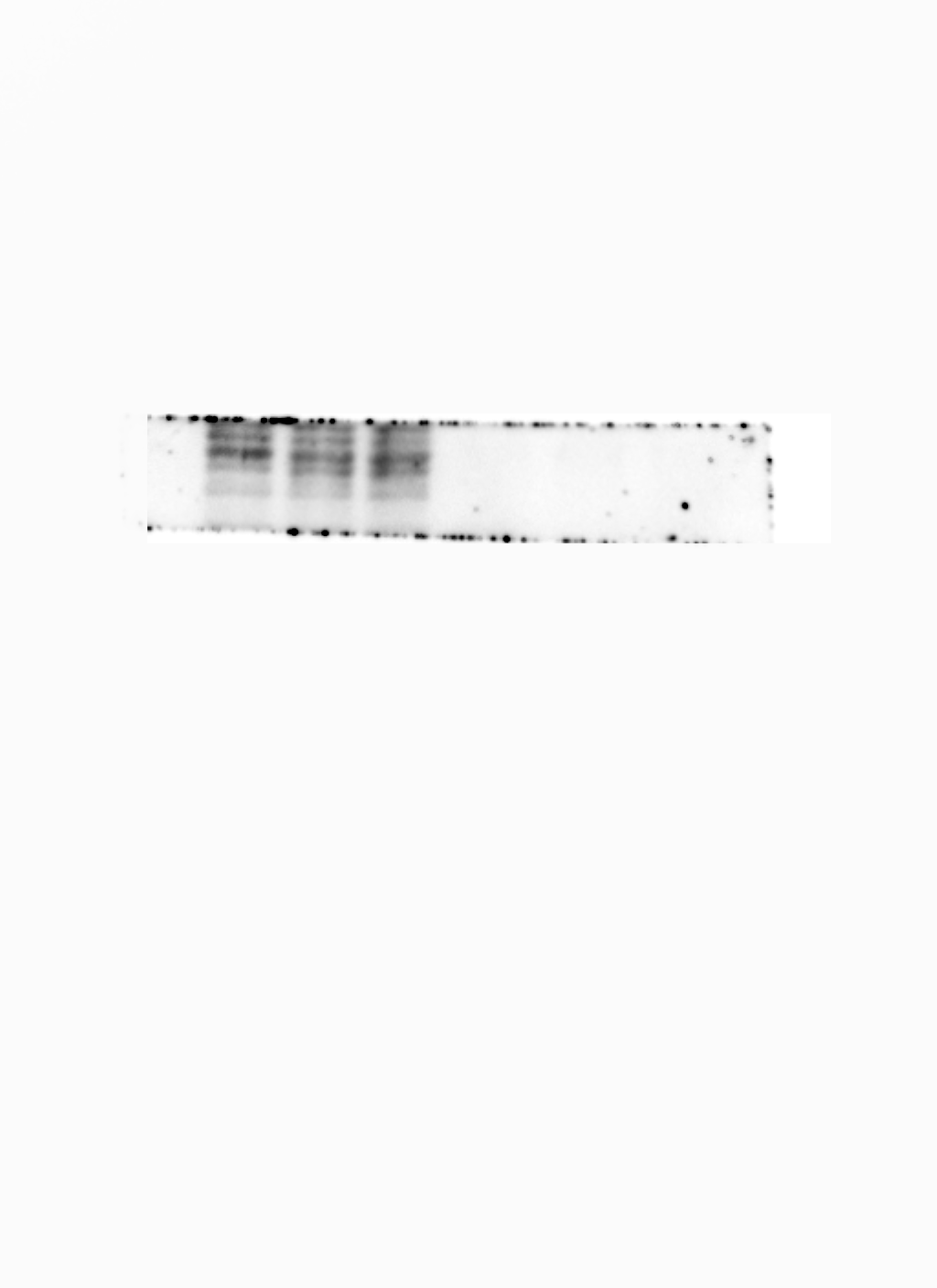

Supplement: Supplemental Information 4 [file peerj-10-13498-s004.zip › 2.Exosome/181202wb/12.2cd9-1 2018.12.02_18.52.36_Ch/12.2cd9-1 ps_Ch.tif]

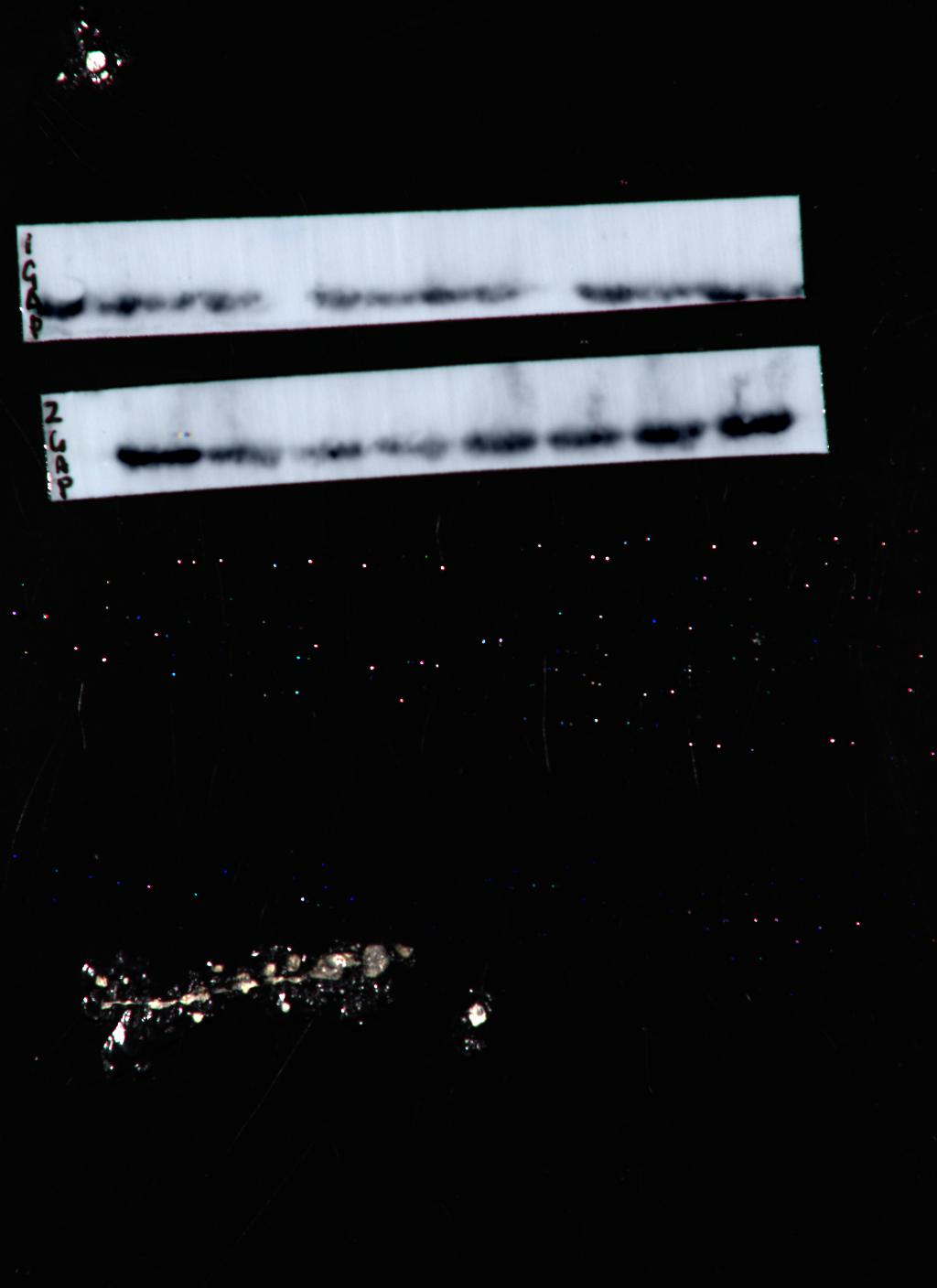

Supplement: Supplemental Information 4 [file peerj-10-13498-s004.zip › 2.Exosome/181202wb/12.2gap 2018.12.02_19.11.12_Ch/12.2gap 2018.12.02_19.11.12_Ch+Marker.jpg]

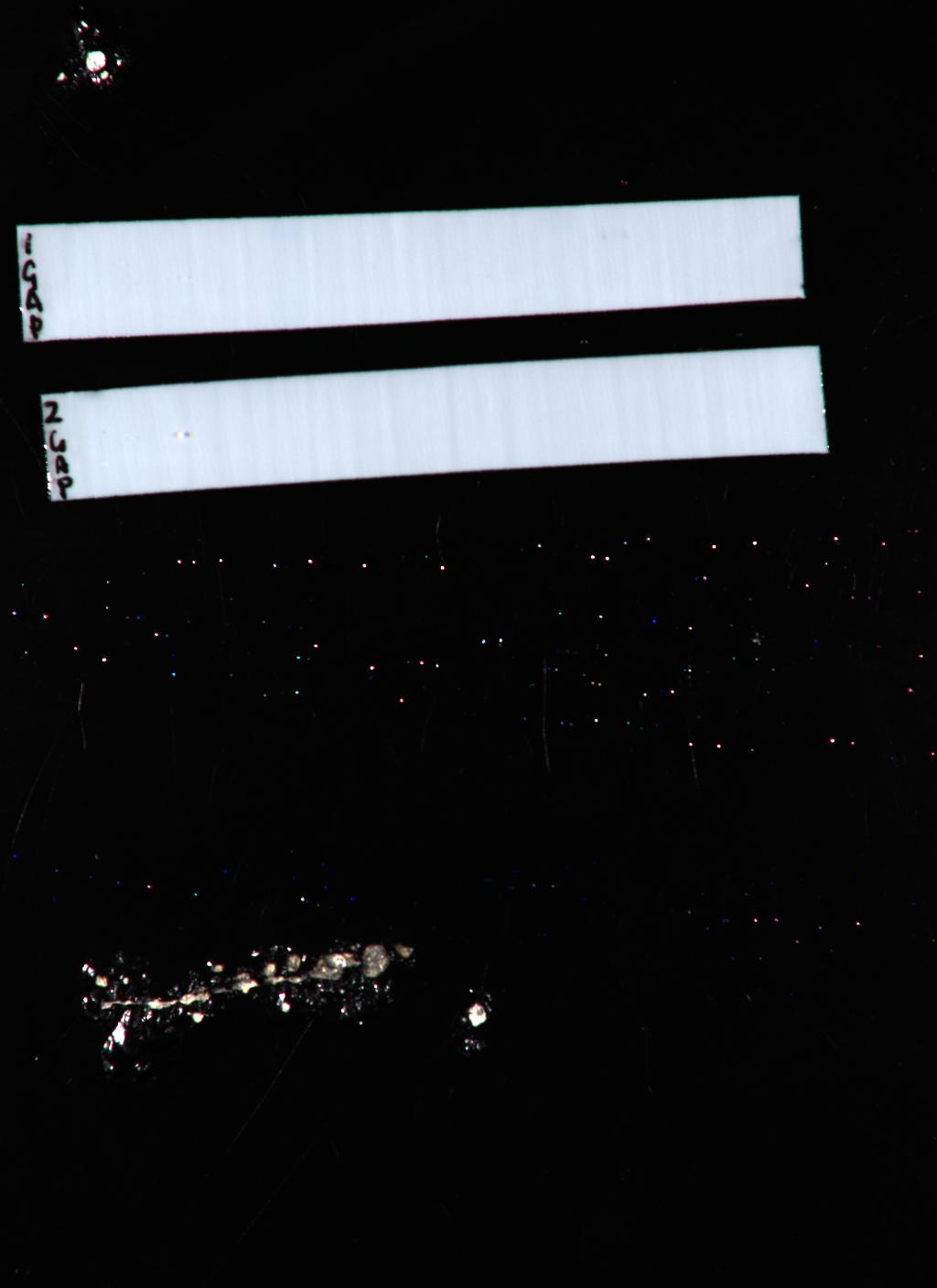

Supplement: Supplemental Information 4 [file peerj-10-13498-s004.zip › 2.Exosome/181202wb/12.2gap 2018.12.02_19.11.12_Ch/12.2gap 2018.12.02_19.11.12_Ch-Marker.jpg]

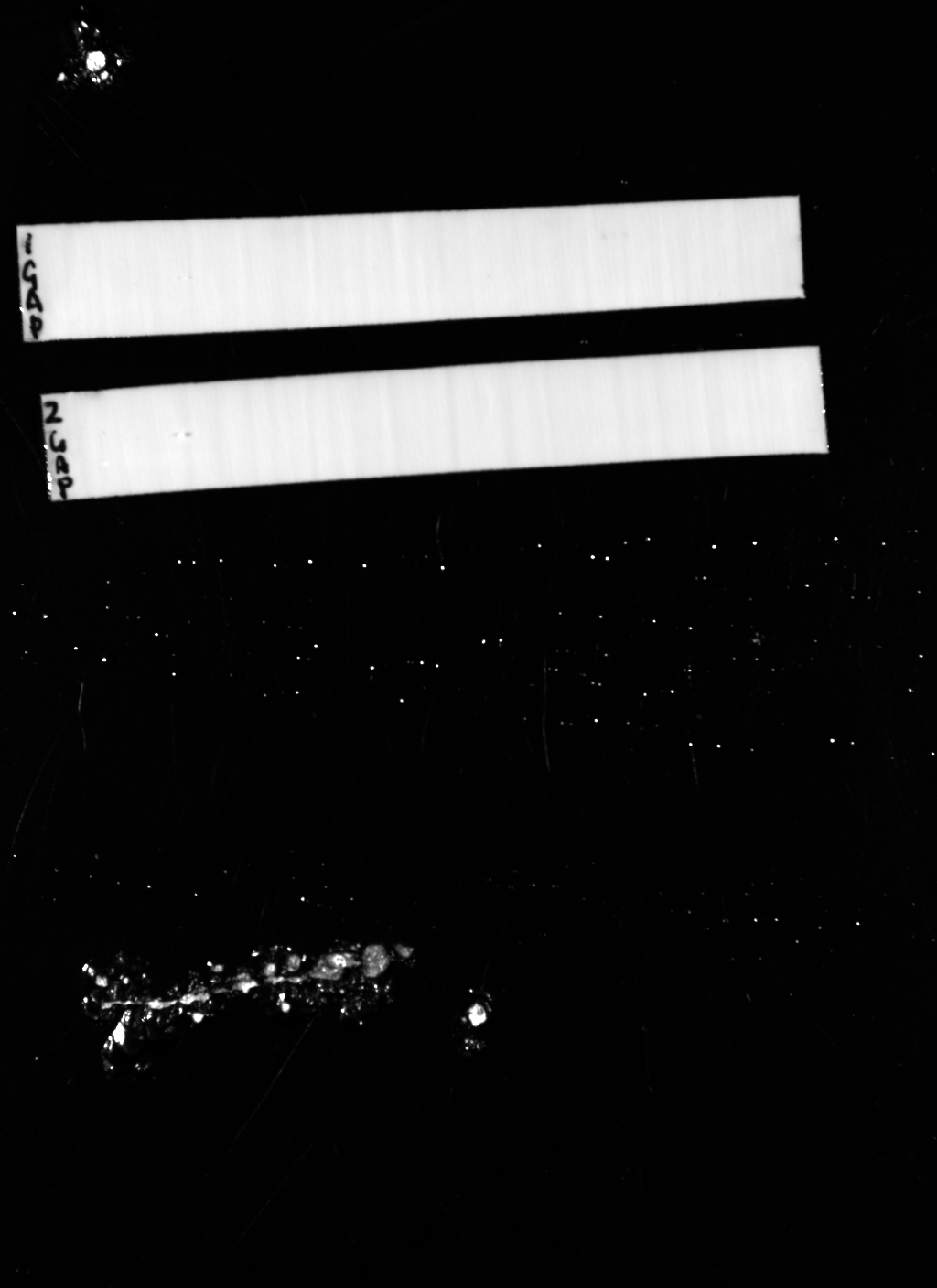

Supplement: Supplemental Information 4 [file peerj-10-13498-s004.zip › 2.Exosome/181202wb/12.2gap 2018.12.02_19.11.12_Ch/12.2gap 2018.12.02_19.11.12_Ch-Marker.tif]

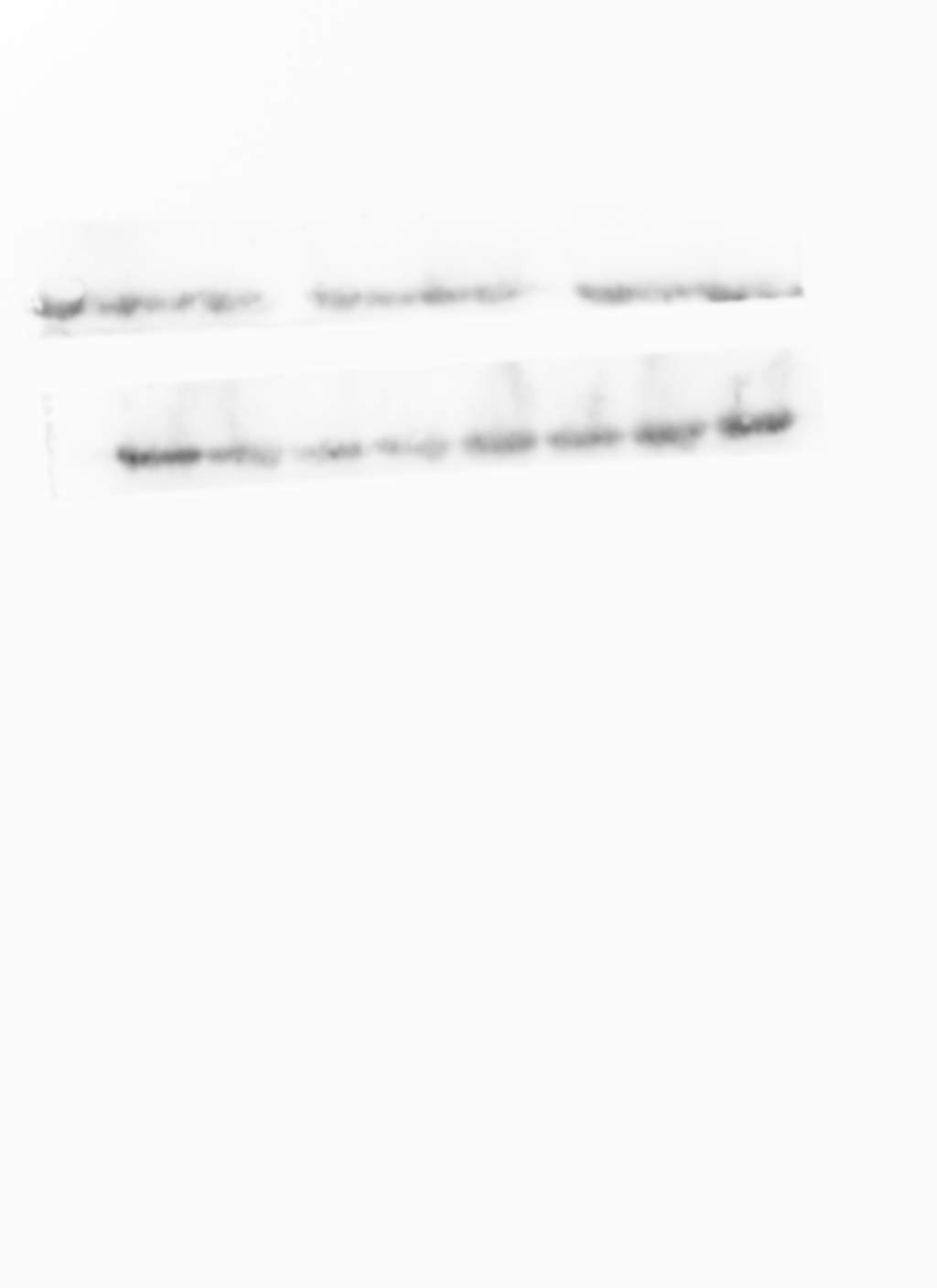

Supplement: Supplemental Information 4 [file peerj-10-13498-s004.zip › 2.Exosome/181202wb/12.2gap 2018.12.02_19.11.12_Ch/12.2gap 2018.12.02_19.11.12_Ch.tif]

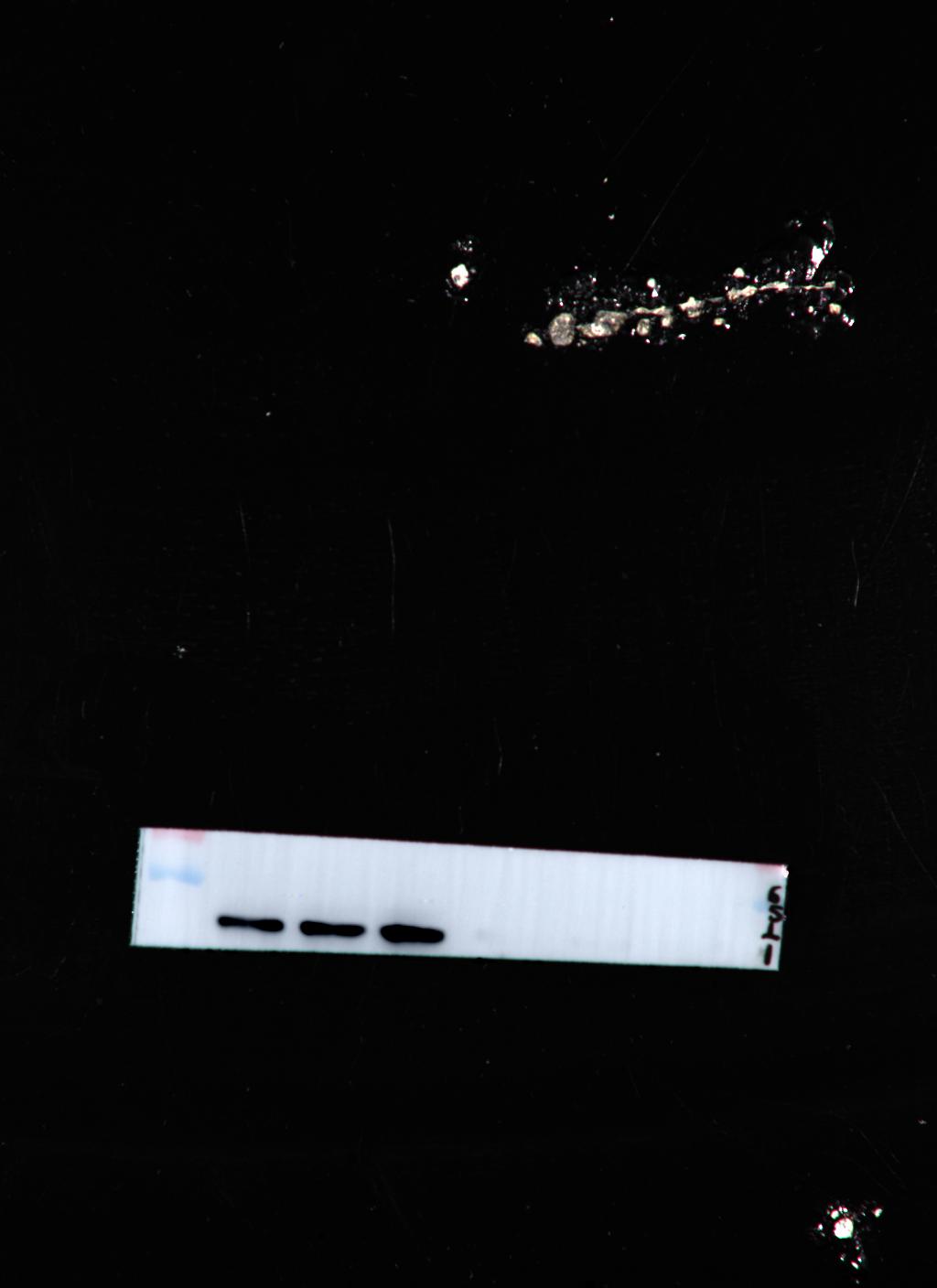

Supplement: Supplemental Information 4 [file peerj-10-13498-s004.zip › 2.Exosome/181202wb/12.2tsg101-1 2018.12.02_18.47.40_Ch/12.2tsg101-1 2018.12.02_18.47.40_Ch+Marker.jpg]

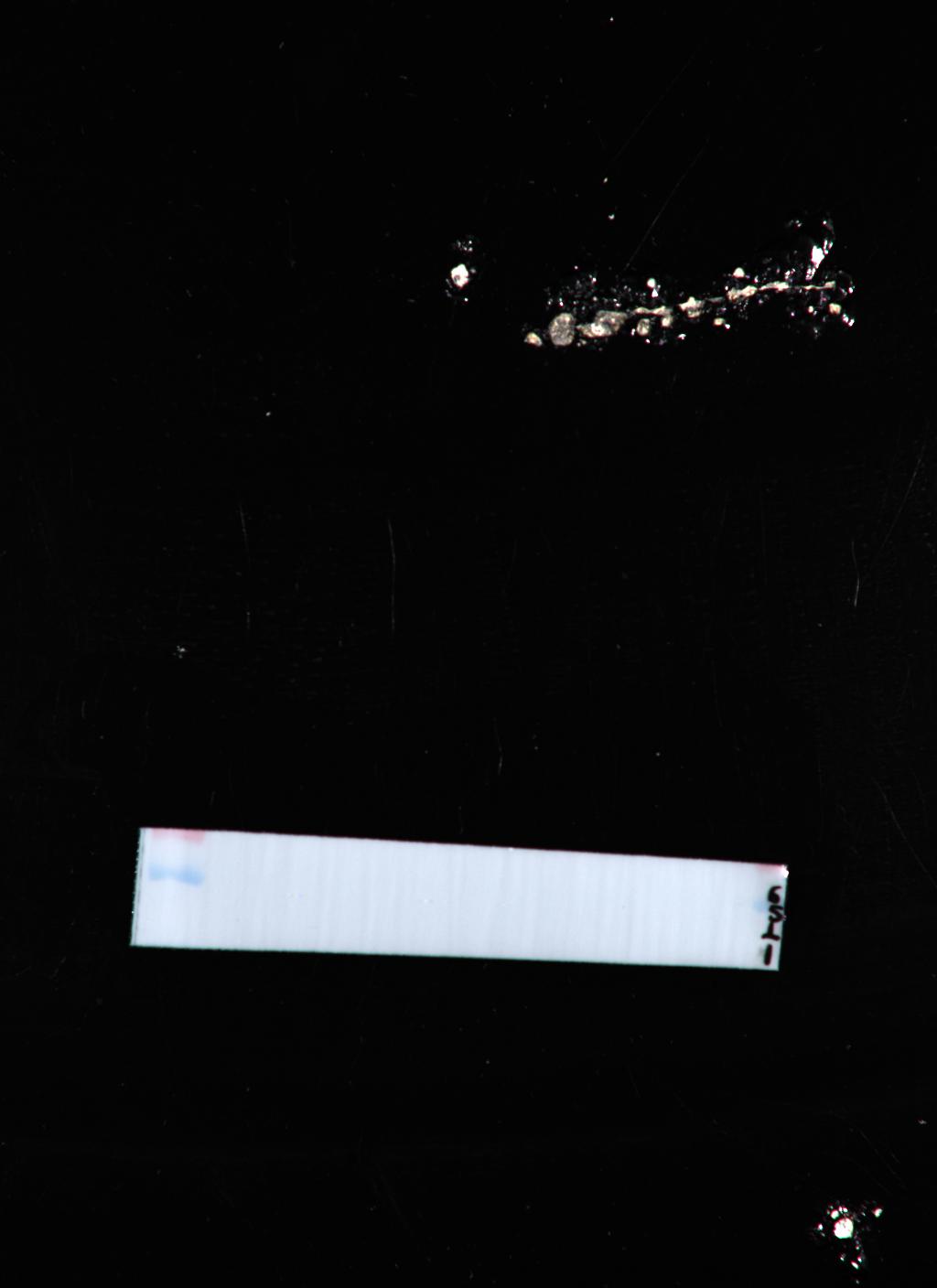

Supplement: Supplemental Information 4 [file peerj-10-13498-s004.zip › 2.Exosome/181202wb/12.2tsg101-1 2018.12.02_18.47.40_Ch/12.2tsg101-1 2018.12.02_18.47.40_Ch-Marker.jpg]

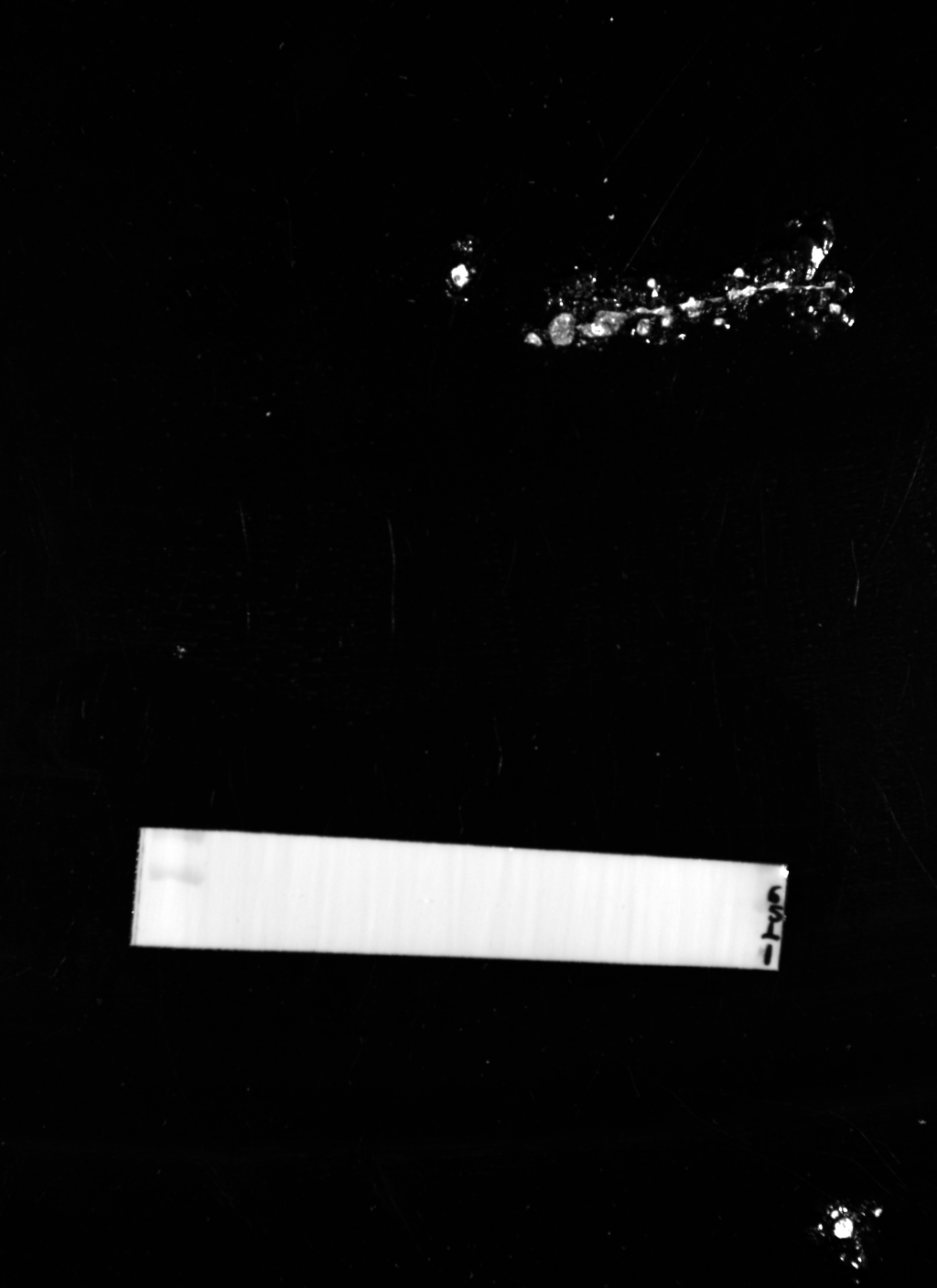

Supplement: Supplemental Information 4 [file peerj-10-13498-s004.zip › 2.Exosome/181202wb/12.2tsg101-1 2018.12.02_18.47.40_Ch/12.2tsg101-1 2018.12.02_18.47.40_Ch-Marker.tif]

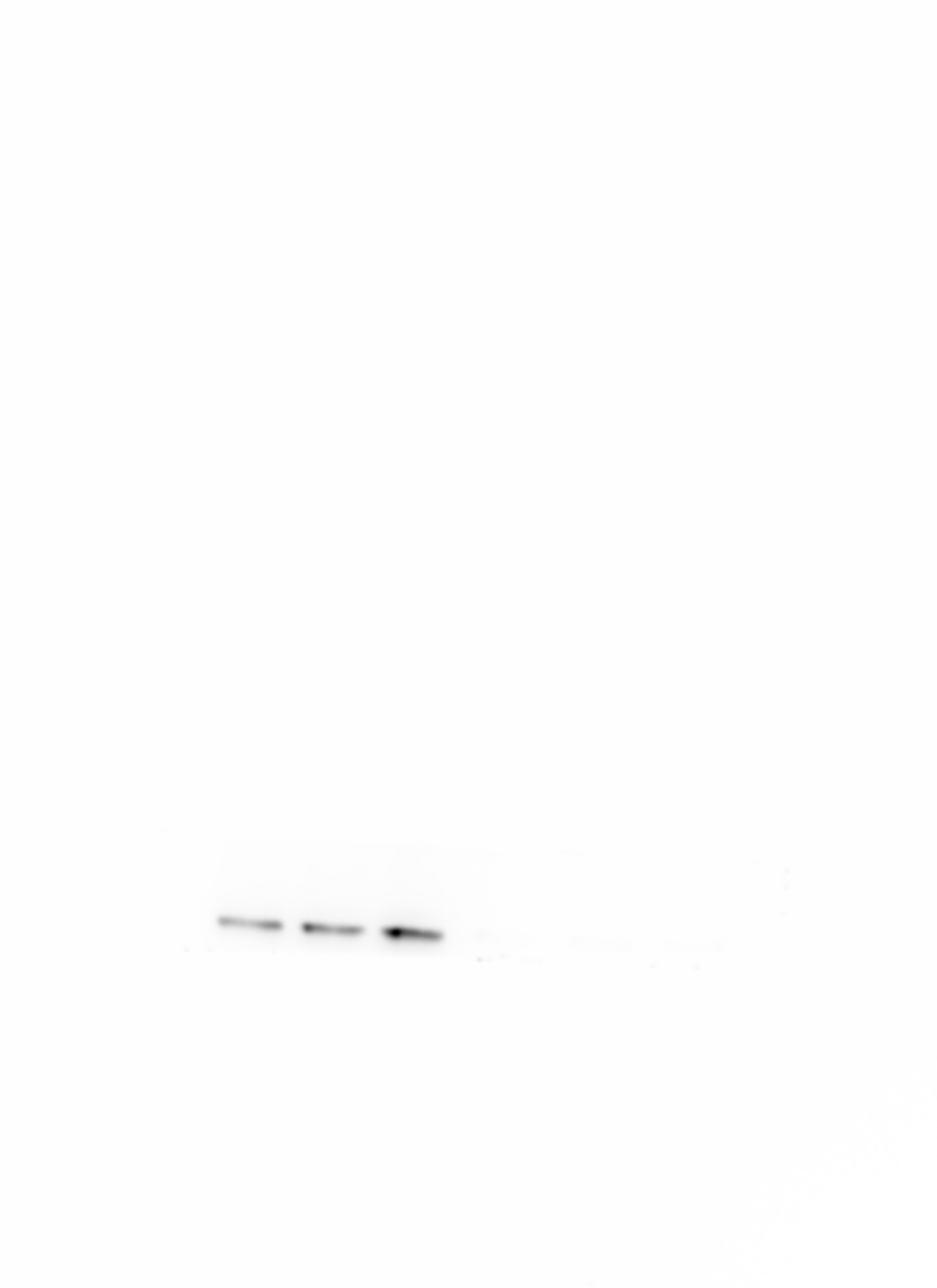

Supplement: Supplemental Information 4 [file peerj-10-13498-s004.zip › 2.Exosome/181202wb/12.2tsg101-1 2018.12.02_18.47.40_Ch/12.2tsg101-1 2018.12.02_18.47.40_Ch.tif]

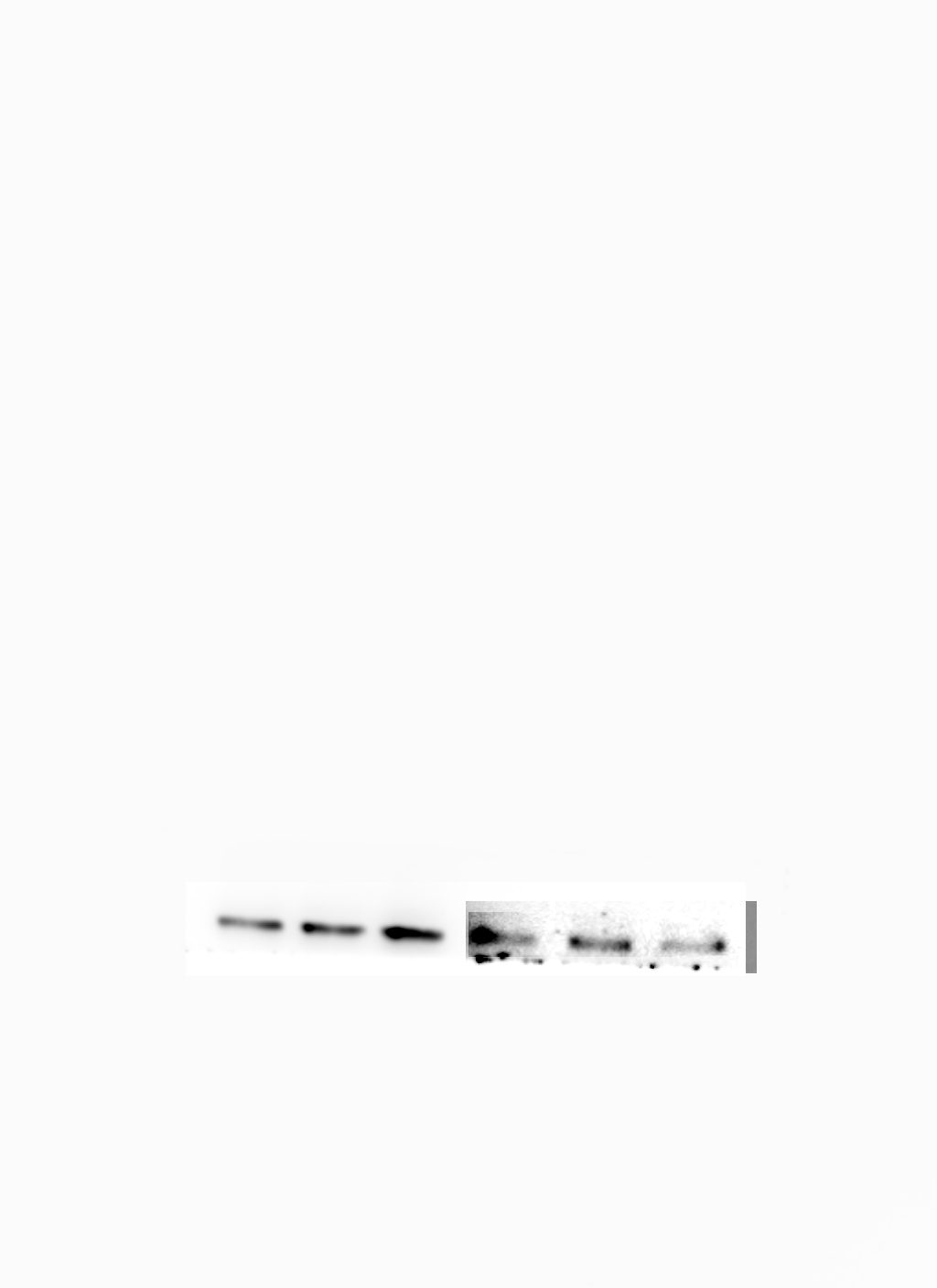

Supplement: Supplemental Information 4 [file peerj-10-13498-s004.zip › 2.Exosome/181202wb/12.2tsg101-1 2018.12.02_18.47.40_Ch/12.2tsg101-1 2psh.tif]

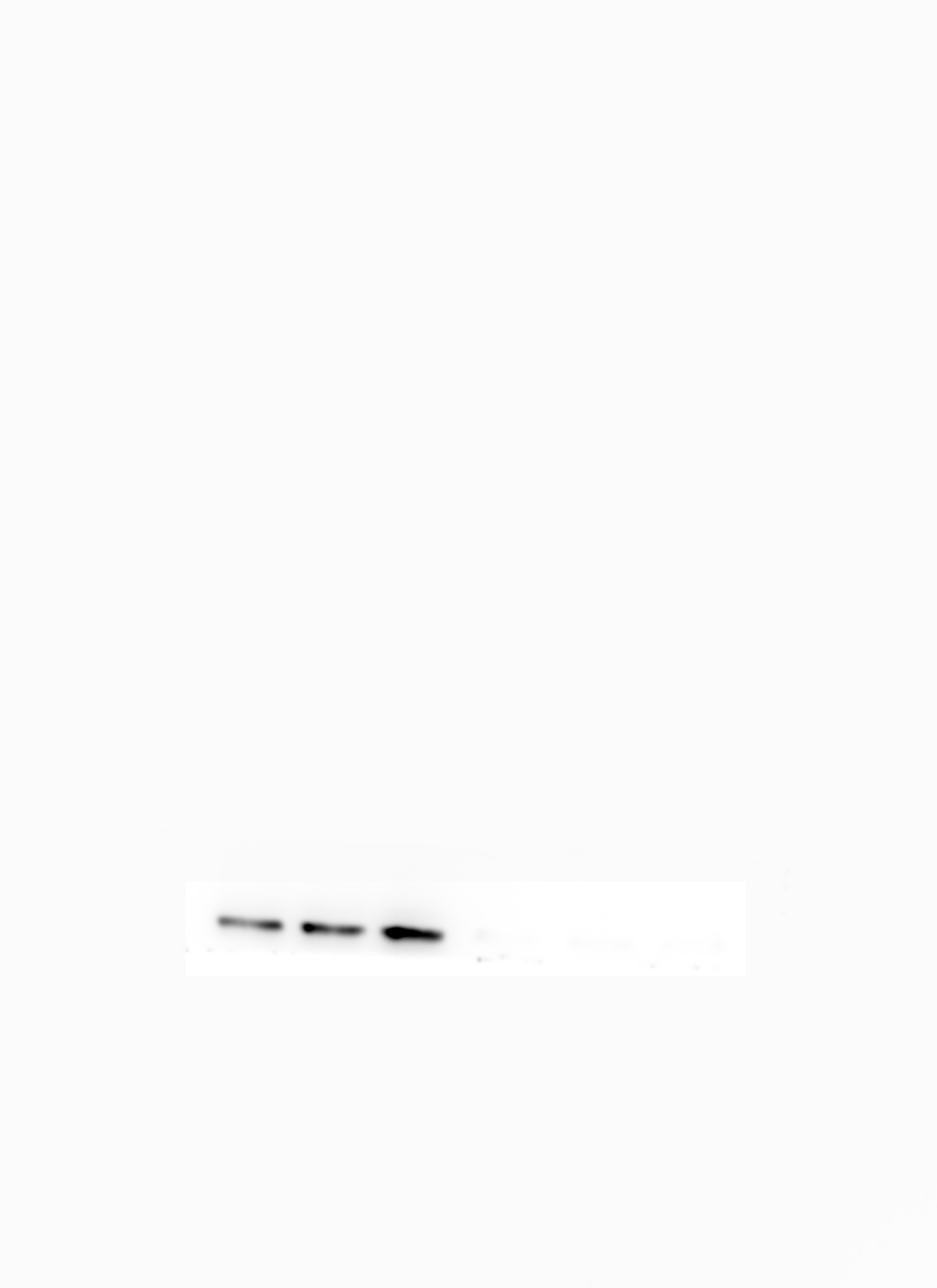

Supplement: Supplemental Information 4 [file peerj-10-13498-s004.zip › 2.Exosome/181202wb/12.2tsg101-1 2018.12.02_18.47.40_Ch/12.2tsg101-1 psh.tif]

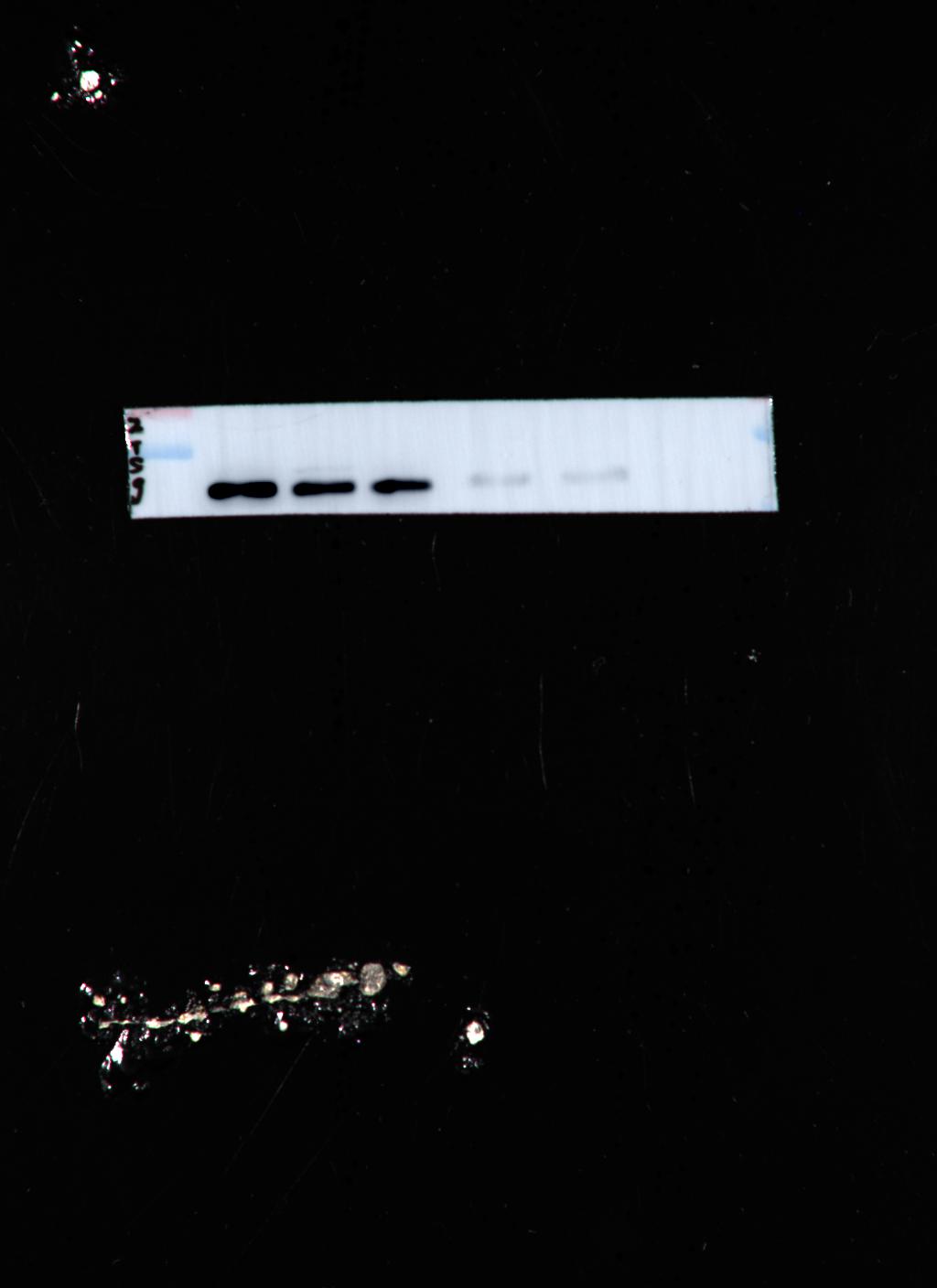

Supplement: Supplemental Information 4 [file peerj-10-13498-s004.zip › 2.Exosome/181202wb/12.2tsg101-2 2018.12.02_18.43.57_Ch/12.2tsg101-2 2018.12.02_18.43.57_Ch+Marker.jpg]

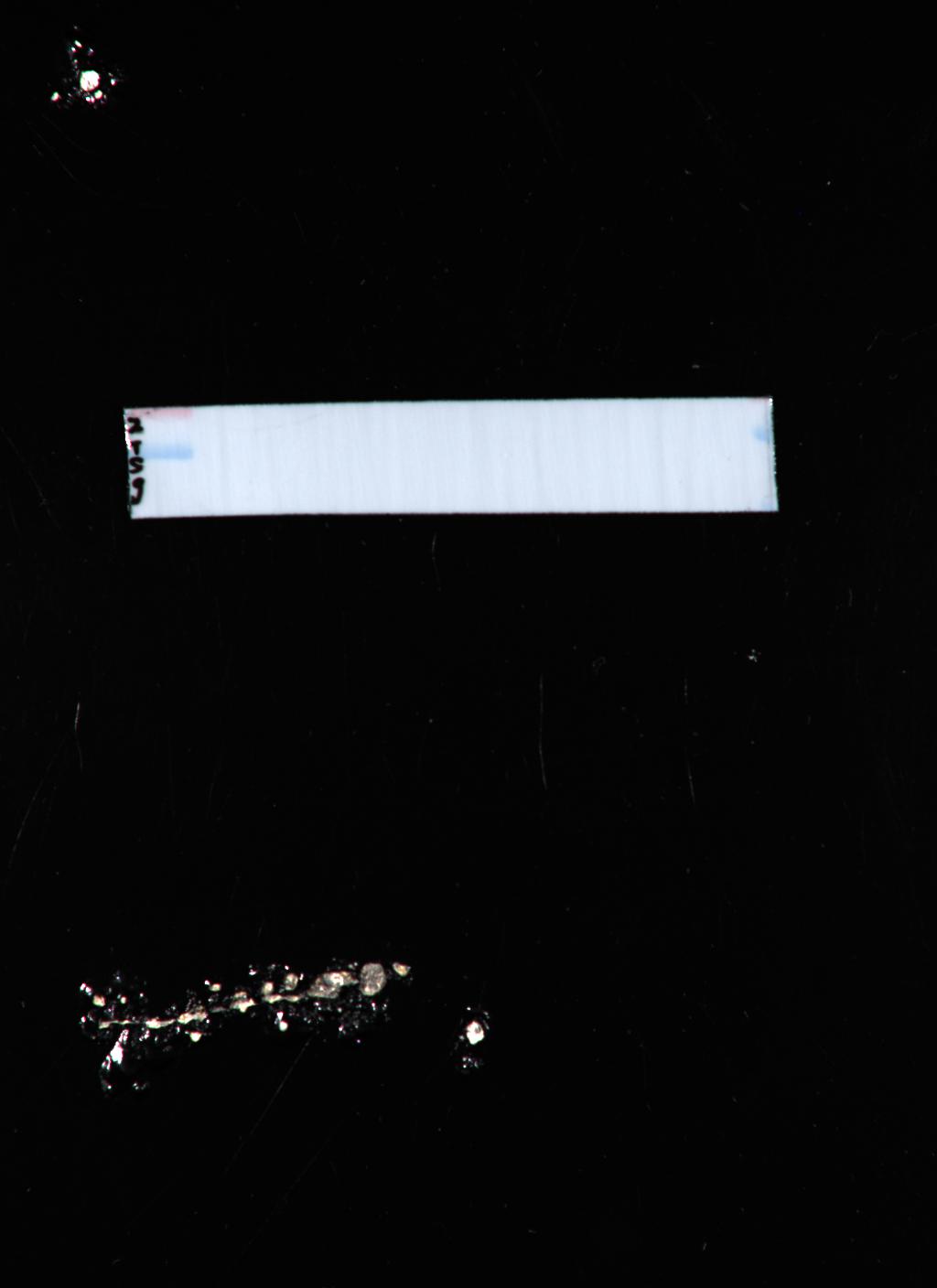

Supplement: Supplemental Information 4 [file peerj-10-13498-s004.zip › 2.Exosome/181202wb/12.2tsg101-2 2018.12.02_18.43.57_Ch/12.2tsg101-2 2018.12.02_18.43.57_Ch-Marker.jpg]

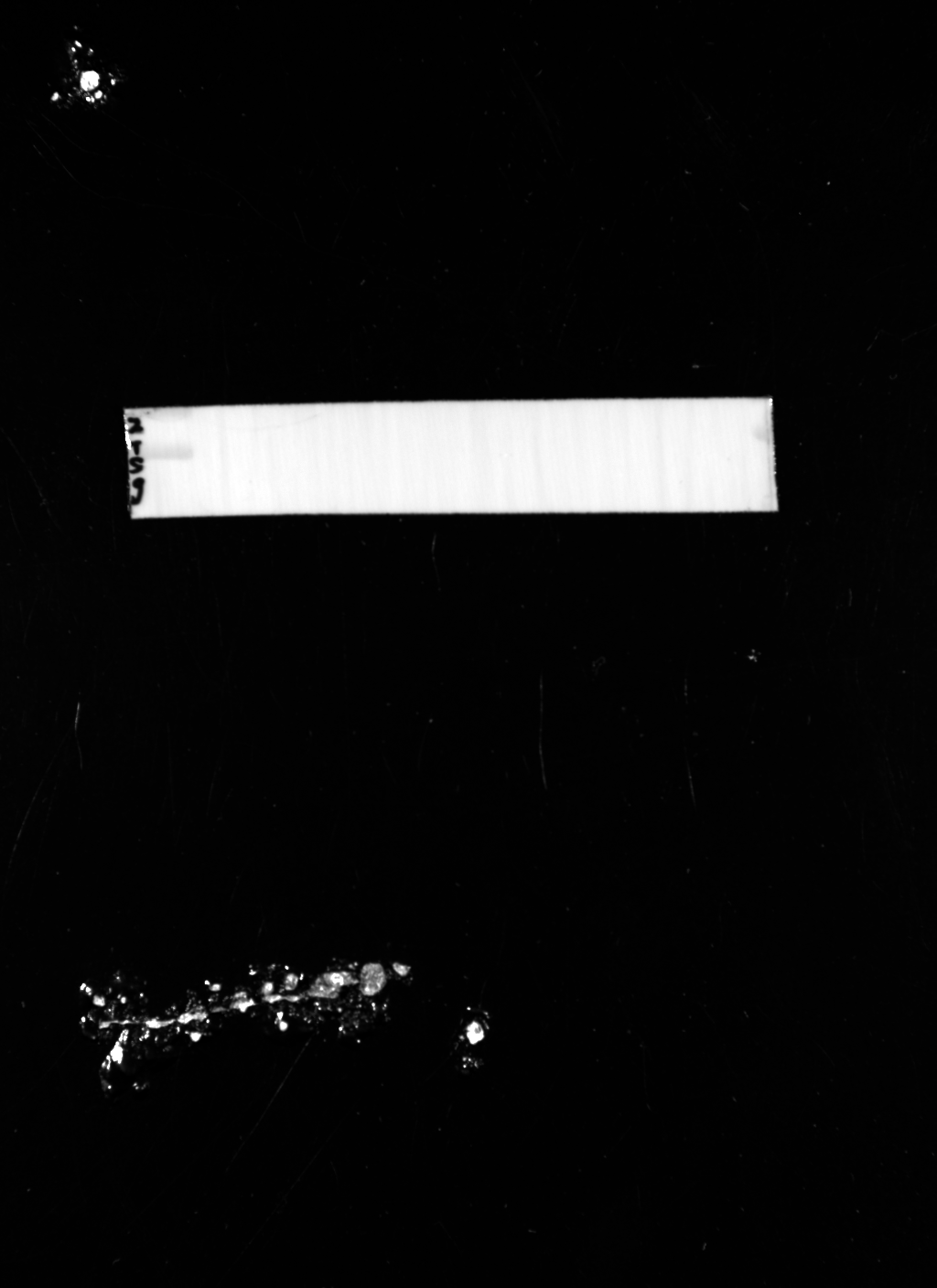

Supplement: Supplemental Information 4 [file peerj-10-13498-s004.zip › 2.Exosome/181202wb/12.2tsg101-2 2018.12.02_18.43.57_Ch/12.2tsg101-2 2018.12.02_18.43.57_Ch-Marker.tif]

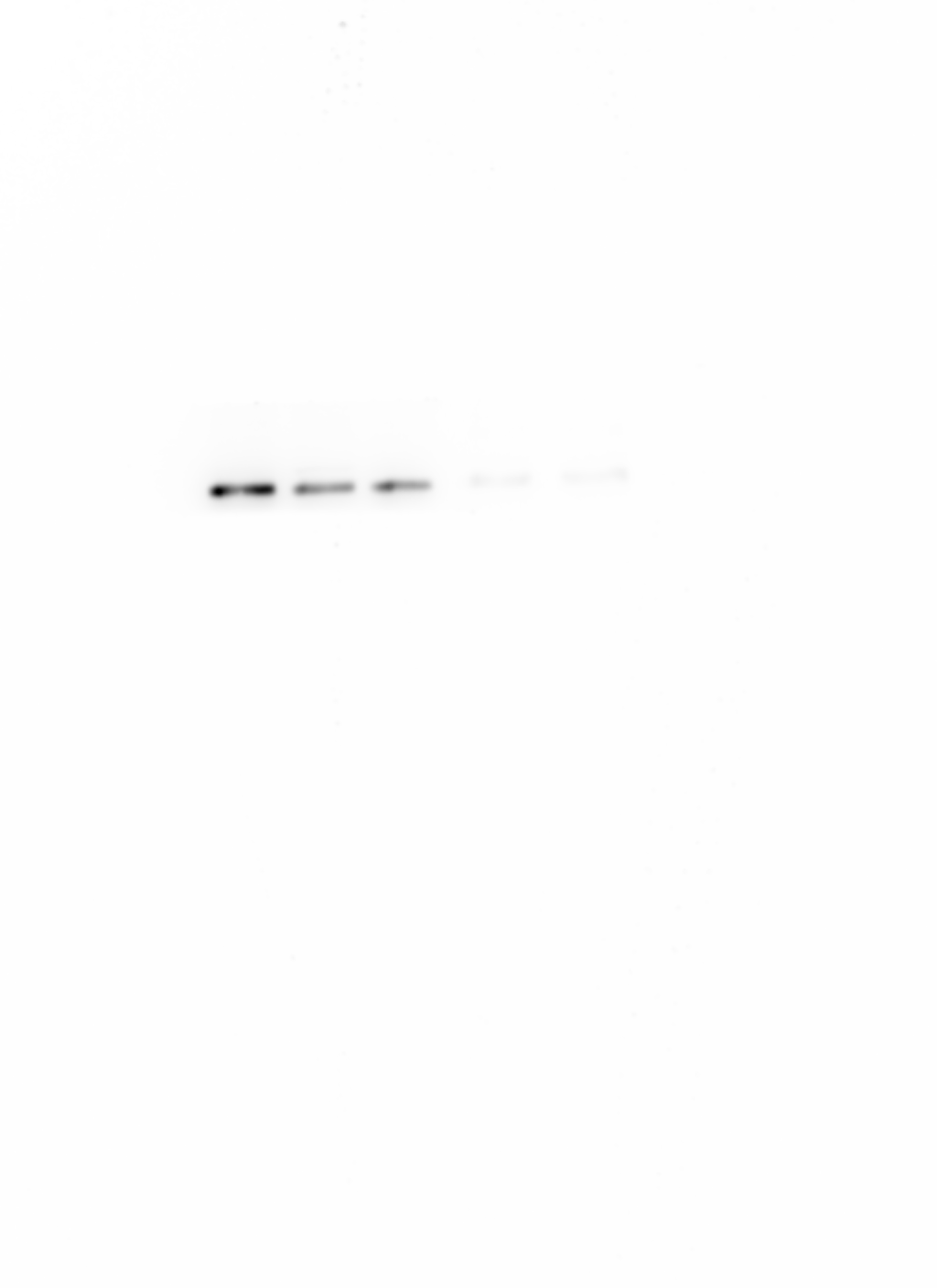

Supplement: Supplemental Information 4 [file peerj-10-13498-s004.zip › 2.Exosome/181202wb/12.2tsg101-2 2018.12.02_18.43.57_Ch/12.2tsg101-2 2018.12.02_18.43.57_Ch.tif]

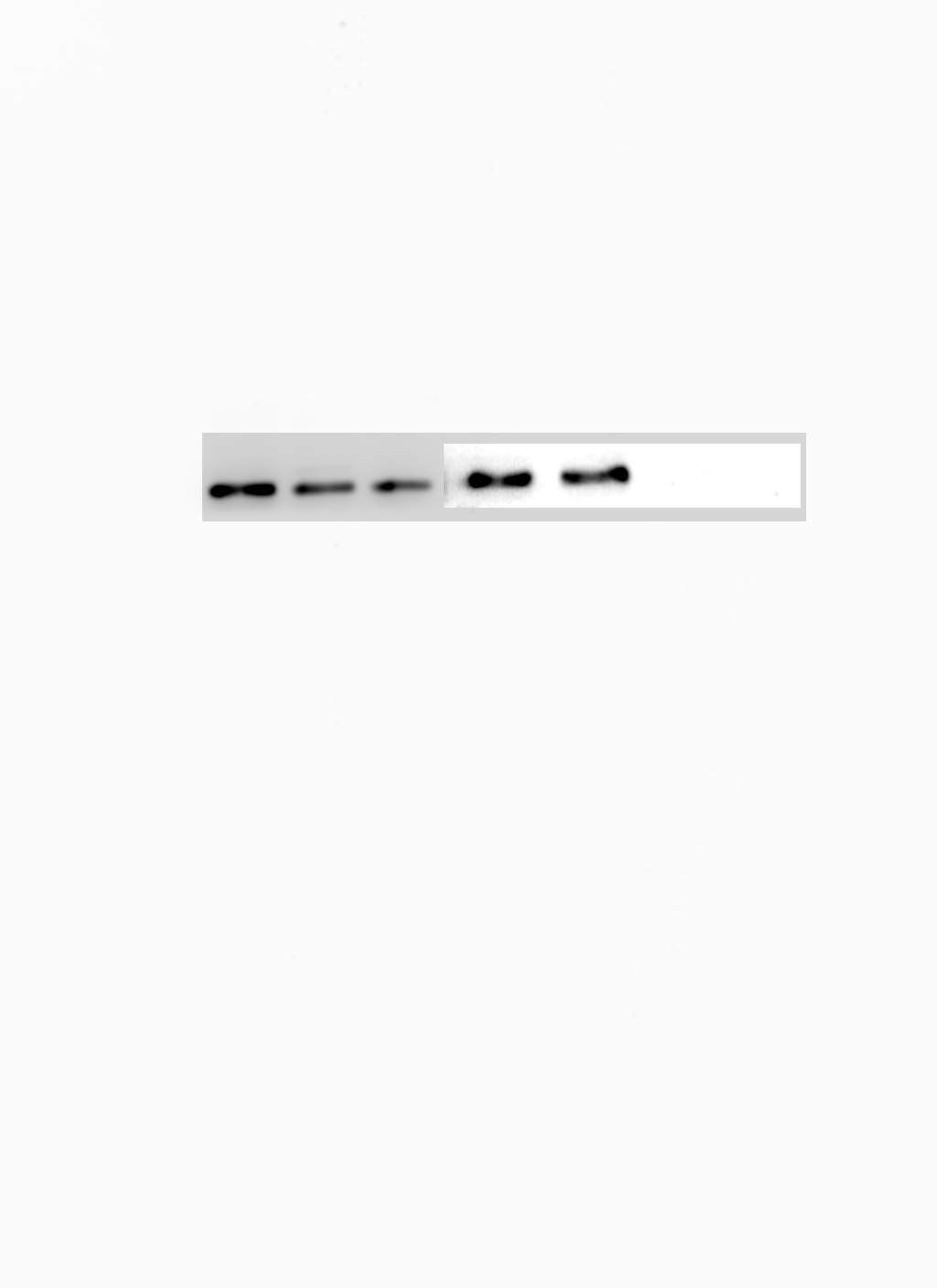

Supplement: Supplemental Information 4 [file peerj-10-13498-s004.zip › 2.Exosome/181202wb/12.2tsg101-2 2018.12.02_18.43.57_Ch/12.2tsg101-2ps.tif]

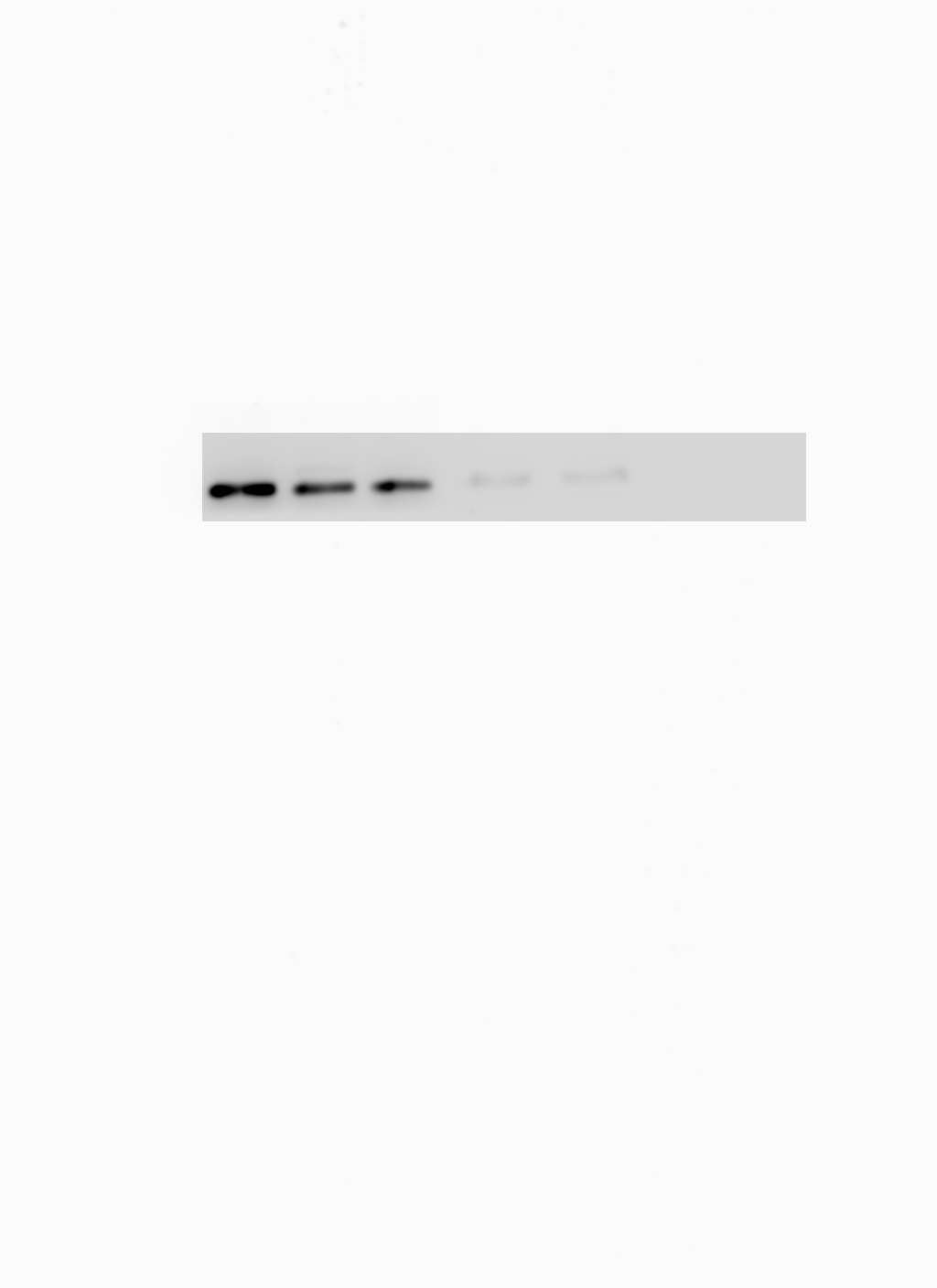

Supplement: Supplemental Information 4 [file peerj-10-13498-s004.zip › 2.Exosome/181202wb/12.2tsg101-2 2018.12.02_18.43.57_Ch/12.2tsg101-2ps_Ch.tif]

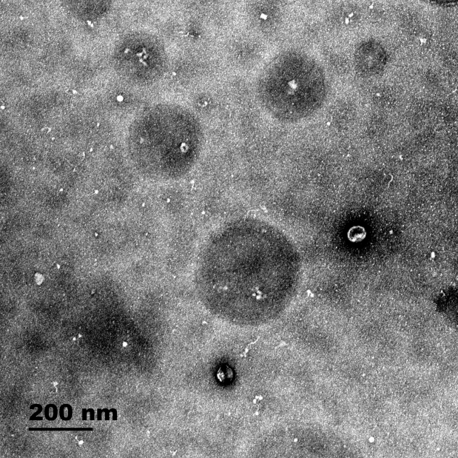

Supplement: Supplemental Information 4 [file peerj-10-13498-s004.zip › 2.Exosome/electronic speculum-U937-con-200nm.tif]

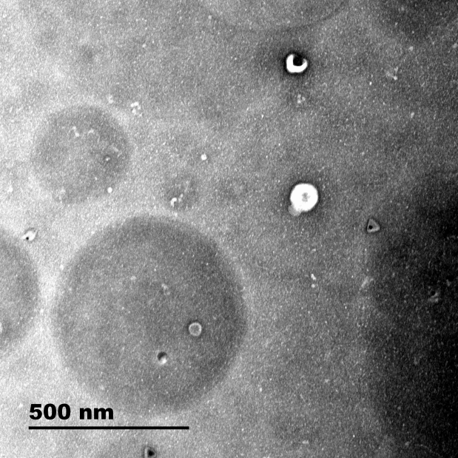

Supplement: Supplemental Information 4 [file peerj-10-13498-s004.zip › 2.Exosome/electronic speculum-U937-con-500nm.tif]

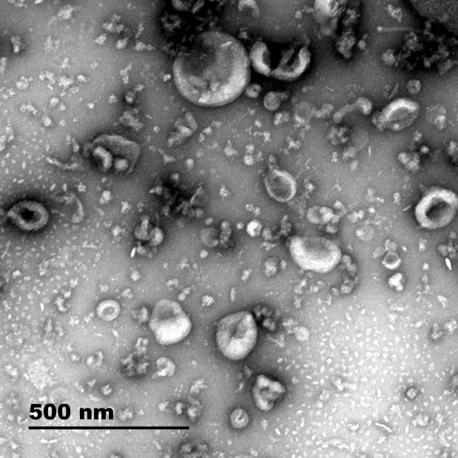

Supplement: Supplemental Information 4 [file peerj-10-13498-s004.zip › 2.Exosome/electronic speculum-U937-p62-00nm.tif]

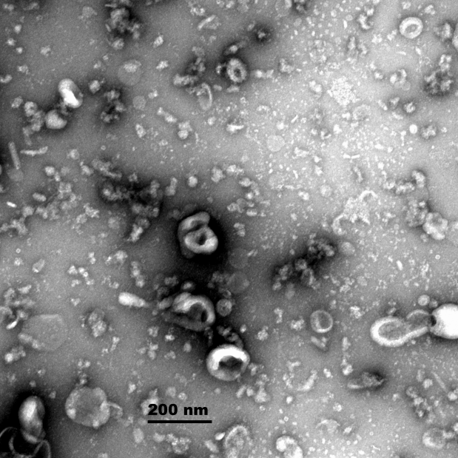

Supplement: Supplemental Information 4 [file peerj-10-13498-s004.zip › 2.Exosome/electronic speculum-U937-p62-200nm.tif]

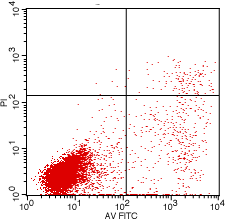

Supplement: Supplemental Information 5 [file peerj-10-13498-s005.zip › 4.flow cytometry/LC1126/u937-2.tif]

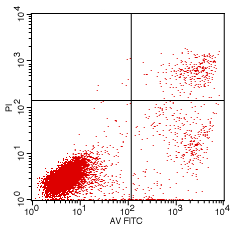

Supplement: Supplemental Information 5 [file peerj-10-13498-s005.zip › 4.flow cytometry/LC1130/con-1.tif]

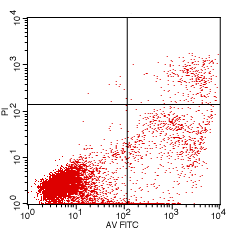

Supplement: Supplemental Information 5 [file peerj-10-13498-s005.zip › 4.flow cytometry/LC1130/p621.tif]

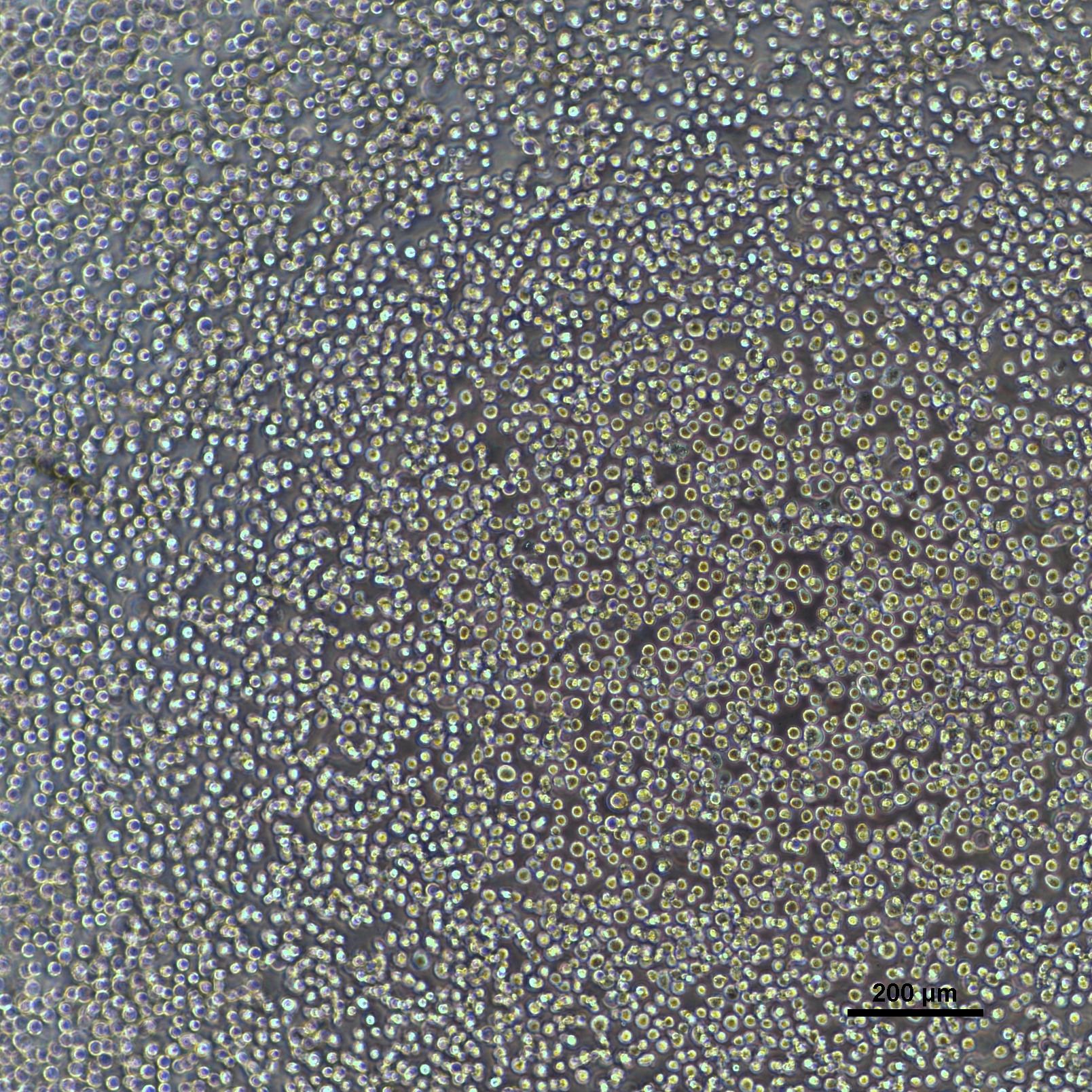

Supplement: Supplemental Information 8 [file peerj-10-13498-s008.zip › 0h/h1.tif]

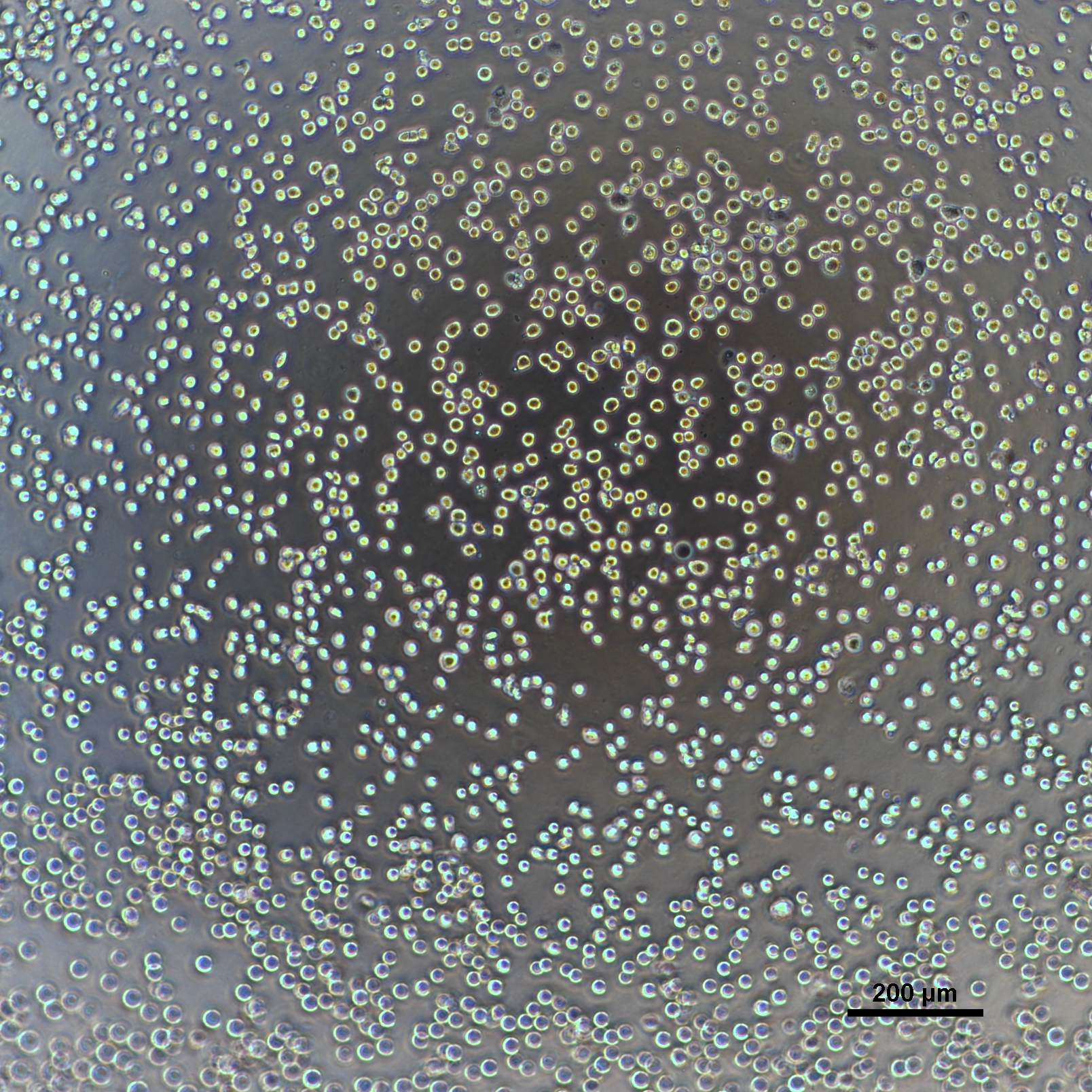

Supplement: Supplemental Information 8 [file peerj-10-13498-s008.zip › 0h/hce1.tif]

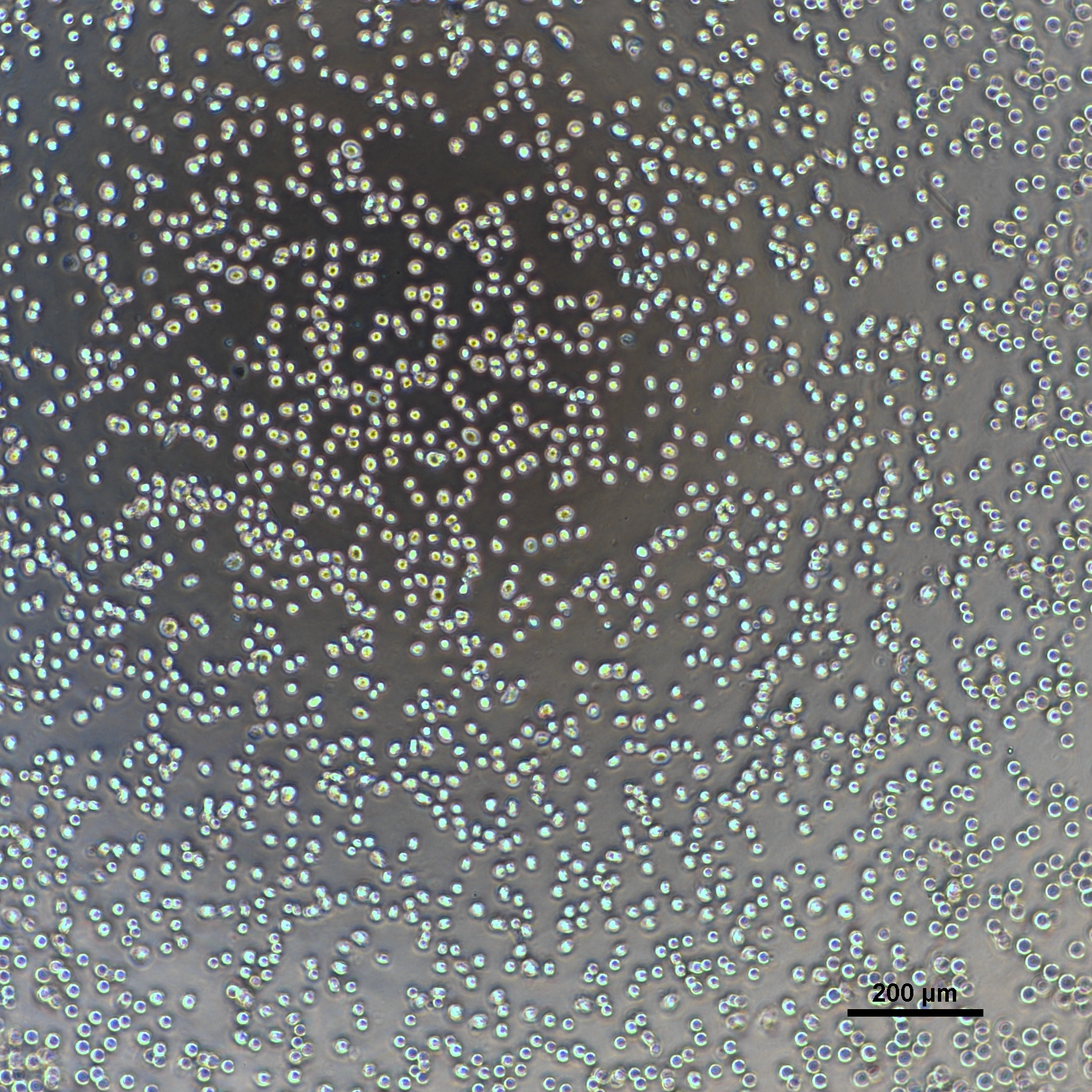

Supplement: Supplemental Information 8 [file peerj-10-13498-s008.zip › 0h/hpe1.tif]

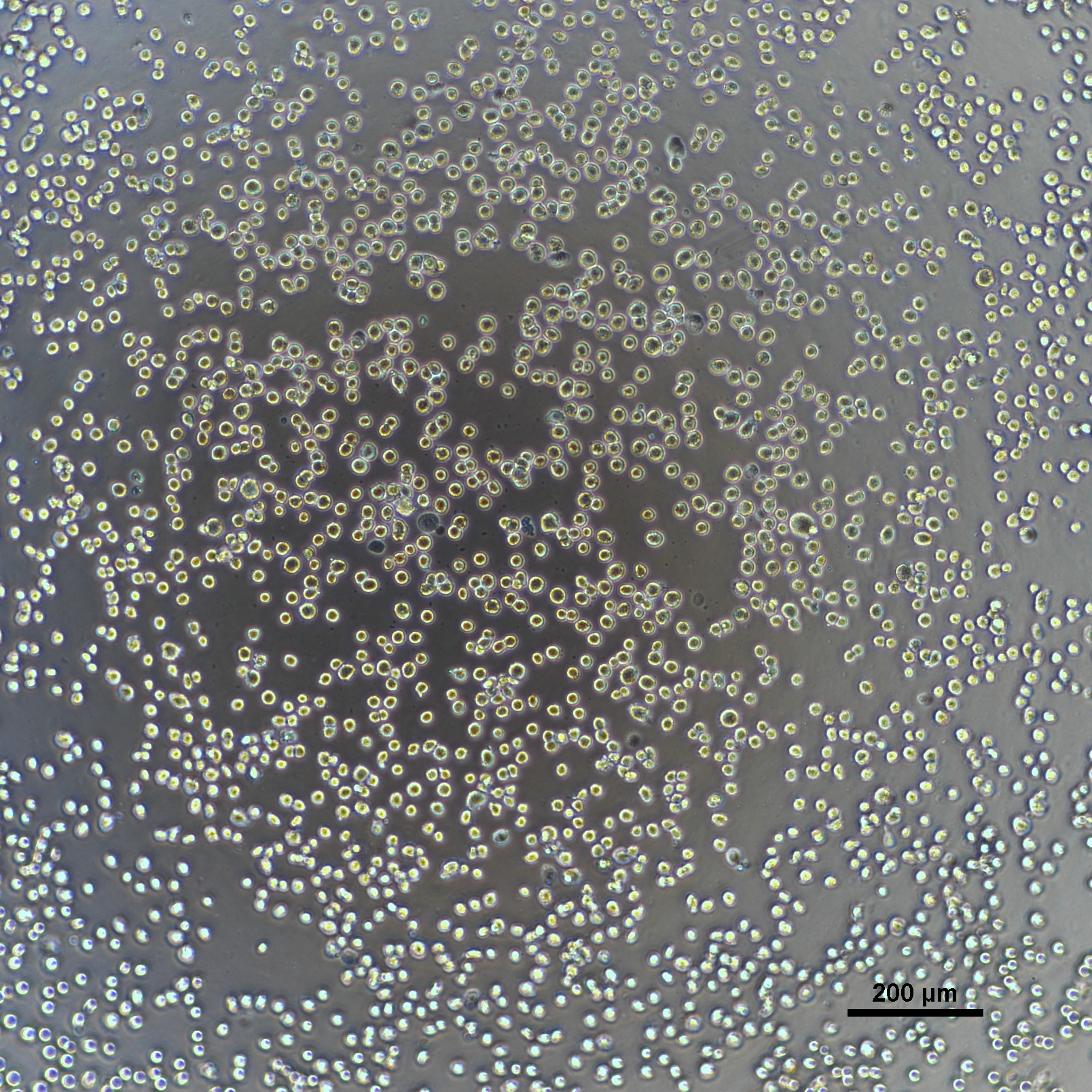

Supplement: Supplemental Information 8 [file peerj-10-13498-s008.zip › 0h/hue1.tif]

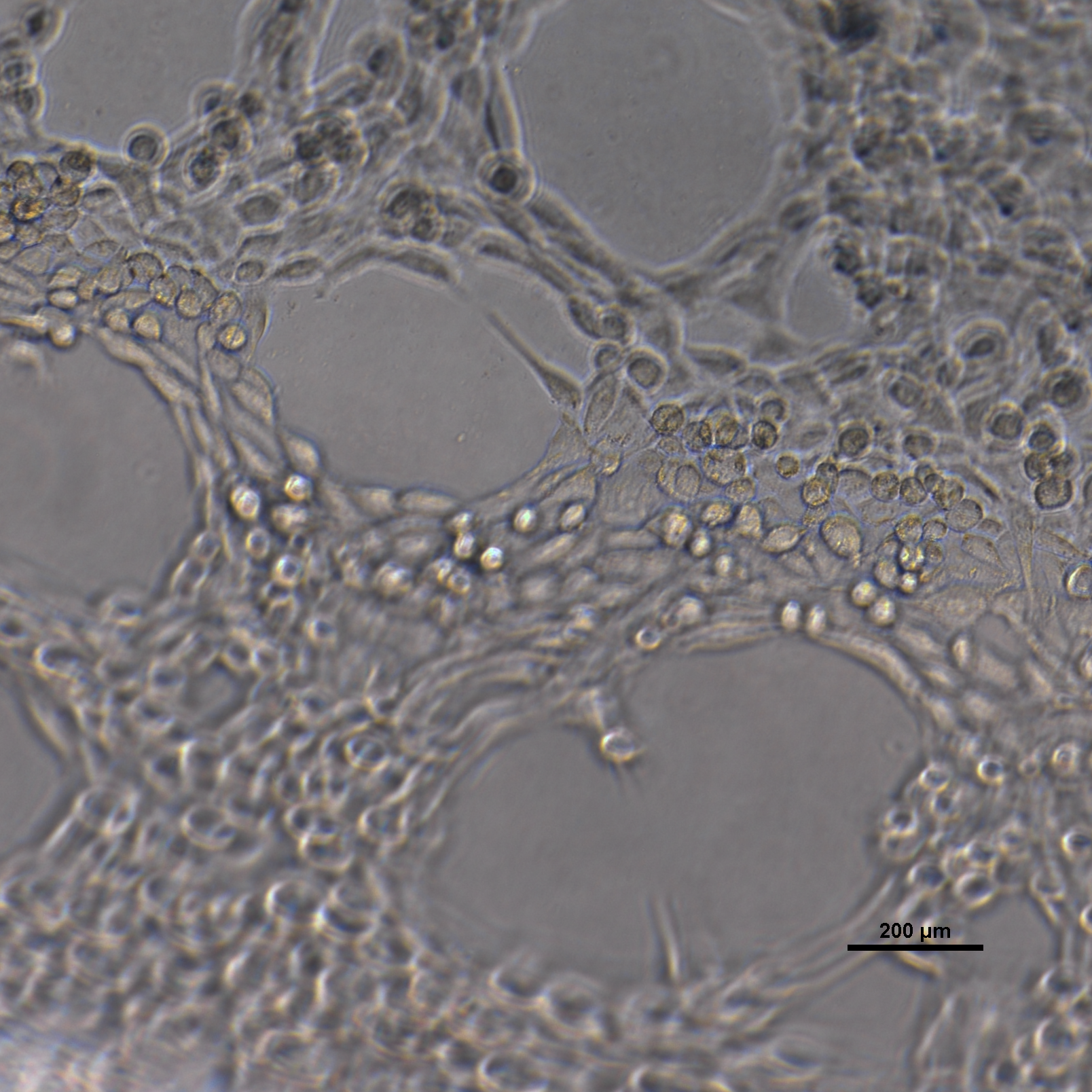

Supplement: Supplemental Information 12 [file peerj-10-13498-s012.tif]

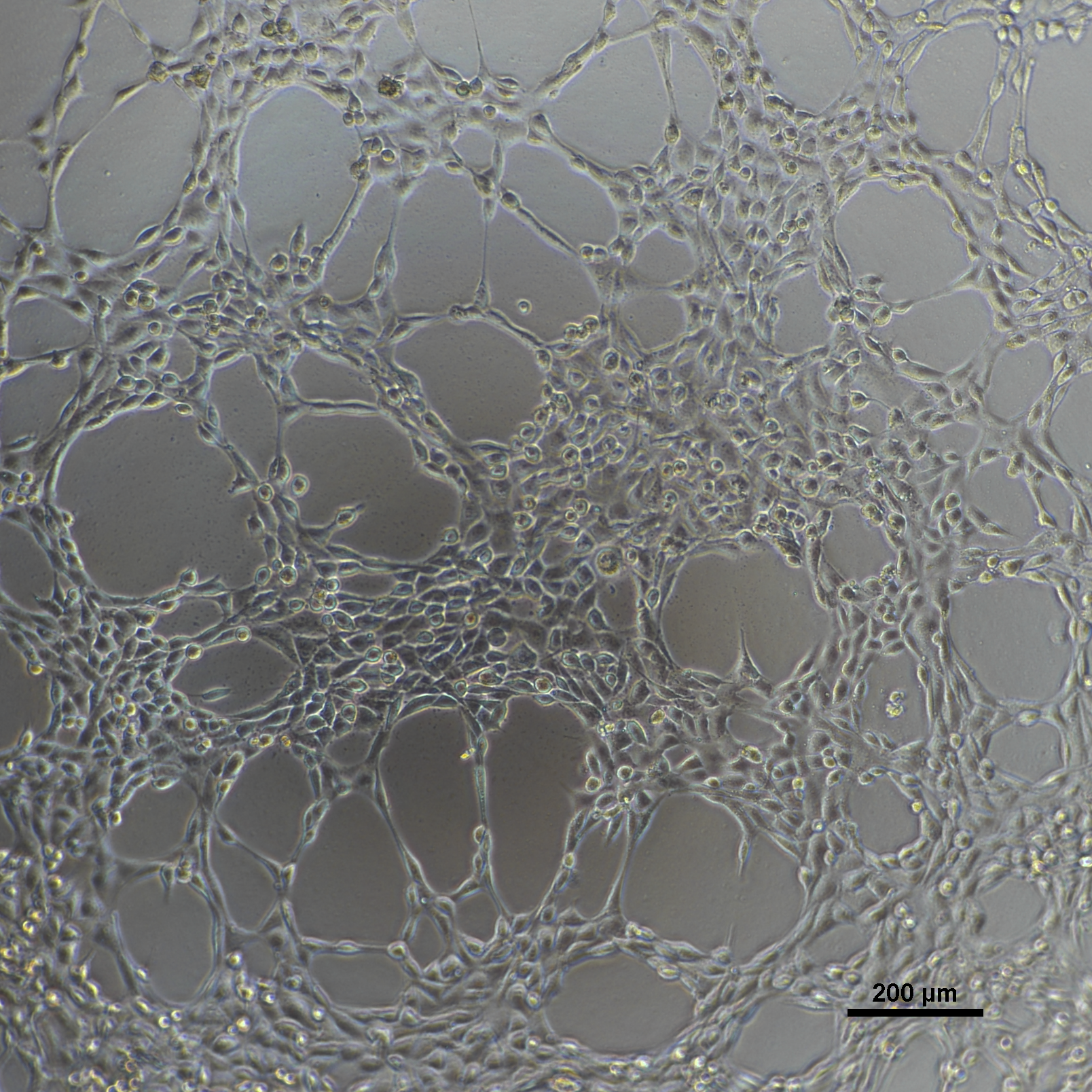

Supplement: Supplemental Information 13 [file peerj-10-13498-s013.tif]

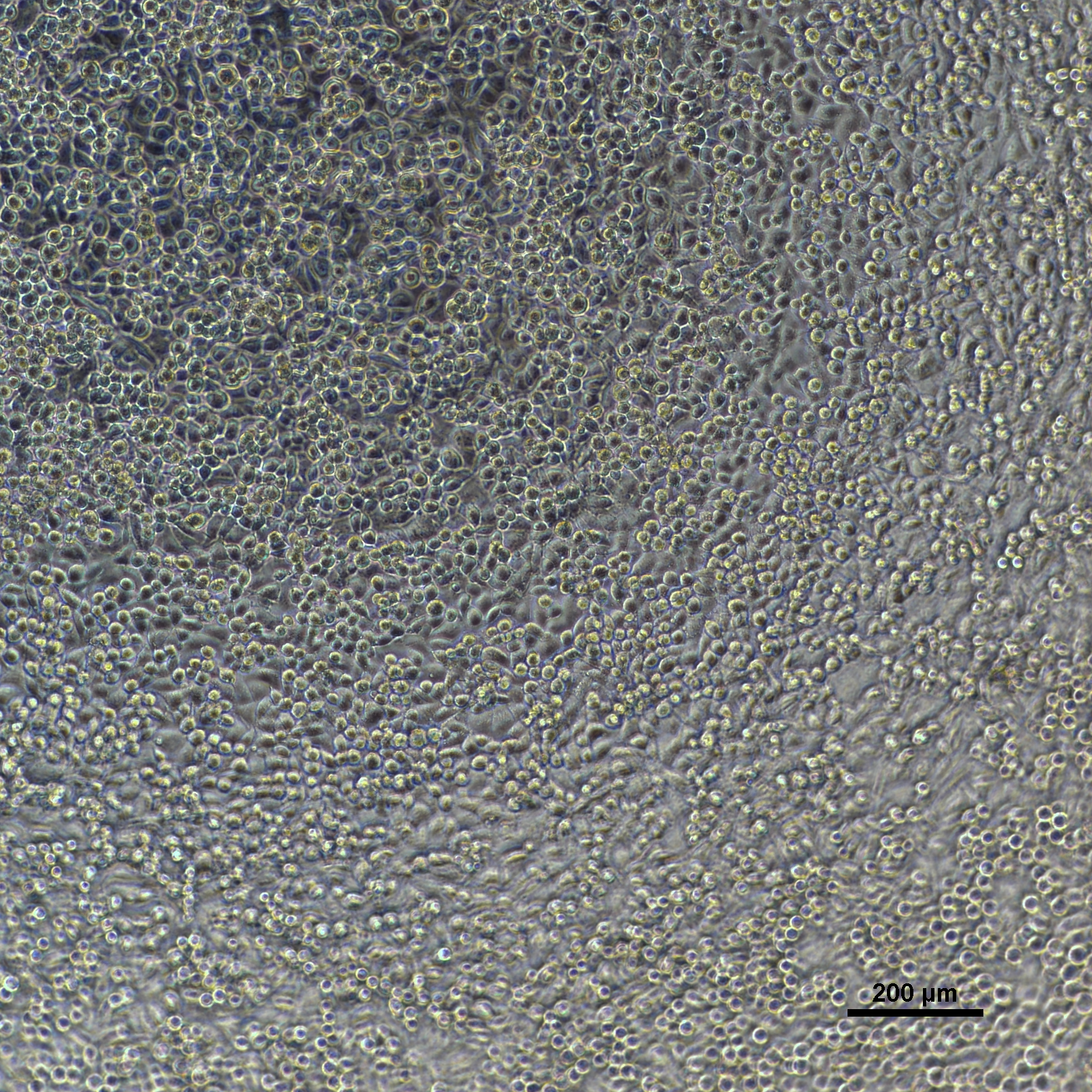

Supplement: Supplemental Information 14 [file peerj-10-13498-s014.tif]

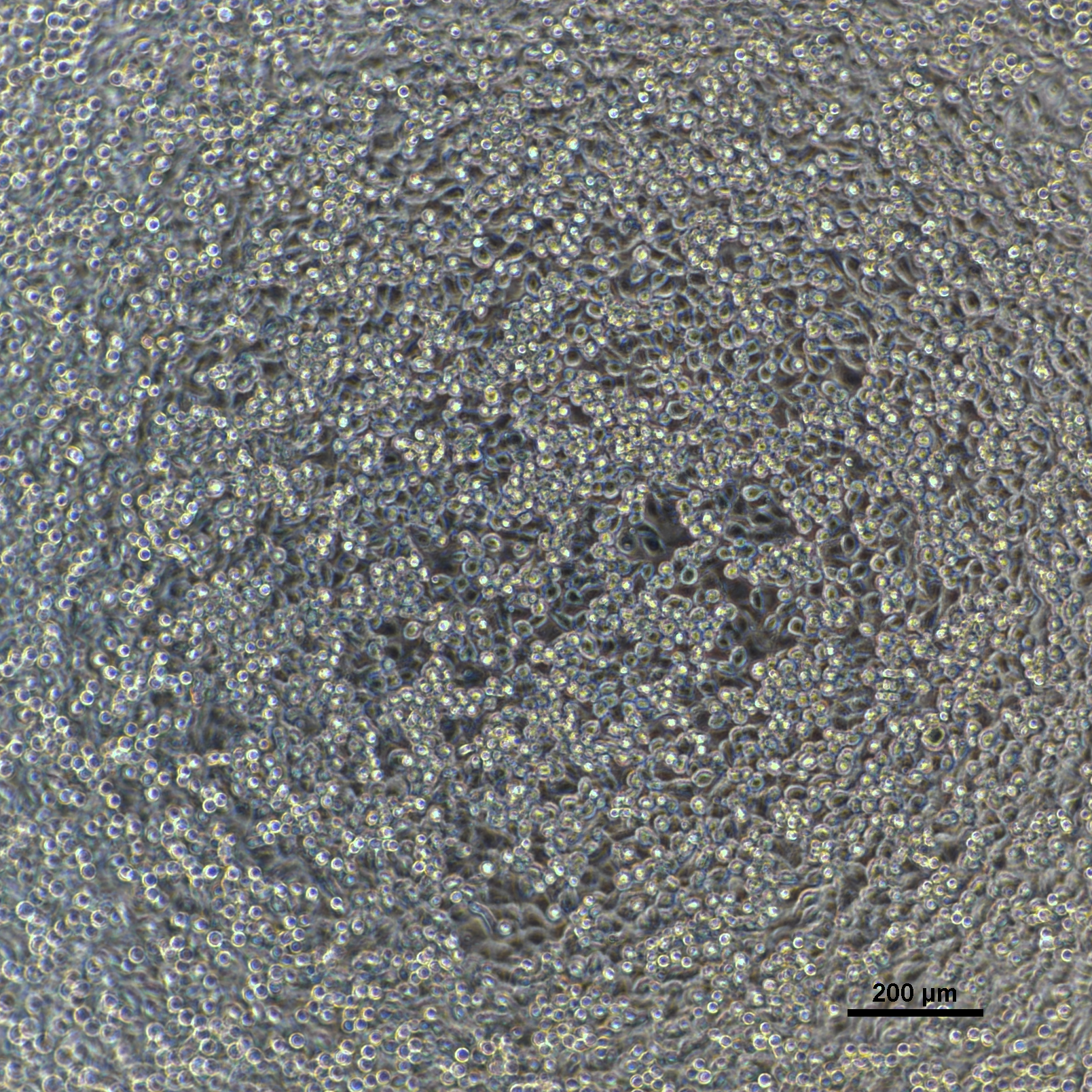

Supplement: Supplemental Information 16 [file peerj-10-13498-s016.tif]

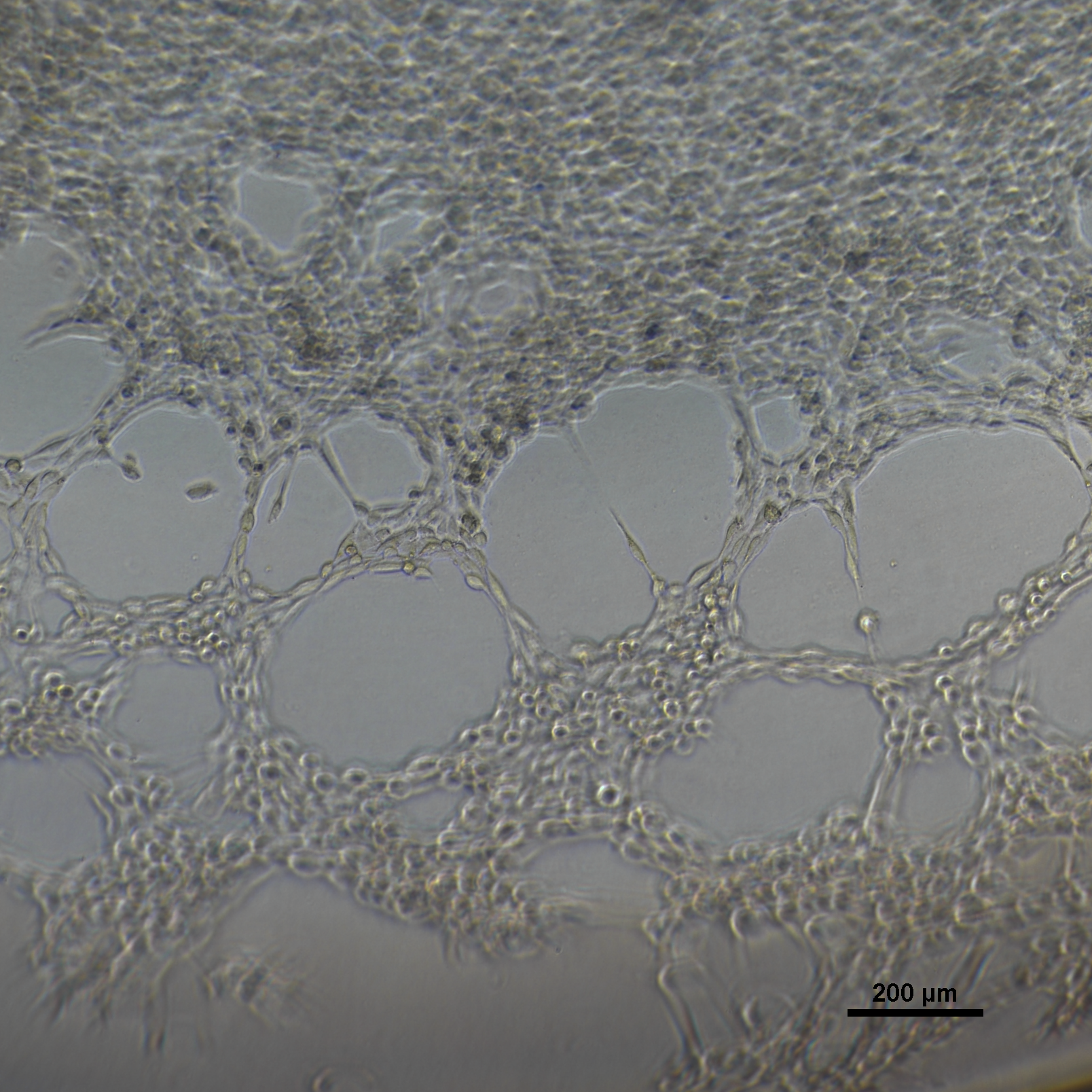

Supplement: Supplemental Information 17 [file peerj-10-13498-s017.tif]

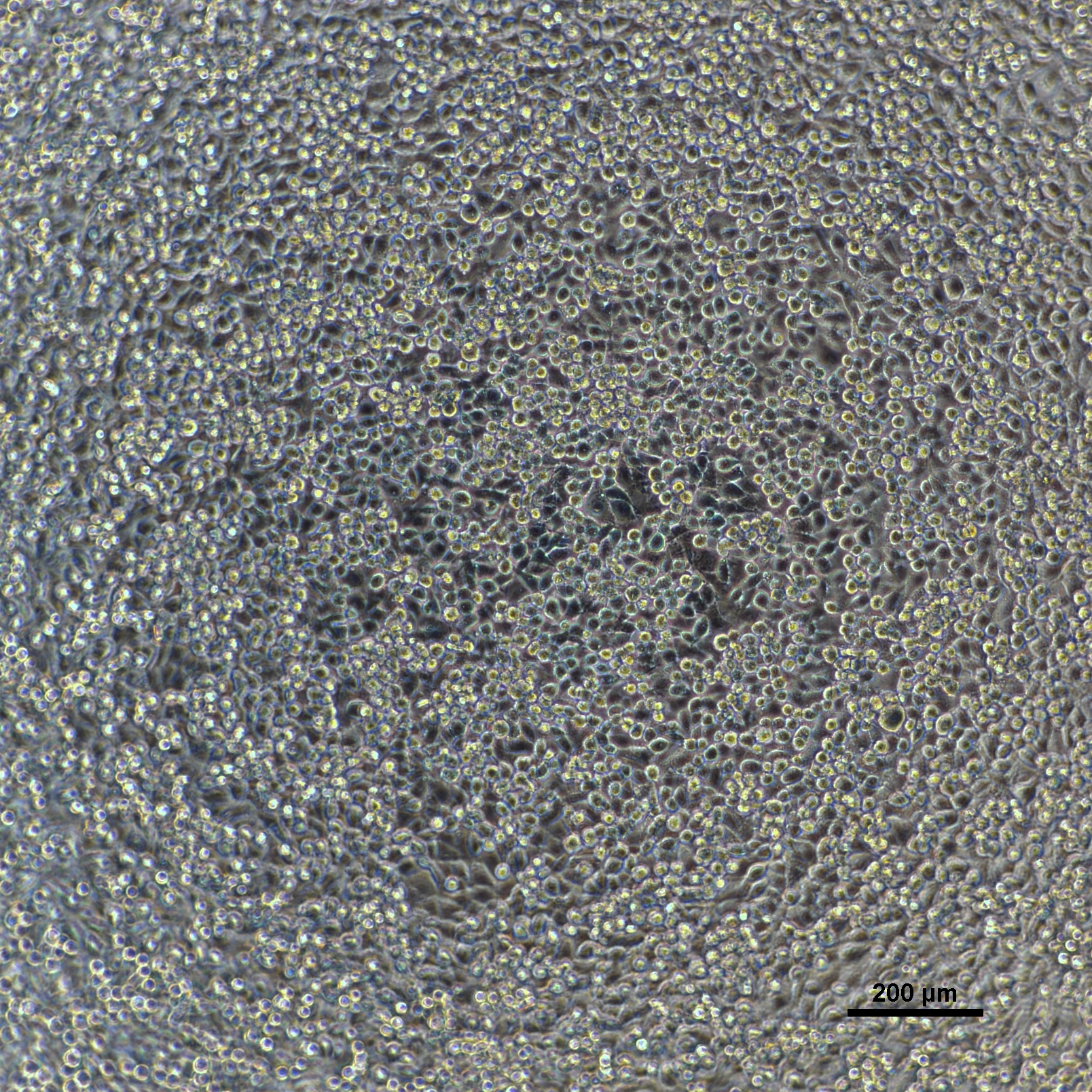

Supplement: Supplemental Information 18 [file peerj-10-13498-s018.tif]

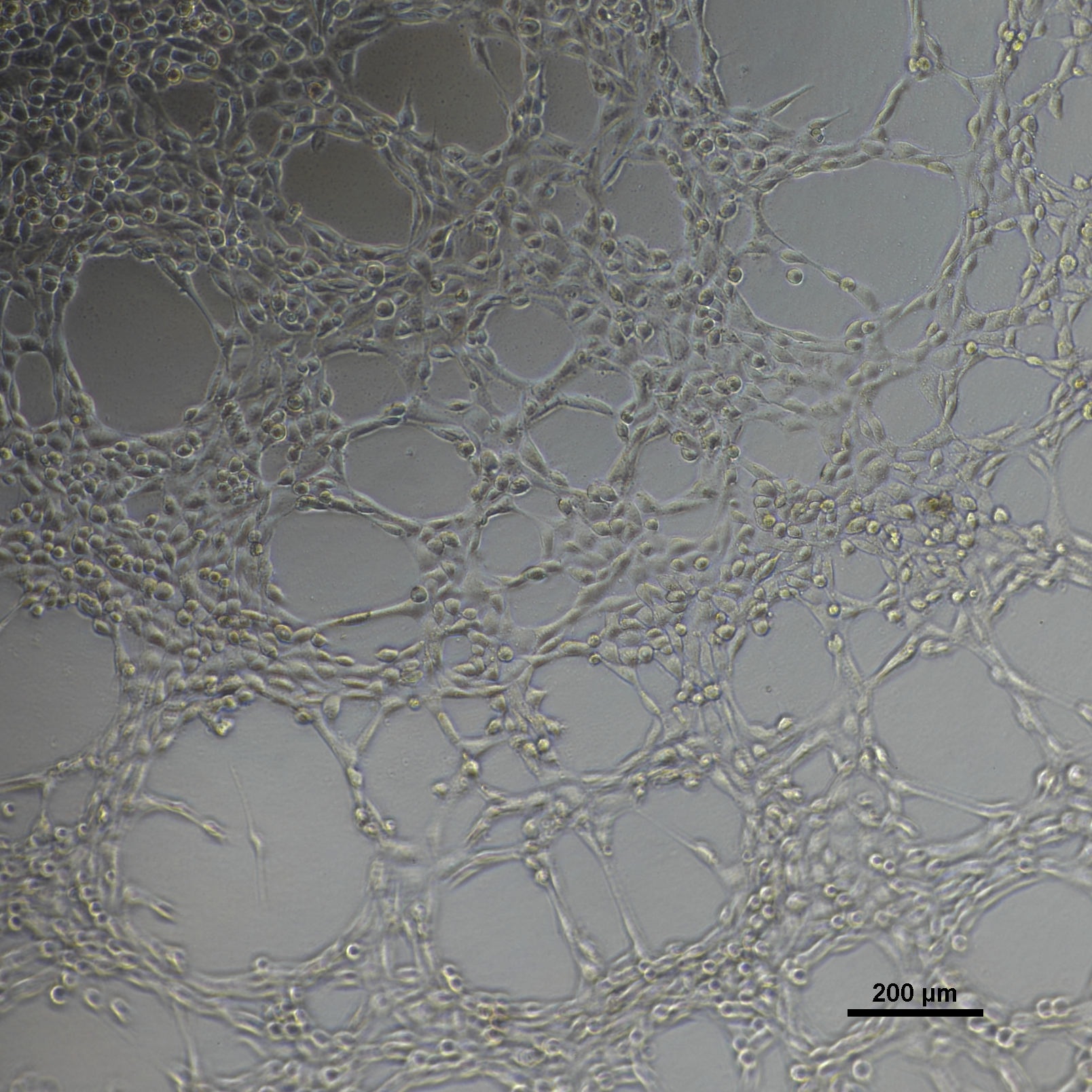

Supplement: Supplemental Information 21 [file peerj-10-13498-s021.tif]

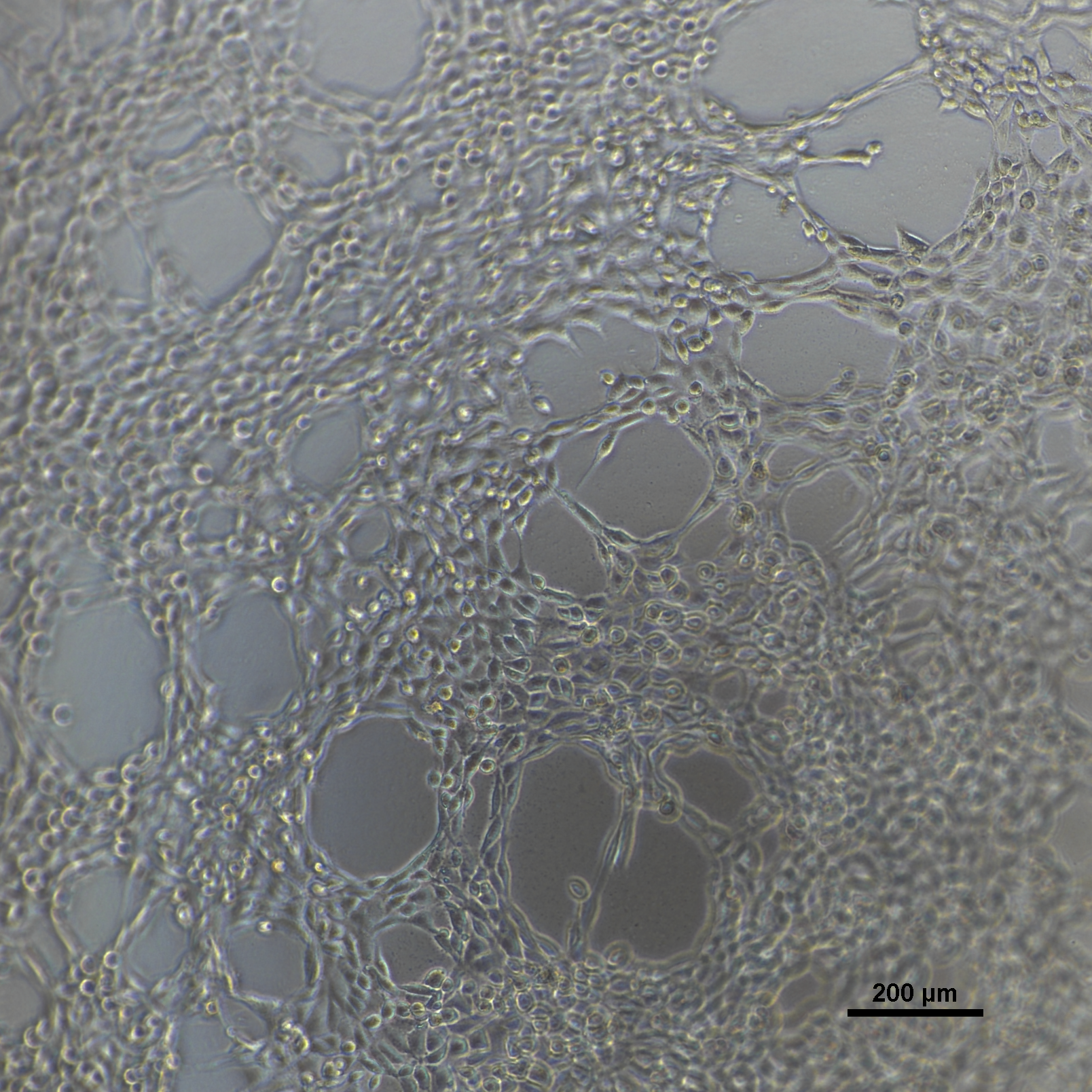

Supplement: Supplemental Information 22 [file peerj-10-13498-s022.tif]

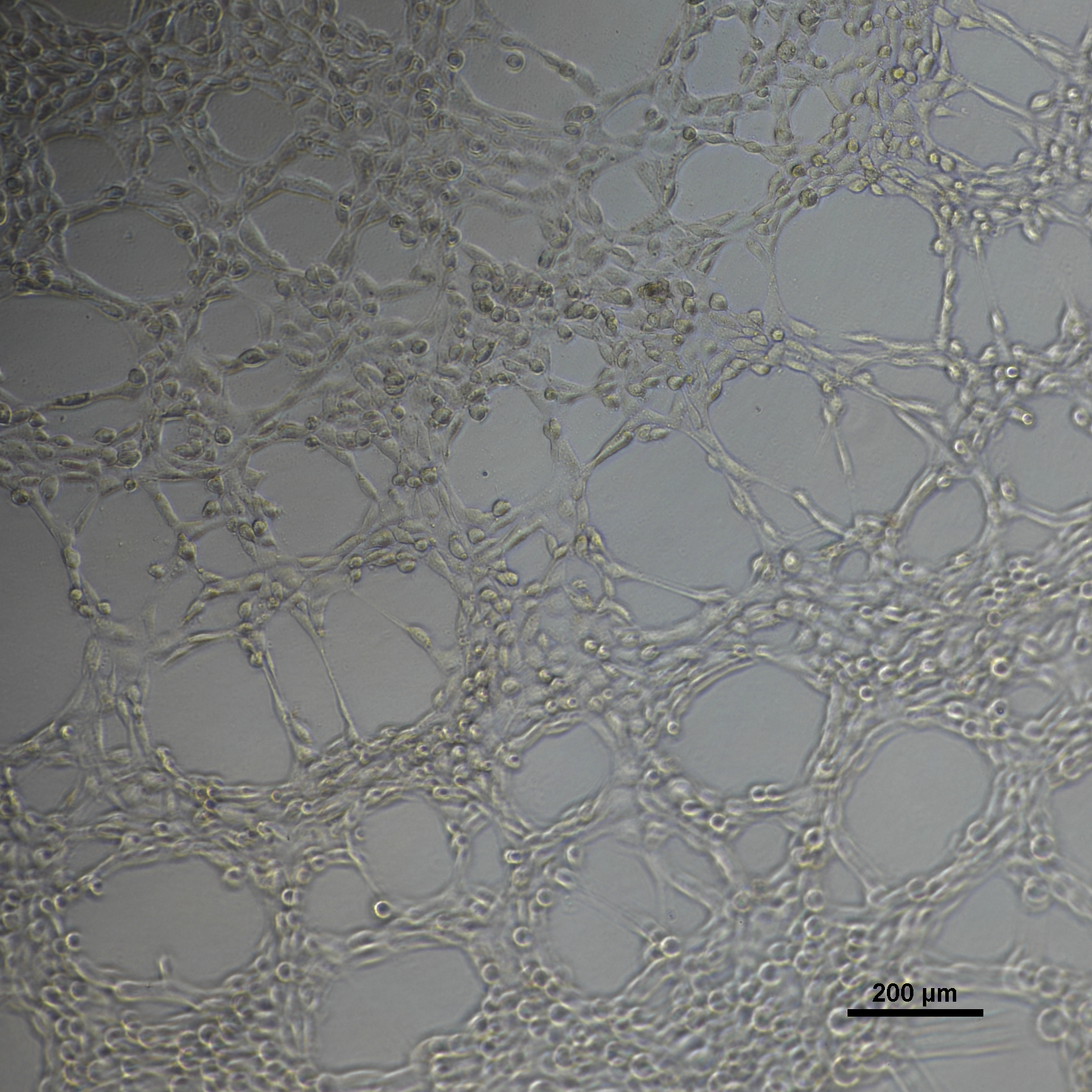

Supplement: Supplemental Information 24 [file peerj-10-13498-s024.tif]

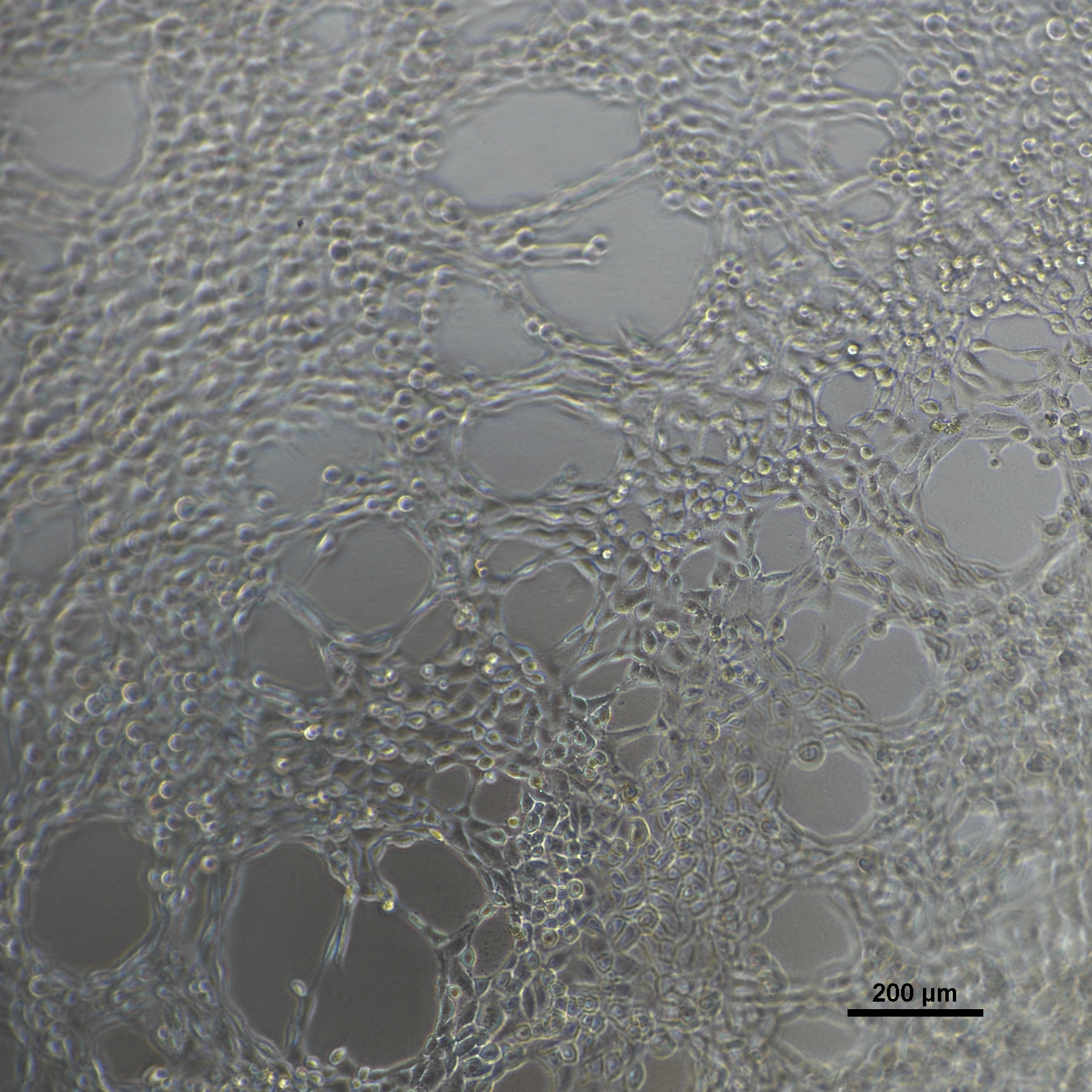

Supplement: Supplemental Information 26 [file peerj-10-13498-s026.tif]

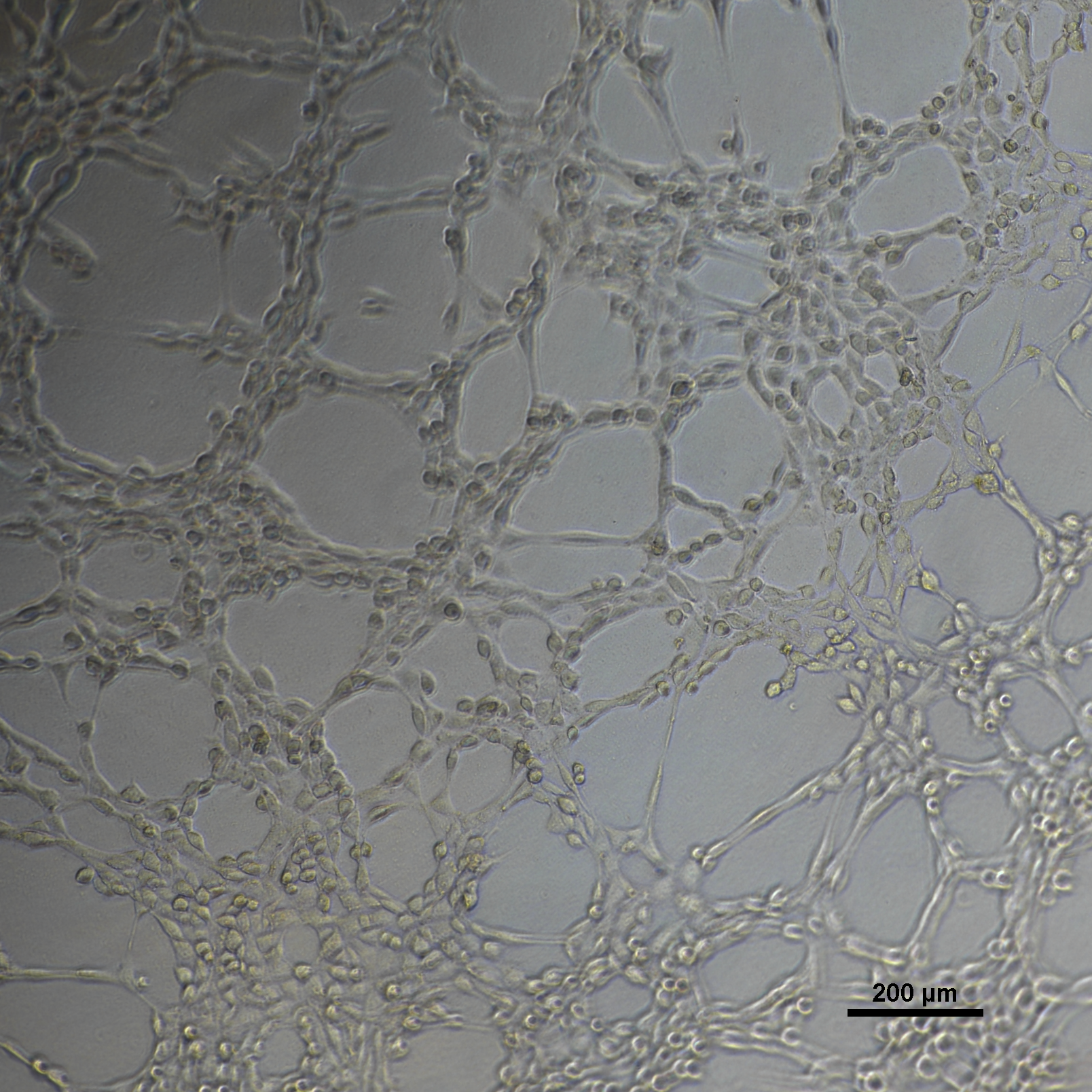

Supplement: Supplemental Information 27 [file peerj-10-13498-s027.tif]

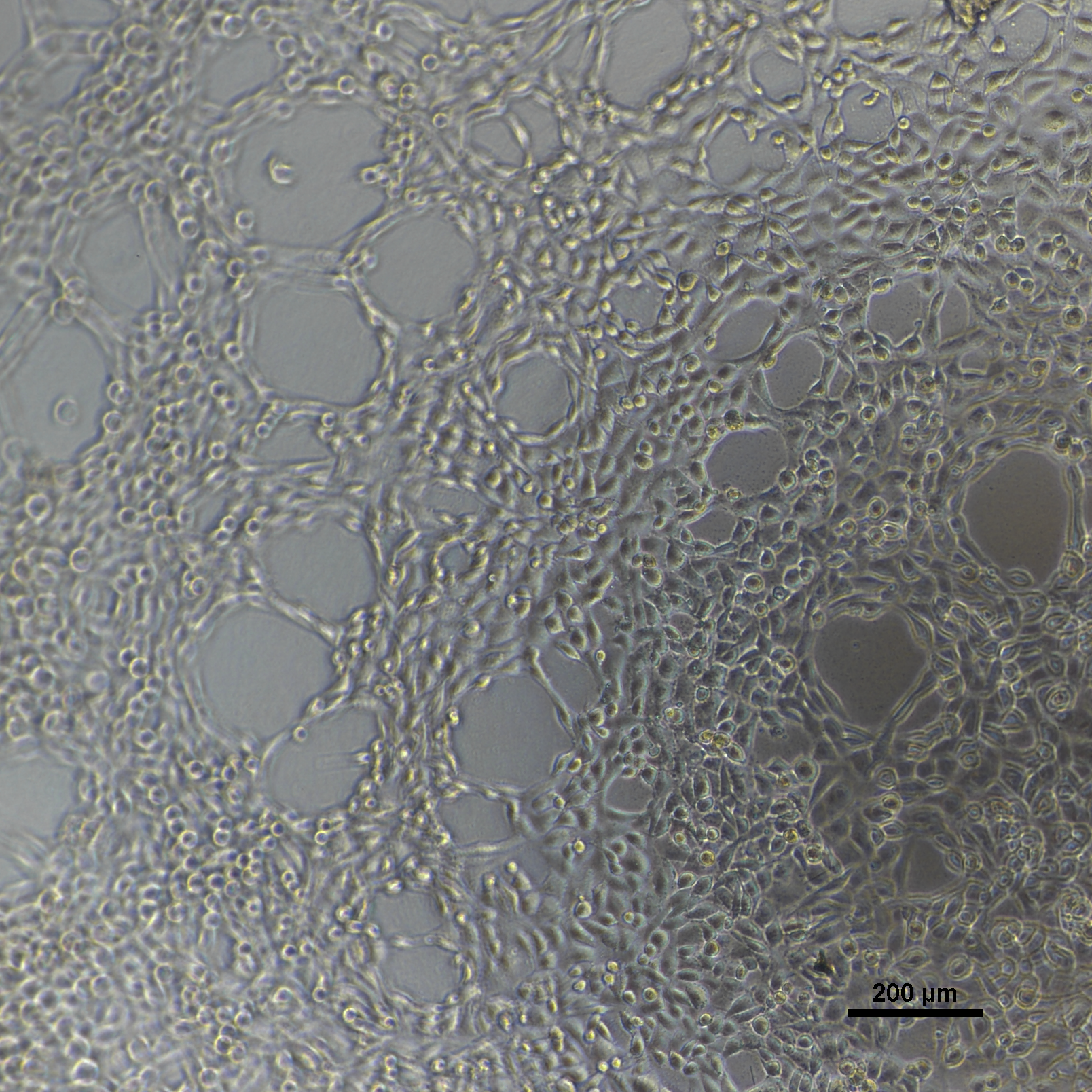

Supplement: Supplemental Information 28 [file peerj-10-13498-s028.tif]

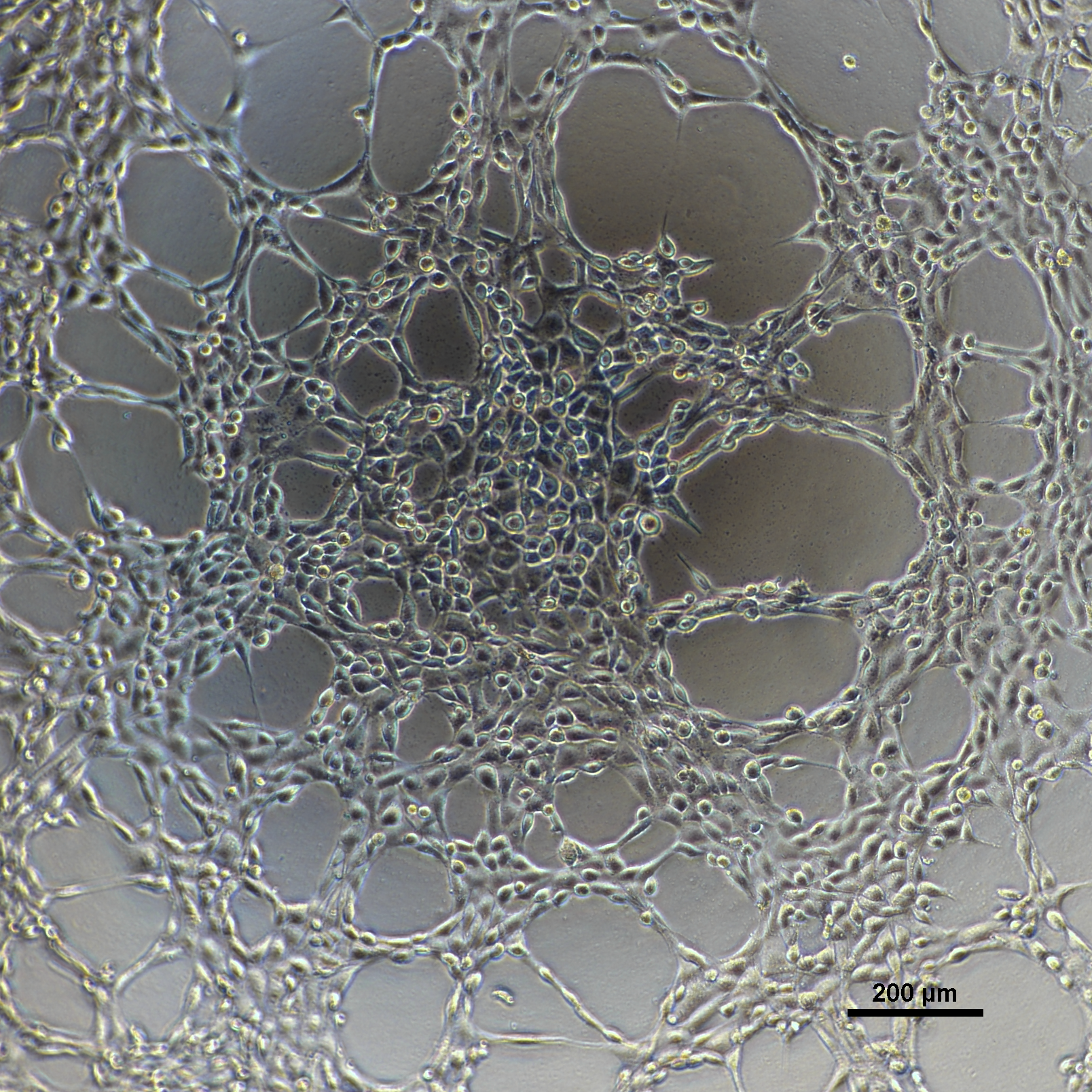

Supplement: Supplemental Information 29 [file peerj-10-13498-s029.tif]

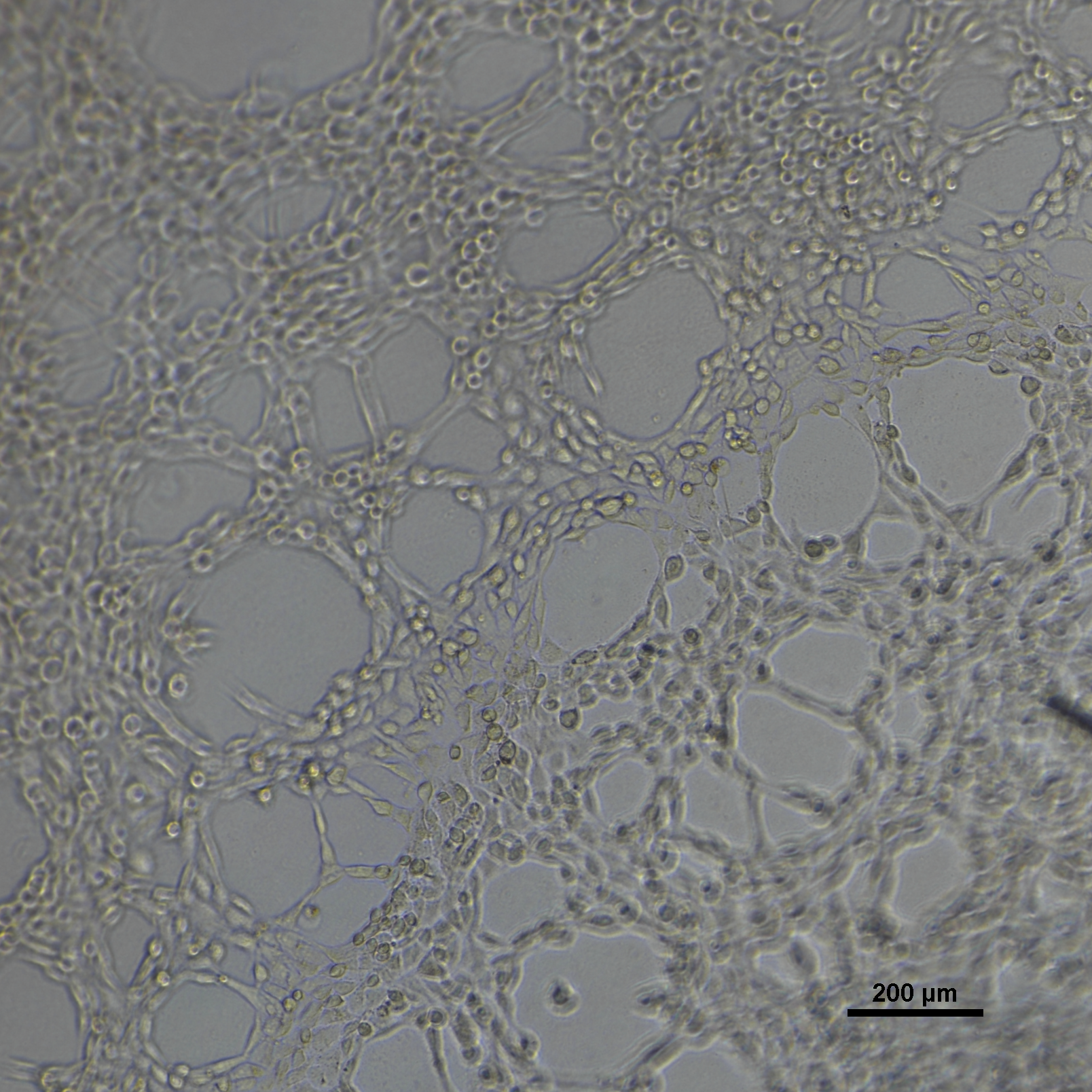

Supplement: Supplemental Information 30 [file peerj-10-13498-s030.tif]

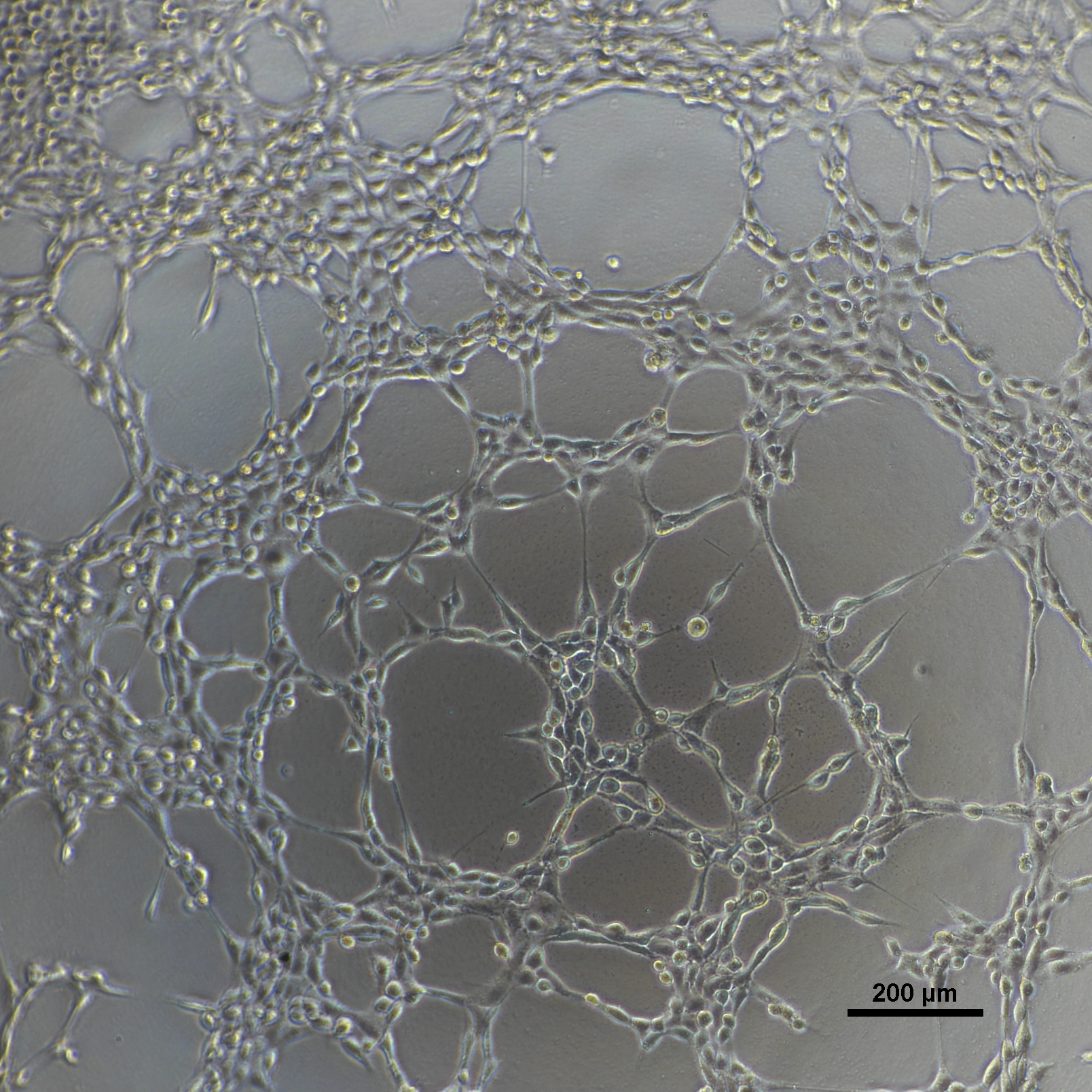

Supplement: Supplemental Information 31 [file peerj-10-13498-s031.tif]

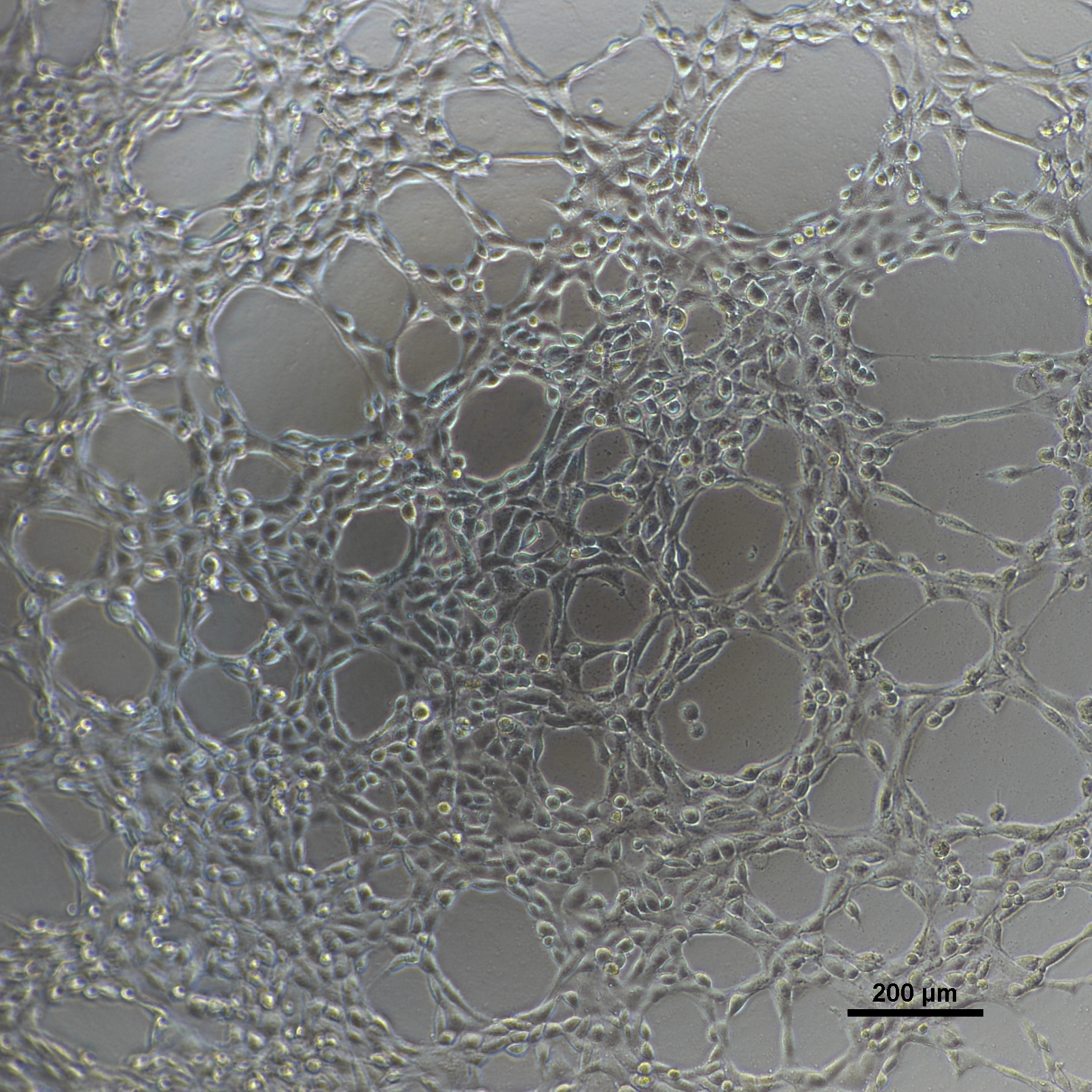

Supplement: Supplemental Information 32 [file peerj-10-13498-s032.tif]

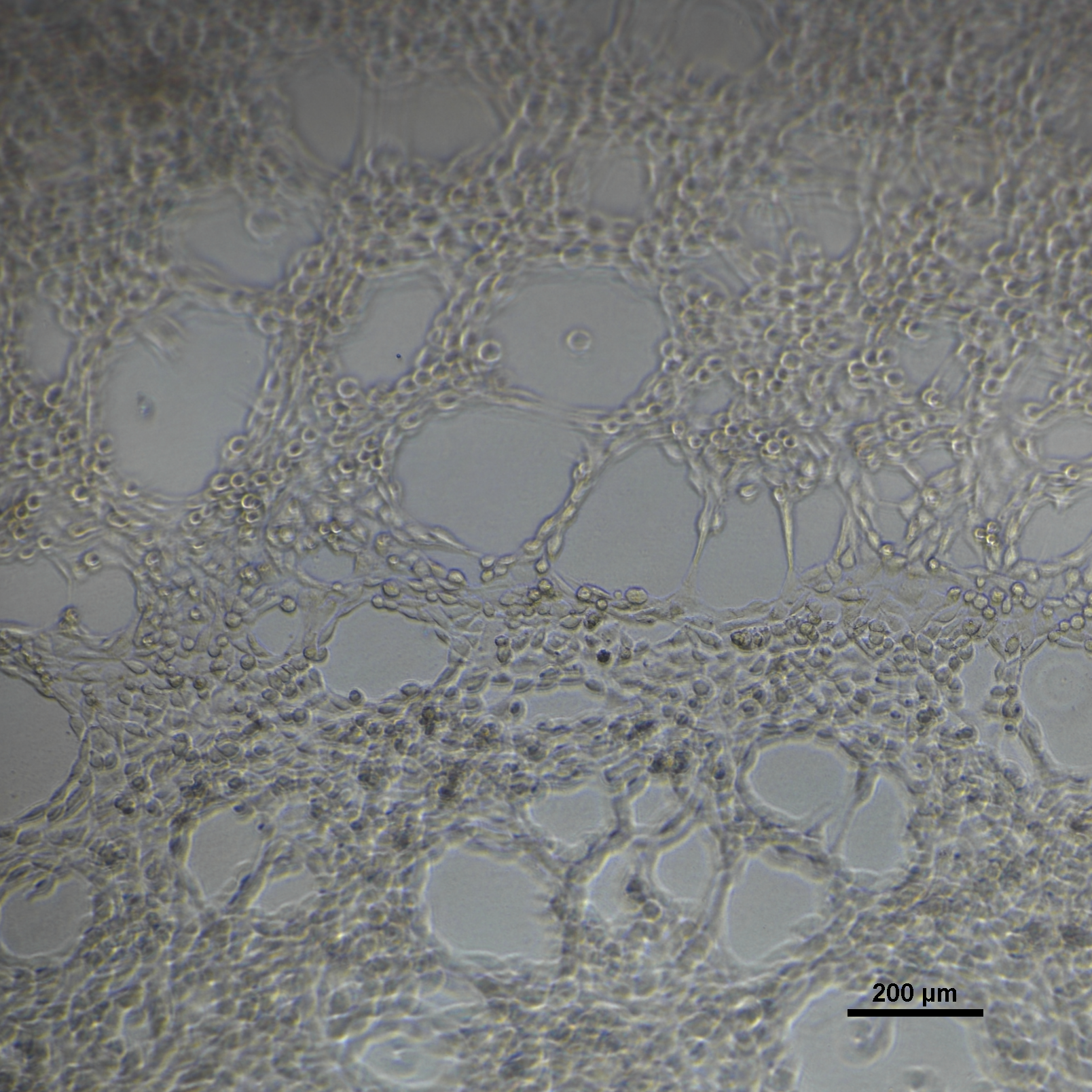

Supplement: Supplemental Information 36 [file peerj-10-13498-s036.tif]

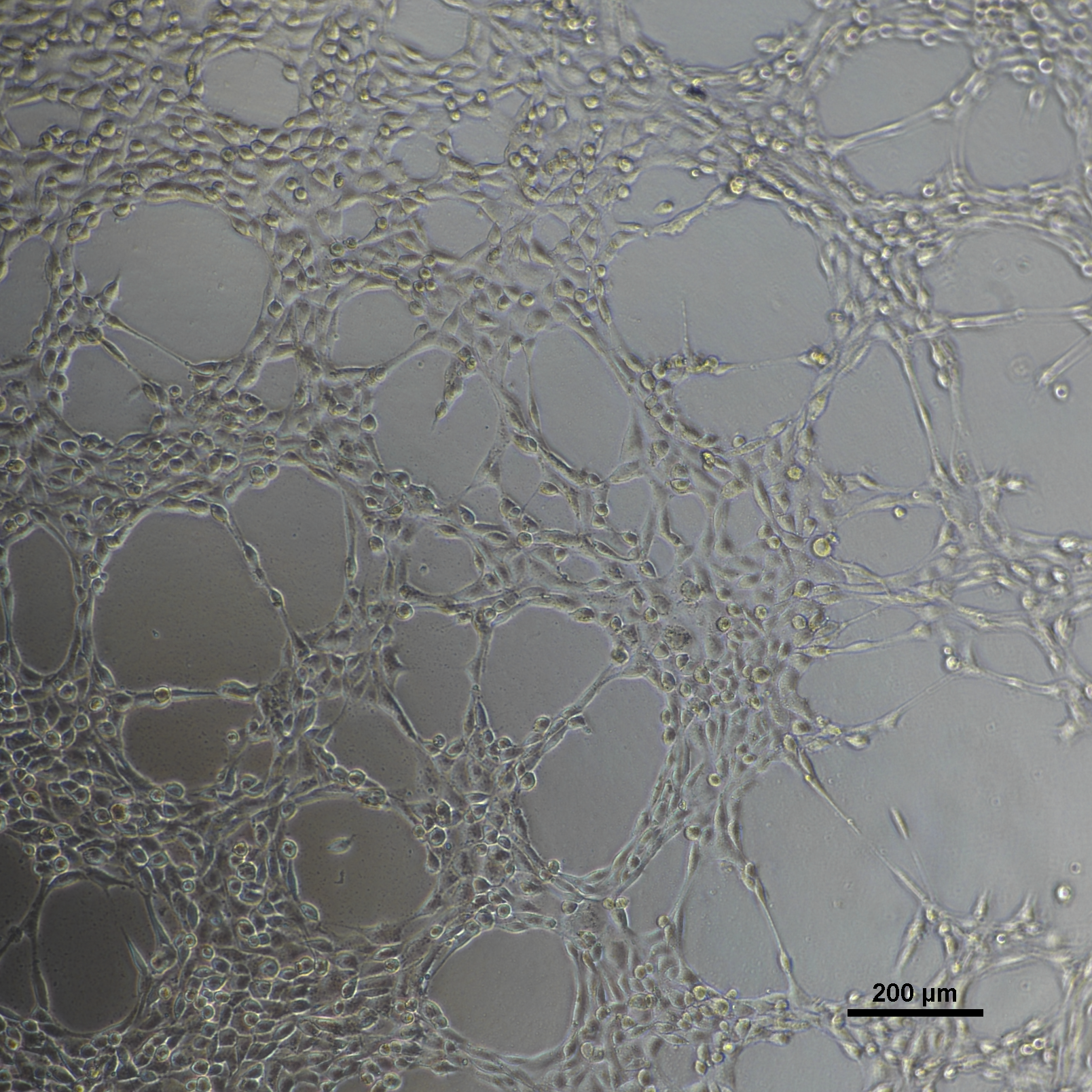

Supplement: Supplemental Information 37 [file peerj-10-13498-s037.tif]

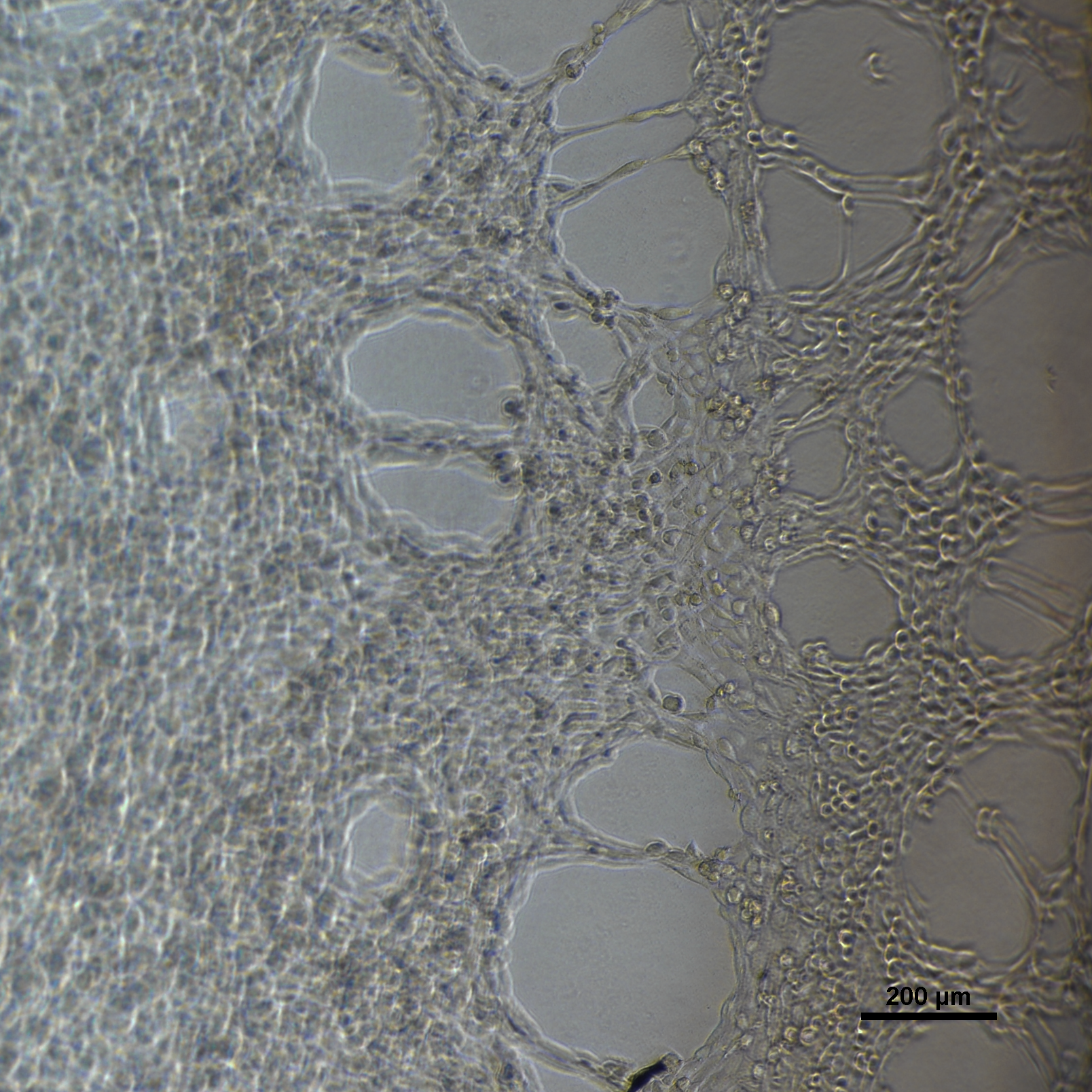

Supplement: Supplemental Information 38 [file peerj-10-13498-s038.tif]

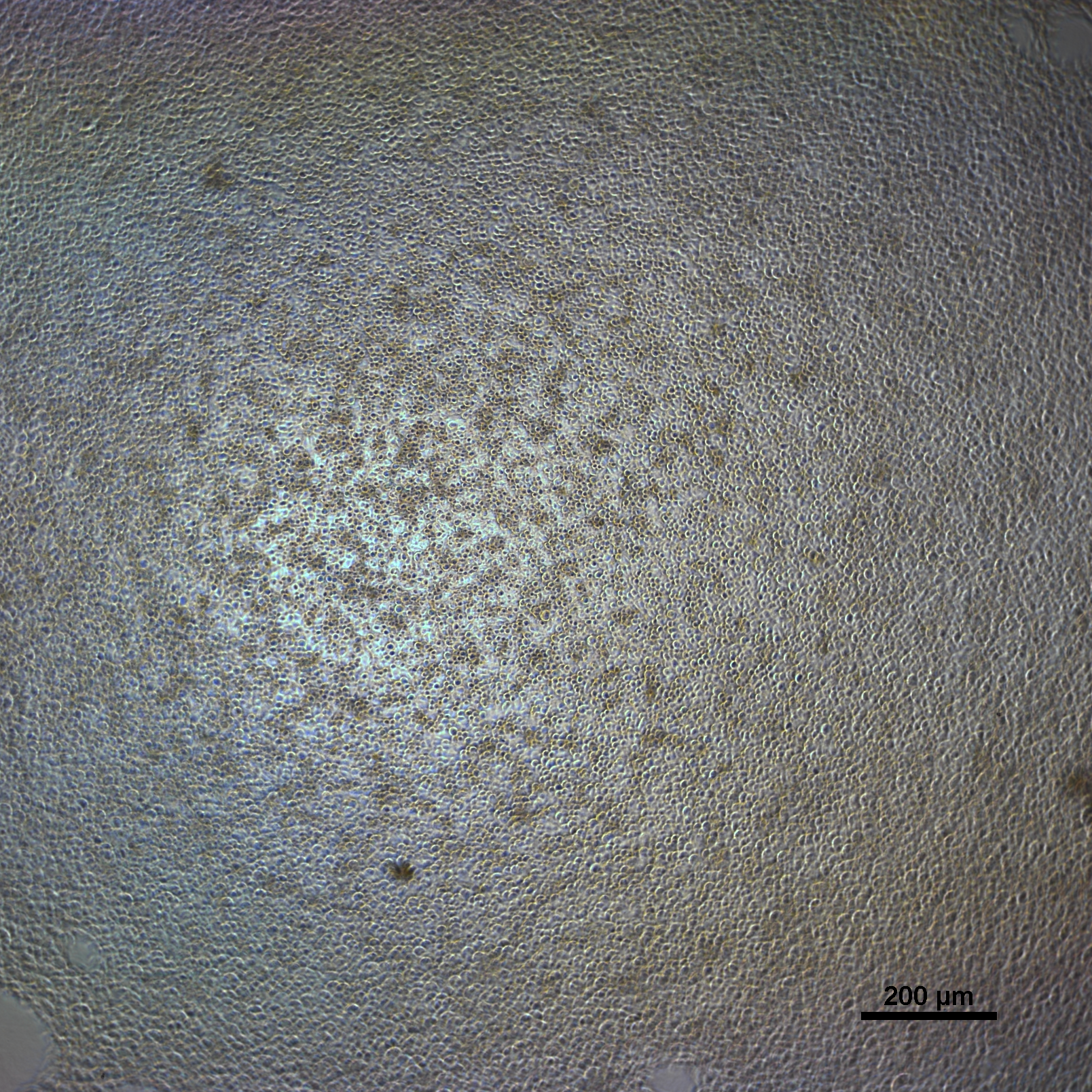

Supplement: Supplemental Information 39 [file peerj-10-13498-s039.tif]

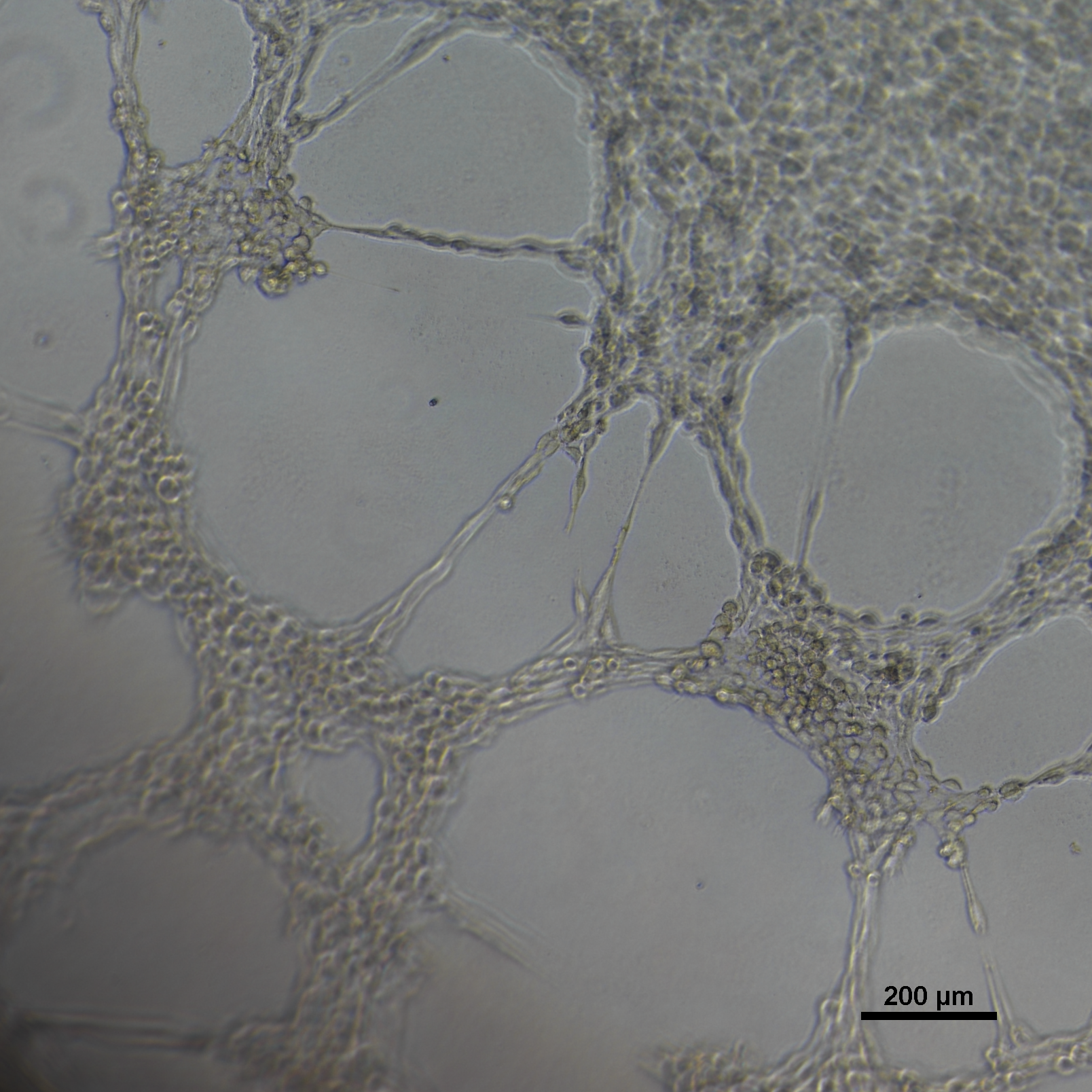

Supplement: Supplemental Information 40 [file peerj-10-13498-s040.tif]

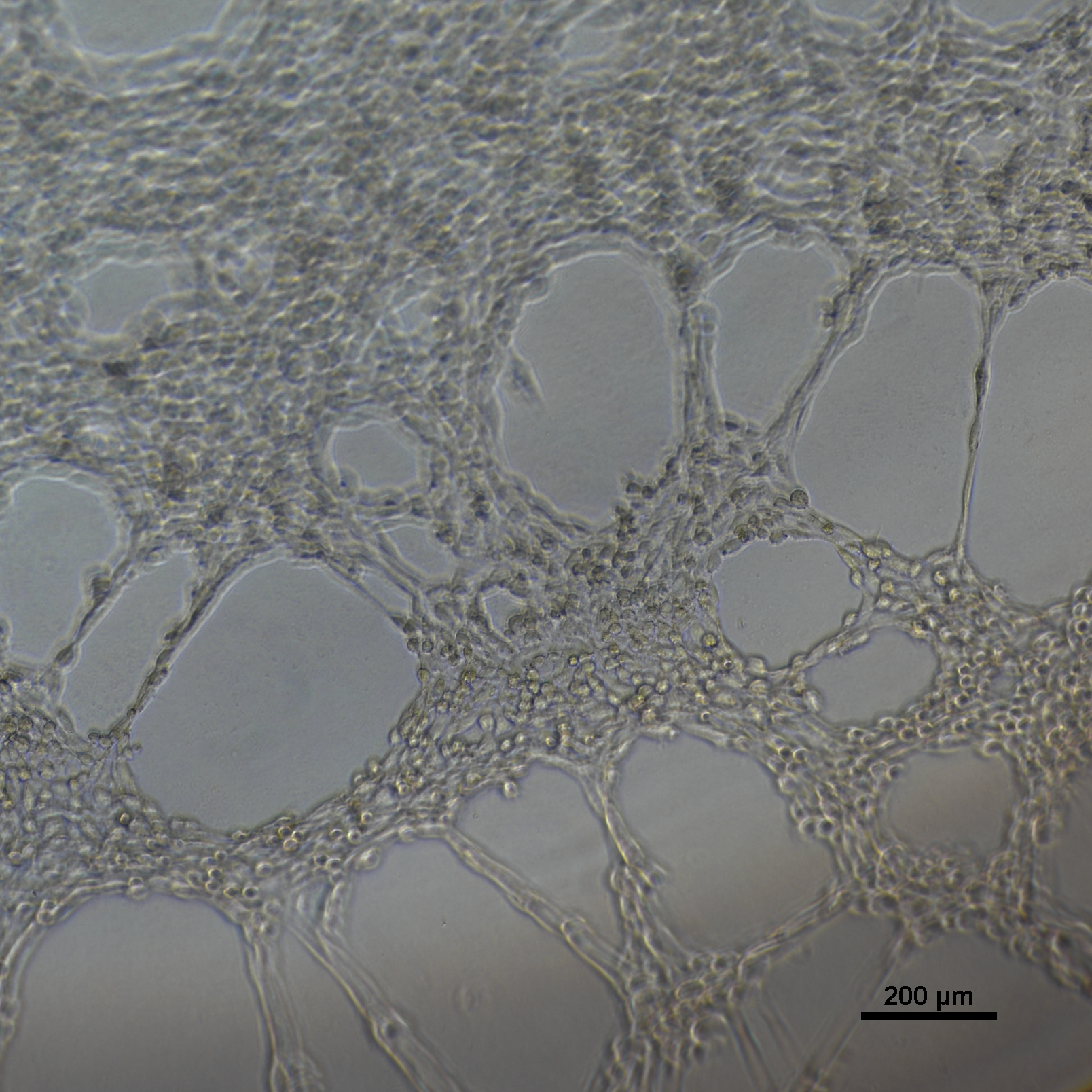

Supplement: Supplemental Information 43 [file peerj-10-13498-s043.tif]

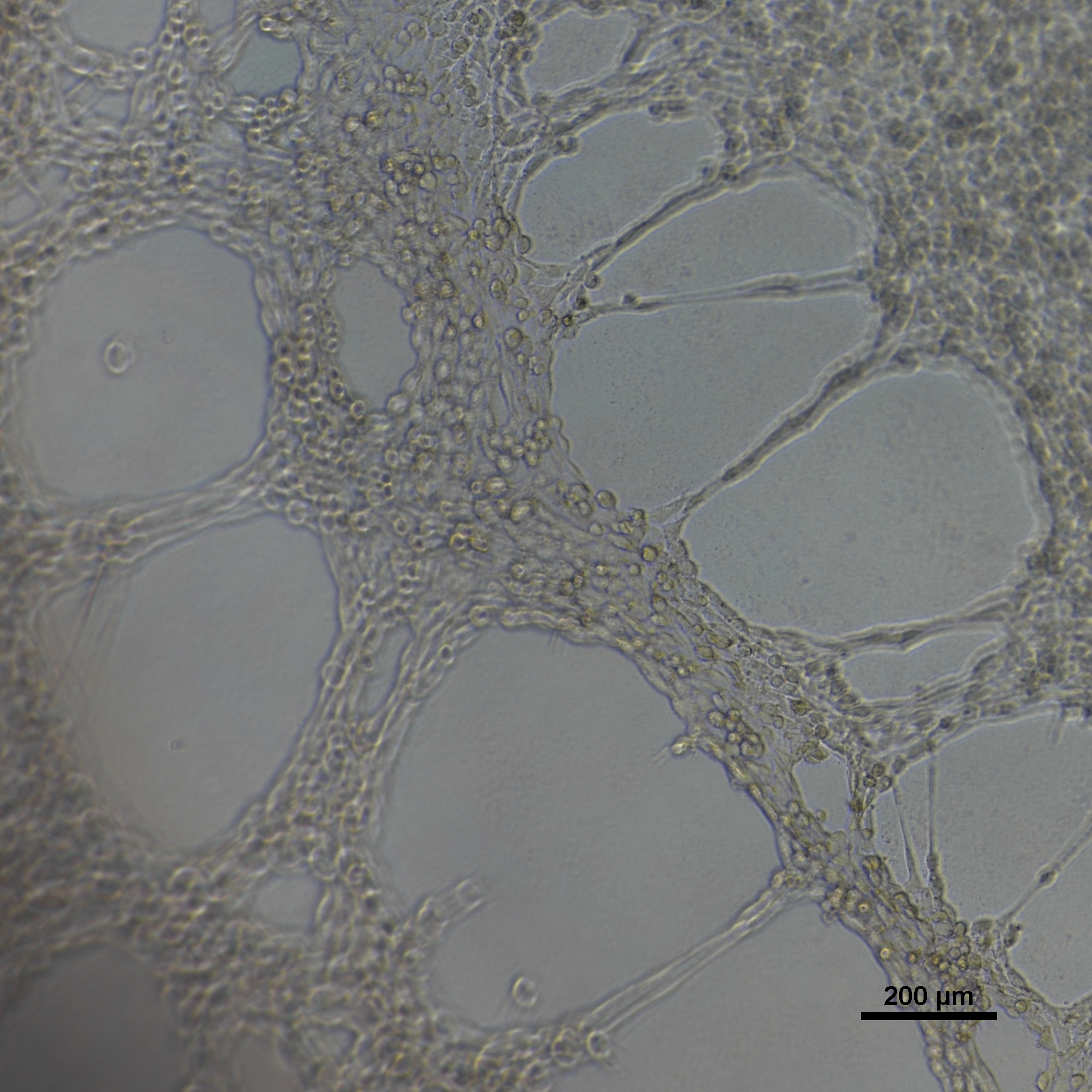

Supplement: Supplemental Information 44 [file peerj-10-13498-s044.tif]

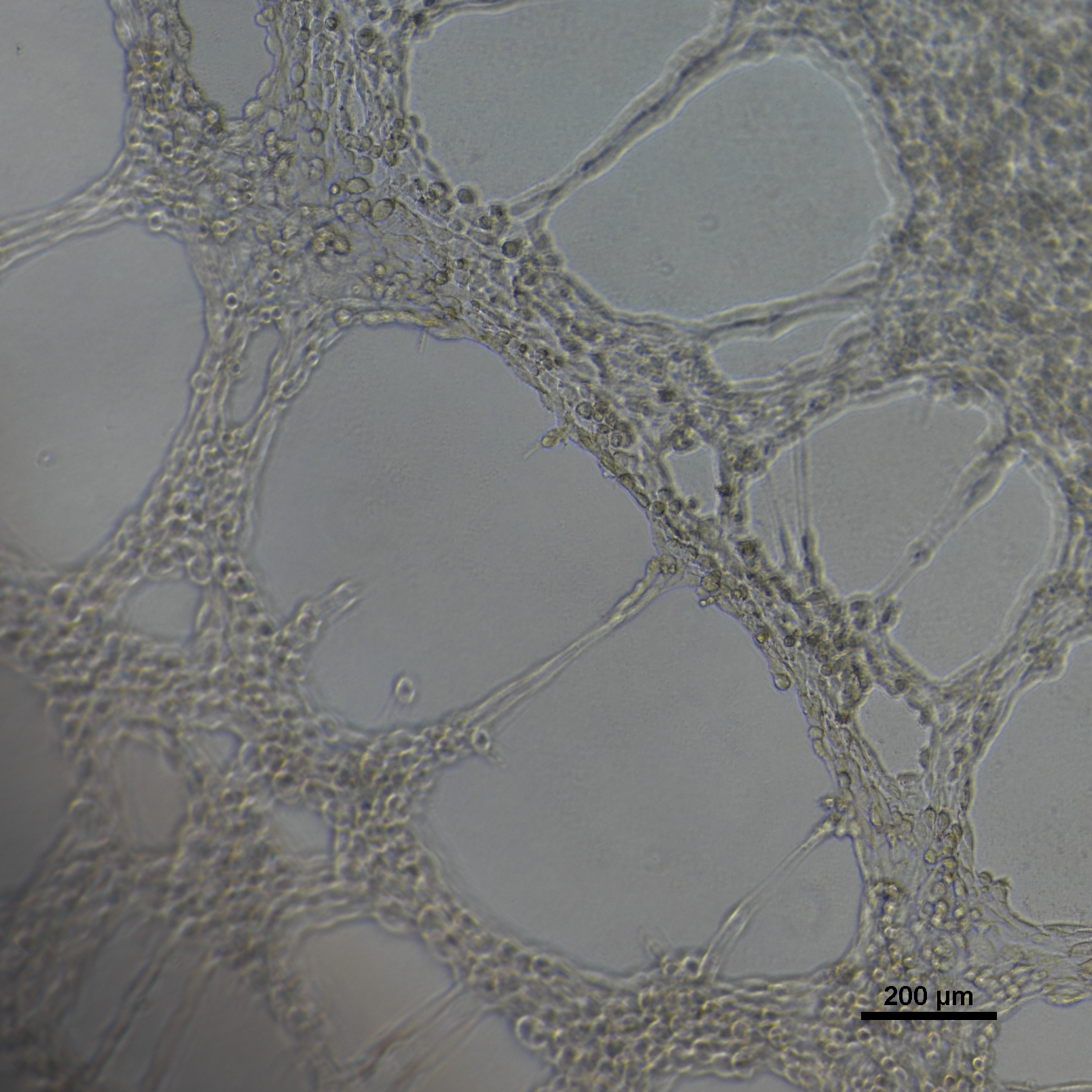

Supplement: Supplemental Information 45 [file peerj-10-13498-s045.tif]

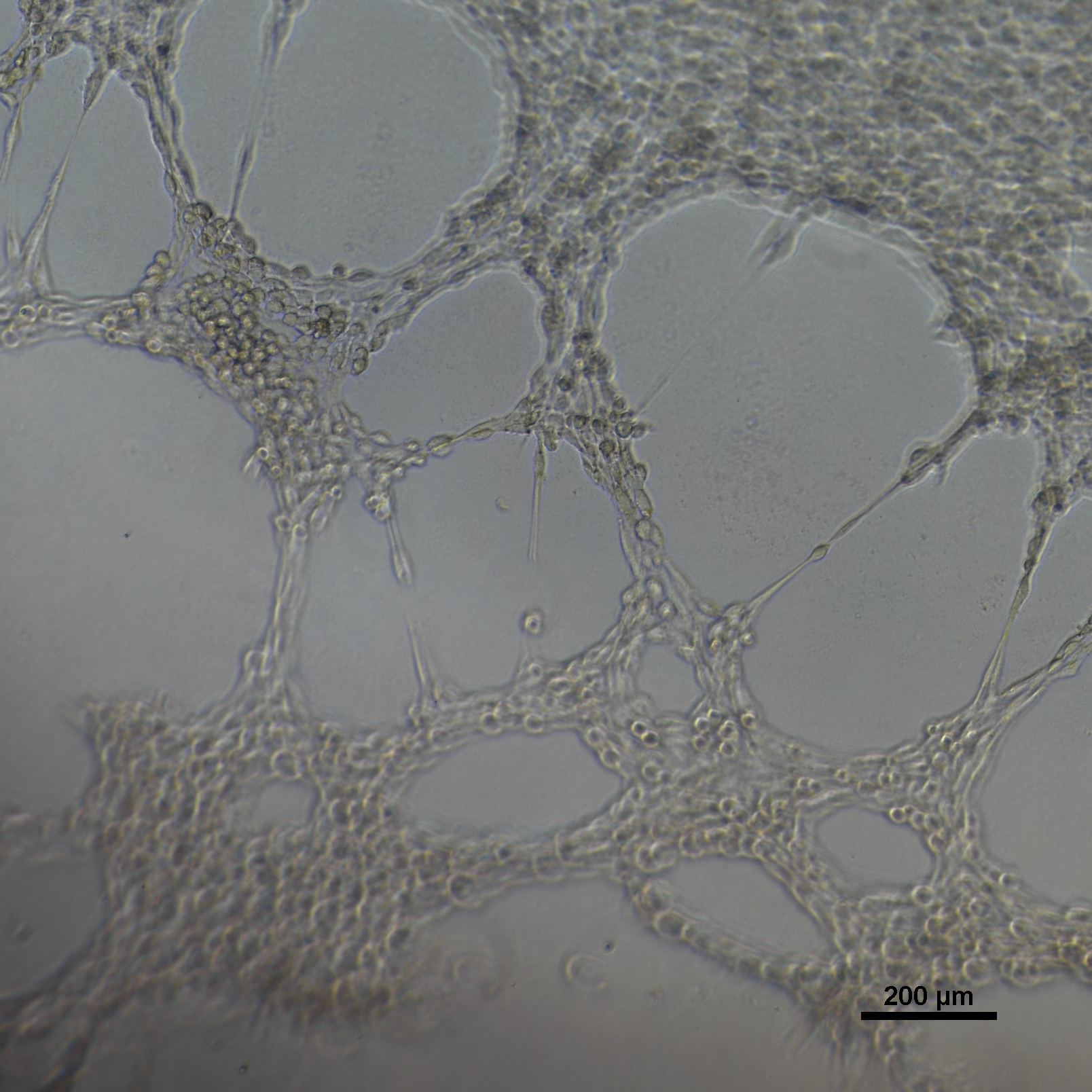

Supplement: Supplemental Information 46 [file peerj-10-13498-s046.tif]

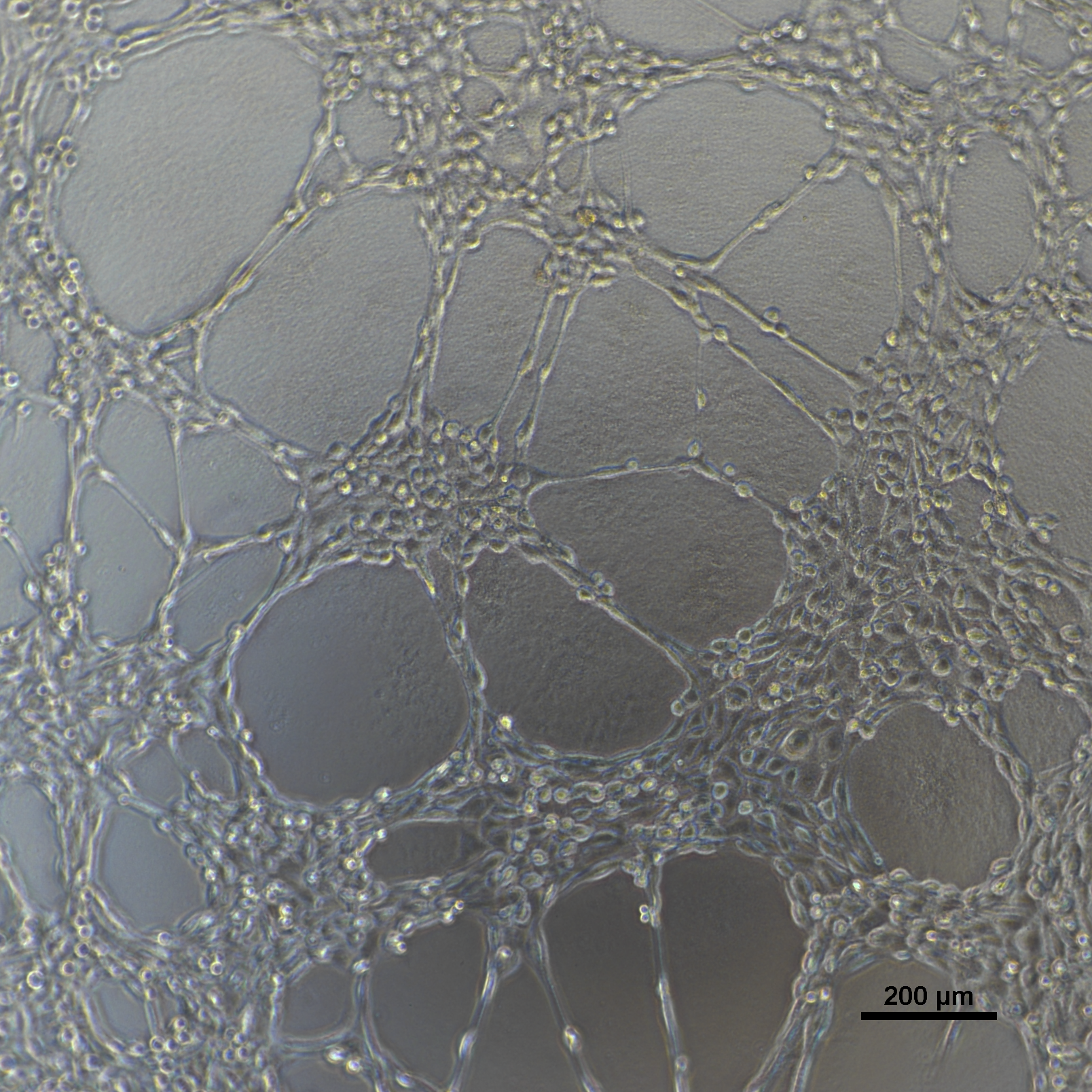

Supplement: Supplemental Information 47 [file peerj-10-13498-s047.tif]

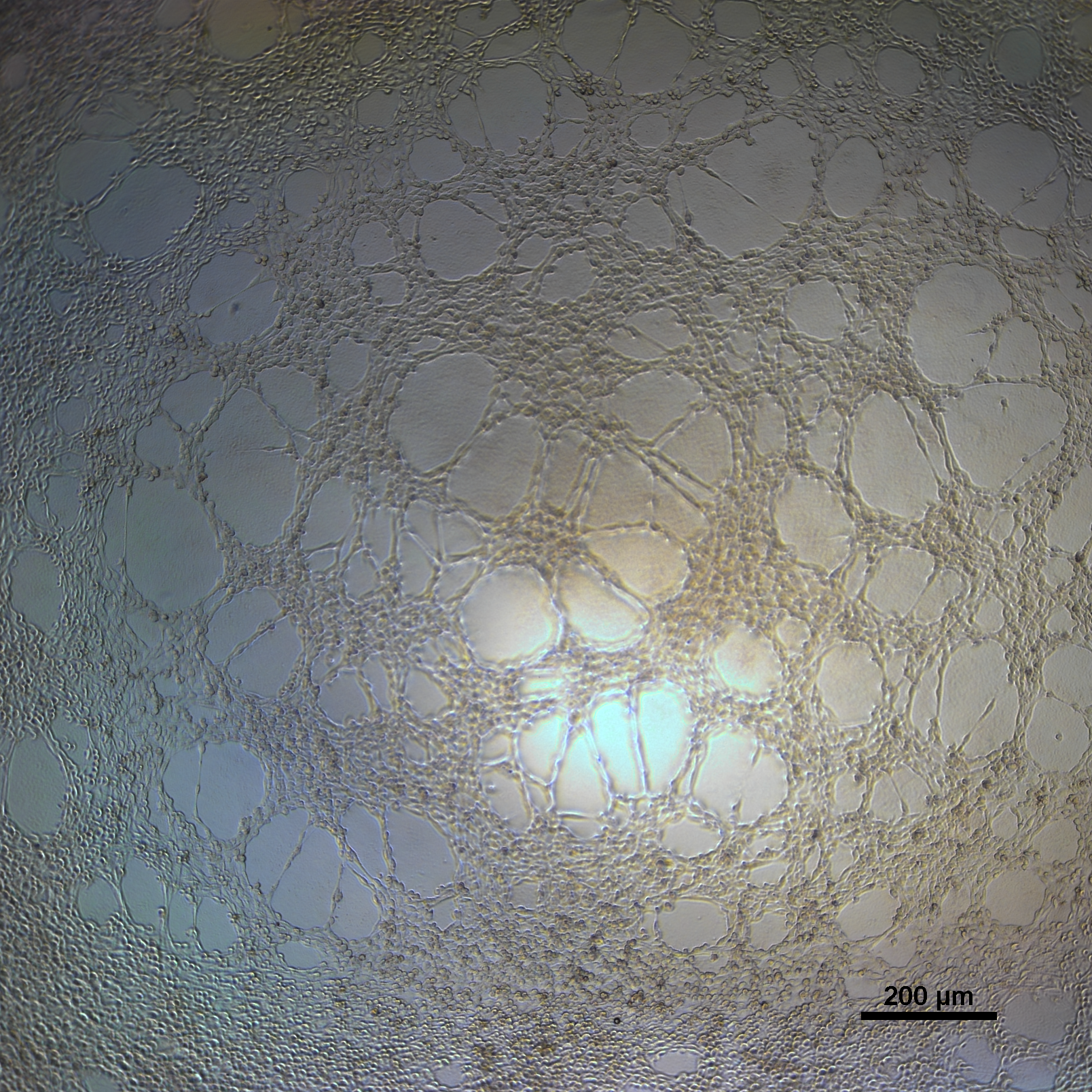

Supplement: Supplemental Information 48 [file peerj-10-13498-s048.tif]

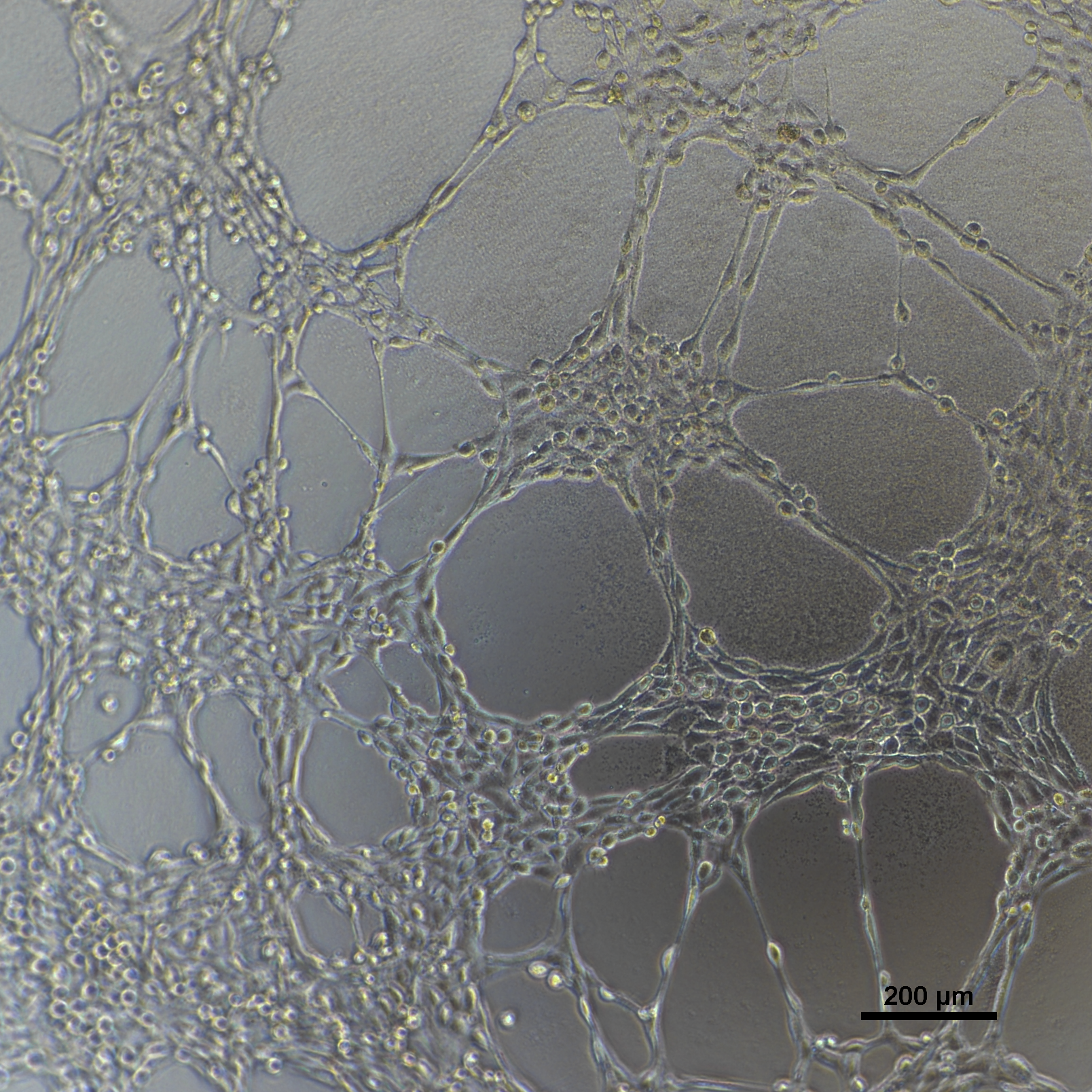

Supplement: Supplemental Information 49 [file peerj-10-13498-s049.tif]

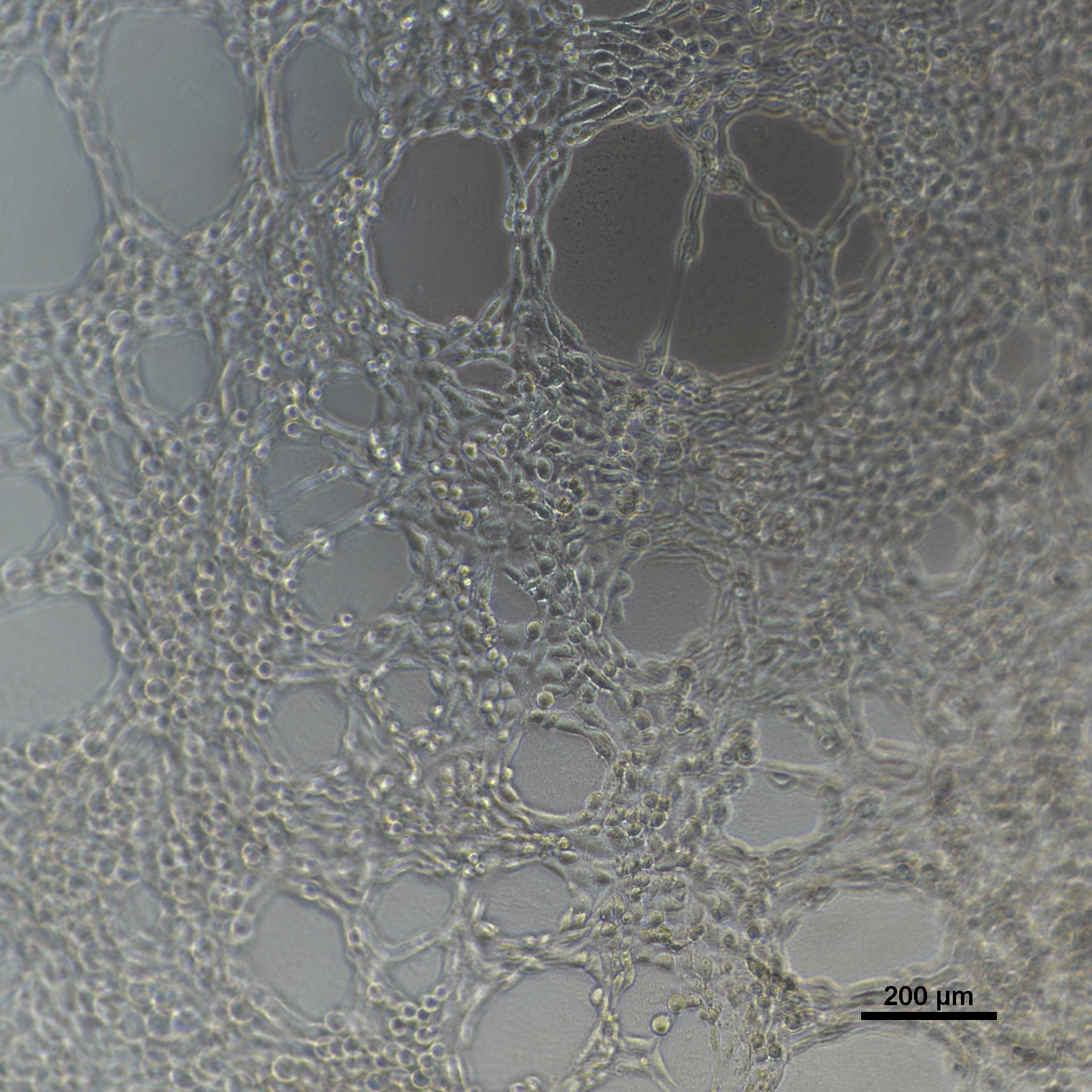

Supplement: Supplemental Information 50 [file peerj-10-13498-s050.tif]
