# Supplementary material for: Analysis of microRNA expression profiles in exosomes derived from acute myeloid leukemia by p62 knockdown and effect on angiogenesis
Source: PeerJ. 2022 Jul 22;10:e13498. doi: 10.7717/peerj.13498 (PMC9310811; doi:10.7717/peerj.13498)
Supplement: Supplemental Information 5 [file peerj-10-13498-s005.zip › 4.flow cytometry/1.pdf]

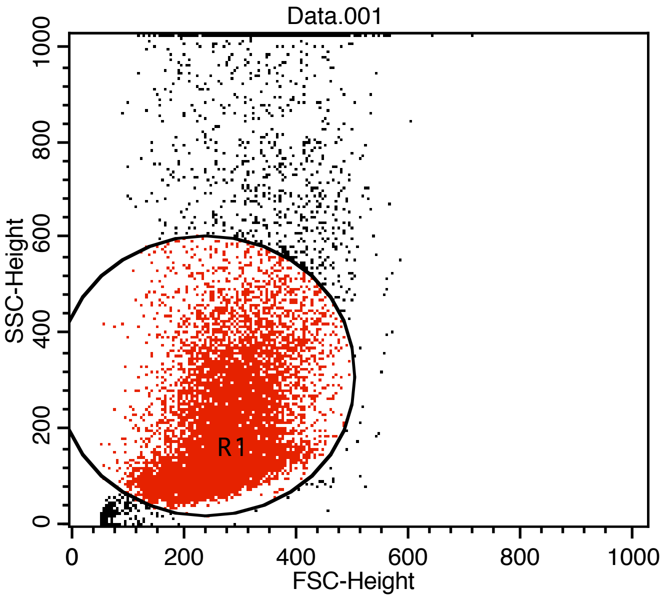

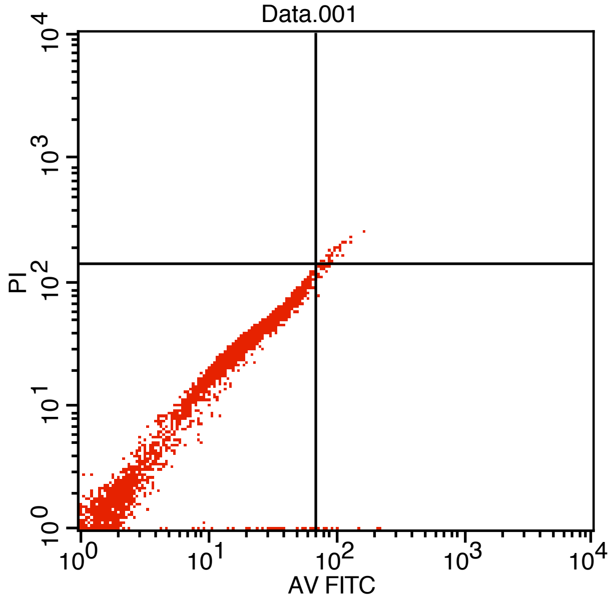

Quadrant Statistics

File: Data.001	Gate: G1
Gated Events: 10000	Total Events: 11300
X Parameter: AV FITC (Log)	Y Parameter: PI (Log)

Quad	Events	% Gated	% Total	X Mean	Y Mean
UL	0	0.00	0.00	***	***
UR	33	0.33	0.29	98.37	169.35
LL	9930	99.30	87.88	19.83	31.44
LR	37	0.37	0.33	87.92	91.51
