# Supplementary material for: Analysis of microRNA expression profiles in exosomes derived from acute myeloid leukemia by p62 knockdown and effect on angiogenesis
Source: PeerJ. 2022 Jul 22;10:e13498. doi: 10.7717/peerj.13498 (PMC9310811; doi:10.7717/peerj.13498)
Supplement: Supplemental Information 5 [file peerj-10-13498-s005.zip › 4.flow cytometry/2transfection pi.pdf]

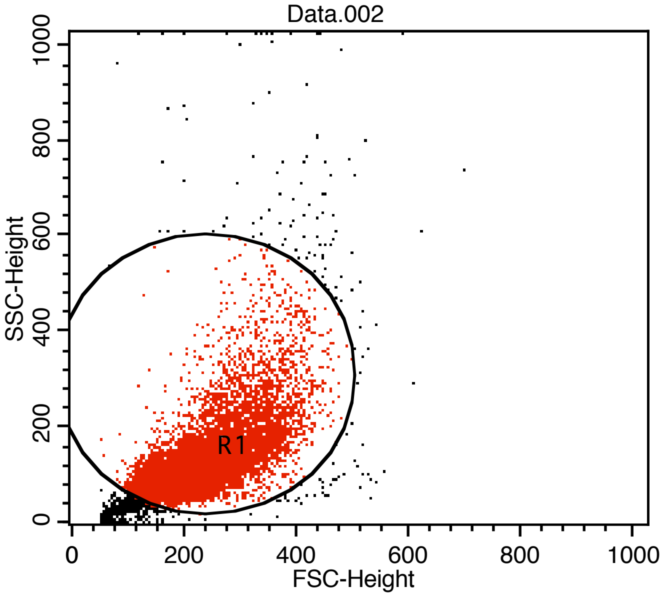

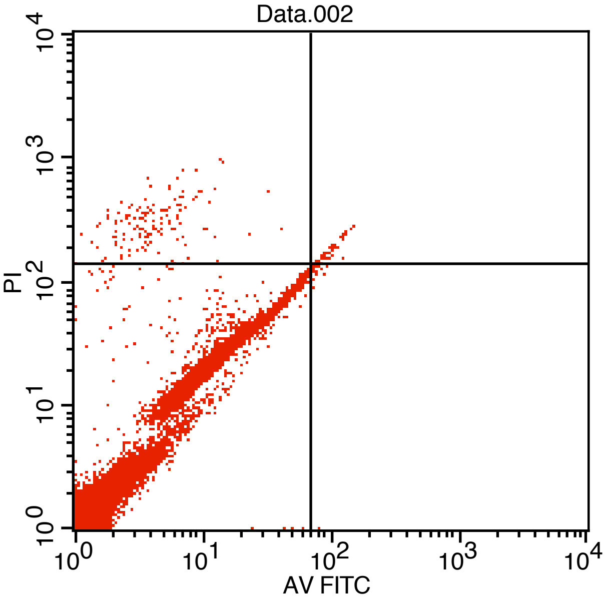

Quadrant Statistics

File: Data.002 — Gate: G1

Gated Events: 10000 — Total Events: 10593

X Parameter: AV FITC (Log) — Y Parameter: PI (Log)

Quad	Events	% Gated	% Total	X Mean	Y Mean
UL	129	1.29	1.22	5.19	326.07
UR	45	0.45	0.42	100.21	179.55
LL	9799	97.99	92.50	9.98	17.12
LR	27	0.27	0.25	76.93	113.90
