# Supplementary material for: Analysis of microRNA expression profiles in exosomes derived from acute myeloid leukemia by p62 knockdown and effect on angiogenesis
Source: PeerJ. 2022 Jul 22;10:e13498. doi: 10.7717/peerj.13498 (PMC9310811; doi:10.7717/peerj.13498)
Supplement: Supplemental Information 5 [file peerj-10-13498-s005.zip › 4.flow cytometry/3no transfection.pdf]

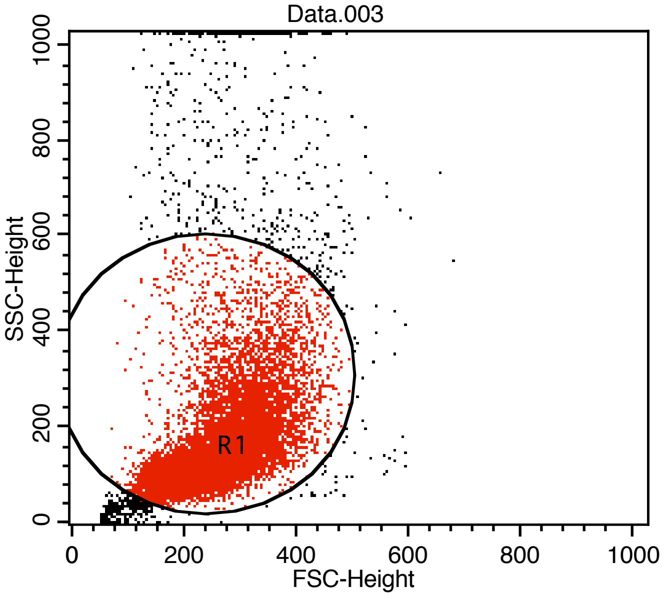

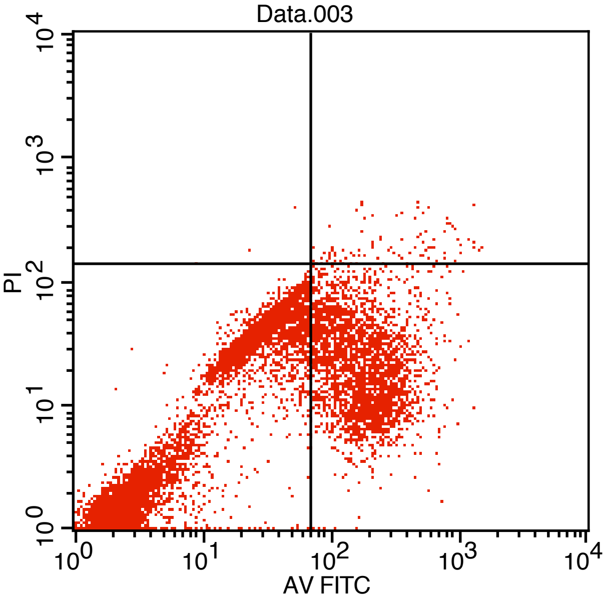

Quadrant Statistics

File: Data.003 Gate: G1
Gated Events: 10000 Total Events: 10873
X Parameter: AV FITC (Log) Y Parameter: PI (Log)

Quad	Events	% Gated	% Total	X Mean	Y Mean
UL	3	0.03	0.03	27.82	233.09
UR	87	0.87	0.80	435.70	213.63
LL	7170	71.70	65.94	17.69	20.75
LR	2740	27.40	25.20	205.48	28.17
