# Supplementary material for: Analysis of microRNA expression profiles in exosomes derived from acute myeloid leukemia by p62 knockdown and effect on angiogenesis
Source: PeerJ. 2022 Jul 22;10:e13498. doi: 10.7717/peerj.13498 (PMC9310811; doi:10.7717/peerj.13498)
Supplement: Supplemental Information 5 [file peerj-10-13498-s005.zip › 4.flow cytometry/4con.pdf]

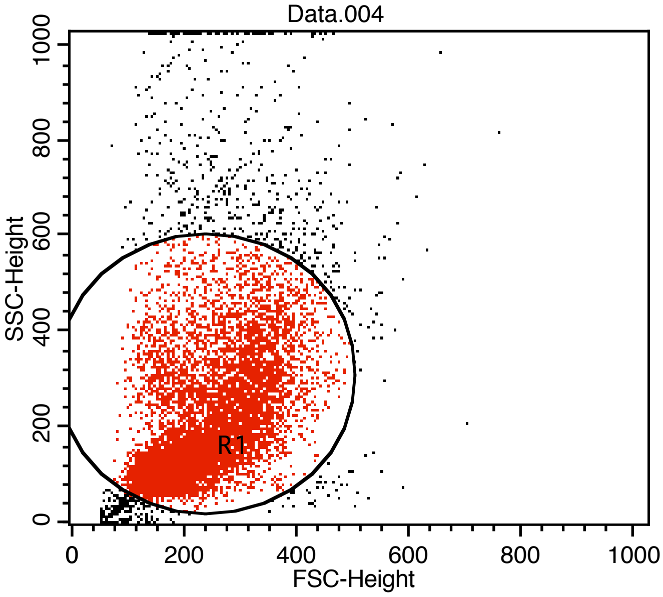

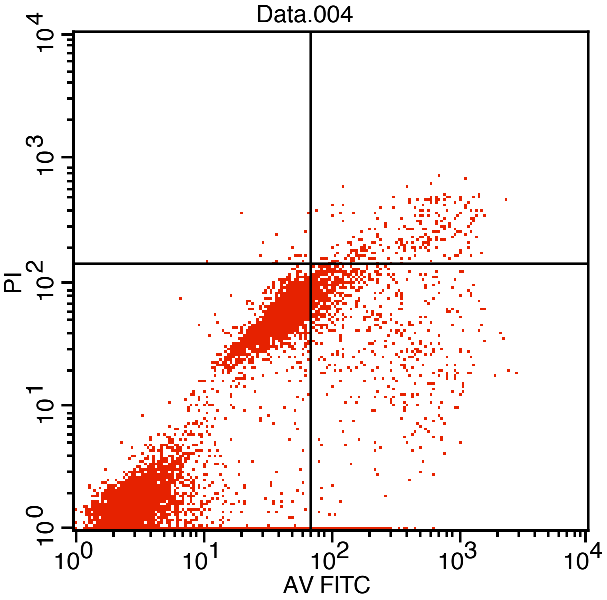

Quadrant Statistics

File: Data.004 Gate: G1

Gated Events: 10000 Total Events: 10795

X Parameter: AV FITC (Log) Y Parameter: PI (Log)

Quad	Events	% Gated	% Total	X Mean	Y Mean
UL	15	0.15	0.14	45.06	197.61
UR	231	2.31	2.14	524.46	264.96
LL	8633	86.33	79.97	21.48	23.41
LR	1121	11.21	10.38	220.82	46.97
