# Supplementary material for: Analysis of microRNA expression profiles in exosomes derived from acute myeloid leukemia by p62 knockdown and effect on angiogenesis
Source: PeerJ. 2022 Jul 22;10:e13498. doi: 10.7717/peerj.13498 (PMC9310811; doi:10.7717/peerj.13498)
Supplement: Supplemental Information 5 [file peerj-10-13498-s005.zip › 4.flow cytometry/5con.pdf]

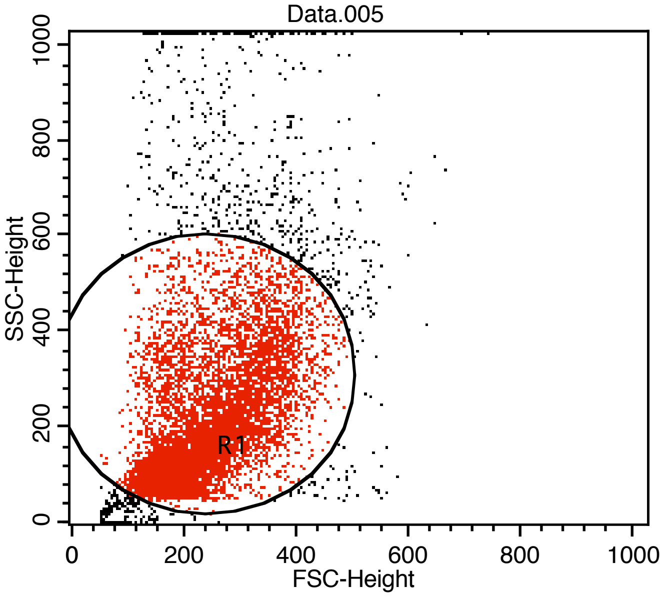

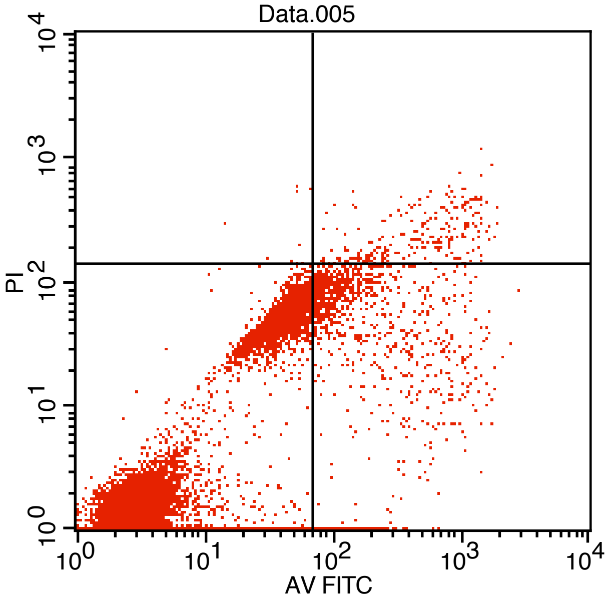

Quadrant Statistics

File: Data.005
Gated Events: 10000
X Parameter: AV FITC (Log)

Gate: G1
Total Events: 10800
Y Parameter: PI (Log)

Quad	Events	% Gated	% Total	X Mean	Y Mean
UL	8	0.08	0.07	46.00	323.74
UR	237	2.37	2.19	640.22	268.83
LL	8373	83.73	77.53	22.06	22.74
LR	1382	13.82	12.80	236.05	49.02
