# Supplementary material for: Analysis of microRNA expression profiles in exosomes derived from acute myeloid leukemia by p62 knockdown and effect on angiogenesis
Source: PeerJ. 2022 Jul 22;10:e13498. doi: 10.7717/peerj.13498 (PMC9310811; doi:10.7717/peerj.13498)
Supplement: Supplemental Information 5 [file peerj-10-13498-s005.zip › 4.flow cytometry/6,p62.pdf]

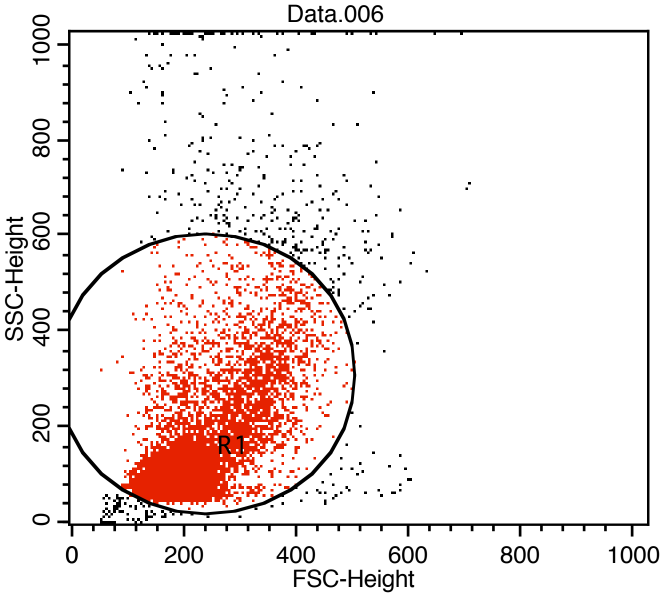

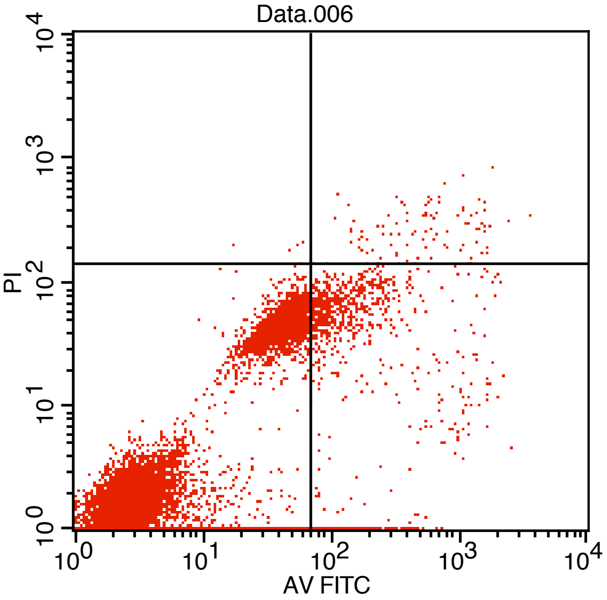

Quadrant Statistics

File: Data.006 Gate: G1

Gated Events: 10000 Total Events: 10487

X Parameter: AV FITC (Log) Y Parameter: PI (Log)

Quad	Events	% Gated	% Total	X Mean	Y Mean
UL	4	0.04	0.04	44.88	192.16
UR	97	0.97	0.92	724.00	285.98
LL	8941	89.41	85.26	14.58	12.02
LR	958	9.58	9.14	229.25	45.03
