# Supplementary material for: Analysis of microRNA expression profiles in exosomes derived from acute myeloid leukemia by p62 knockdown and effect on angiogenesis
Source: PeerJ. 2022 Jul 22;10:e13498. doi: 10.7717/peerj.13498 (PMC9310811; doi:10.7717/peerj.13498)
Supplement: Supplemental Information 5 [file peerj-10-13498-s005.zip › 4.flow cytometry/7p62.pdf]

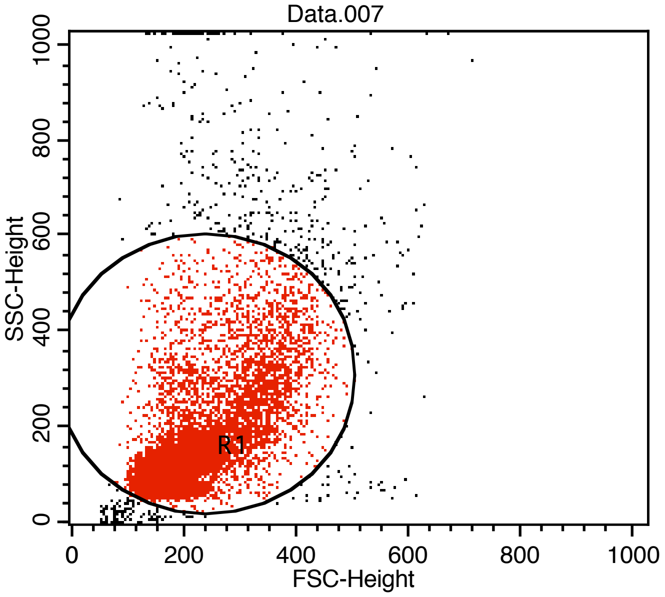

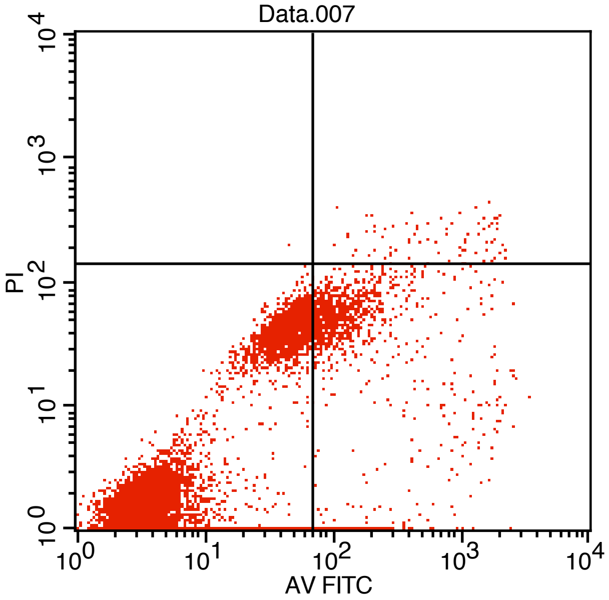

Quadrant Statistics

File: Data.007 | Gate: G1
Gated Events: 10000 | Total Events: 10521
X Parameter: AV FITC (Log) | Y Parameter: PI (Log)

Quad	Events	% Gated	% Total	X Mean	Y Mean
UL	1	0.01	0.01	44.51	191.10
UR	74	0.74	0.70	908.30	232.71
LL	8672	86.72	82.43	15.51	10.80
LR	1253	12.53	11.91	243.17	43.28
