# Supplementary material for: Analysis of microRNA expression profiles in exosomes derived from acute myeloid leukemia by p62 knockdown and effect on angiogenesis
Source: PeerJ. 2022 Jul 22;10:e13498. doi: 10.7717/peerj.13498 (PMC9310811; doi:10.7717/peerj.13498)
Supplement: Supplemental Information 5 [file peerj-10-13498-s005.zip › 4.flow cytometry/LC1126/1.pdf]

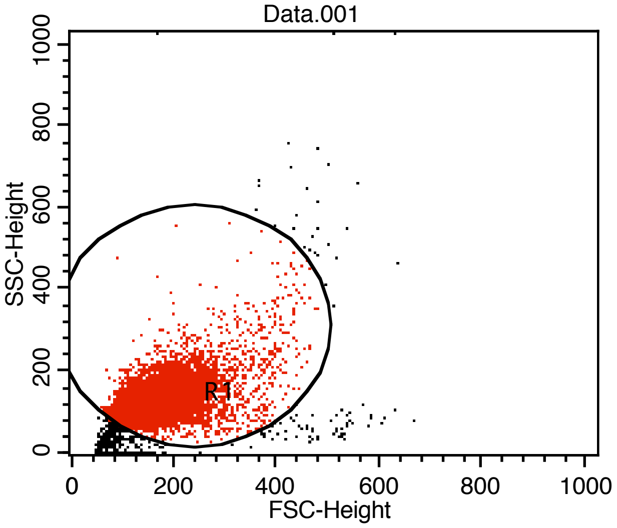

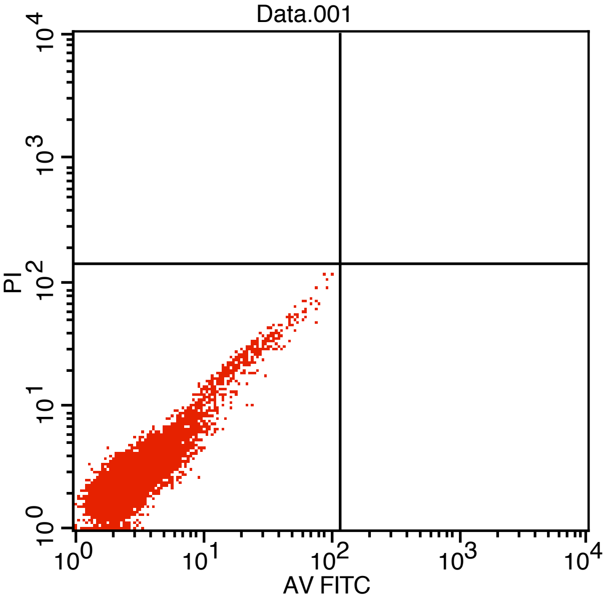

Quadrant Statistics

File: Data.001 Gate: G1

Gated Events: 10000 Total Events: 10471

X Parameter: AV FITC (Log) Y Parameter: PI (Log)

Quad	Events	% Gated	% Total	X Mean	Y Mean
UL	0	0.00	0.00	***	***
UR	0	0.00	0.00	***	***
LL	10000	100.00	95.50	4.01	3.75
LR	0	0.00	0.00	***	***
