# Supplementary material for: Analysis of microRNA expression profiles in exosomes derived from acute myeloid leukemia by p62 knockdown and effect on angiogenesis
Source: PeerJ. 2022 Jul 22;10:e13498. doi: 10.7717/peerj.13498 (PMC9310811; doi:10.7717/peerj.13498)
Supplement: Supplemental Information 5 [file peerj-10-13498-s005.zip › 4.flow cytometry/LC1126/10.pdf]

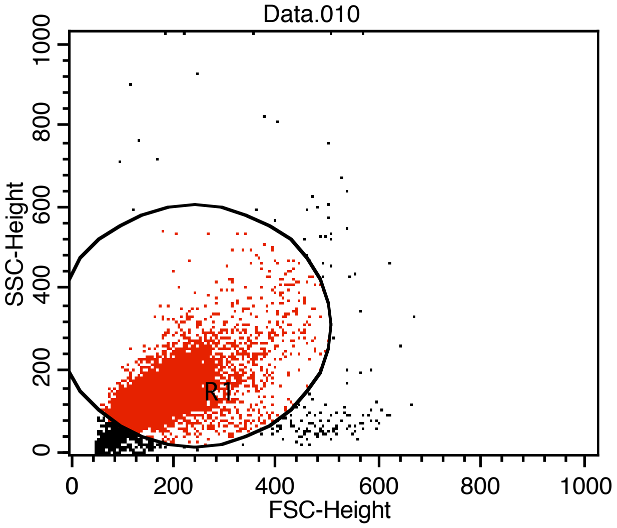

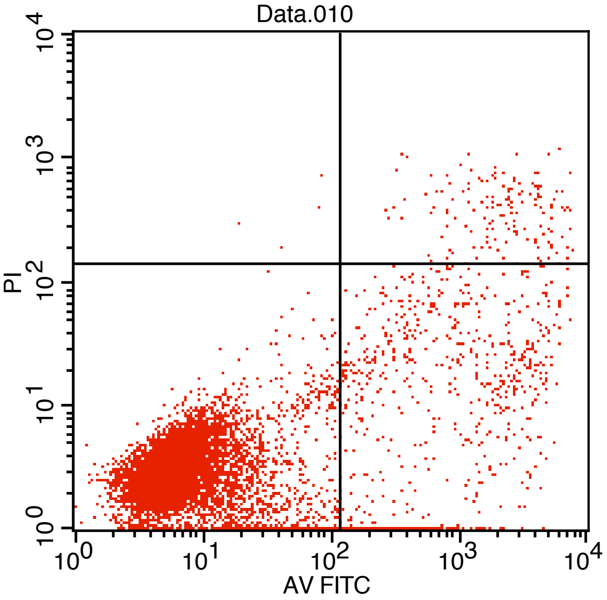

Quadrant Statistics

File: Data.010
Gate: G1
Gated Events: 10000
Total Events: 10644
X Parameter: AV FITC (Log)
Y Parameter: PI (Log)

Quad	Events	% Gated	% Total	X Mean	Y Mean
UL	4	0.04	0.04	55.85	393.70
UR	149	1.49	1.40	2789.78	429.23
LL	8248	82.48	77.49	19.70	3.09
LR	1599	15.99	15.02	712.80	11.57
