# Supplementary material for: Analysis of microRNA expression profiles in exosomes derived from acute myeloid leukemia by p62 knockdown and effect on angiogenesis
Source: PeerJ. 2022 Jul 22;10:e13498. doi: 10.7717/peerj.13498 (PMC9310811; doi:10.7717/peerj.13498)
Supplement: Supplemental Information 5 [file peerj-10-13498-s005.zip › 4.flow cytometry/LC1126/11.pdf]

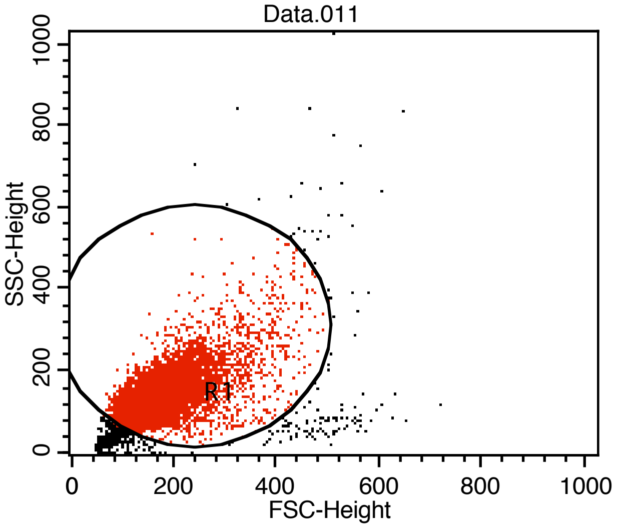

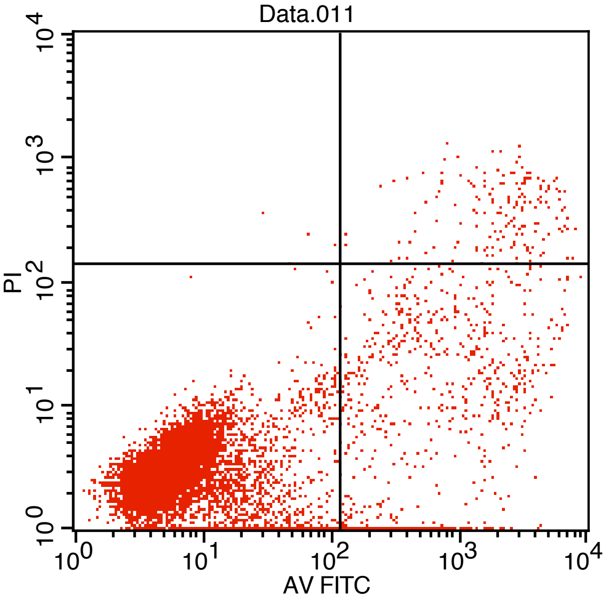

Quadrant Statistics

File: Data.011 Gate: G1
Gated Events: 10000 Total Events: 10545
X Parameter: AV FITC (Log) Y Parameter: PI (Log)

Quad	Events	% Gated	% Total	X Mean	Y Mean
UL	4	0.04	0.04	62.92	230.60
UR	162	1.62	1.54	2801.56	437.57
LL	8204	82.04	77.80	19.61	3.08
LR	1630	16.30	15.46	613.87	10.56
