# Supplementary material for: Analysis of microRNA expression profiles in exosomes derived from acute myeloid leukemia by p62 knockdown and effect on angiogenesis
Source: PeerJ. 2022 Jul 22;10:e13498. doi: 10.7717/peerj.13498 (PMC9310811; doi:10.7717/peerj.13498)
Supplement: Supplemental Information 5 [file peerj-10-13498-s005.zip › 4.flow cytometry/LC1126/2ta.pdf]

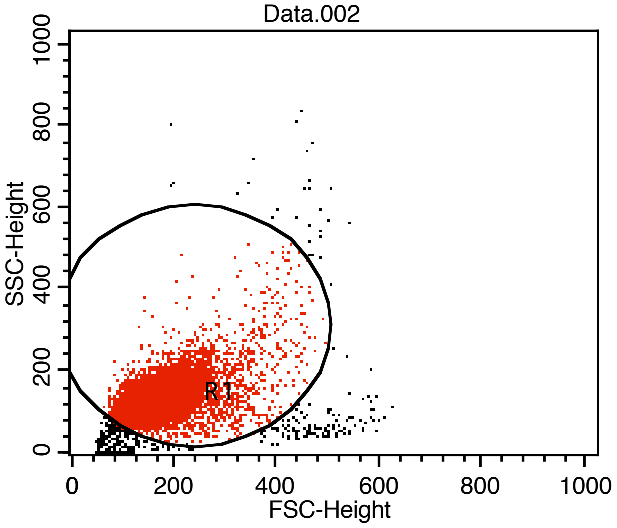

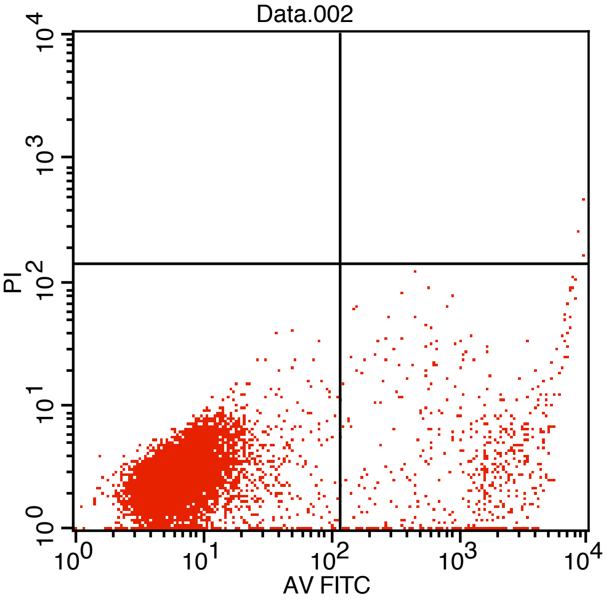

Quadrant Statistics

File: Data.002 Gate: G1
Gated Events: 10000 Total Events: 10432
X Parameter: AV FITC (Log) Y Parameter: PI (Log)

Quad	Events	% Gated	% Total	X Mean	Y Mean
UL	0	0.00	0.00	***	***
UR	3	0.03	0.03	9286.12	285.59
LL	9432	94.32	90.41	7.91	2.79
LR	565	5.65	5.42	1958.60	8.04
