# Supplementary material for: Analysis of microRNA expression profiles in exosomes derived from acute myeloid leukemia by p62 knockdown and effect on angiogenesis
Source: PeerJ. 2022 Jul 22;10:e13498. doi: 10.7717/peerj.13498 (PMC9310811; doi:10.7717/peerj.13498)
Supplement: Supplemental Information 5 [file peerj-10-13498-s005.zip › 4.flow cytometry/LC1126/3pi.pdf]

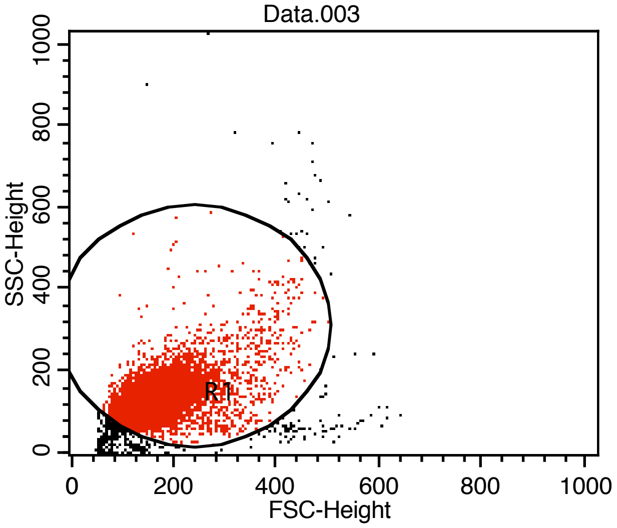

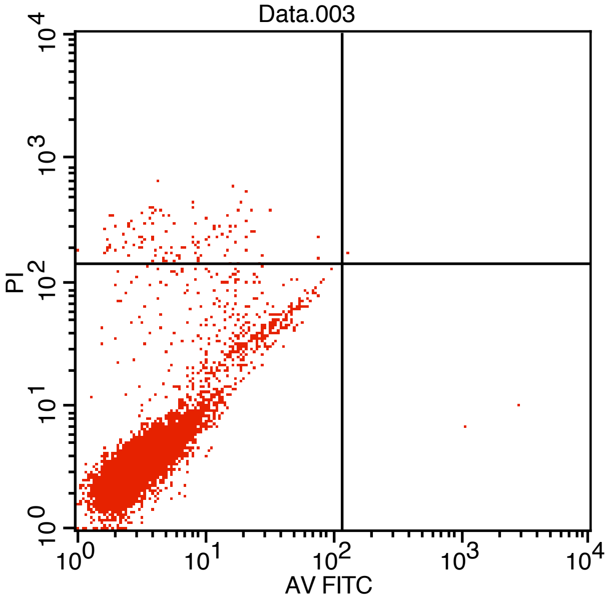

Quadrant Statistics

File: Data.003 Gate: G1
Gated Events: 10000 Total Events: 10368
X Parameter: AV FITC (Log) Y Parameter: PI (Log)

Quad	Events	% Gated	% Total	X Mean	Y Mean
UL	108	1.08	1.04	9.42	244.39
UR	1	0.01	0.01	127.49	164.00
LL	9889	98.89	95.38	4.21	5.40
LR	2	0.02	0.02	1958.66	8.35
