# Supplementary material for: Analysis of microRNA expression profiles in exosomes derived from acute myeloid leukemia by p62 knockdown and effect on angiogenesis
Source: PeerJ. 2022 Jul 22;10:e13498. doi: 10.7717/peerj.13498 (PMC9310811; doi:10.7717/peerj.13498)
Supplement: Supplemental Information 5 [file peerj-10-13498-s005.zip › 4.flow cytometry/LC1126/4.pdf]

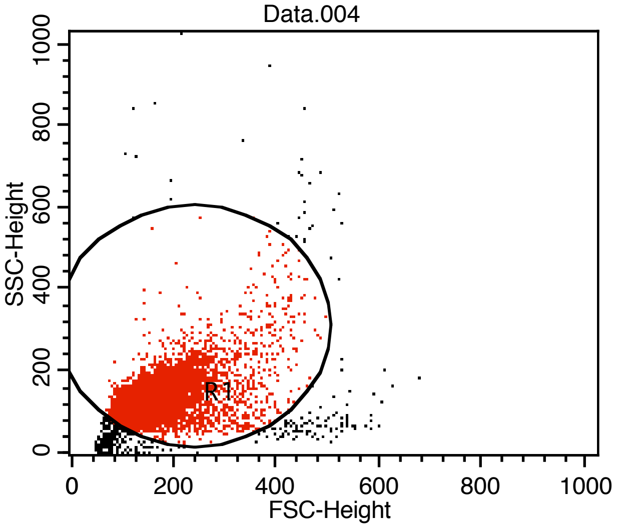

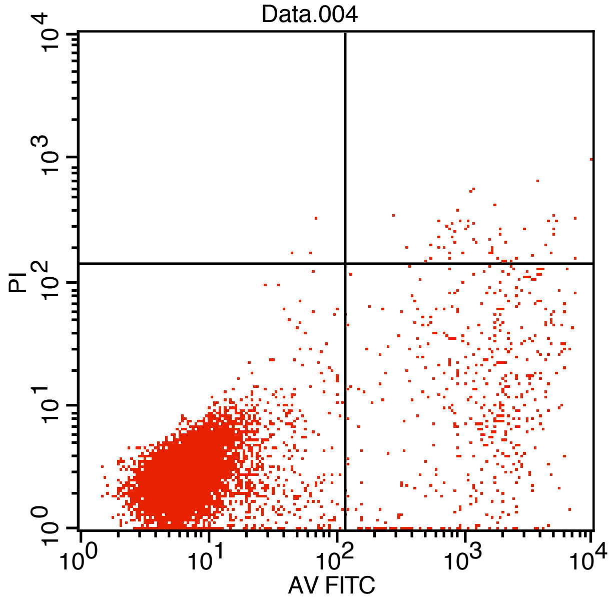

Quadrant Statistics

File: Data.004 Gate: G1

Gated Events: 10000 Total Events: 10363

X Parameter: AV FITC (Log) Y Parameter: PI (Log)

Quad	Events	% Gated	% Total	X Mean	Y Mean
UL	3	0.03	0.03	59.81	216.19
UR	57	0.57	0.55	2157.38	255.85
LL	9459	94.59	91.28	8.41	3.10
LR	481	4.81	4.64	1864.87	23.21
