# Supplementary material for: Analysis of microRNA expression profiles in exosomes derived from acute myeloid leukemia by p62 knockdown and effect on angiogenesis
Source: PeerJ. 2022 Jul 22;10:e13498. doi: 10.7717/peerj.13498 (PMC9310811; doi:10.7717/peerj.13498)
Supplement: Supplemental Information 5 [file peerj-10-13498-s005.zip › 4.flow cytometry/LC1126/5.pdf]

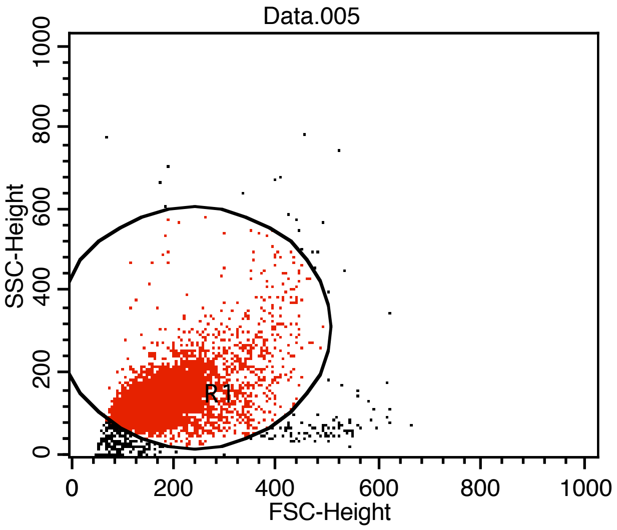

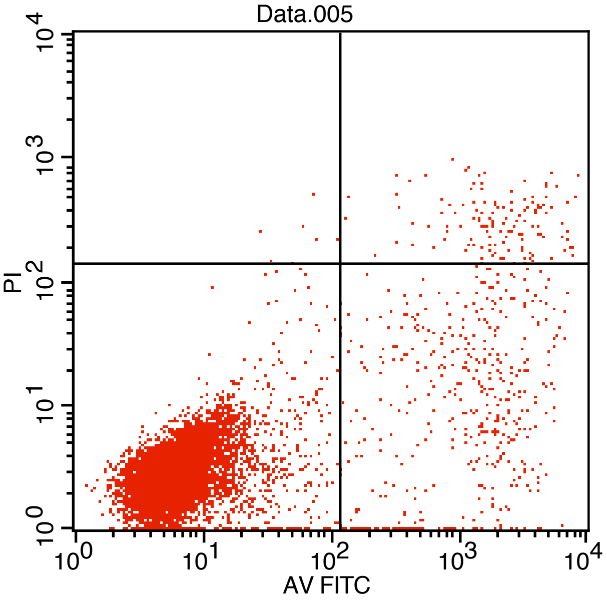

Quadrant Statistics

File: Data.005 | Gate: G1
Gated Events: 10000 | Total Events: 10352
X Parameter: AV FITC (Log) | Y Parameter: PI (Log)

Quad	Events	% Gated	% Total	X Mean	Y Mean
UL	7	0.07	0.07	61.51	248.62
UR	132	1.32	1.28	2685.54	344.70
LL	9418	94.18	90.98	8.02	3.34
LR	443	4.43	4.28	1629.01	21.87
