# Supplementary material for: Analysis of microRNA expression profiles in exosomes derived from acute myeloid leukemia by p62 knockdown and effect on angiogenesis
Source: PeerJ. 2022 Jul 22;10:e13498. doi: 10.7717/peerj.13498 (PMC9310811; doi:10.7717/peerj.13498)
Supplement: Supplemental Information 5 [file peerj-10-13498-s005.zip › 4.flow cytometry/LC1126/6.pdf]

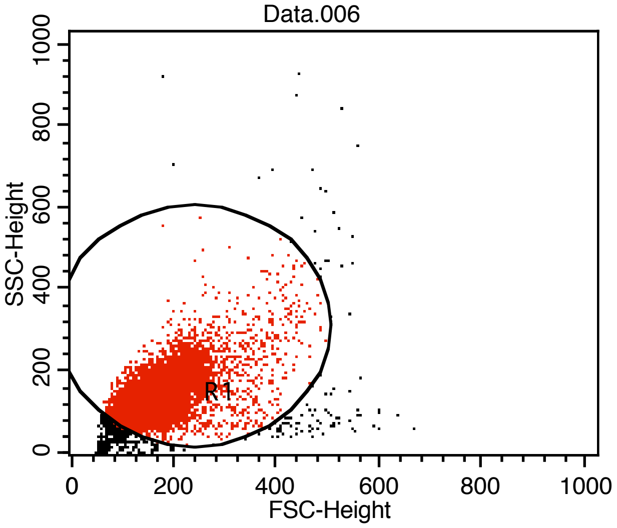

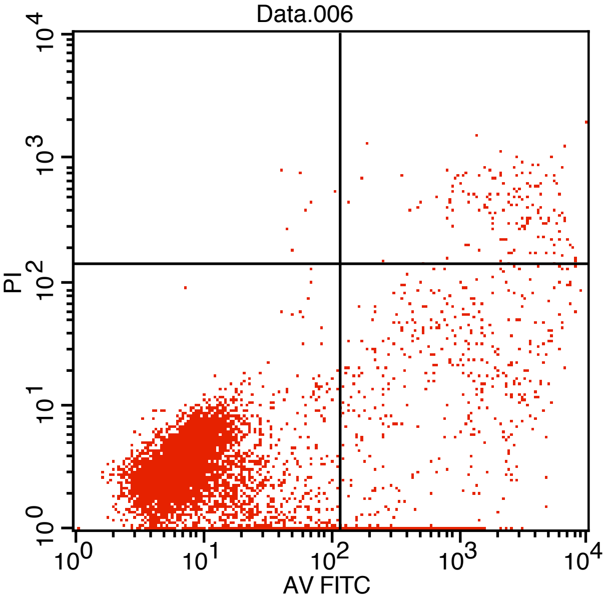

Quadrant Statistics

File: Data.006
Gate: G1
Gated Events: 10000
Total Events: 10456
X Parameter: AV FITC (Log)
Y Parameter: PI (Log)

Quad	Events	% Gated	% Total	X Mean	Y Mean
UL	7	0.07	0.07	61.86	473.99
UR	147	1.47	1.41	3070.59	469.24
LL	7527	75.27	71.99	21.93	3.06
LR	2319	23.19	22.18	530.86	5.99
