# Supplementary material for: Analysis of microRNA expression profiles in exosomes derived from acute myeloid leukemia by p62 knockdown and effect on angiogenesis
Source: PeerJ. 2022 Jul 22;10:e13498. doi: 10.7717/peerj.13498 (PMC9310811; doi:10.7717/peerj.13498)
Supplement: Supplemental Information 5 [file peerj-10-13498-s005.zip › 4.flow cytometry/LC1126/7.pdf]

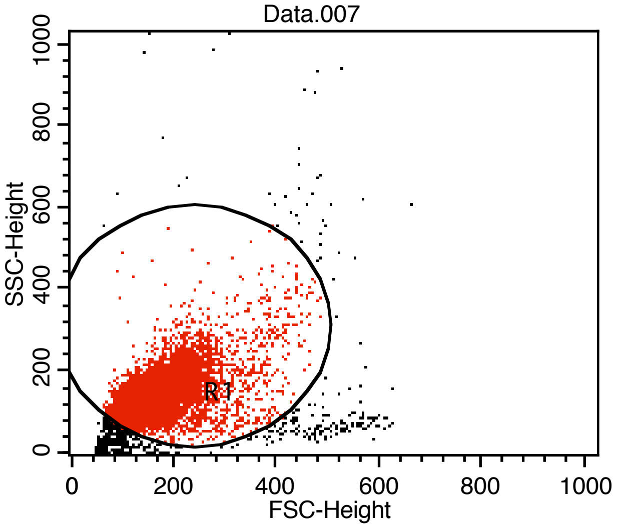

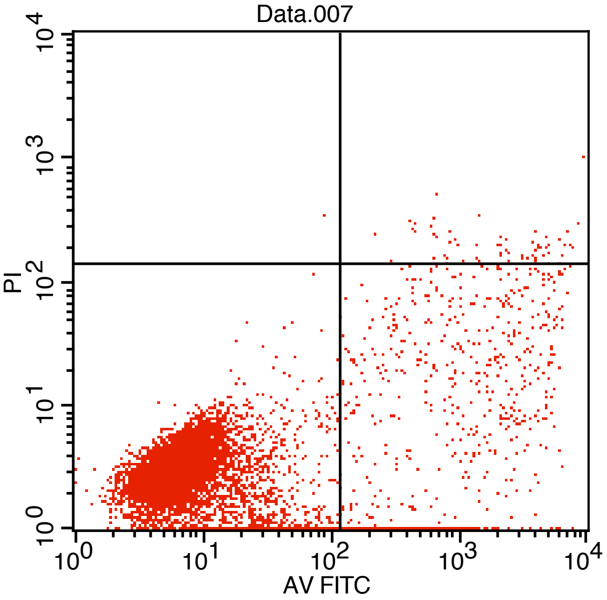

Quadrant Statistics

File: Data.007 Gate: G1
Gated Events: 10000 Total Events: 10624
X Parameter: AV FITC (Log) Y Parameter: PI (Log)

Quad	Events	% Gated	% Total	X Mean	Y Mean
UL	1	0.01	0.01	86.60	342.89
UR	60	0.60	0.56	3000.17	217.83
LL	7514	75.14	70.73	22.32	2.82
LR	2425	24.25	22.83	621.42	7.25
