# Supplementary material for: Analysis of microRNA expression profiles in exosomes derived from acute myeloid leukemia by p62 knockdown and effect on angiogenesis
Source: PeerJ. 2022 Jul 22;10:e13498. doi: 10.7717/peerj.13498 (PMC9310811; doi:10.7717/peerj.13498)
Supplement: Supplemental Information 5 [file peerj-10-13498-s005.zip › 4.flow cytometry/LC1126/8.pdf]

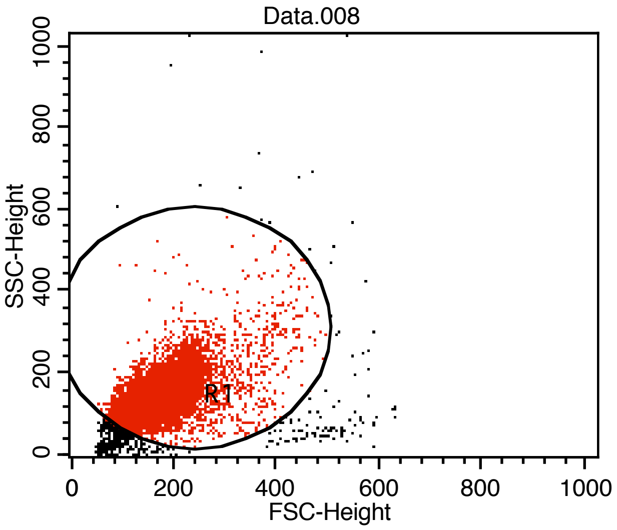

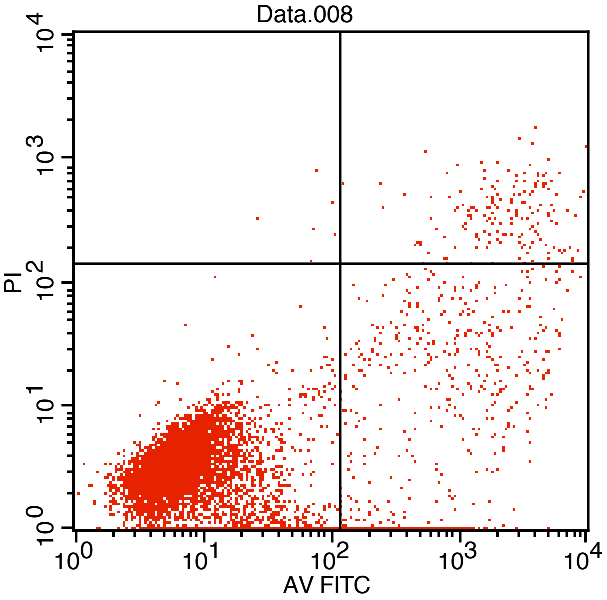

Quadrant Statistics

File: Data.008	Gate: G1
Gated Events: 10000	Total Events: 10500
X Parameter: AV FITC (Log)	Y Parameter: PI (Log)

Quad	Events	% Gated	% Total	X Mean	Y Mean
UL	6	0.06	0.06	75.46	363.17
UR	168	1.68	1.60	2944.26	416.43
LL	7492	74.92	71.35	21.84	3.03
LR	2334	23.34	22.23	488.26	5.93
