# Supplementary material for: Analysis of microRNA expression profiles in exosomes derived from acute myeloid leukemia by p62 knockdown and effect on angiogenesis
Source: PeerJ. 2022 Jul 22;10:e13498. doi: 10.7717/peerj.13498 (PMC9310811; doi:10.7717/peerj.13498)
Supplement: Supplemental Information 5 [file peerj-10-13498-s005.zip › 4.flow cytometry/LC1126/9.pdf]

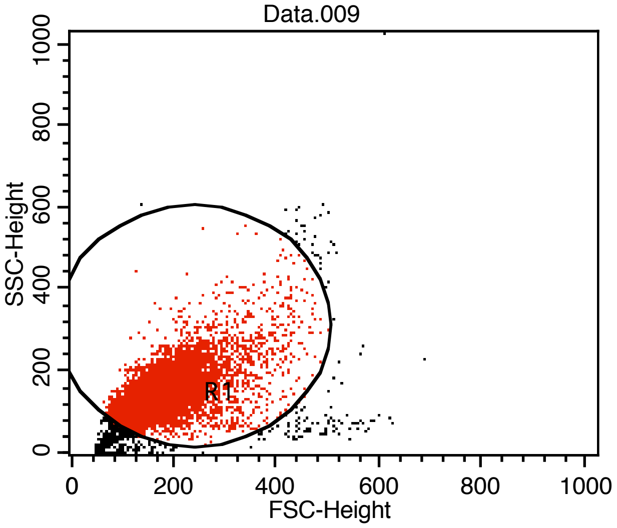

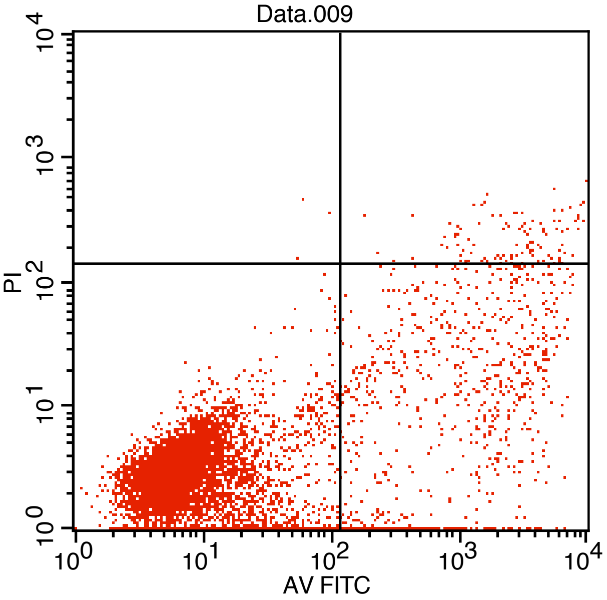

Quadrant Statistics

File: Data.009 | Gate: G1

Gated Events: 10000 | Total Events: 10600

X Parameter: AV FITC (Log) | Y Parameter: PI (Log)

Quad	Events	% Gated	% Total	X Mean	Y Mean
UL	3	0.03	0.03	70.58	323.15
UR	104	1.04	0.98	3505.57	241.55
LL	8161	81.61	76.99	20.62	2.88
LR	1732	17.32	16.34	851.61	12.12
