# Supplementary material for: Analysis of microRNA expression profiles in exosomes derived from acute myeloid leukemia by p62 knockdown and effect on angiogenesis
Source: PeerJ. 2022 Jul 22;10:e13498. doi: 10.7717/peerj.13498 (PMC9310811; doi:10.7717/peerj.13498)
Supplement: Supplemental Information 5 [file peerj-10-13498-s005.zip › 4.flow cytometry/LC1130/10.pdf]

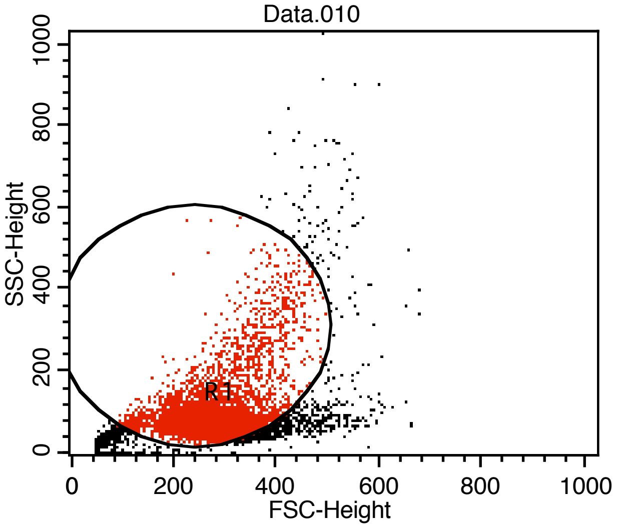

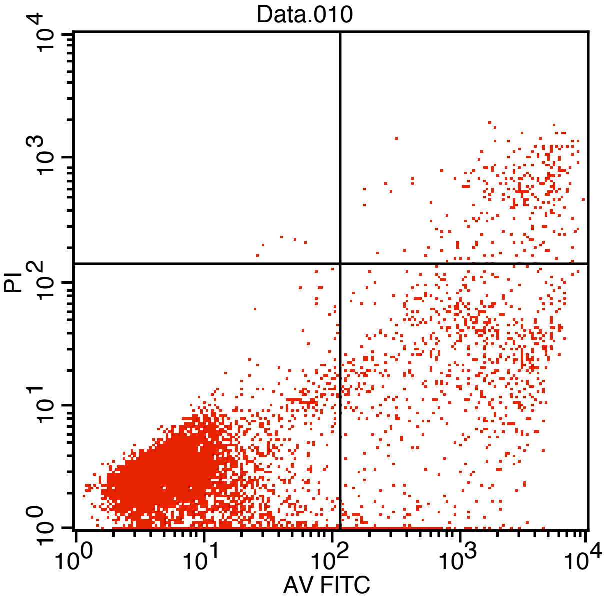

Quadrant Statistics

File: Data.010 Gate: G1
Gated Events: 10000 Total Events: 11033
X Parameter: AV FITC (Log) Y Parameter: PI (Log)

Quad	Events	% Gated	% Total	X Mean	Y Mean
UL	5	0.05	0.05	42.41	199.67
UR	277	2.77	2.51	3625.16	608.05
LL	8190	81.90	74.23	18.40	2.71
LR	1528	15.28	13.85	1022.69	16.61
