# Supplementary material for: Analysis of microRNA expression profiles in exosomes derived from acute myeloid leukemia by p62 knockdown and effect on angiogenesis
Source: PeerJ. 2022 Jul 22;10:e13498. doi: 10.7717/peerj.13498 (PMC9310811; doi:10.7717/peerj.13498)
Supplement: Supplemental Information 5 [file peerj-10-13498-s005.zip › 4.flow cytometry/LC1130/11.pdf]

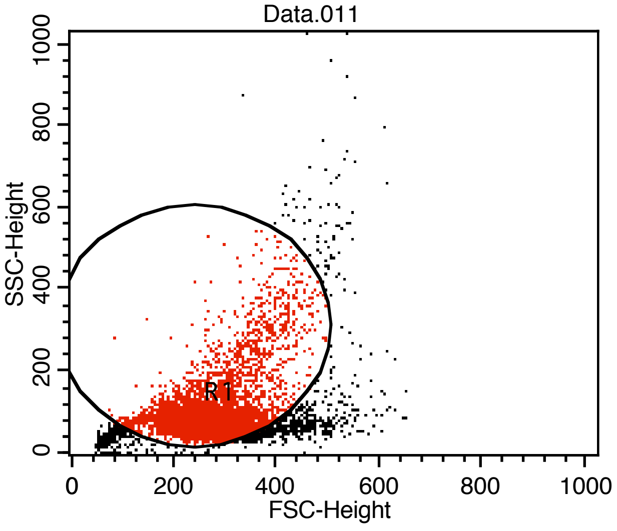

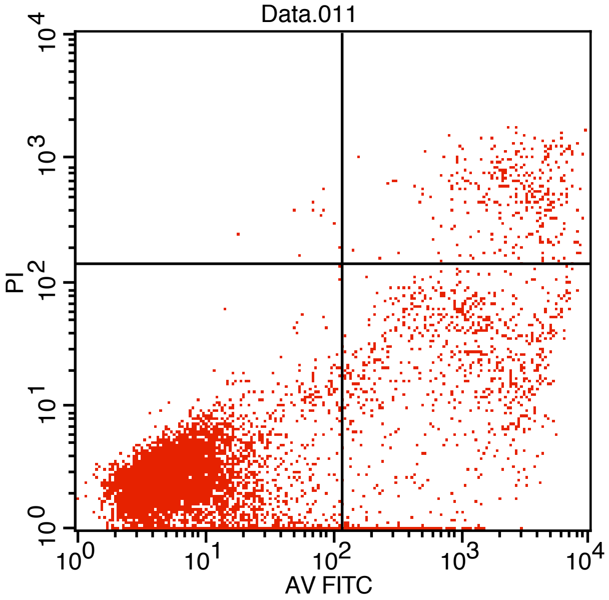

Quadrant Statistics

File: Data.011
Gate: G1
Gated Events: 10000
Total Events: 10987
X Parameter: AV FITC (Log)
Y Parameter: PI (Log)

Quad	Events	% Gated	% Total	X Mean	Y Mean
UL	10	0.10	0.09	72.49	334.01
UR	332	3.32	3.02	3437.06	612.02
LL	8085	80.85	73.59	18.51	2.61
LR	1573	15.73	14.32	984.68	16.46
