# Supplementary material for: Analysis of microRNA expression profiles in exosomes derived from acute myeloid leukemia by p62 knockdown and effect on angiogenesis
Source: PeerJ. 2022 Jul 22;10:e13498. doi: 10.7717/peerj.13498 (PMC9310811; doi:10.7717/peerj.13498)
Supplement: Supplemental Information 5 [file peerj-10-13498-s005.zip › 4.flow cytometry/LC1130/2.pdf]

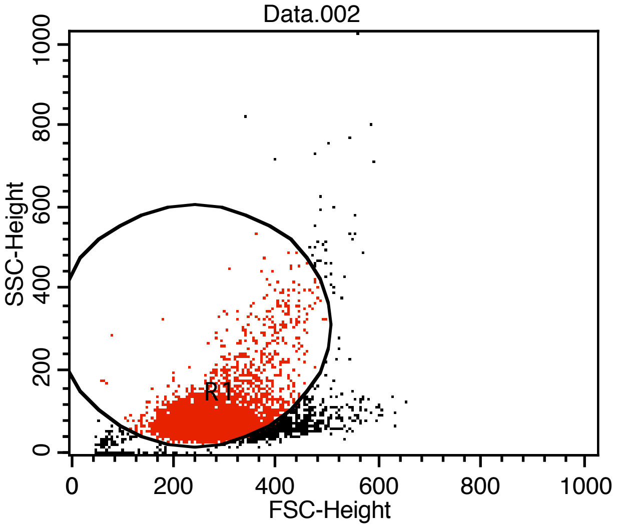

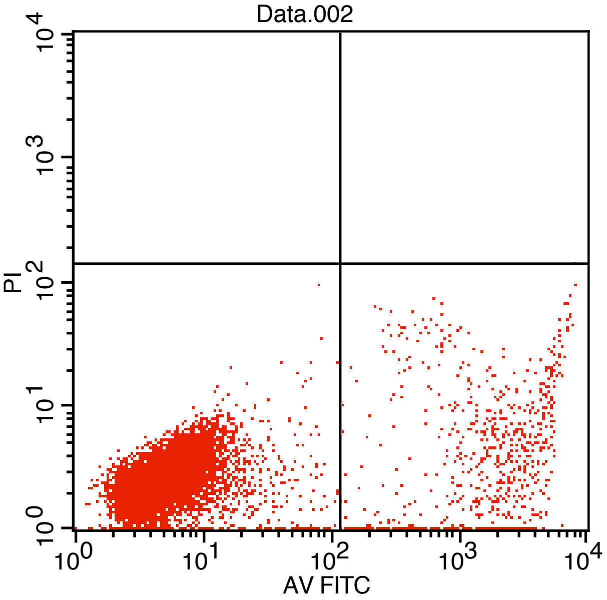

Quadrant Statistics

File: Data.002
Gate: G1
Gated Events: 10000
Total Events: 10679
X Parameter: AV FITC (Log)
Y Parameter: PI (Log)

Quad	Events	% Gated	% Total	X Mean	Y Mean
UL	0	0.00	0.00	***	***
UR	1	0.01	0.01	8131.23	138.24
LL	9266	92.66	86.77	6.42	2.75
LR	733	7.33	6.86	2252.62	8.18
