# Supplementary material for: Analysis of microRNA expression profiles in exosomes derived from acute myeloid leukemia by p62 knockdown and effect on angiogenesis
Source: PeerJ. 2022 Jul 22;10:e13498. doi: 10.7717/peerj.13498 (PMC9310811; doi:10.7717/peerj.13498)
Supplement: Supplemental Information 5 [file peerj-10-13498-s005.zip › 4.flow cytometry/LC1130/3.pdf]

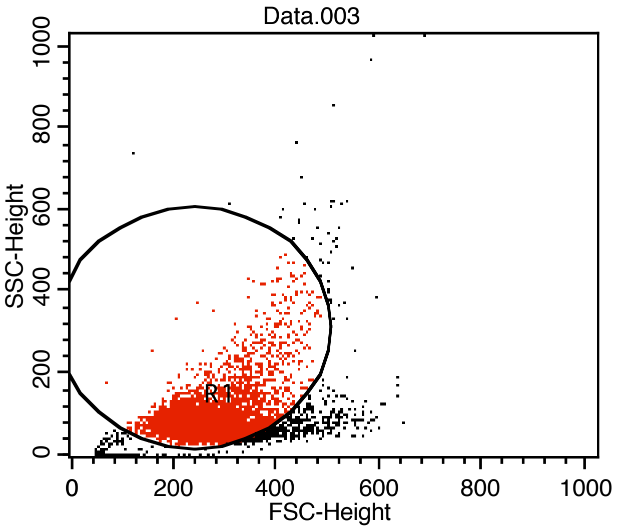


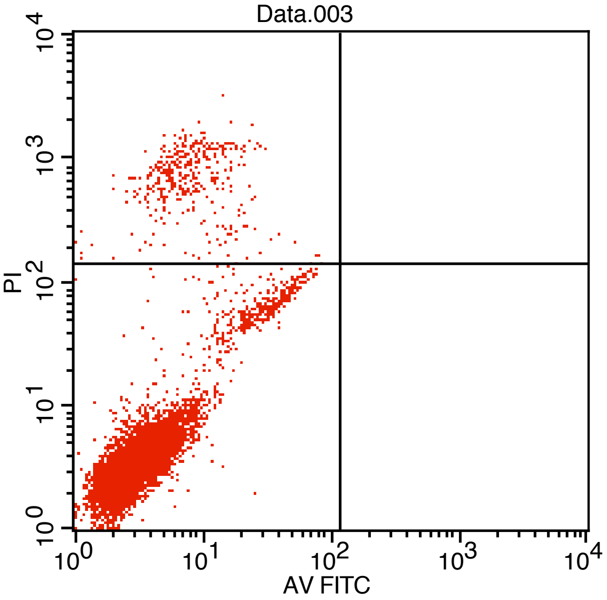


Quadrant Statistics

File: Data.003
Gate: G1
Gated Events: 10000
Total Events: 10613
X Parameter: AV FITC (Log)
Y Parameter: PI (Log)

| Quad | Events | % Gated | % Total | X Mean | Y Mean |
|---|---|---|---|---|---|
| UL | 361 | 3.61 | 3.40 | 9.54 | 773.56 |
| UR | 0 | 0.00 | 0.00 | *** | *** |
| LL | 9639 | 96.39 | 90.82 | 4.17 | 5.96 |
| LR | 0 | 0.00 | 0.00 | *** | *** |
