# Supplementary material for: Analysis of microRNA expression profiles in exosomes derived from acute myeloid leukemia by p62 knockdown and effect on angiogenesis
Source: PeerJ. 2022 Jul 22;10:e13498. doi: 10.7717/peerj.13498 (PMC9310811; doi:10.7717/peerj.13498)
Supplement: Supplemental Information 5 [file peerj-10-13498-s005.zip › 4.flow cytometry/LC1130/4.pdf]

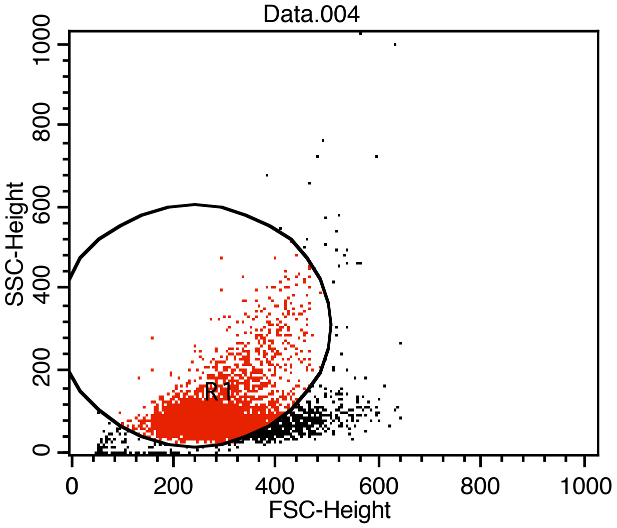

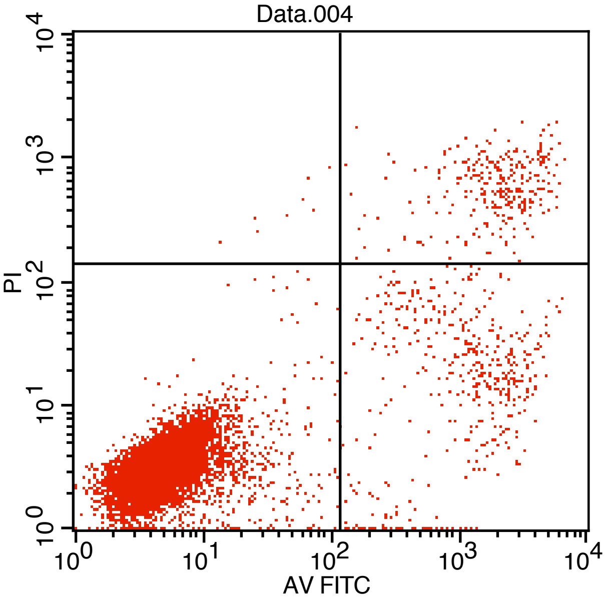

Quadrant Statistics

File: Data.004 — Gate: G1

Gated Events: 10000 — Total Events: 10698

X Parameter: AV FITC (Log) — Y Parameter: PI (Log)

Quad	Events	% Gated	% Total	X Mean	Y Mean
UL	8	0.08	0.07	50.79	427.06
UR	296	2.96	2.77	2466.64	653.65
LL	9240	92.40	86.37	5.95	3.48
LR	456	4.56	4.26	1457.87	27.74
