# Supplementary material for: Analysis of microRNA expression profiles in exosomes derived from acute myeloid leukemia by p62 knockdown and effect on angiogenesis
Source: PeerJ. 2022 Jul 22;10:e13498. doi: 10.7717/peerj.13498 (PMC9310811; doi:10.7717/peerj.13498)
Supplement: Supplemental Information 5 [file peerj-10-13498-s005.zip › 4.flow cytometry/LC1130/6.pdf]

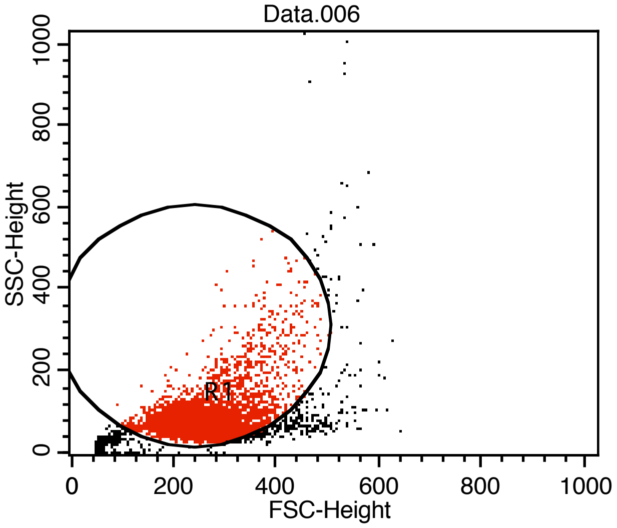

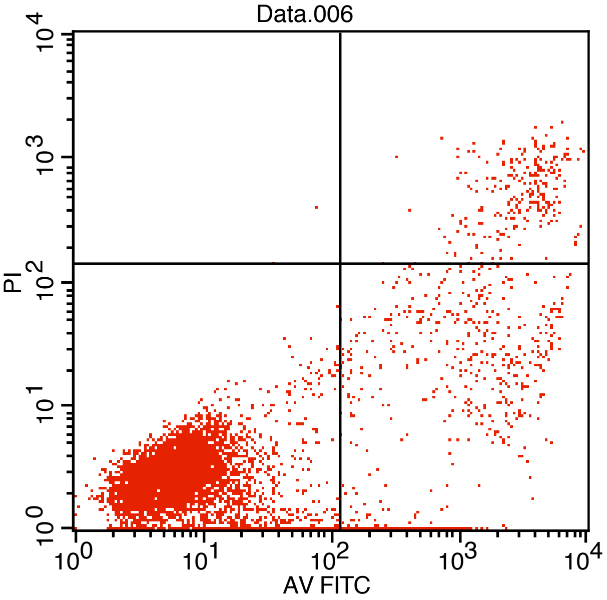

Quadrant Statistics

File: Data.006 Gate: G1
Gated Events: 10000 Total Events: 10529
X Parameter: AV FITC (Log) Y Parameter: PI (Log)

Quad	Events	% Gated	% Total	X Mean	Y Mean
UL	1	0.01	0.01	76.35	381.97
UR	265	2.65	2.52	3578.65	616.27
LL	7415	74.15	70.42	20.34	2.60
LR	2319	23.19	22.02	581.61	7.52
