# Supplementary material for: Analysis of microRNA expression profiles in exosomes derived from acute myeloid leukemia by p62 knockdown and effect on angiogenesis
Source: PeerJ. 2022 Jul 22;10:e13498. doi: 10.7717/peerj.13498 (PMC9310811; doi:10.7717/peerj.13498)
Supplement: Supplemental Information 5 [file peerj-10-13498-s005.zip › 4.flow cytometry/LC1130/7.pdf]

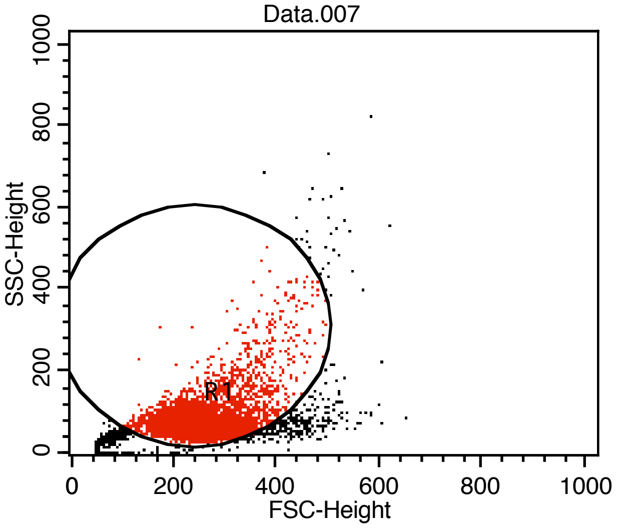

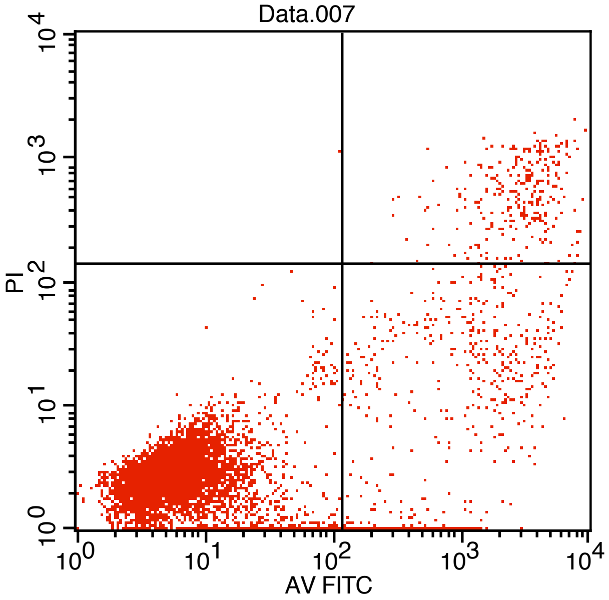

Quadrant Statistics

File: Data.007 Gate: G1
Gated Events: 10000 Total Events: 10552
X Parameter: AV FITC (Log) Y Parameter: PI (Log)

Quad	Events	% Gated	% Total	X Mean	Y Mean
UL	1	0.01	0.01	114.44	1134.19
UR	277	2.77	2.63	3375.05	603.35
LL	7475	74.75	70.84	20.50	2.63
LR	2247	22.47	21.29	543.76	6.39
