# Supplementary material for: Analysis of microRNA expression profiles in exosomes derived from acute myeloid leukemia by p62 knockdown and effect on angiogenesis
Source: PeerJ. 2022 Jul 22;10:e13498. doi: 10.7717/peerj.13498 (PMC9310811; doi:10.7717/peerj.13498)
Supplement: Supplemental Information 5 [file peerj-10-13498-s005.zip › 4.flow cytometry/LC1130/8.pdf]

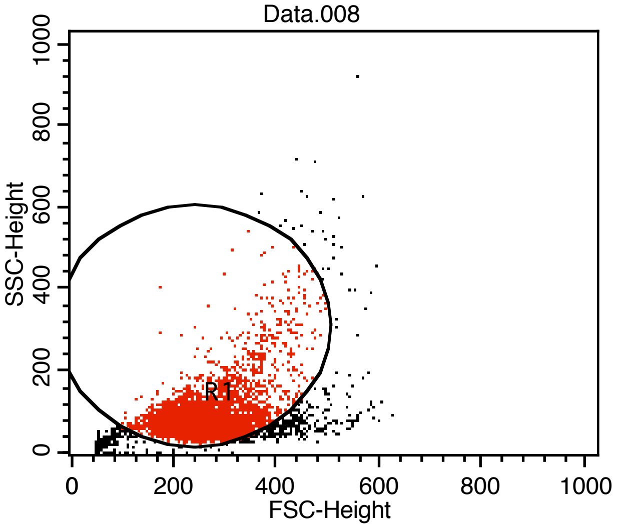

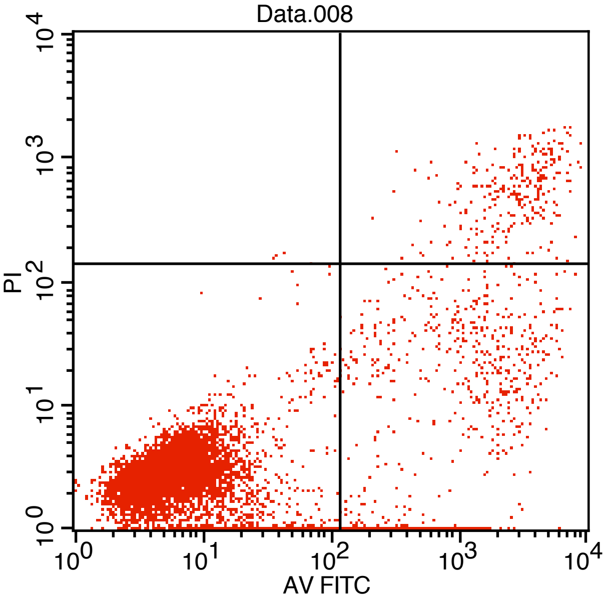

Quadrant Statistics

File: Data.008 Gate: G1
Gated Events: 10000 Total Events: 10500
X Parameter: AV FITC (Log) Y Parameter: PI (Log)

Quad	Events	% Gated	% Total	X Mean	Y Mean
UL	3	0.03	0.03	38.10	157.41
UR	276	2.76	2.63	3282.46	615.32
LL	7388	73.88	70.36	19.82	2.69
LR	2333	23.33	22.22	598.44	7.14
