# Supplementary material for: Analysis of microRNA expression profiles in exosomes derived from acute myeloid leukemia by p62 knockdown and effect on angiogenesis
Source: PeerJ. 2022 Jul 22;10:e13498. doi: 10.7717/peerj.13498 (PMC9310811; doi:10.7717/peerj.13498)
Supplement: Supplemental Information 5 [file peerj-10-13498-s005.zip › 4.flow cytometry/LC1130/9.pdf]

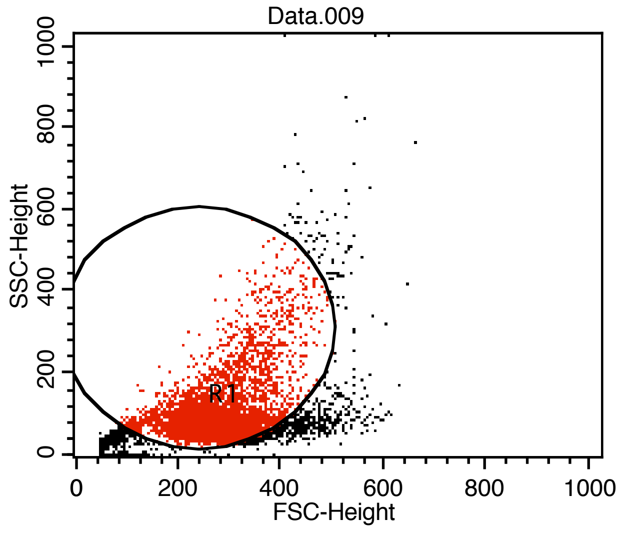

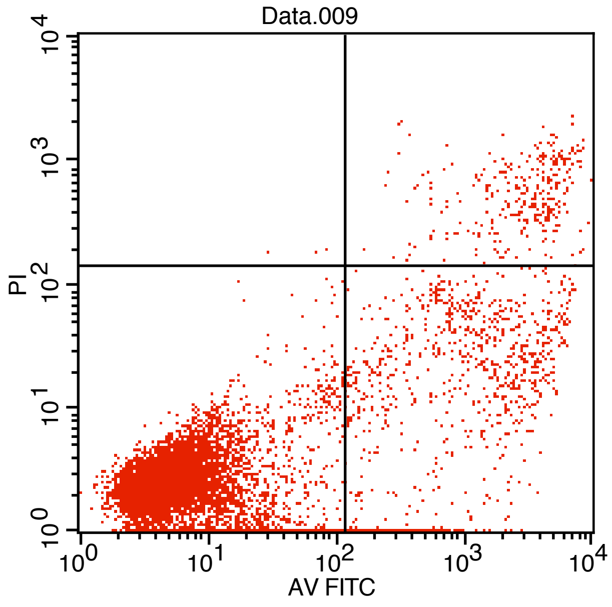

Quadrant Statistics

File: Data.009
Gate: G1
Gated Events: 10000
Total Events: 11015
X Parameter: AV FITC (Log)
Y Parameter: PI (Log)

Quad	Events	% Gated	% Total	X Mean	Y Mean
UL	3	0.03	0.03	60.83	178.42
UR	300	3.00	2.72	3541.38	622.14
LL	8231	82.31	74.73	17.95	2.72
LR	1466	14.66	13.31	1043.40	17.62
