# Supplementary figures and images for: Analysis of microRNA expression profiles in exosomes derived from acute myeloid leukemia by p62 knockdown and effect on angiogenesis (part 2 of 2)
Source: PeerJ. 2022 Jul 22;10:e13498. doi: 10.7717/peerj.13498 (PMC9310811; doi:10.7717/peerj.13498)

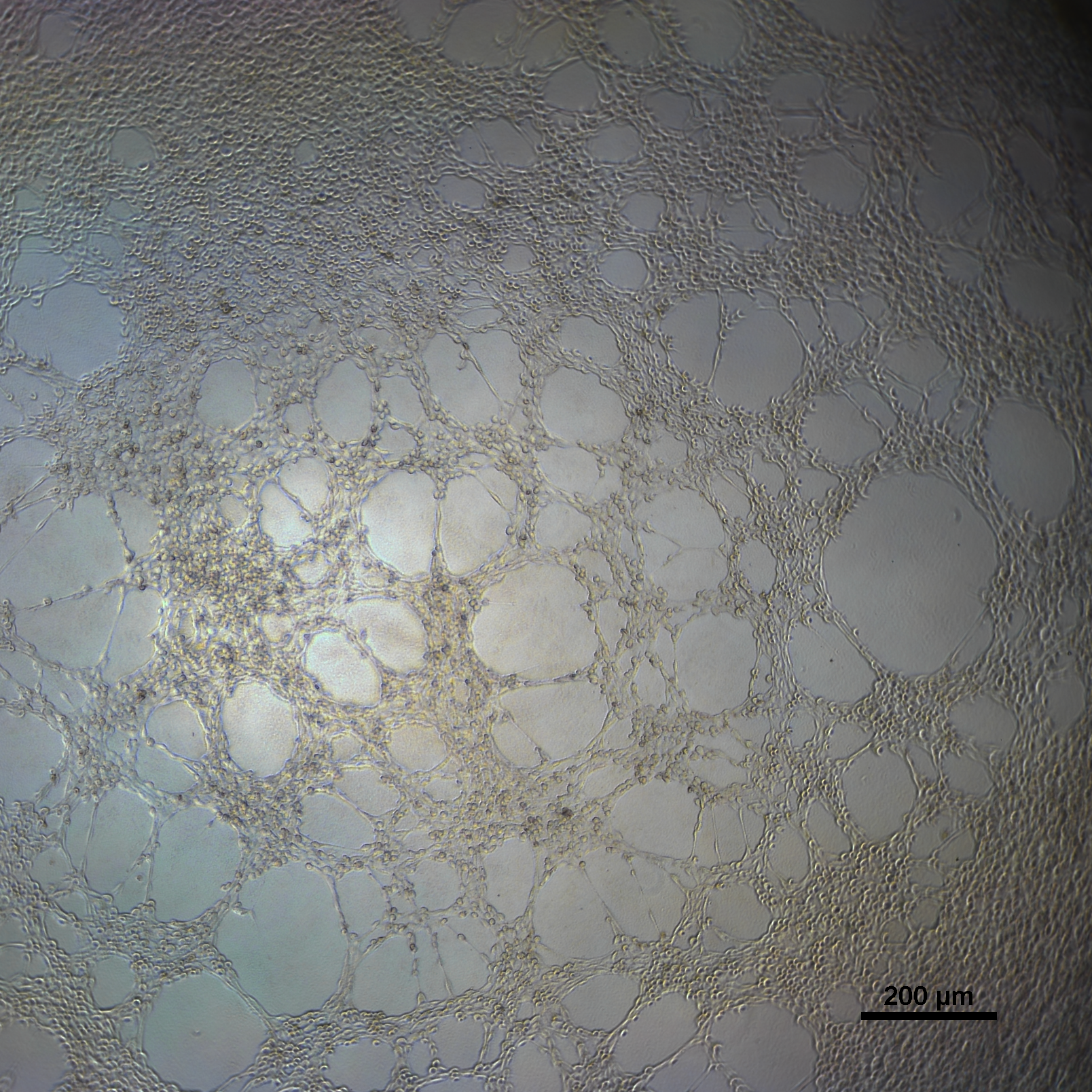

Supplement: Supplemental Information 51 [file peerj-10-13498-s051.tif]

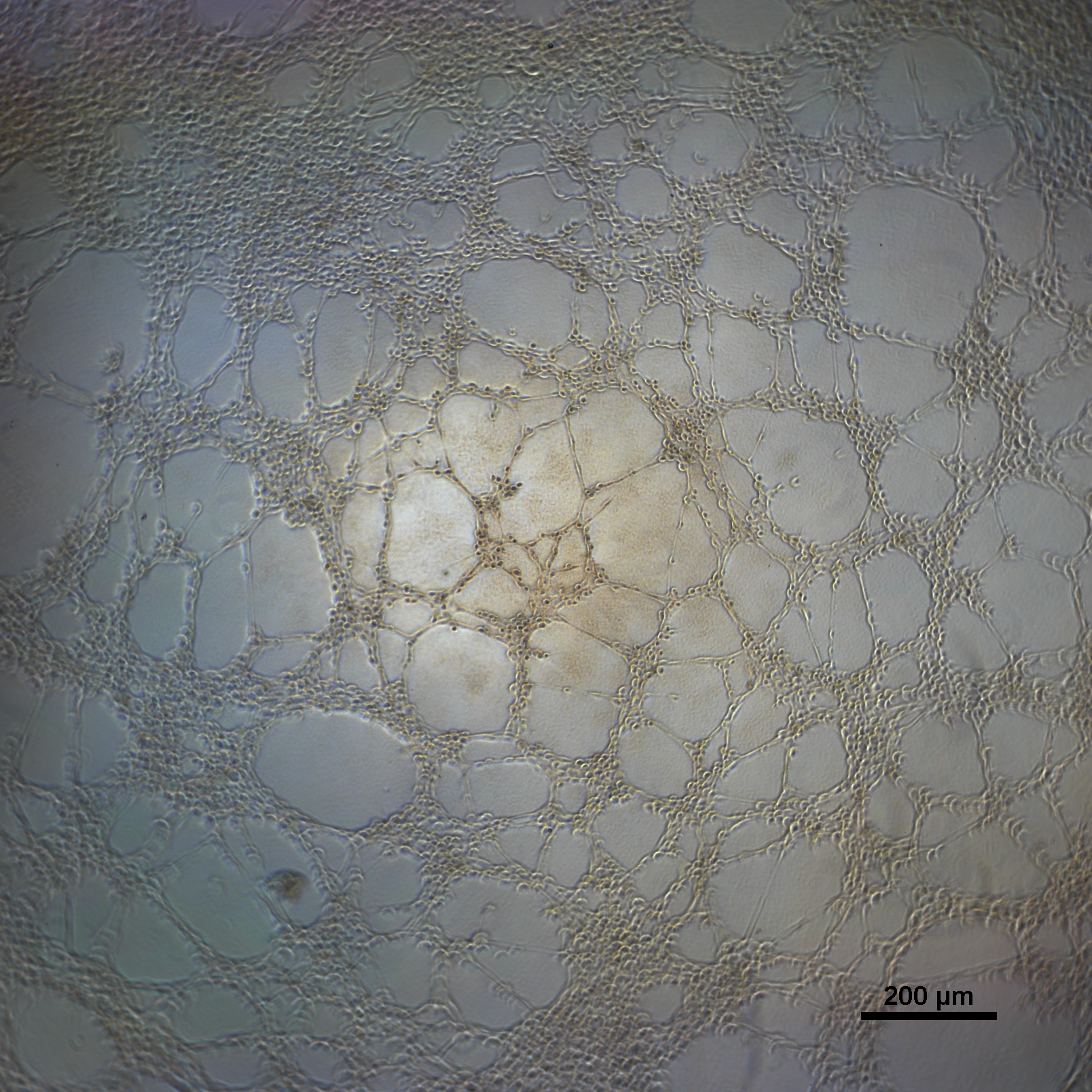

Supplement: Supplemental Information 52 [file peerj-10-13498-s052.tif]

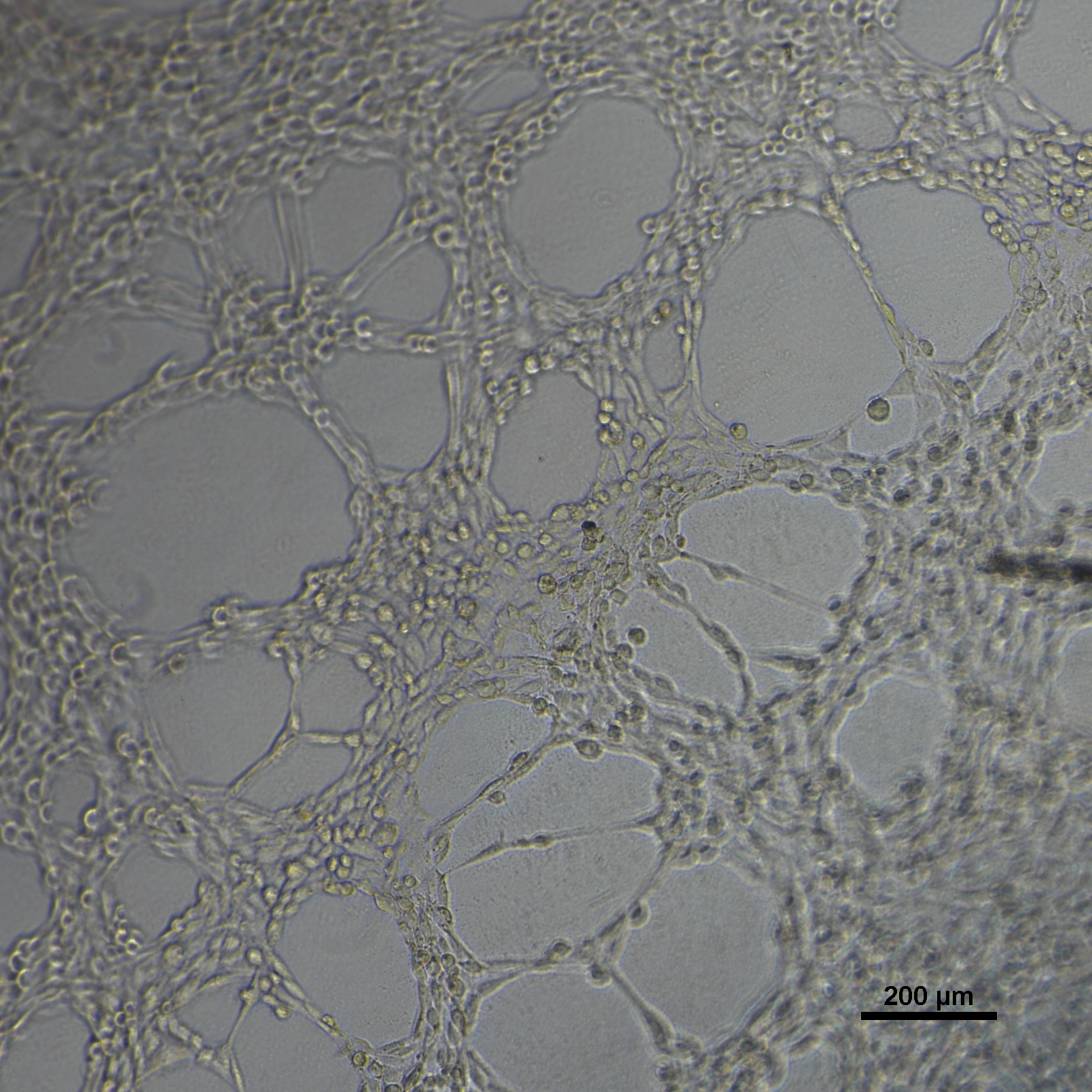

Supplement: Supplemental Information 53 [file peerj-10-13498-s053.tif]

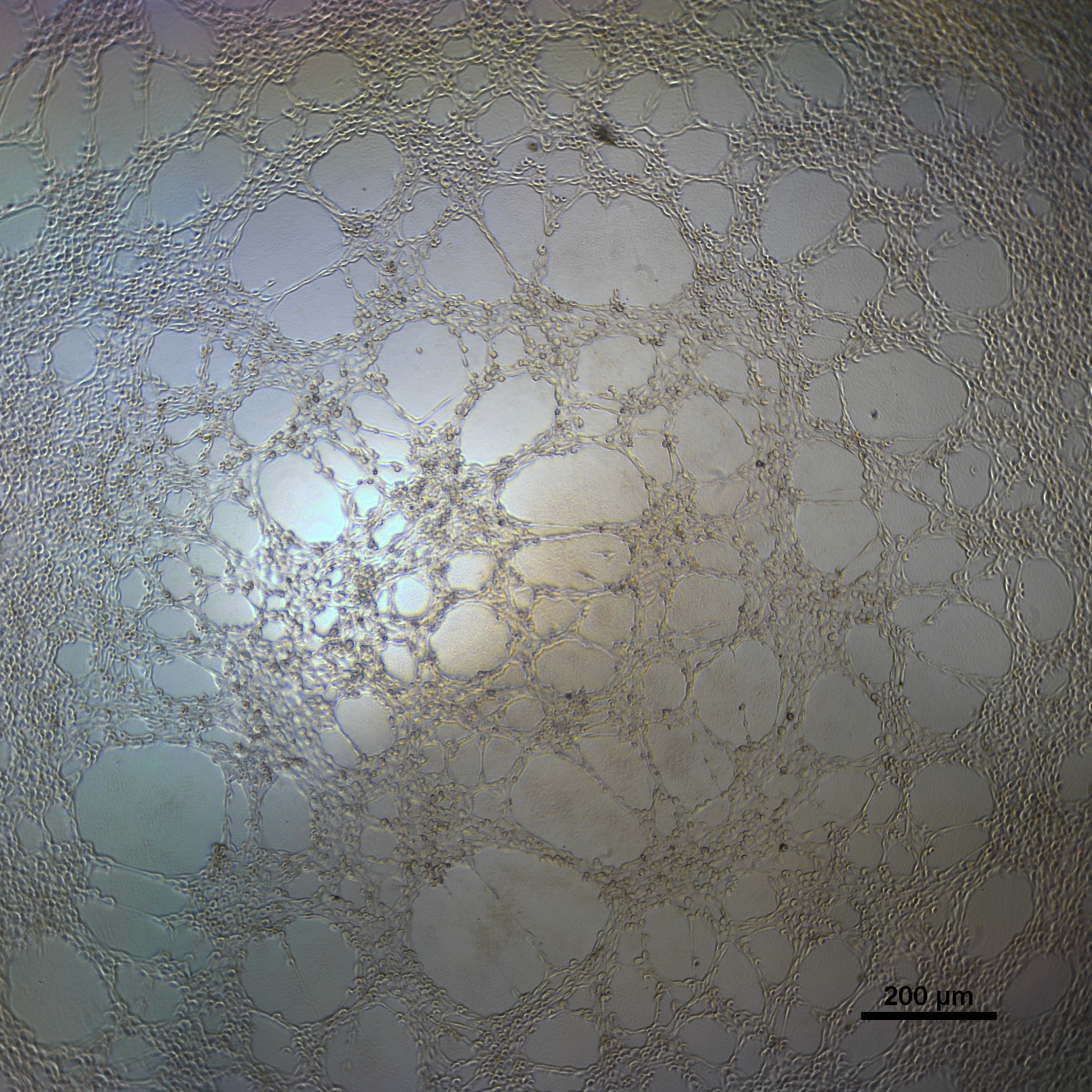

Supplement: Supplemental Information 54 [file peerj-10-13498-s054.tif]

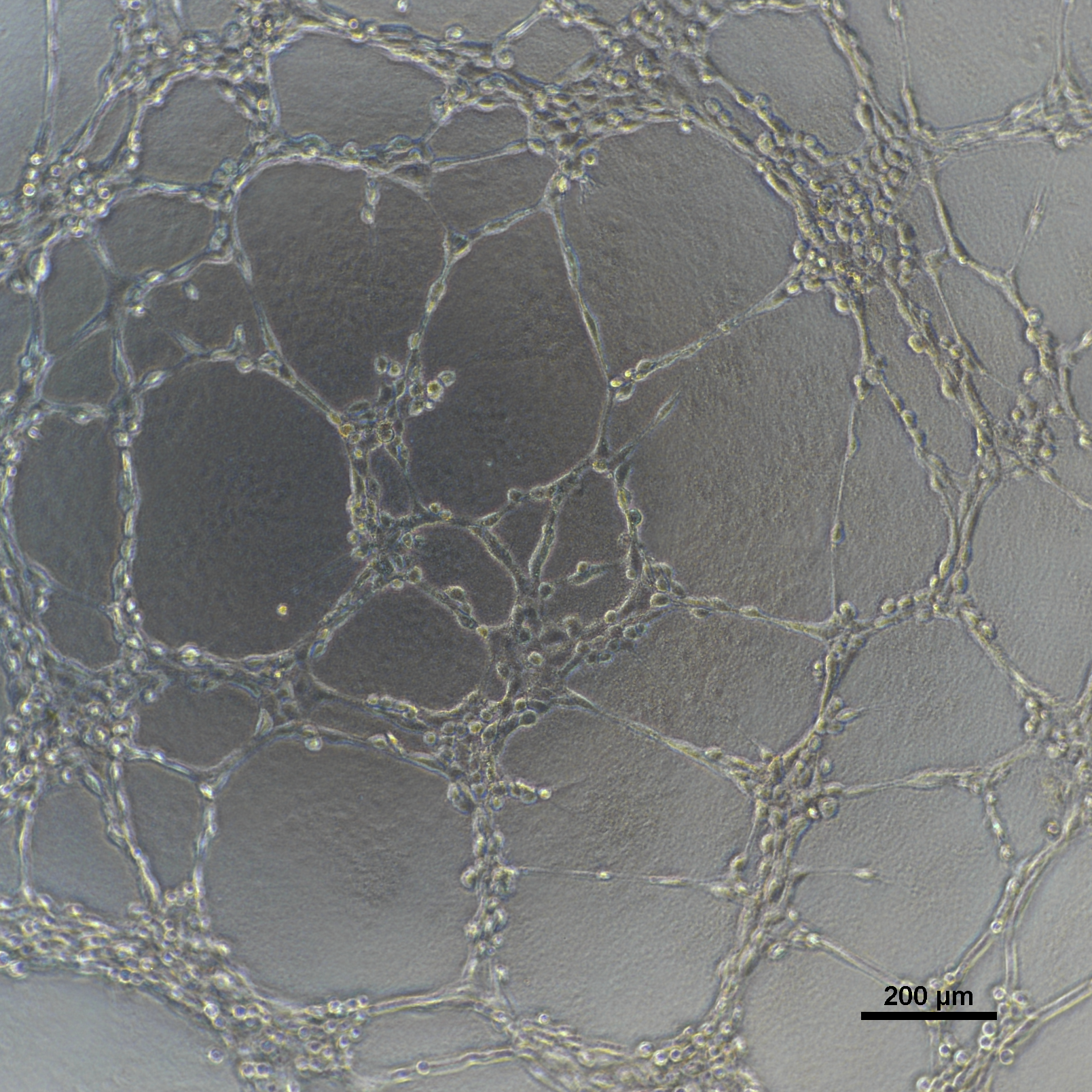

Supplement: Supplemental Information 55 [file peerj-10-13498-s055.tif]

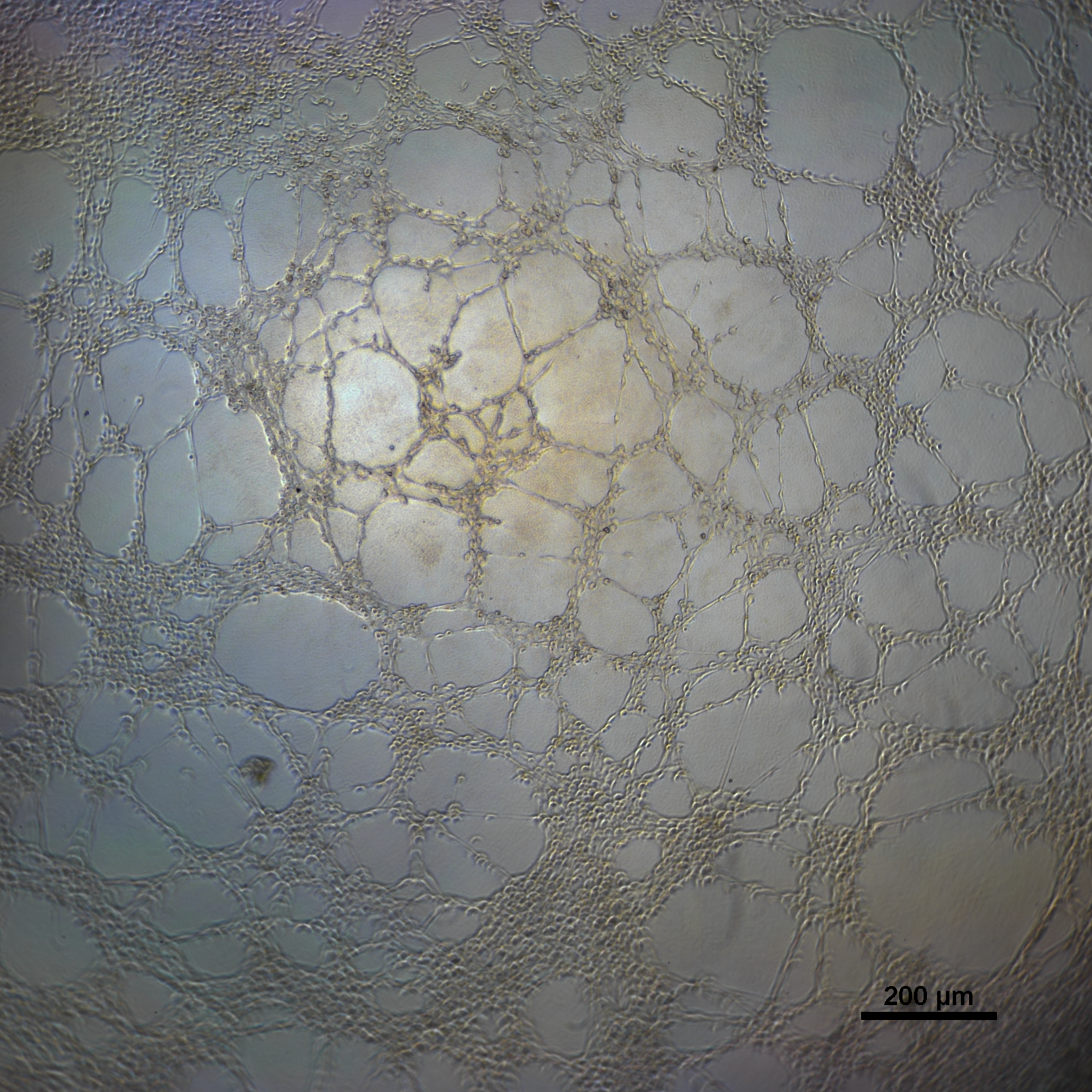

Supplement: Supplemental Information 56 [file peerj-10-13498-s056.tif]

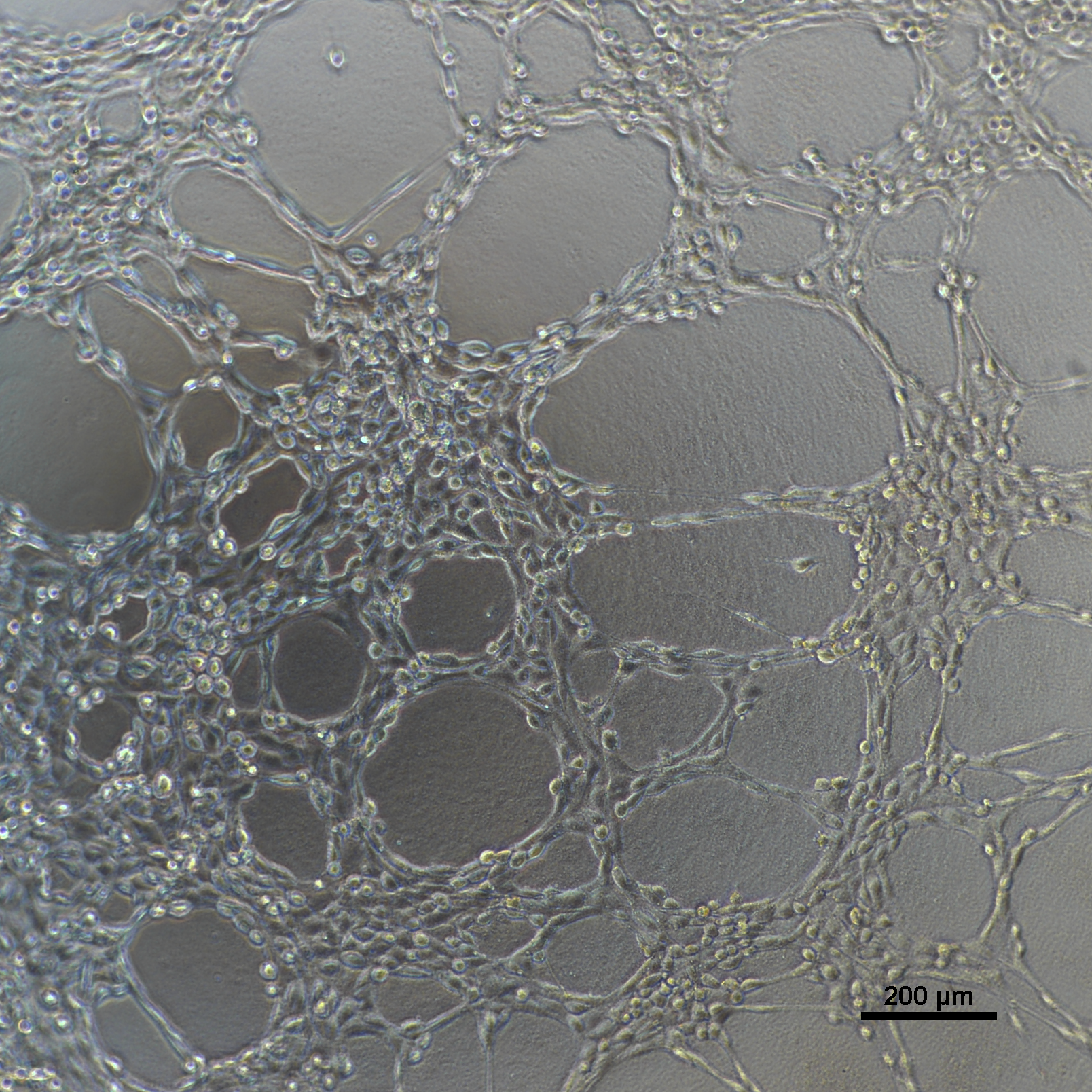

Supplement: Supplemental Information 57 [file peerj-10-13498-s057.tif]

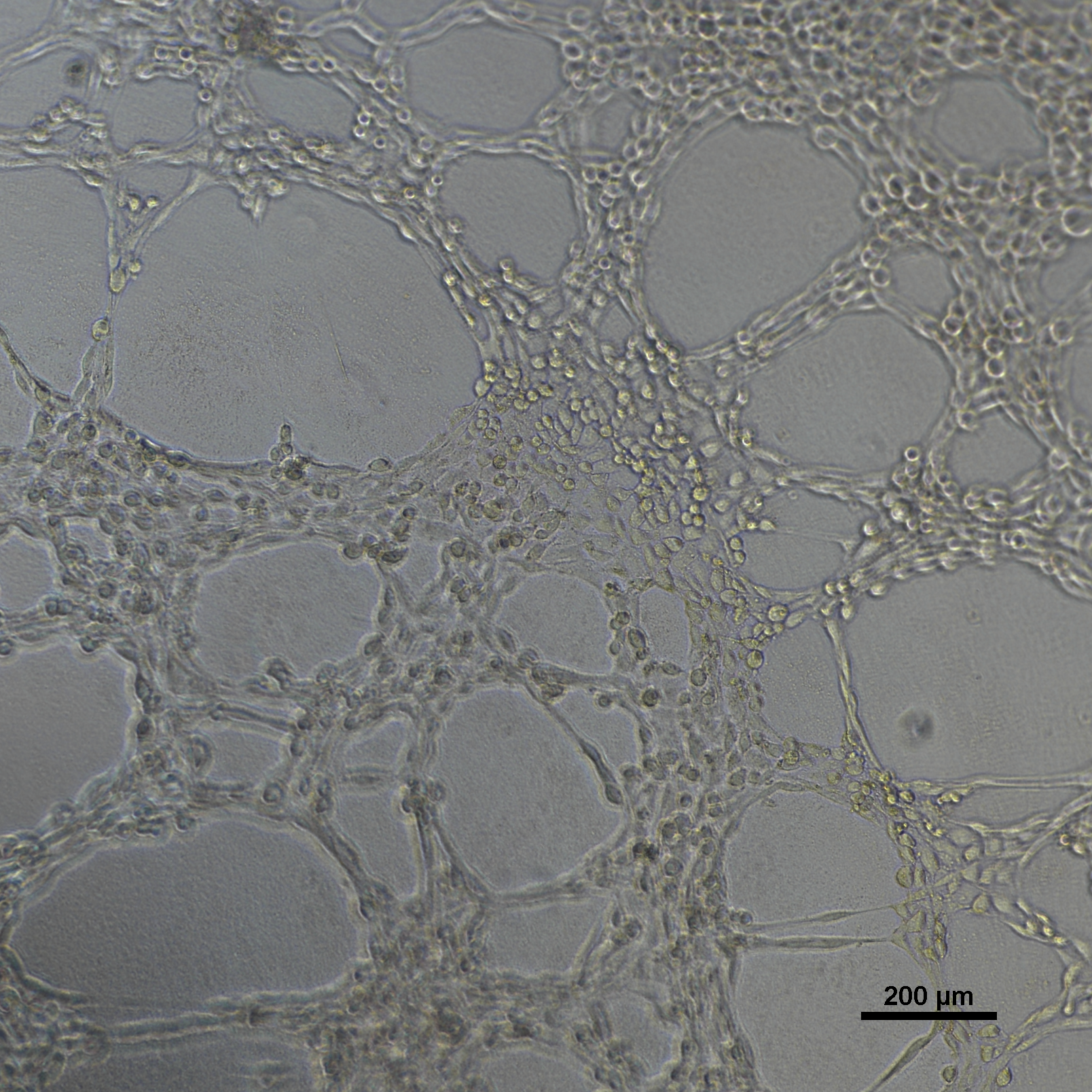

Supplement: Supplemental Information 58 [file peerj-10-13498-s058.tif]

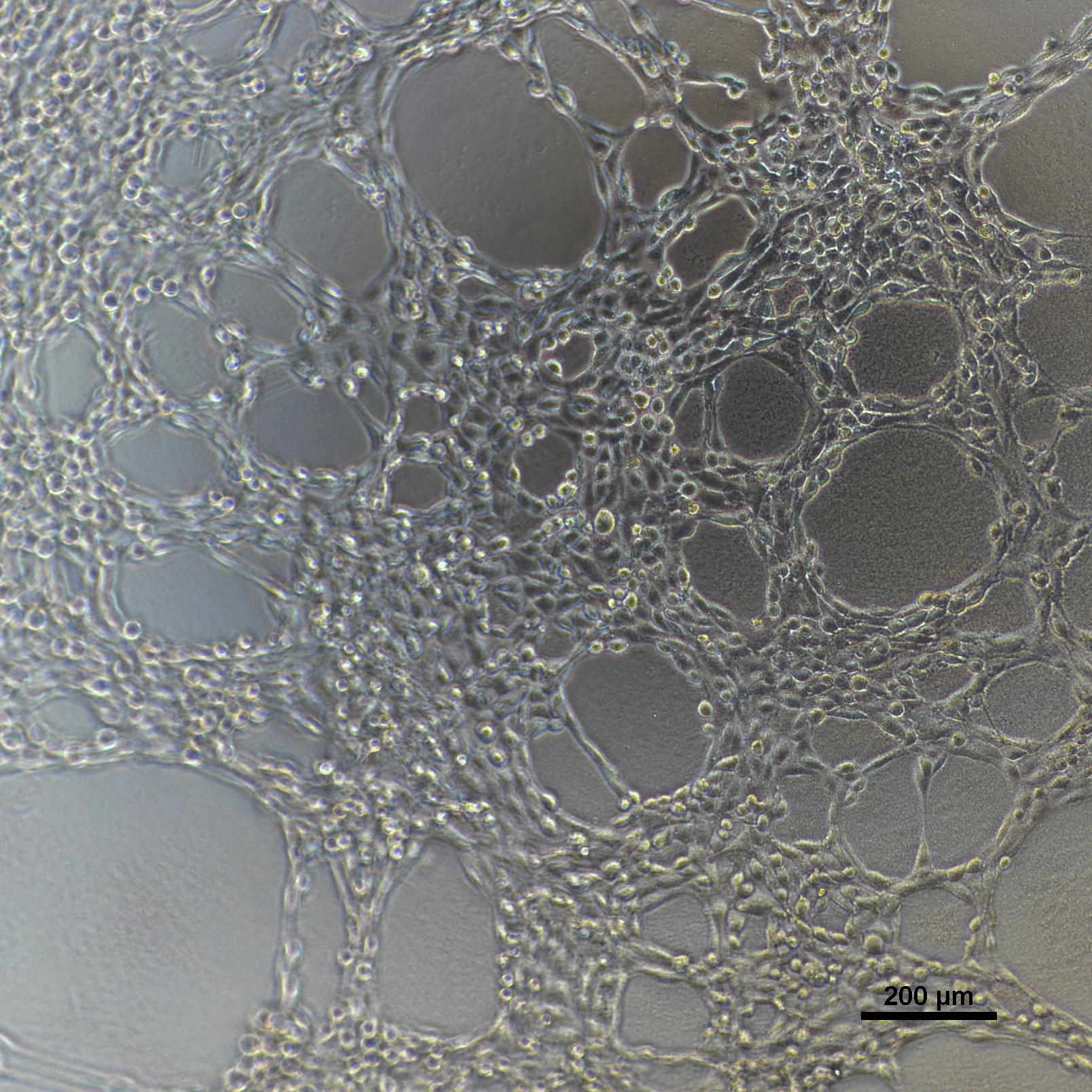

Supplement: Supplemental Information 59 [file peerj-10-13498-s059.tif]

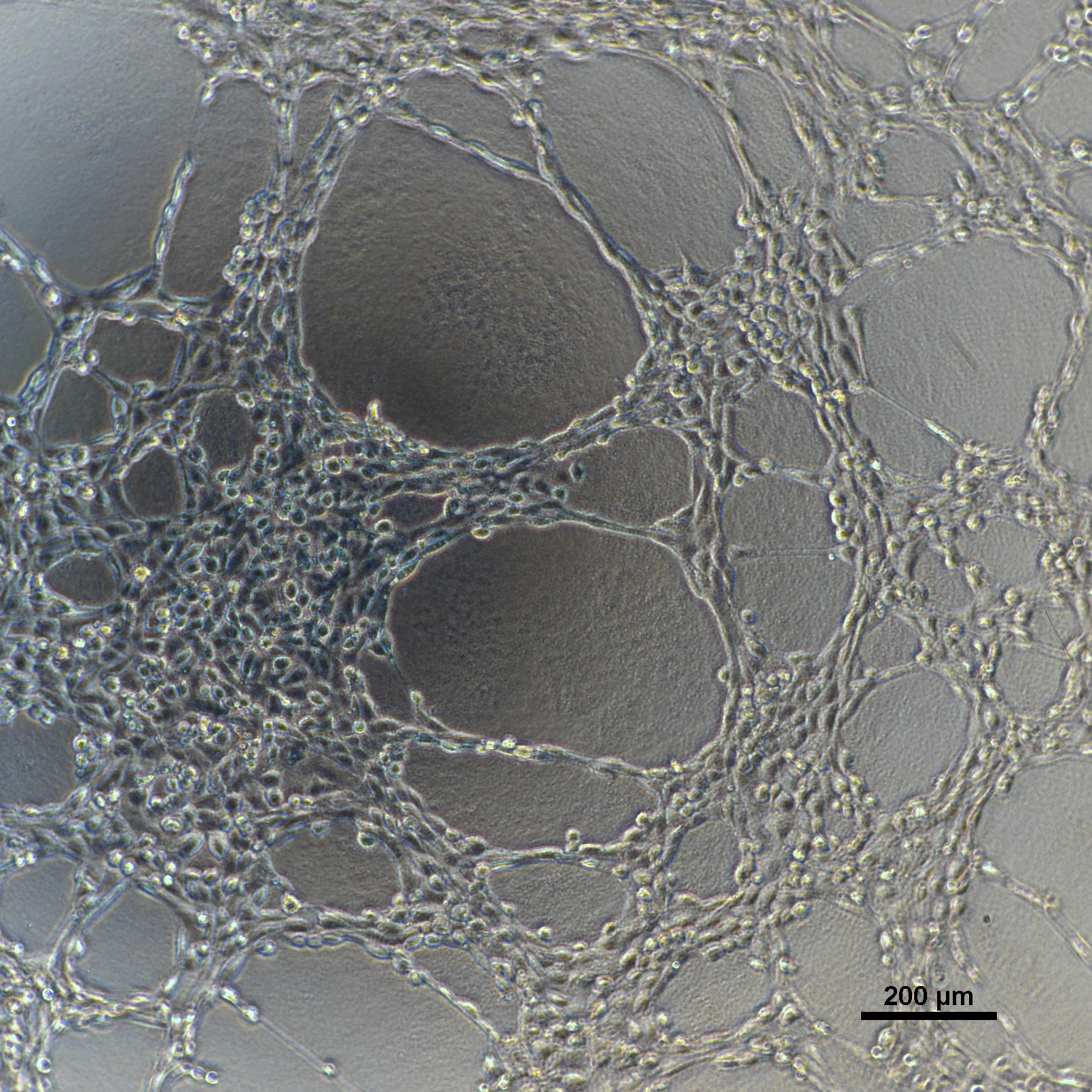

Supplement: Supplemental Information 61 [file peerj-10-13498-s061.tif]

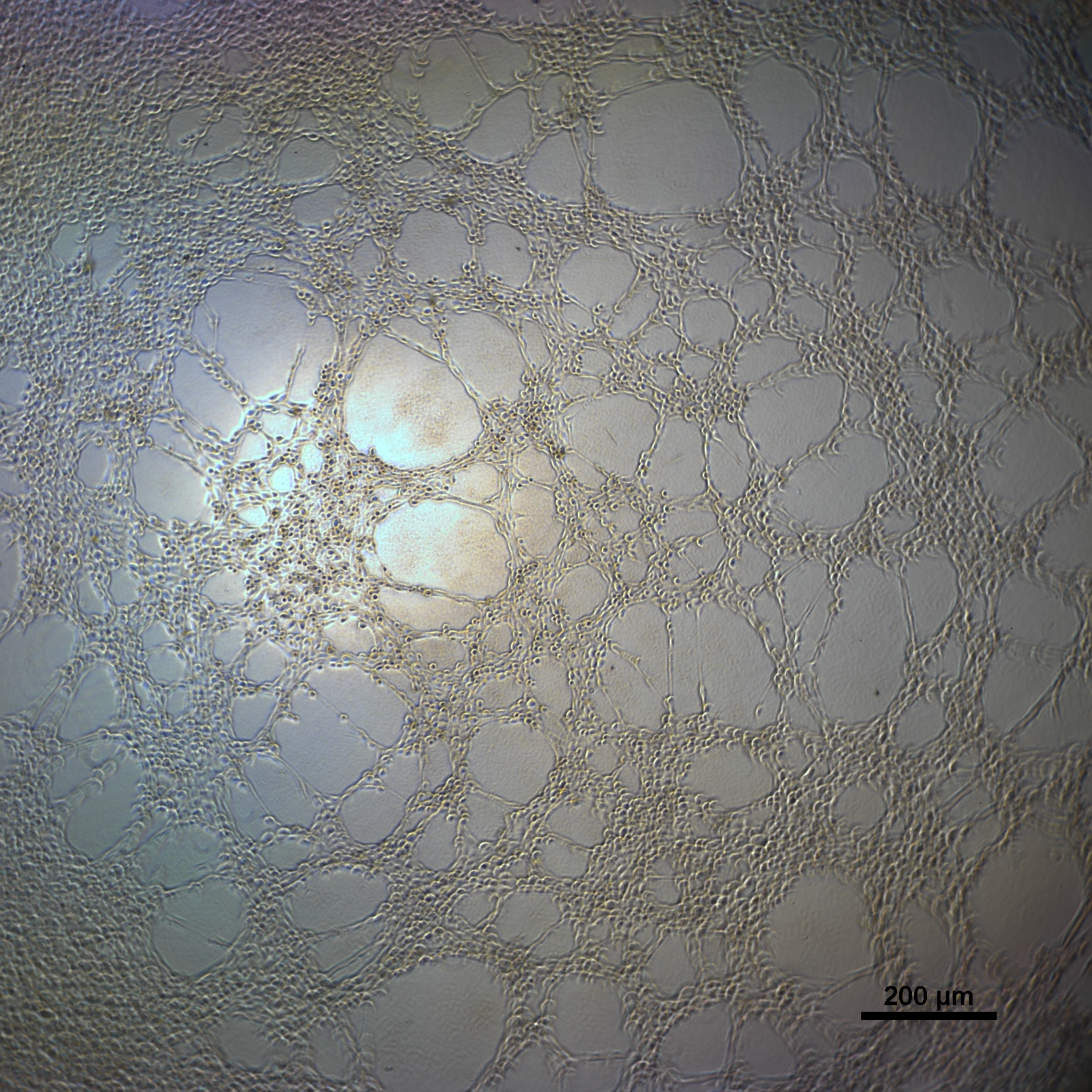

Supplement: Supplemental Information 62 [file peerj-10-13498-s062.tif]

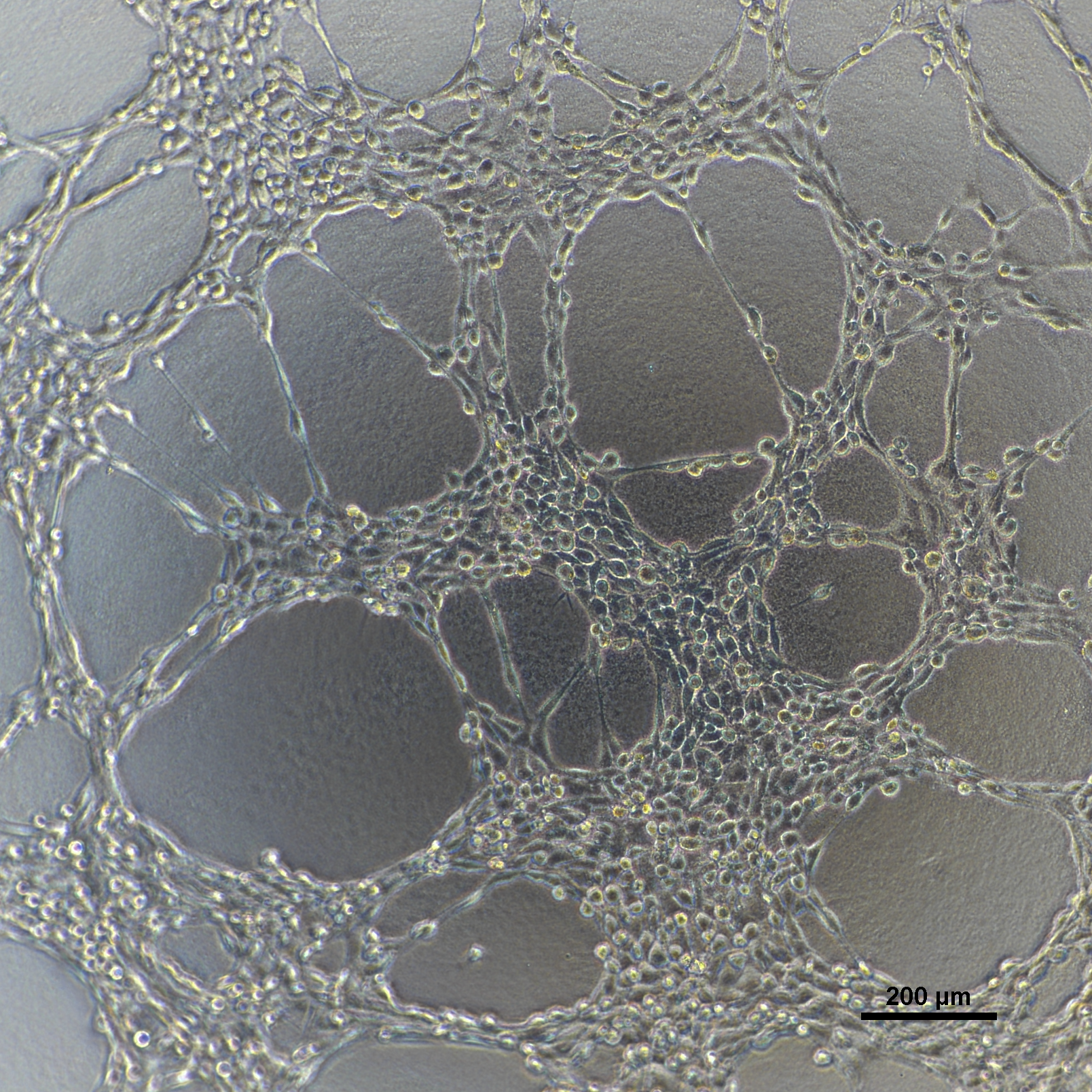

Supplement: Supplemental Information 63 [file peerj-10-13498-s063.tif]

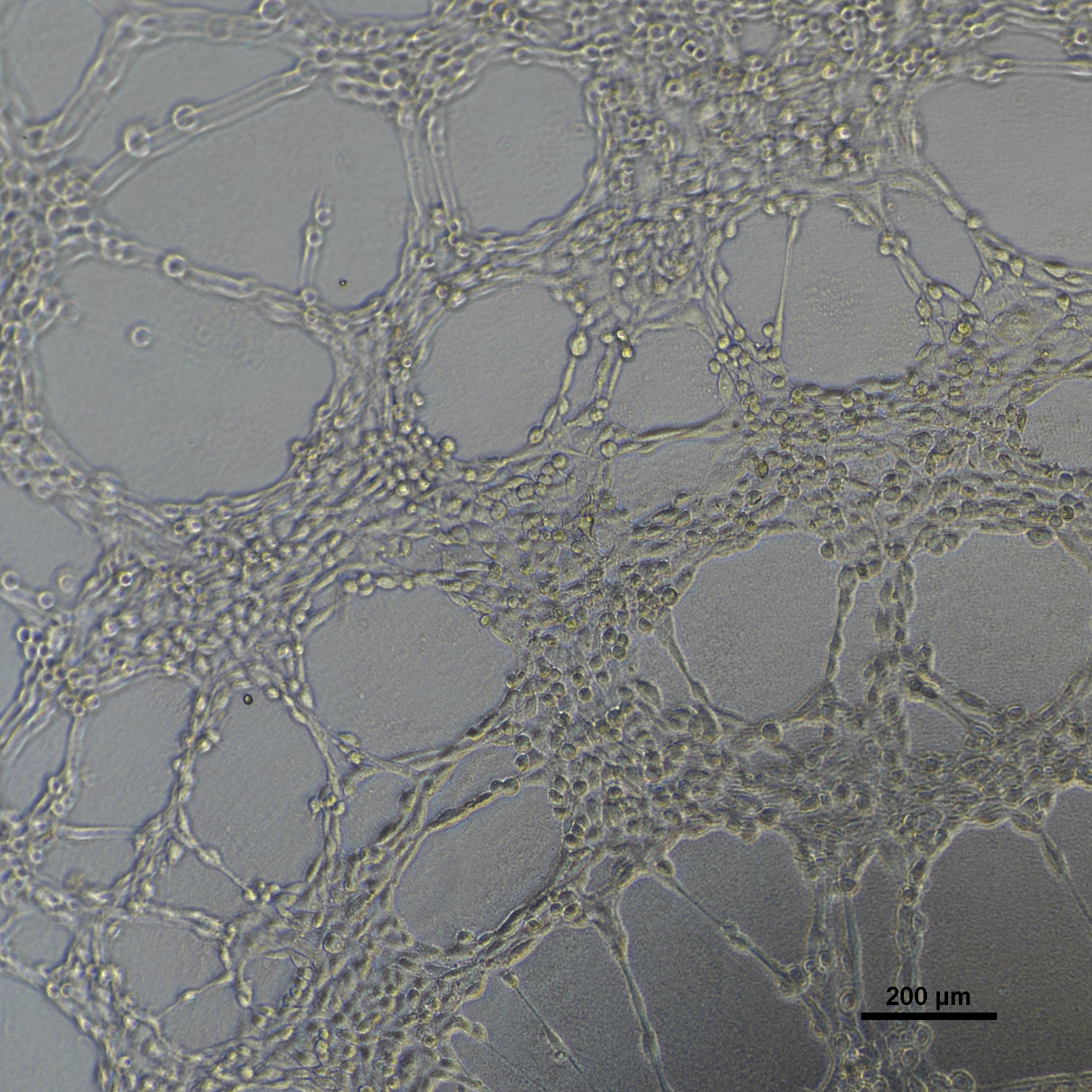

Supplement: Supplemental Information 64 [file peerj-10-13498-s064.tif]

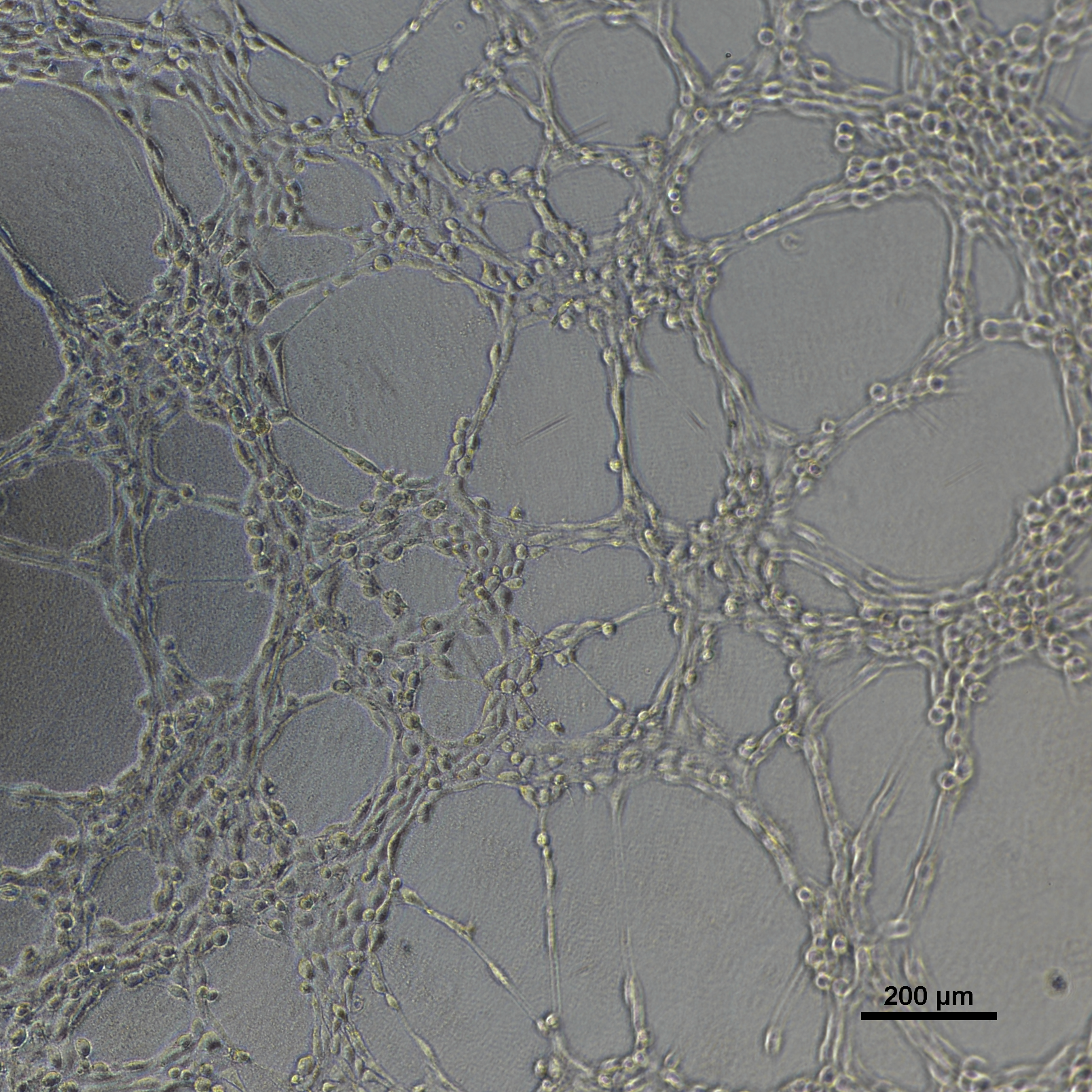

Supplement: Supplemental Information 65 [file peerj-10-13498-s065.tif]

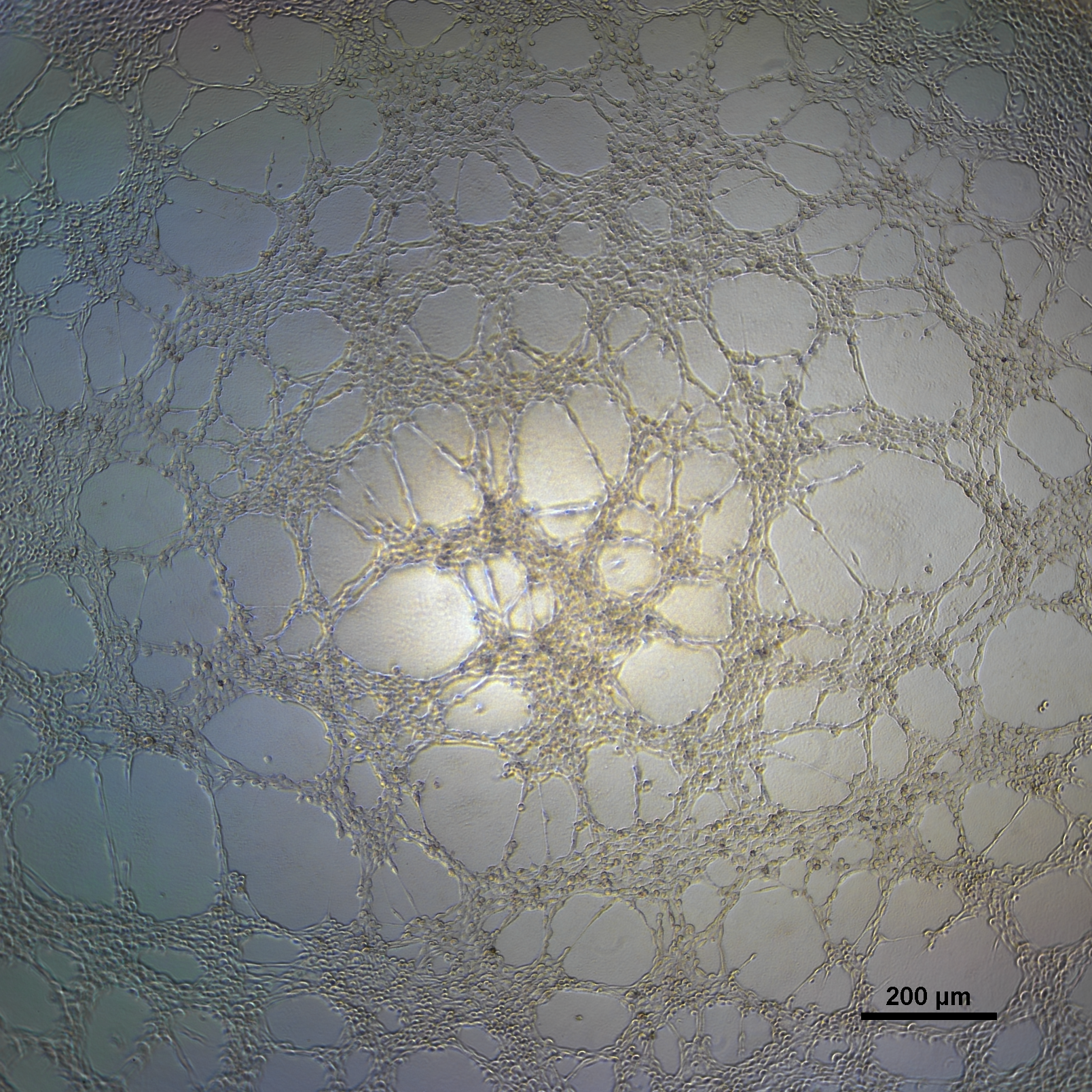

Supplement: Supplemental Information 66 [file peerj-10-13498-s066.tif]

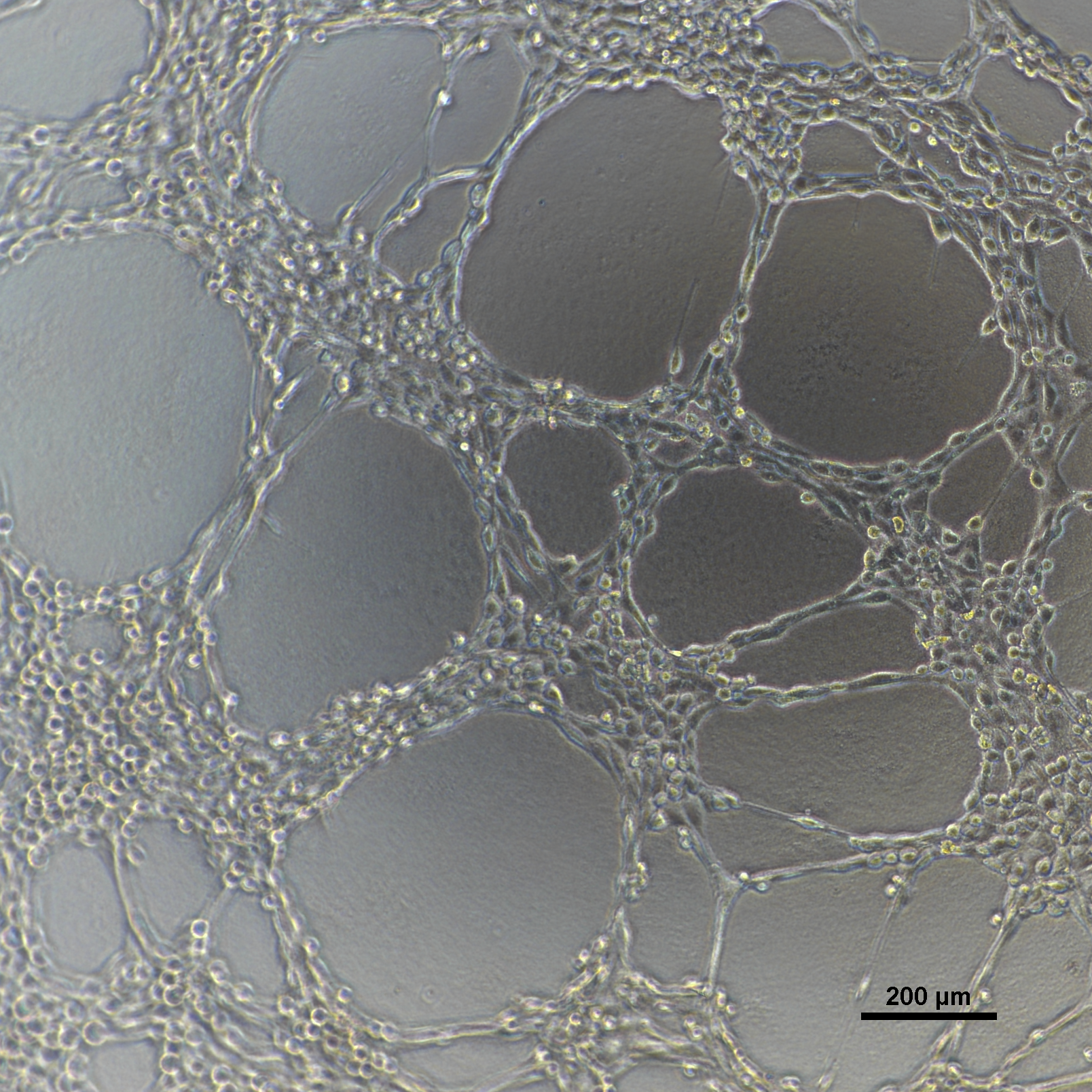

Supplement: Supplemental Information 67 [file peerj-10-13498-s067.tif]

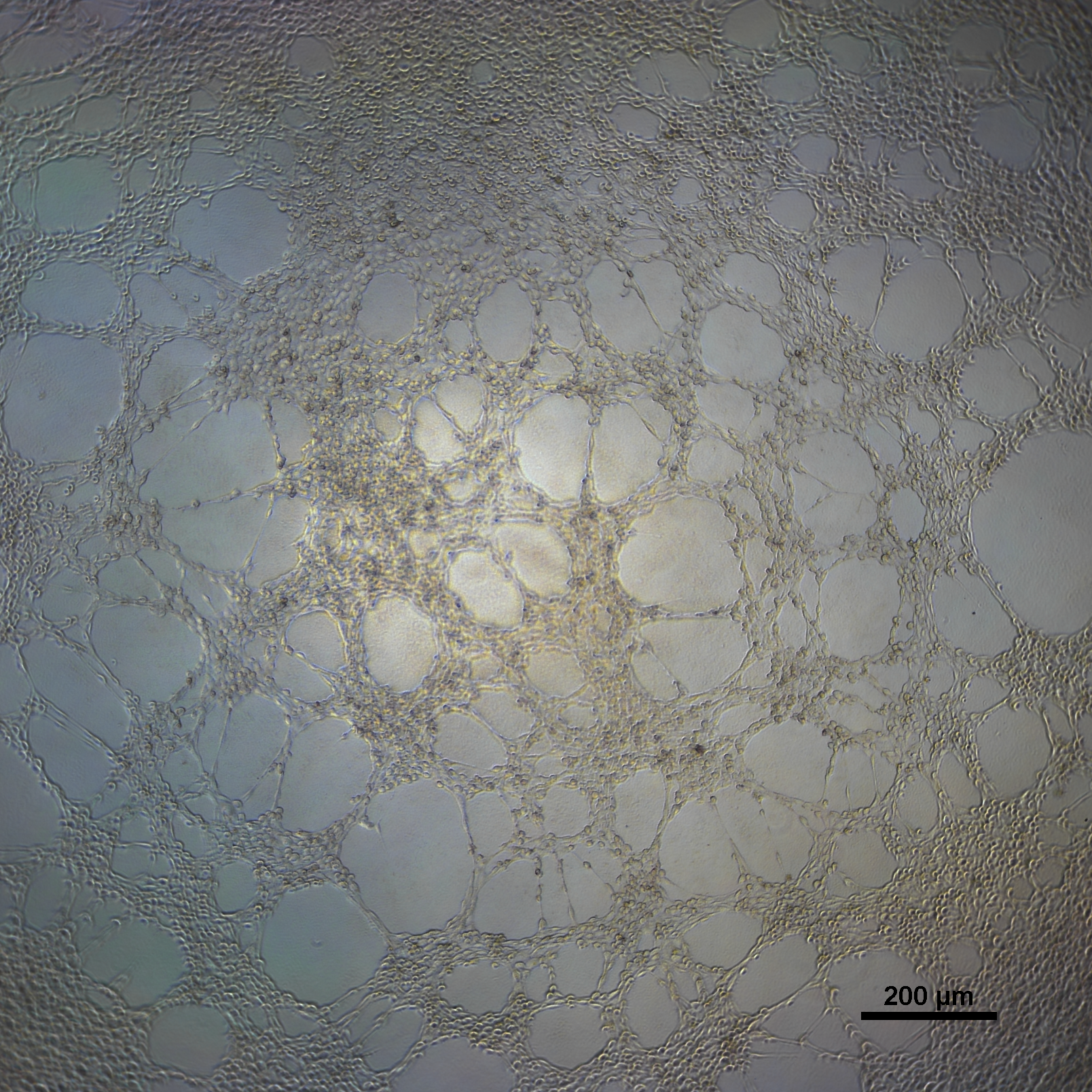

Supplement: Supplemental Information 68 [file peerj-10-13498-s068.tif]

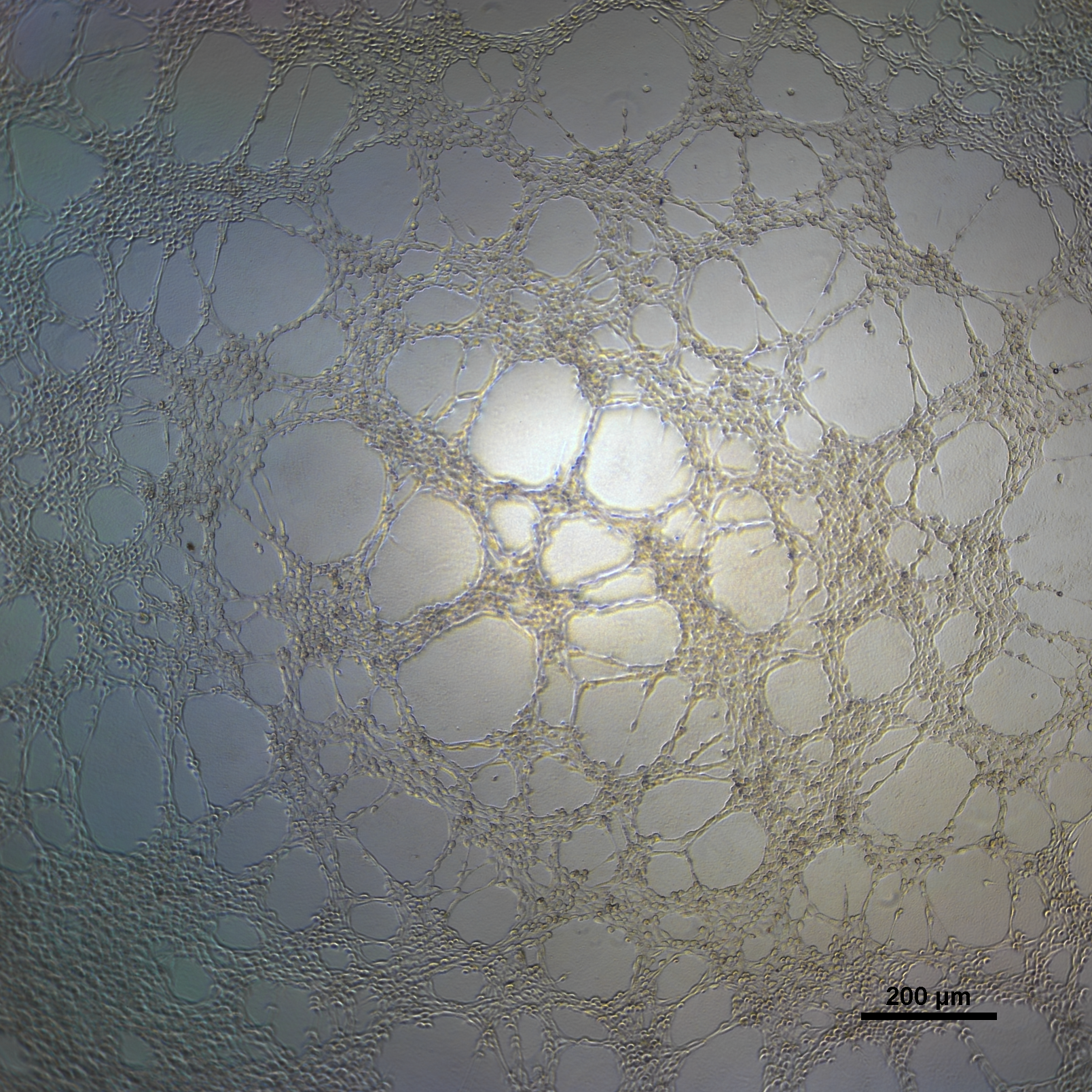

Supplement: Supplemental Information 69 [file peerj-10-13498-s069.tif]

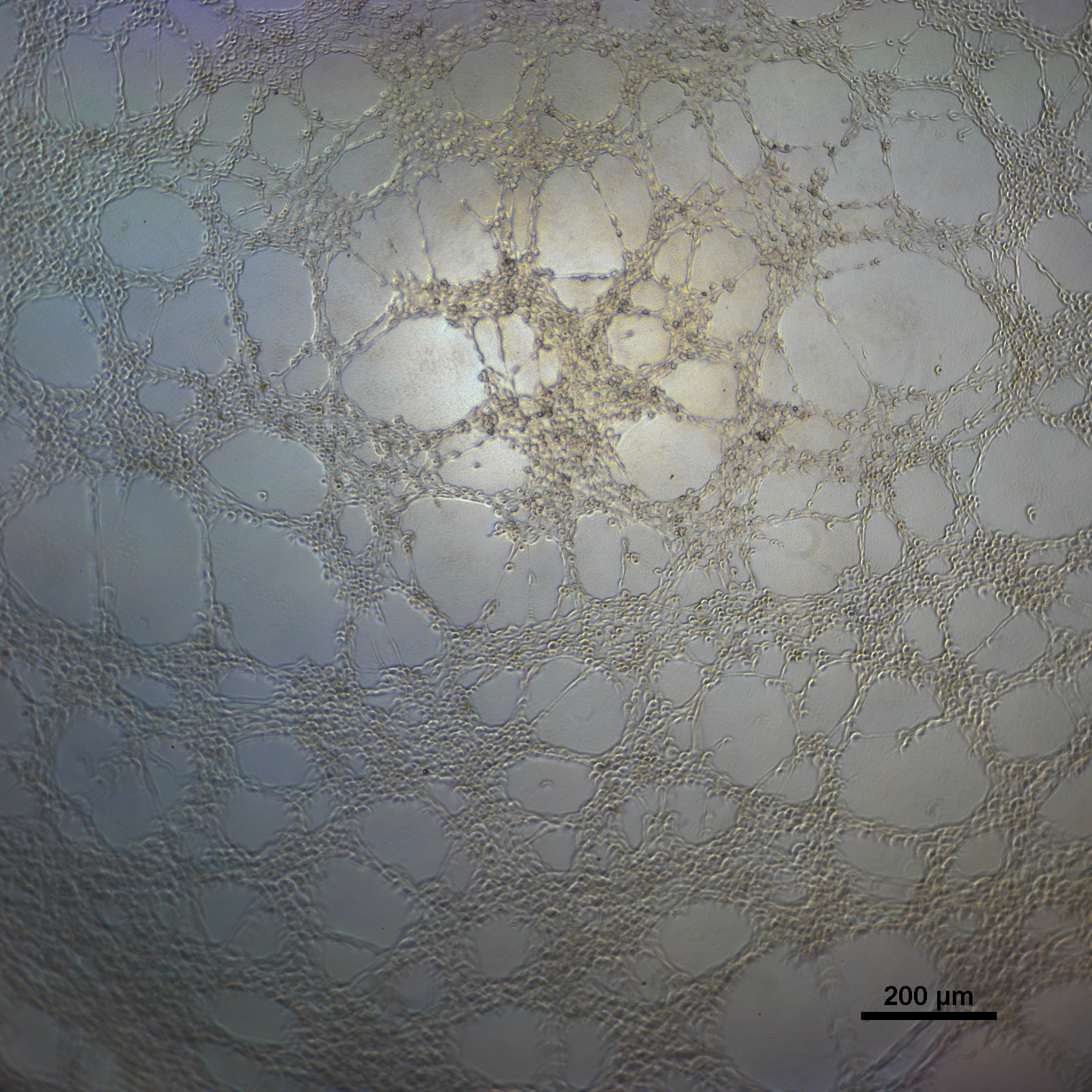

Supplement: Supplemental Information 70 [file peerj-10-13498-s070.tif]

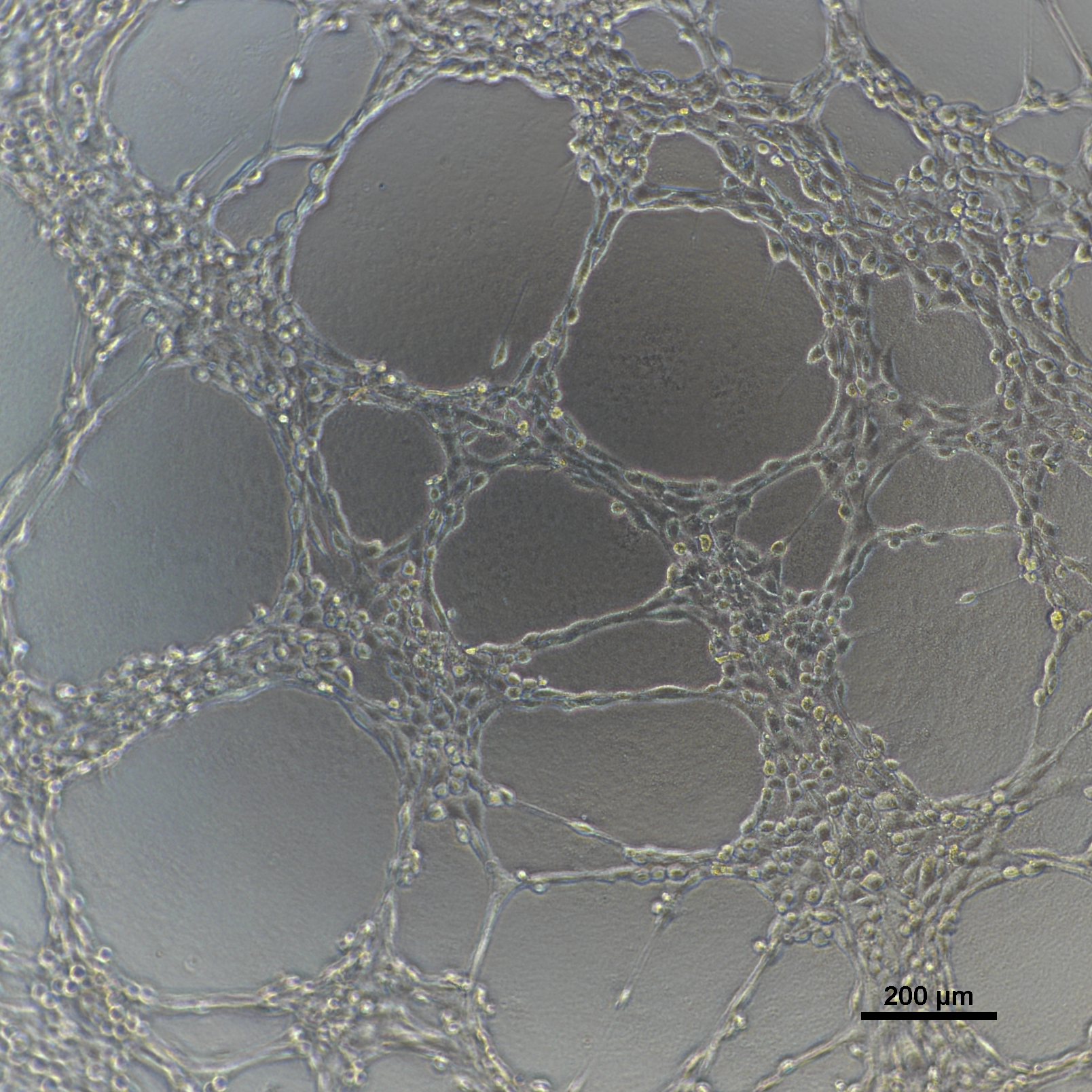

Supplement: Supplemental Information 71 [file peerj-10-13498-s071.tif]

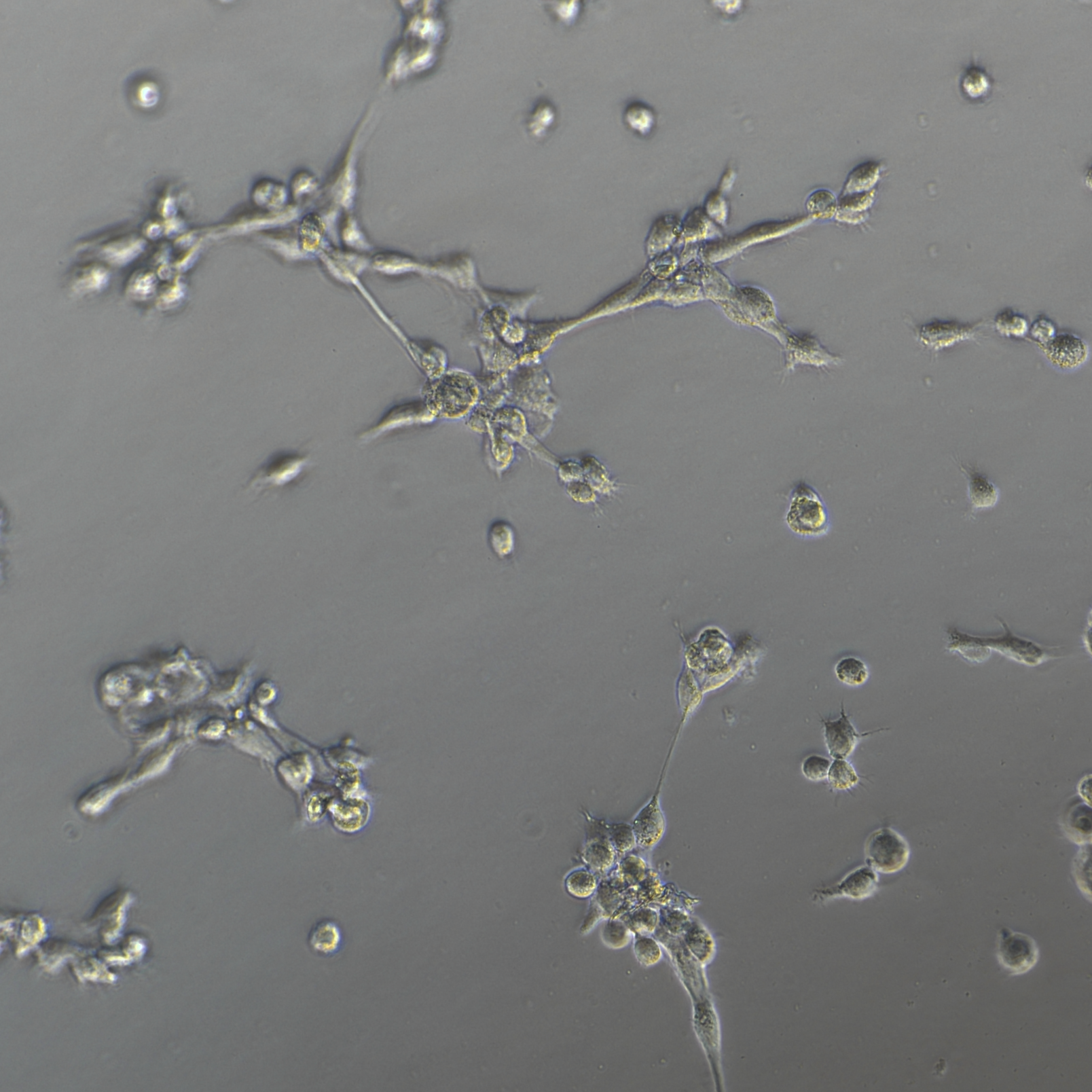

Supplement: Supplemental Information 72 [file peerj-10-13498-s072.tif]

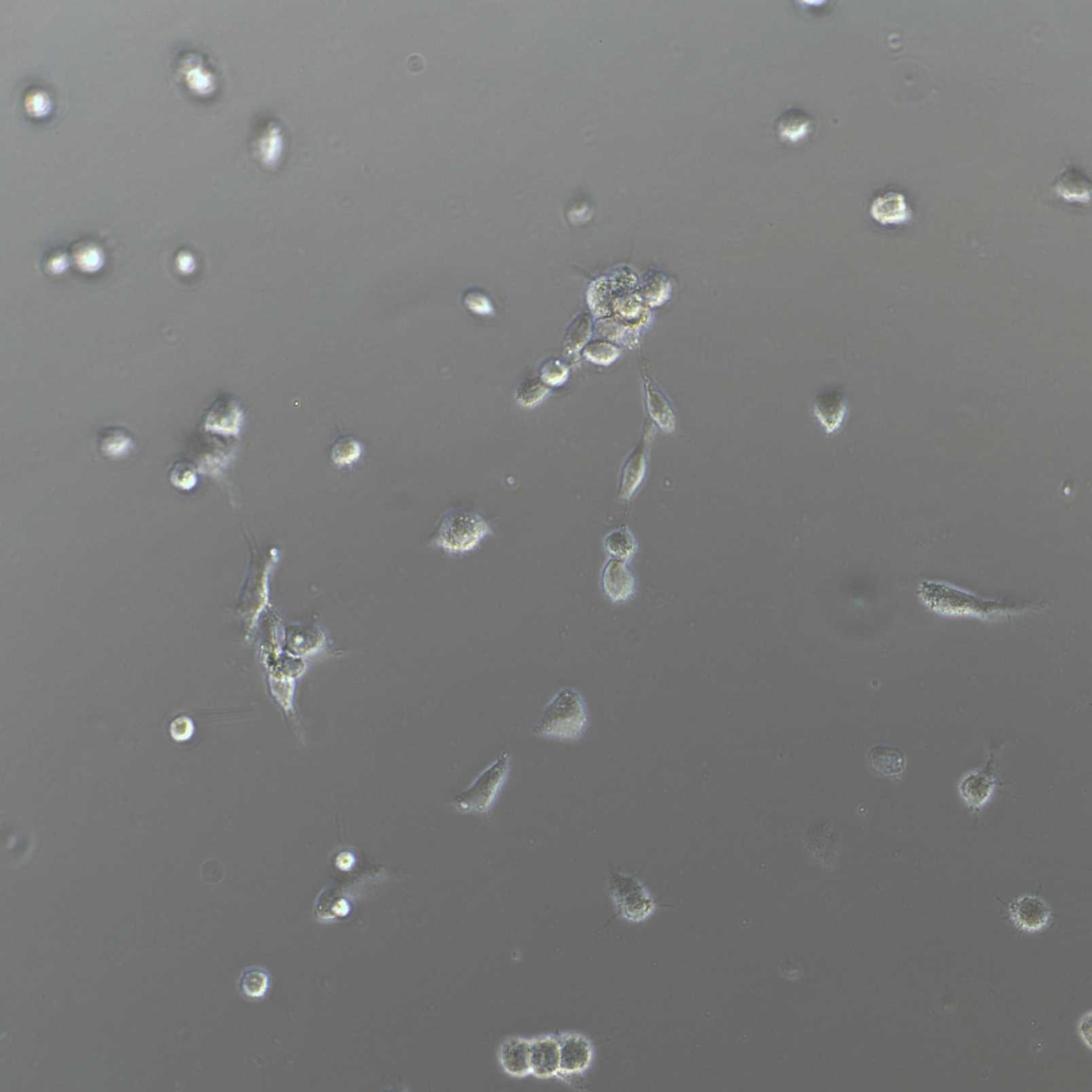

Supplement: Supplemental Information 73 [file peerj-10-13498-s073.tif]

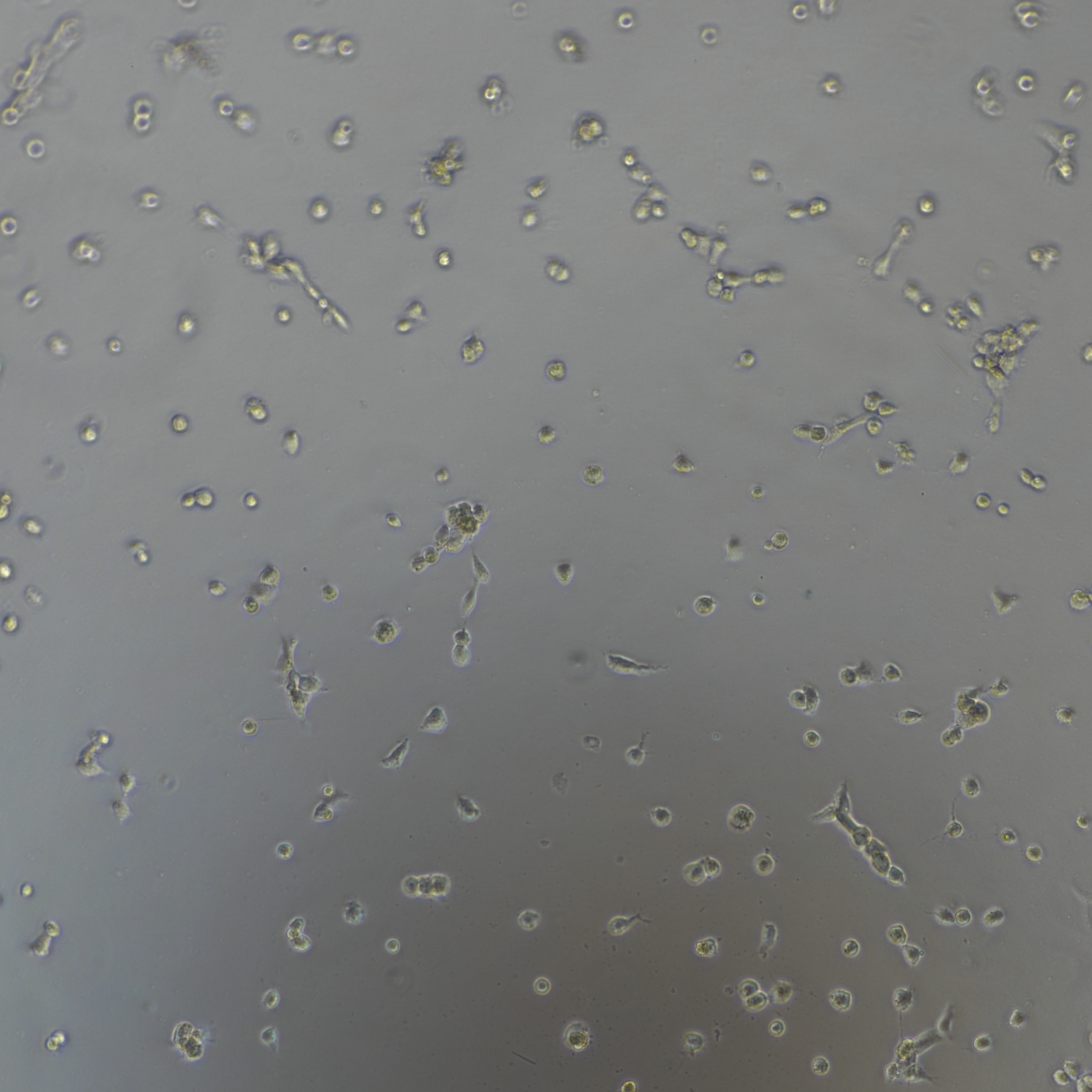

Supplement: Supplemental Information 74 [file peerj-10-13498-s074.tif]

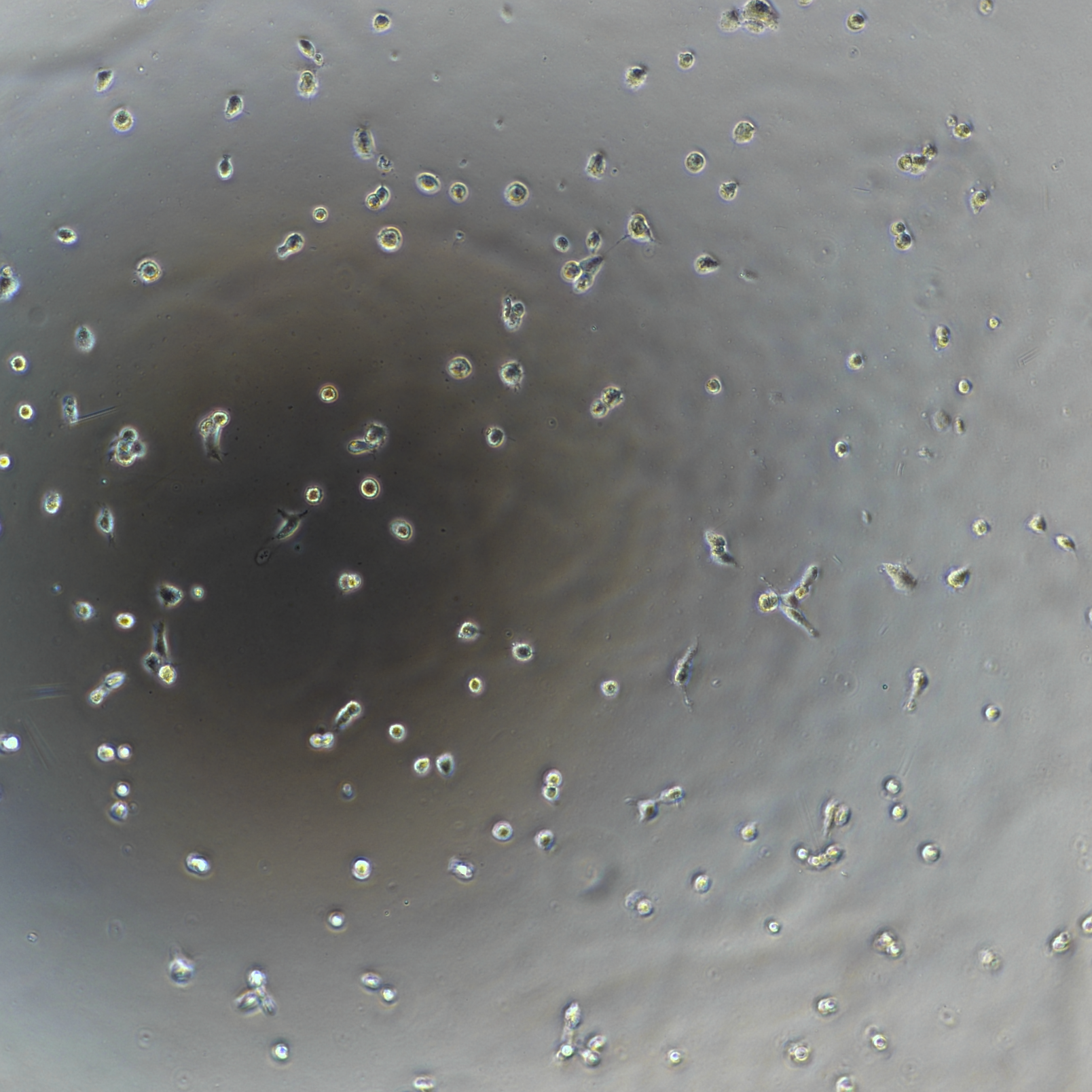

Supplement: Supplemental Information 75 [file peerj-10-13498-s075.tif]

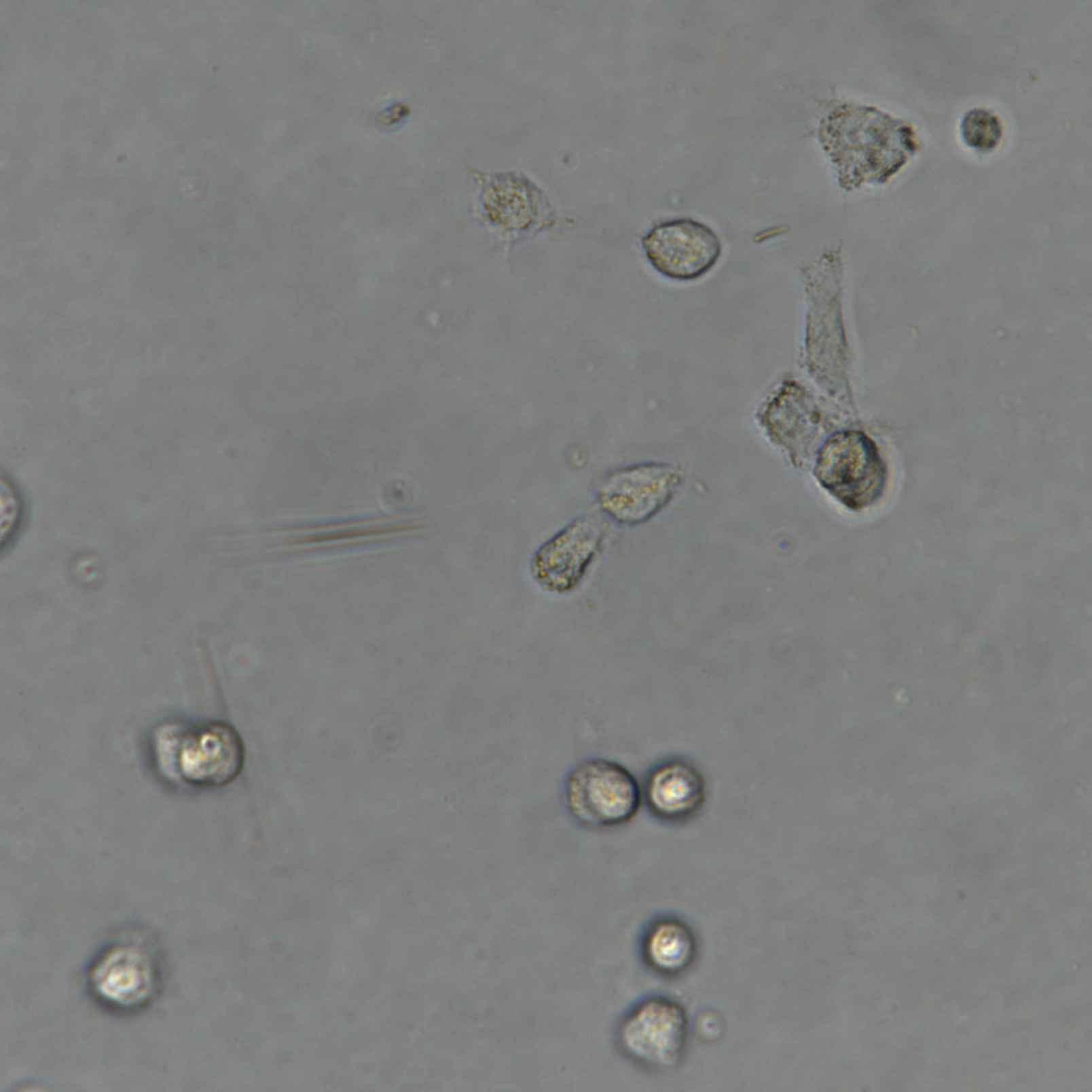

Supplement: Supplemental Information 76 [file peerj-10-13498-s076.tif]

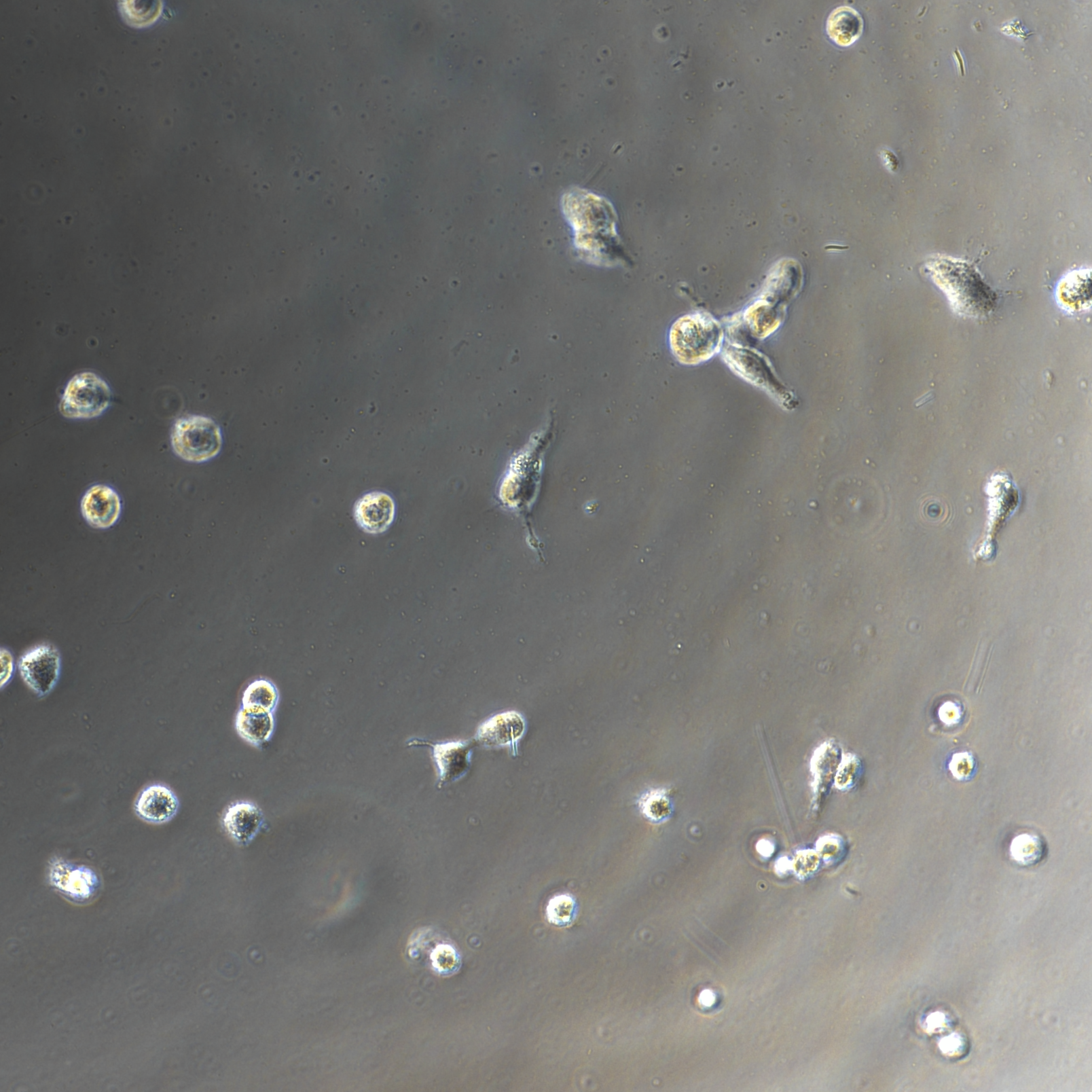

Supplement: Supplemental Information 77 [file peerj-10-13498-s077.tif]

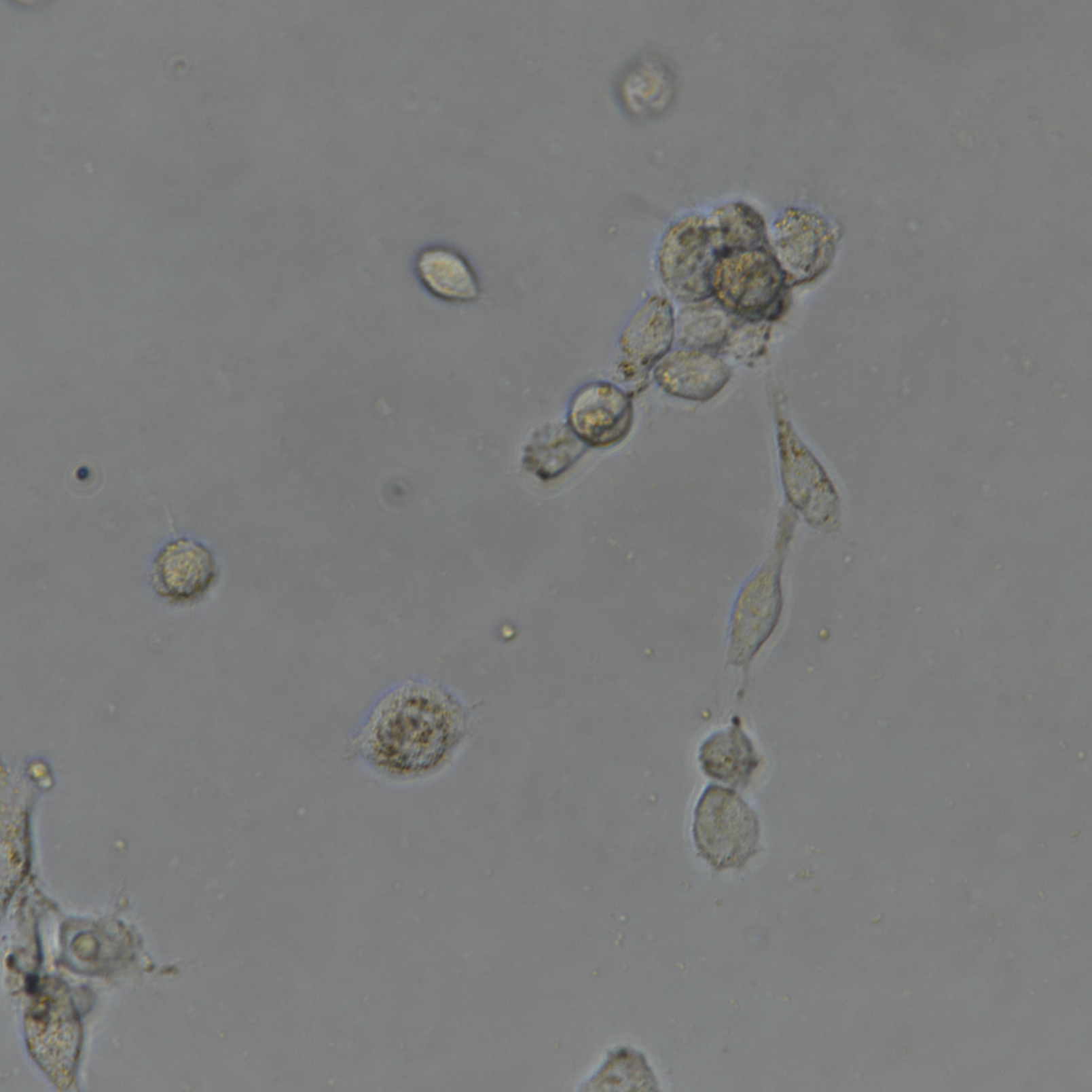

Supplement: Supplemental Information 78 [file peerj-10-13498-s078.tif]

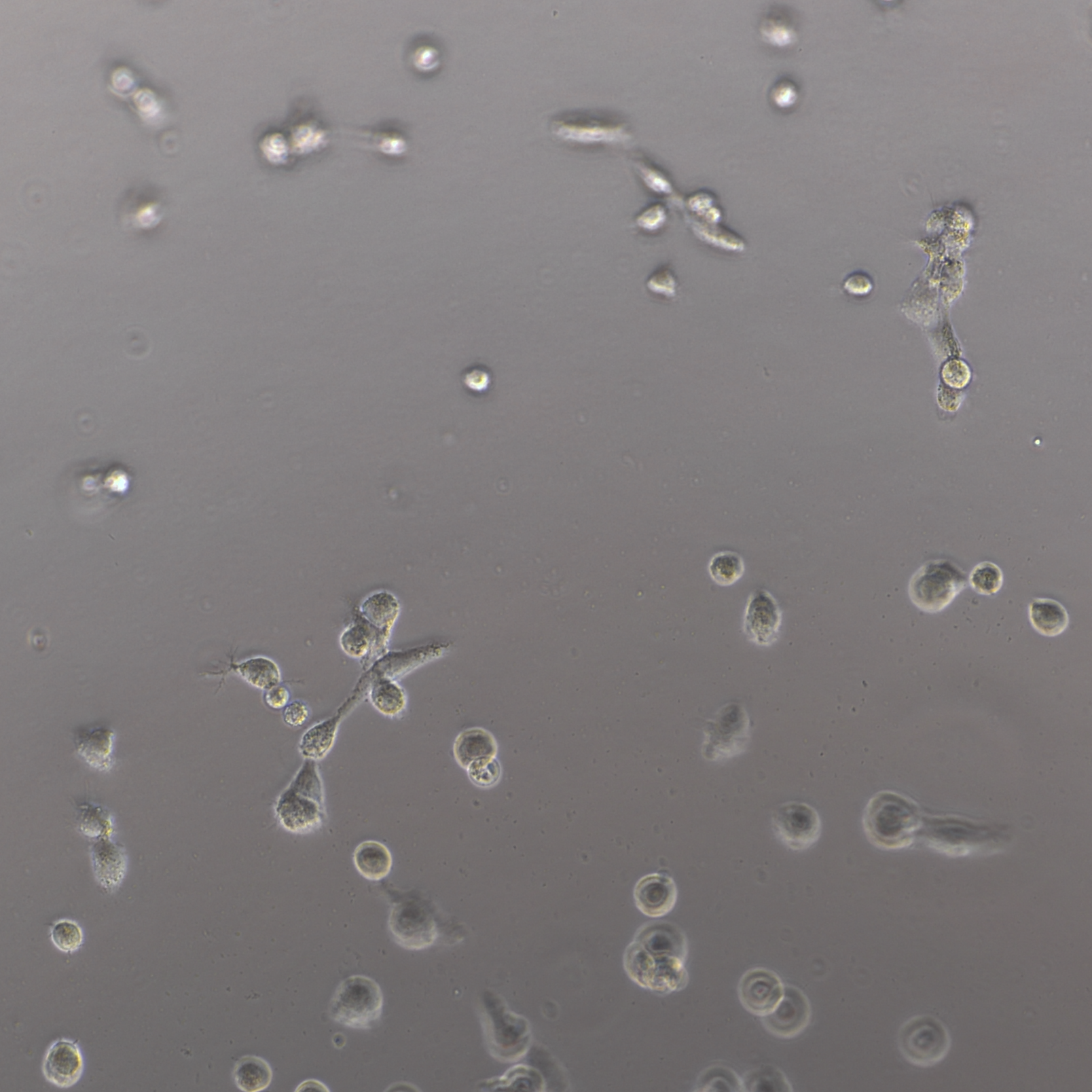

Supplement: Supplemental Information 79 [file peerj-10-13498-s079.tif]

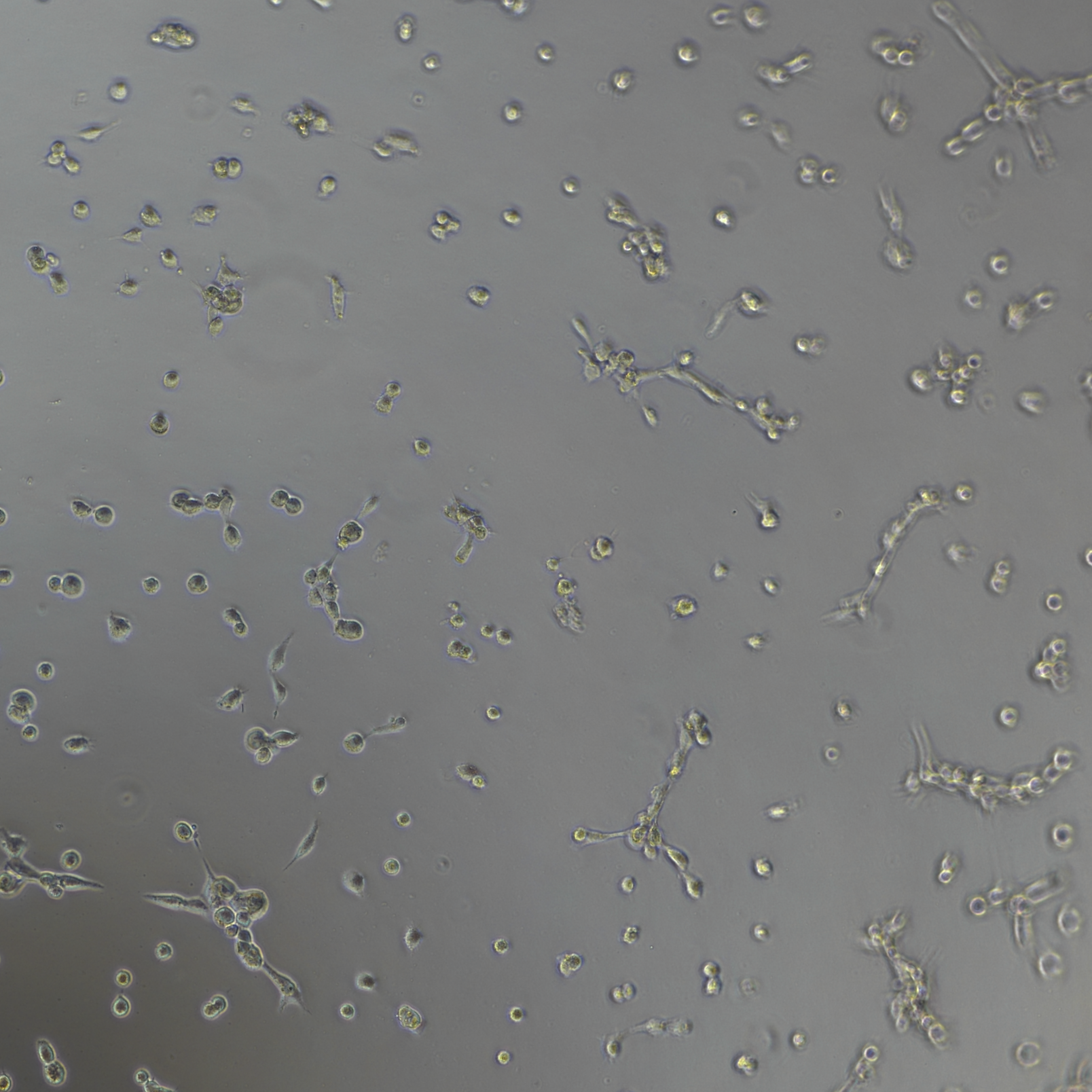

Supplement: Supplemental Information 80 [file peerj-10-13498-s080.tif]

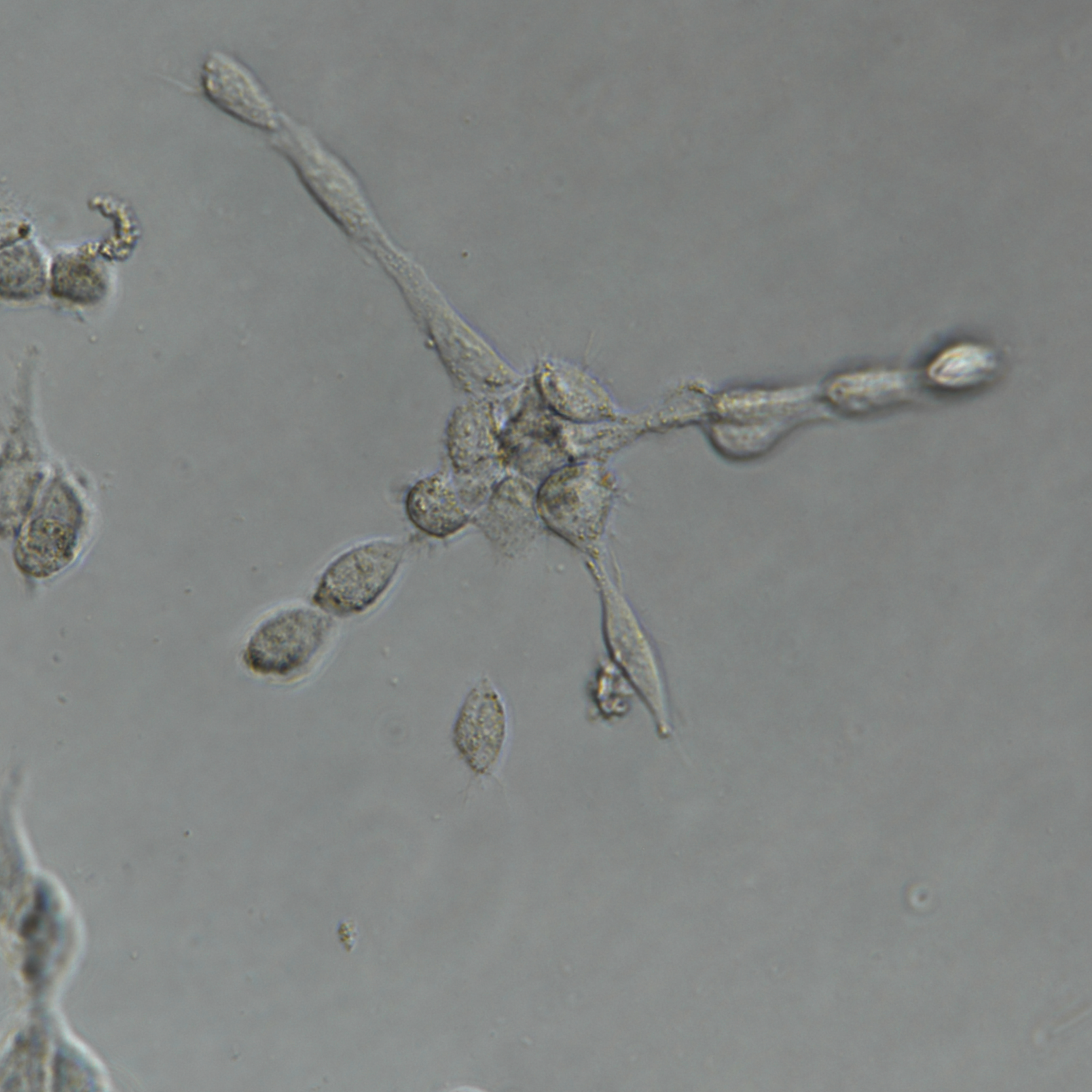

Supplement: Supplemental Information 81 [file peerj-10-13498-s081.tif]

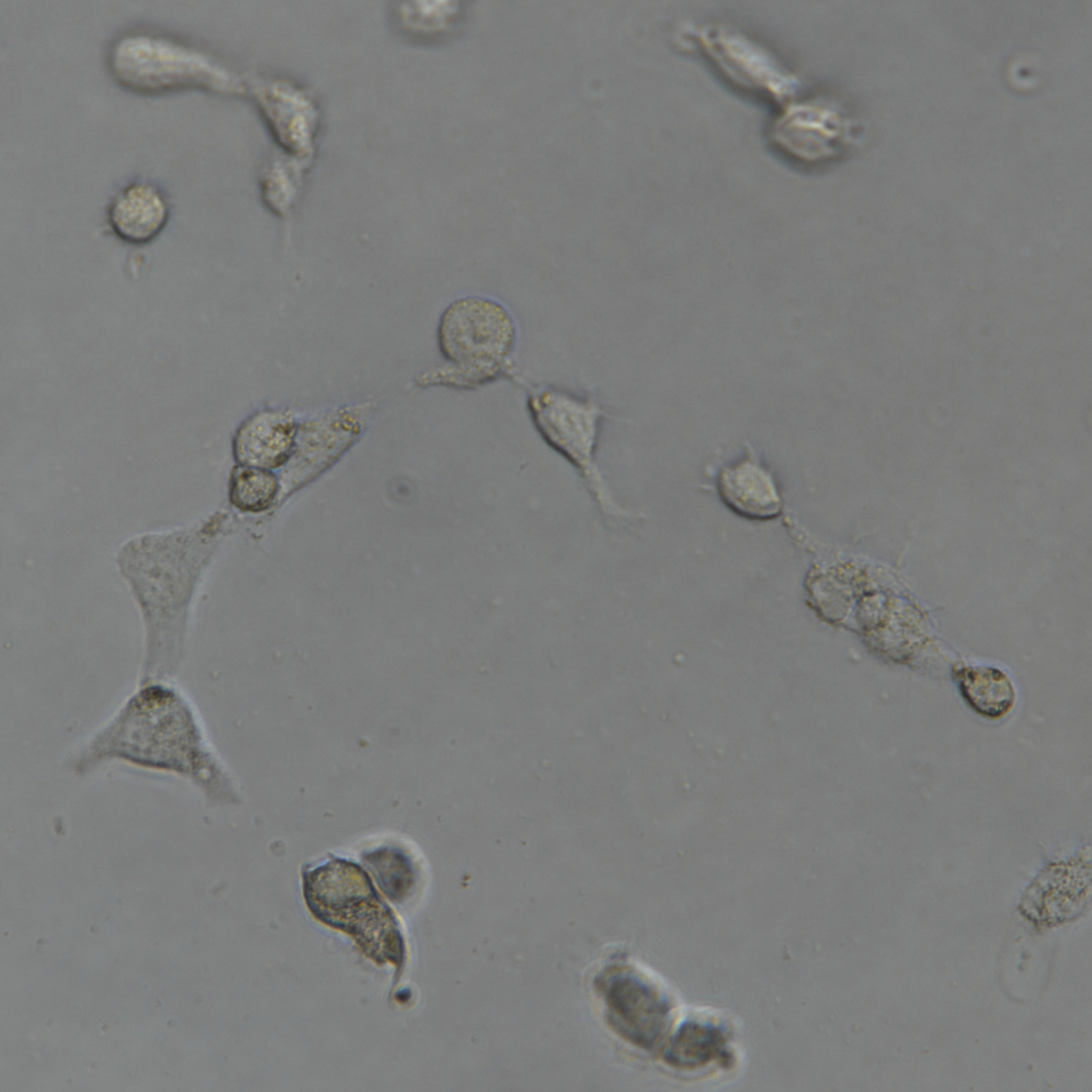

Supplement: Supplemental Information 82 [file peerj-10-13498-s082.tif]

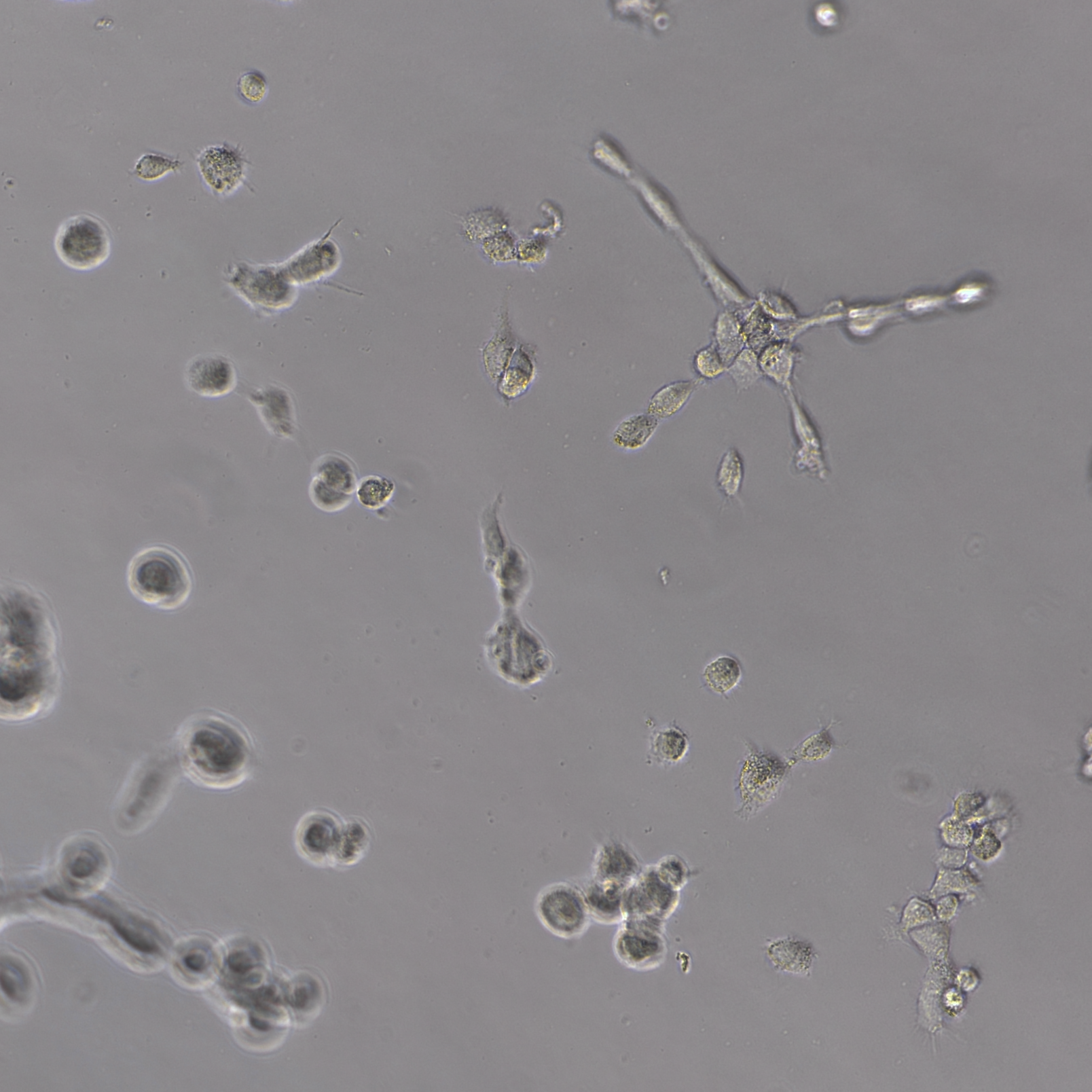

Supplement: Supplemental Information 83 [file peerj-10-13498-s083.tif]

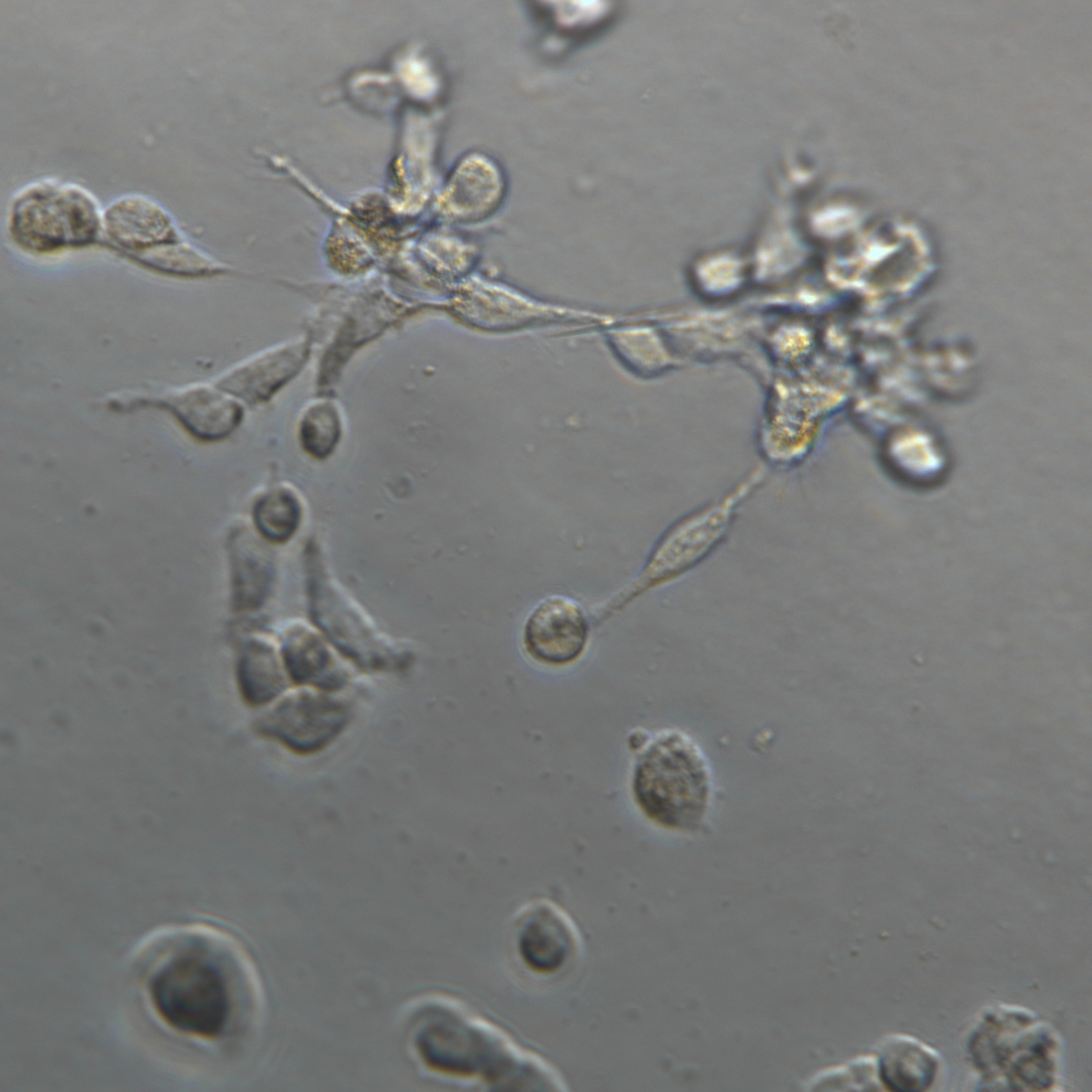

Supplement: Supplemental Information 84 [file peerj-10-13498-s084.tif]

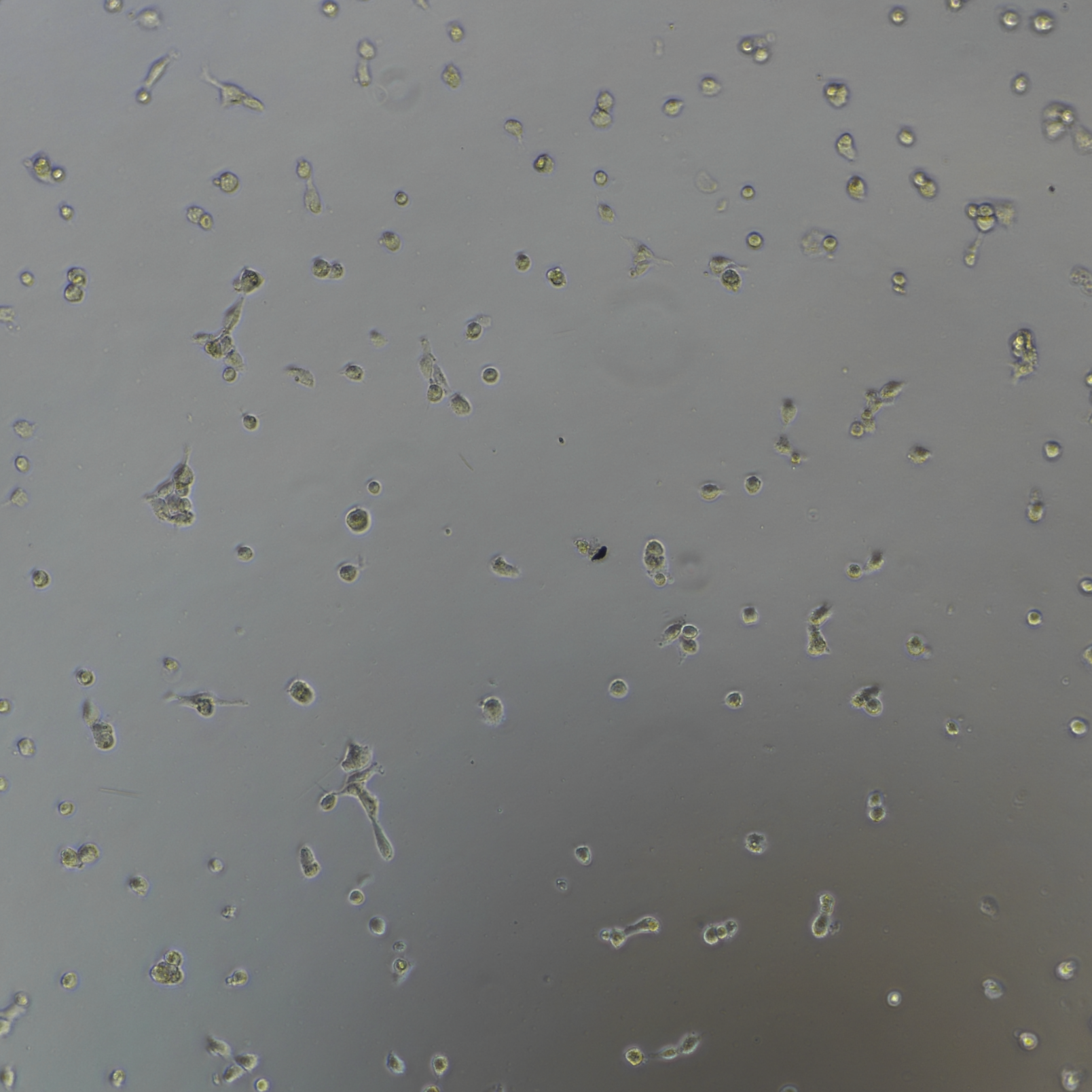

Supplement: Supplemental Information 85 [file peerj-10-13498-s085.tif]

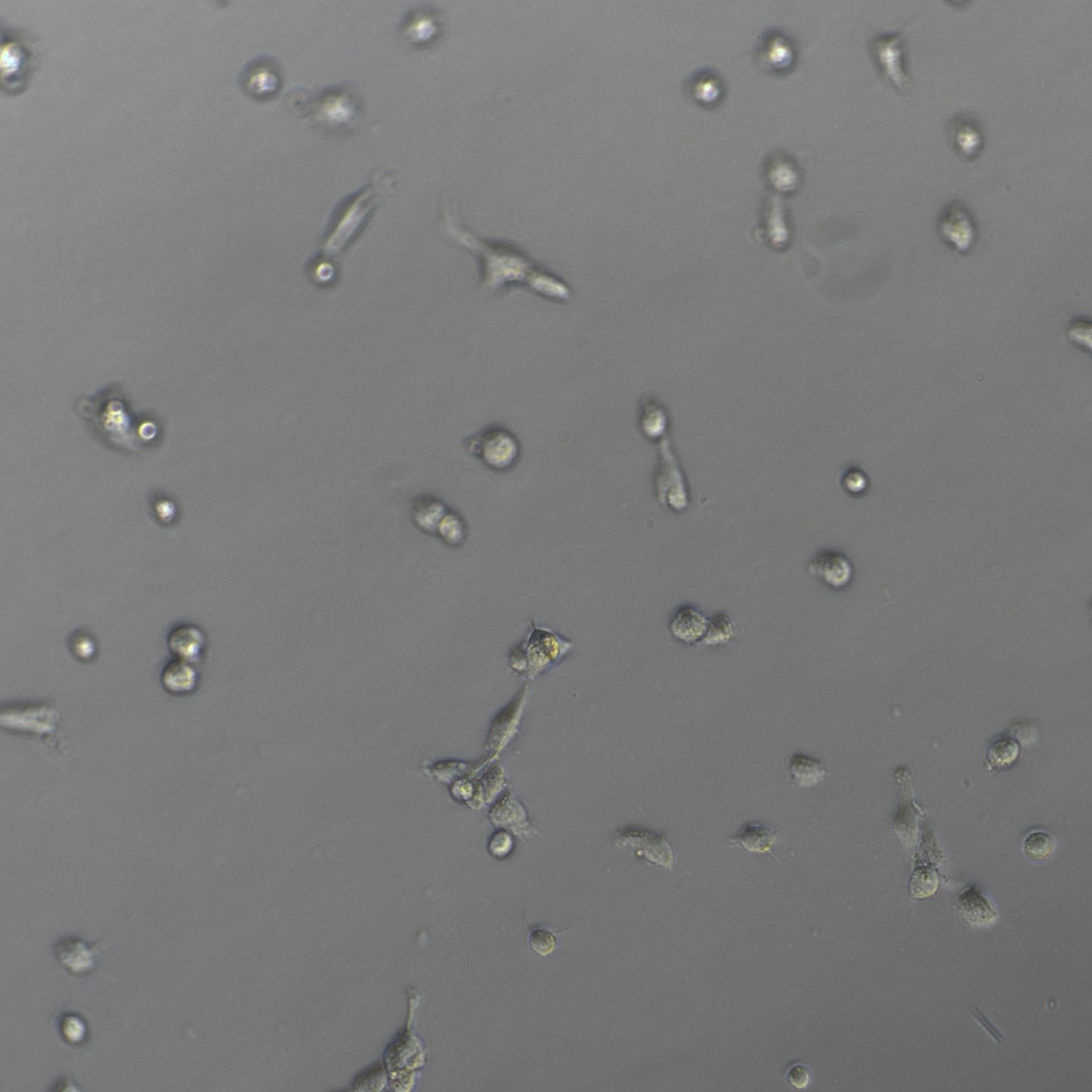

Supplement: Supplemental Information 86 [file peerj-10-13498-s086.tif]

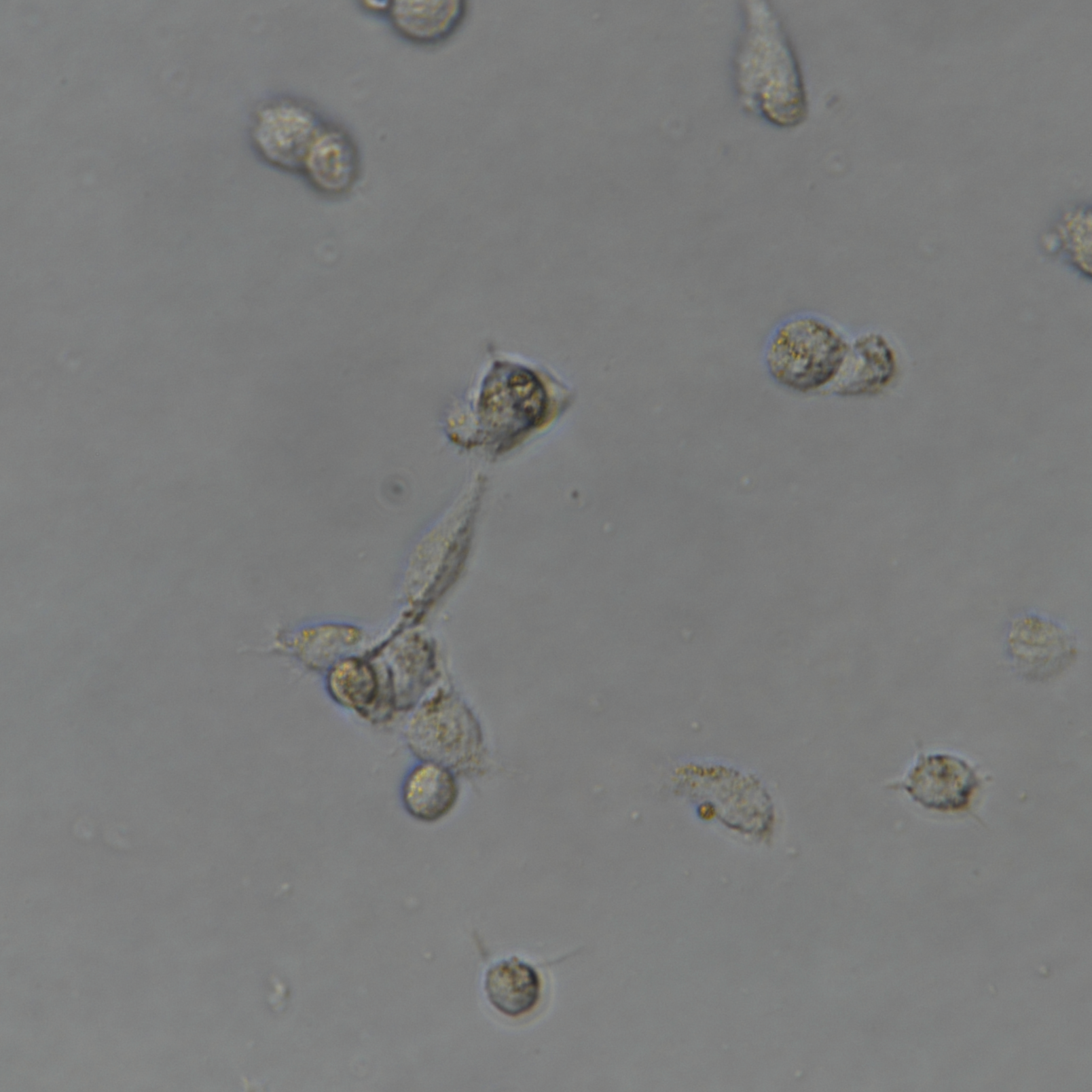

Supplement: Supplemental Information 87 [file peerj-10-13498-s087.tif]

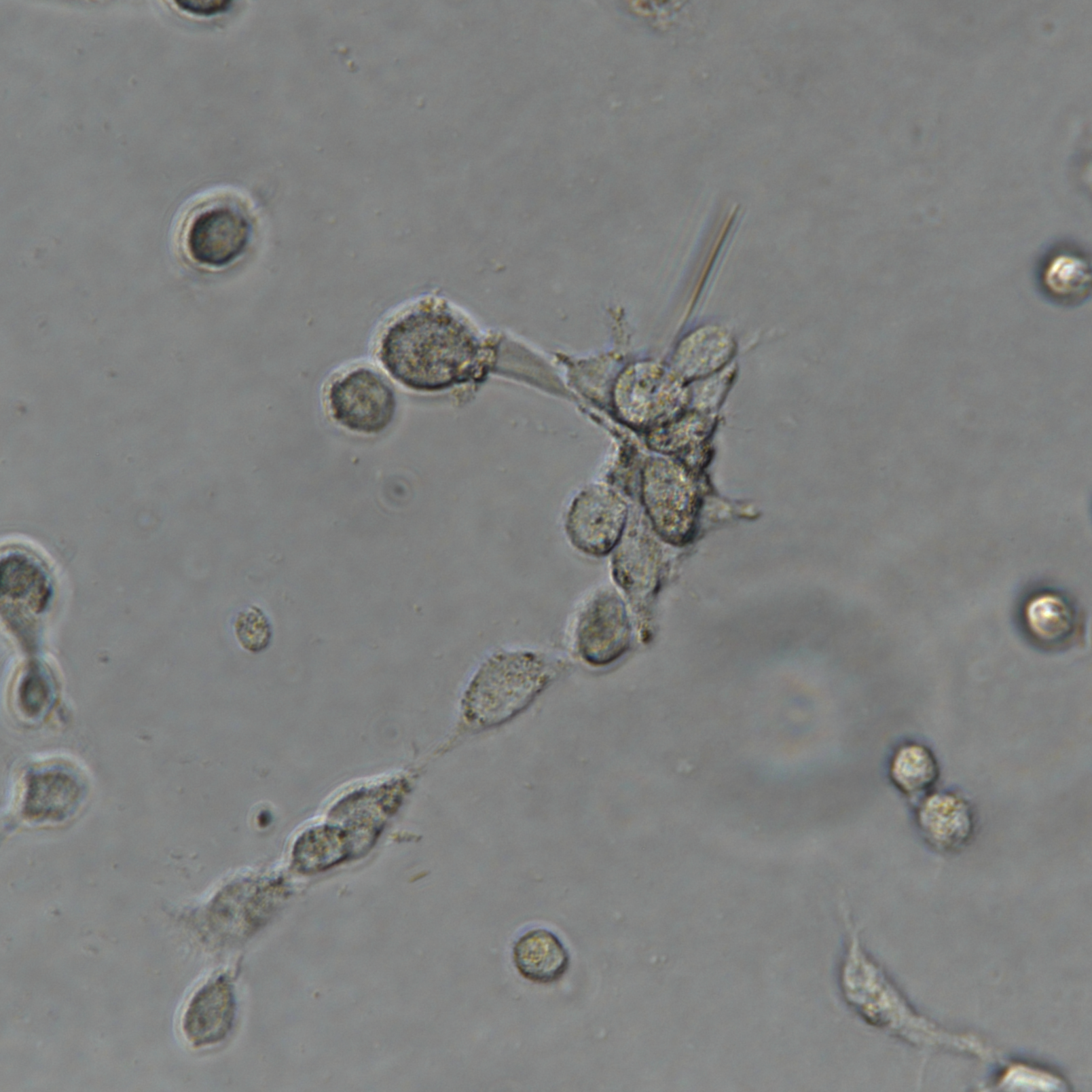

Supplement: Supplemental Information 88 [file peerj-10-13498-s088.tif]

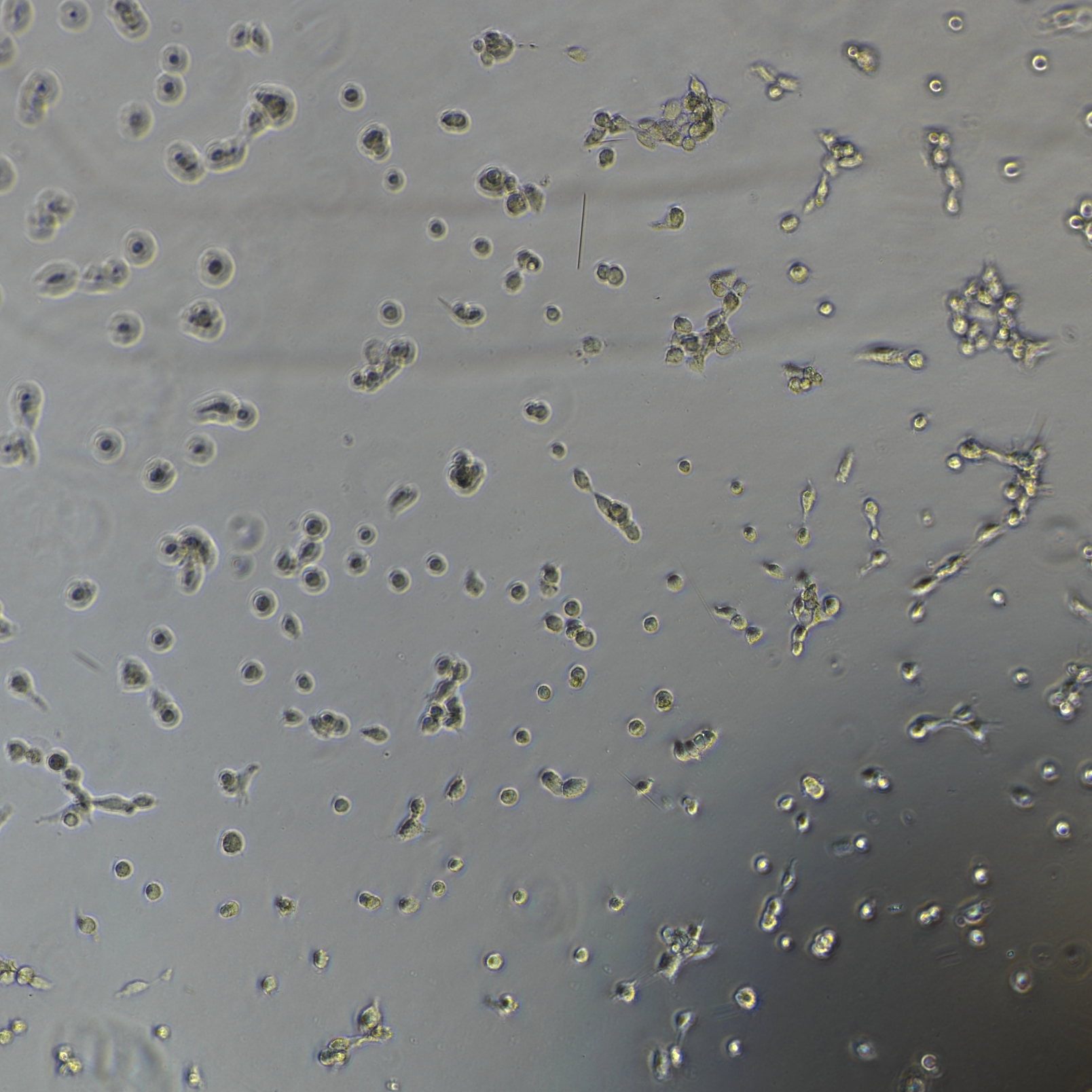

Supplement: Supplemental Information 89 [file peerj-10-13498-s089.tif]

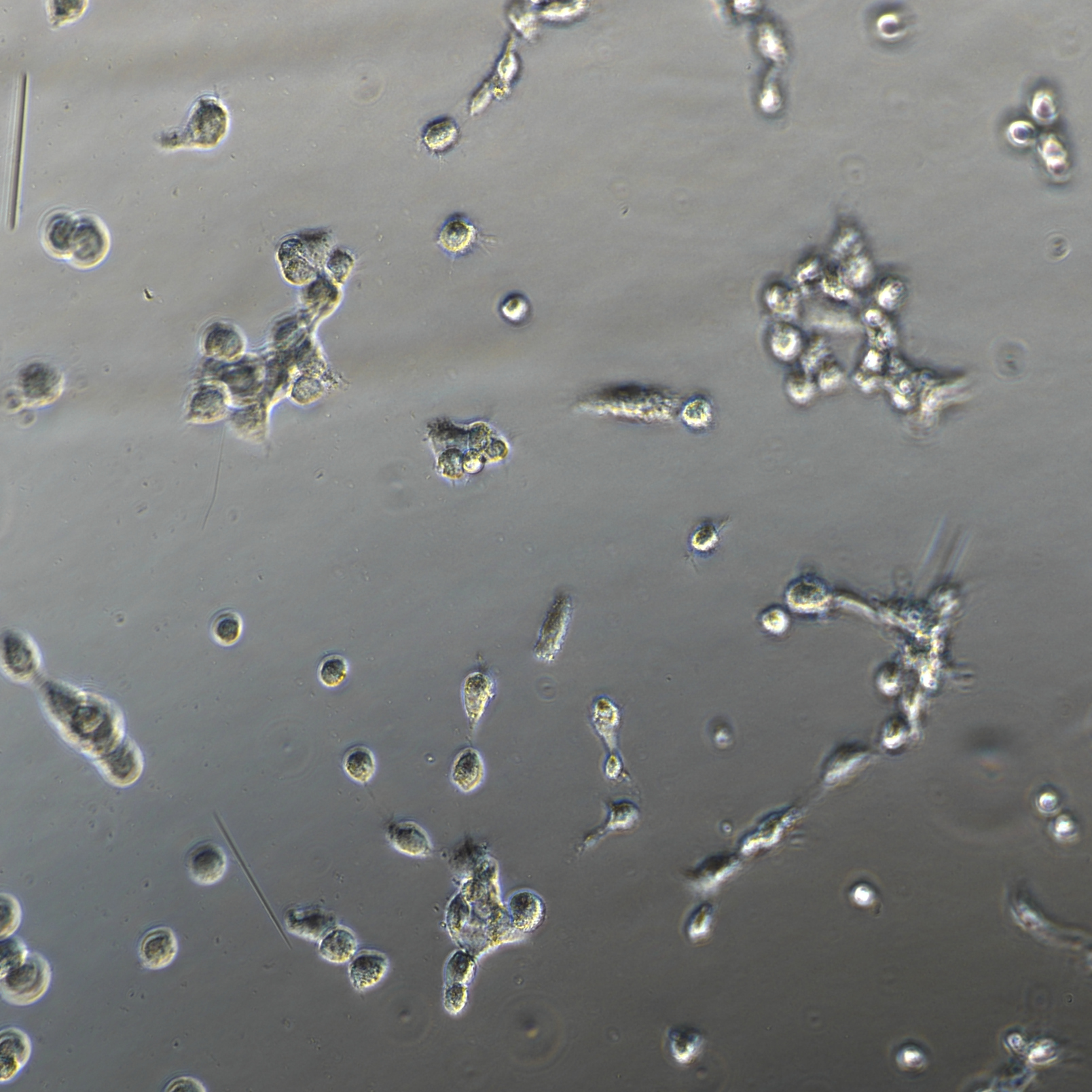

Supplement: Supplemental Information 90 [file peerj-10-13498-s090.tif]

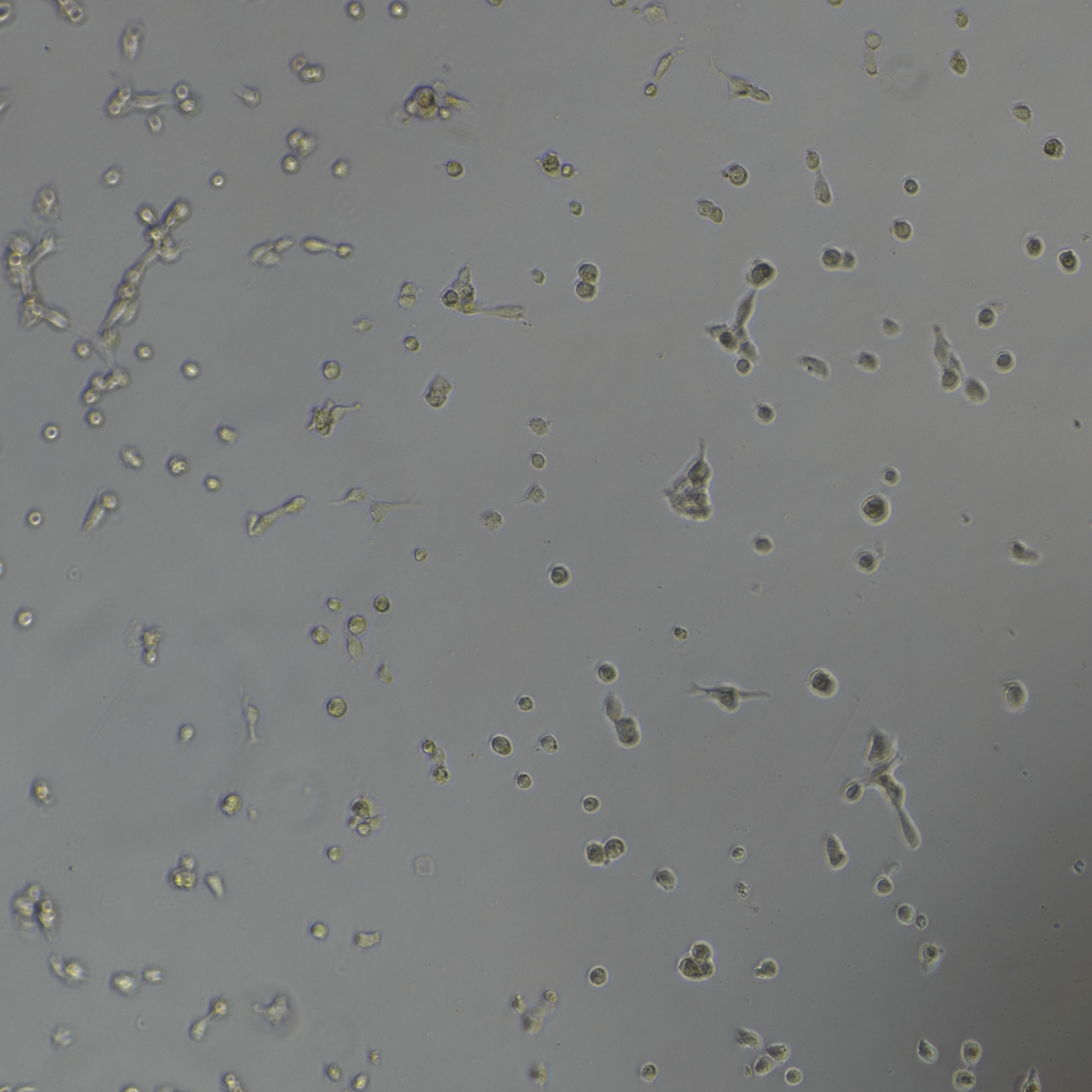

Supplement: Supplemental Information 91 [file peerj-10-13498-s091.tif]

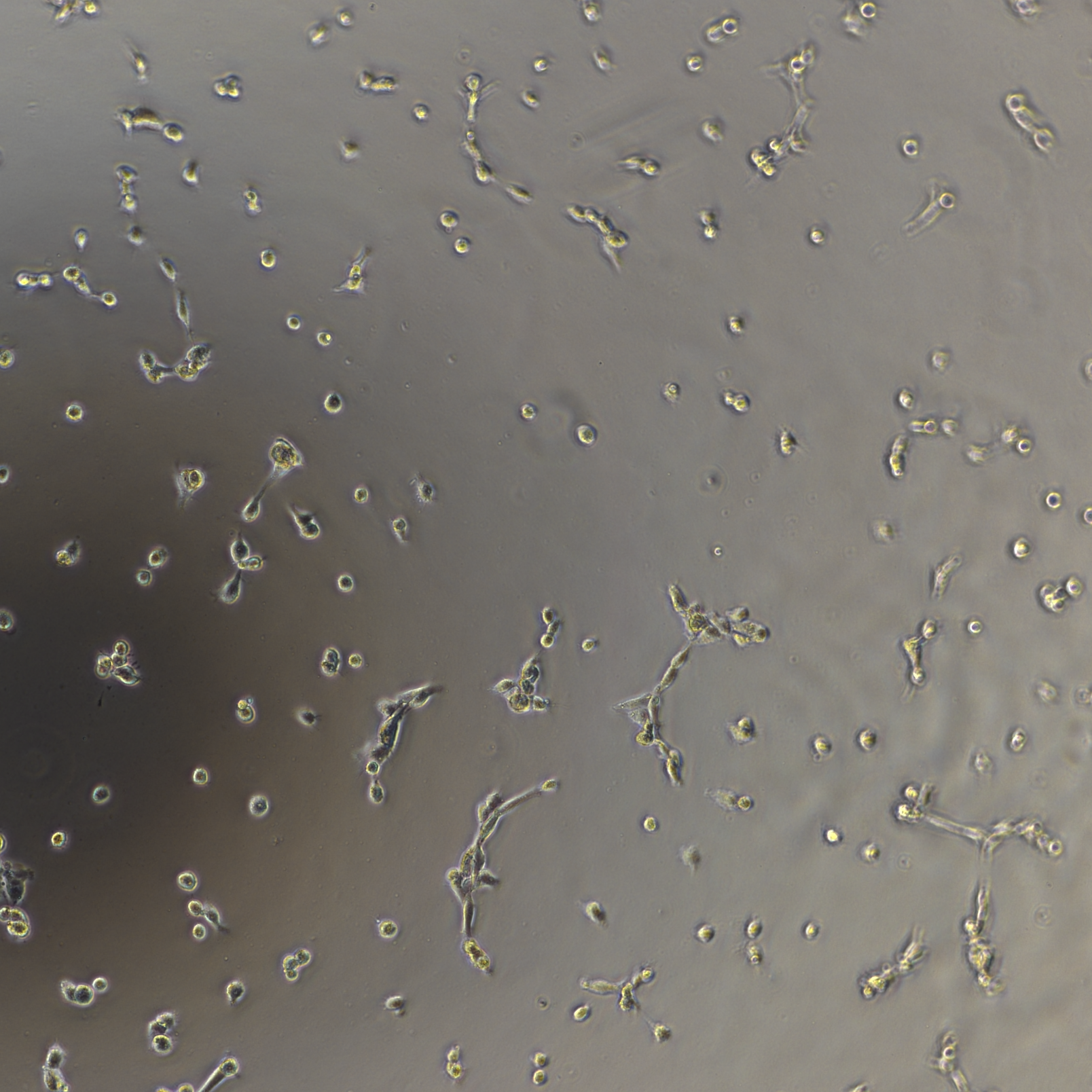

Supplement: Supplemental Information 92 [file peerj-10-13498-s092.tif]

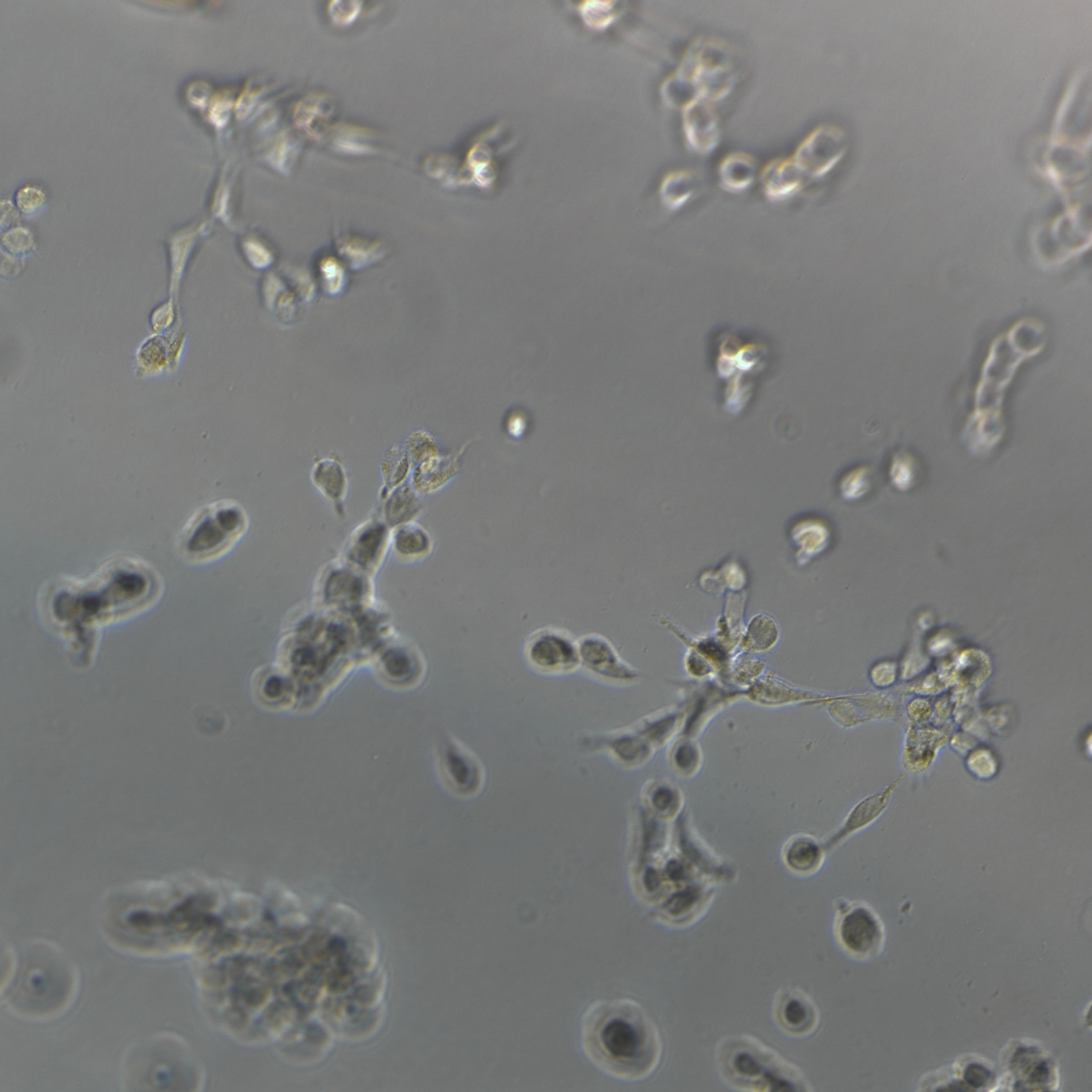

Supplement: Supplemental Information 93 [file peerj-10-13498-s093.tif]

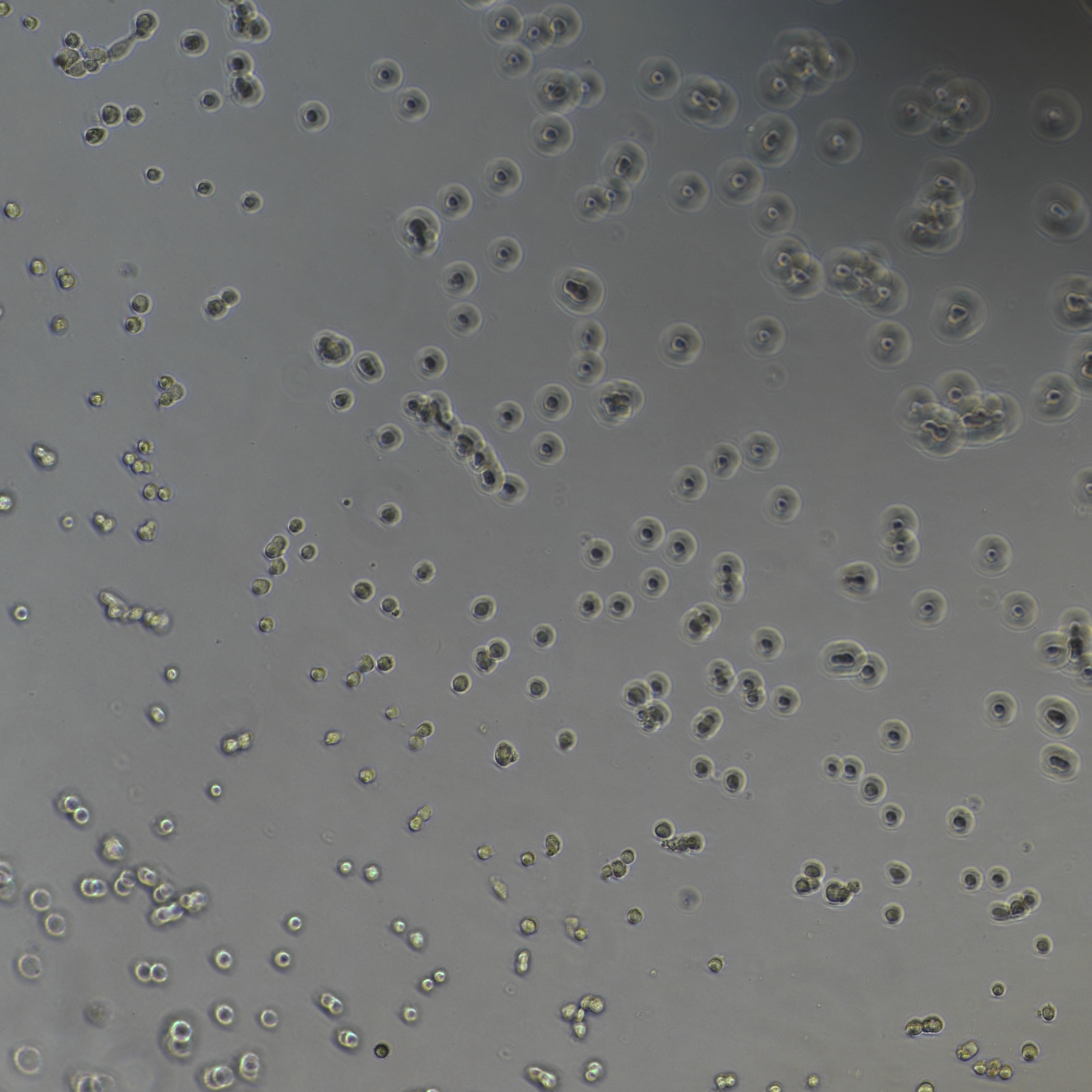

Supplement: Supplemental Information 94 [file peerj-10-13498-s094.tif]

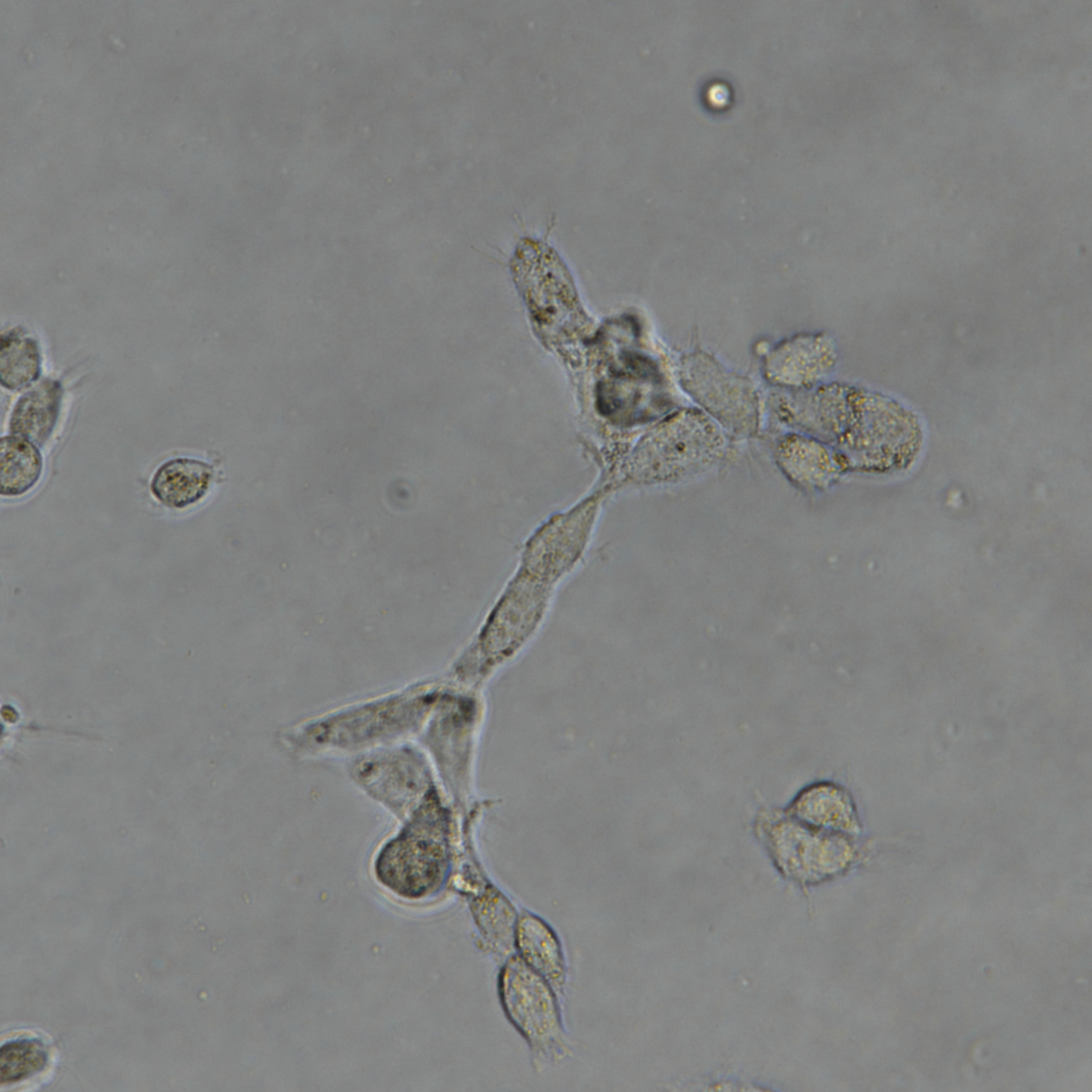

Supplement: Supplemental Information 95 [file peerj-10-13498-s095.tif]

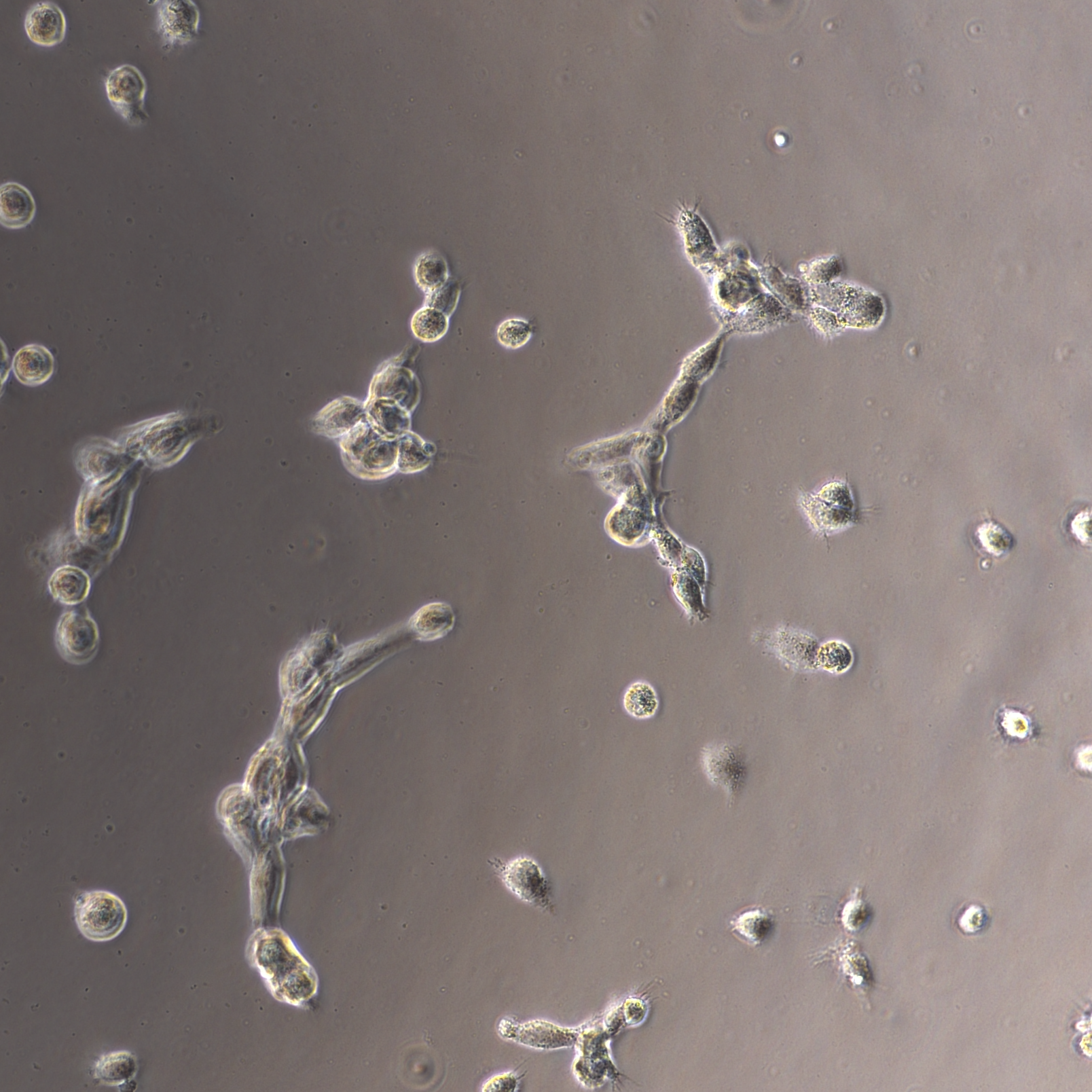

Supplement: Supplemental Information 96 [file peerj-10-13498-s096.tif]

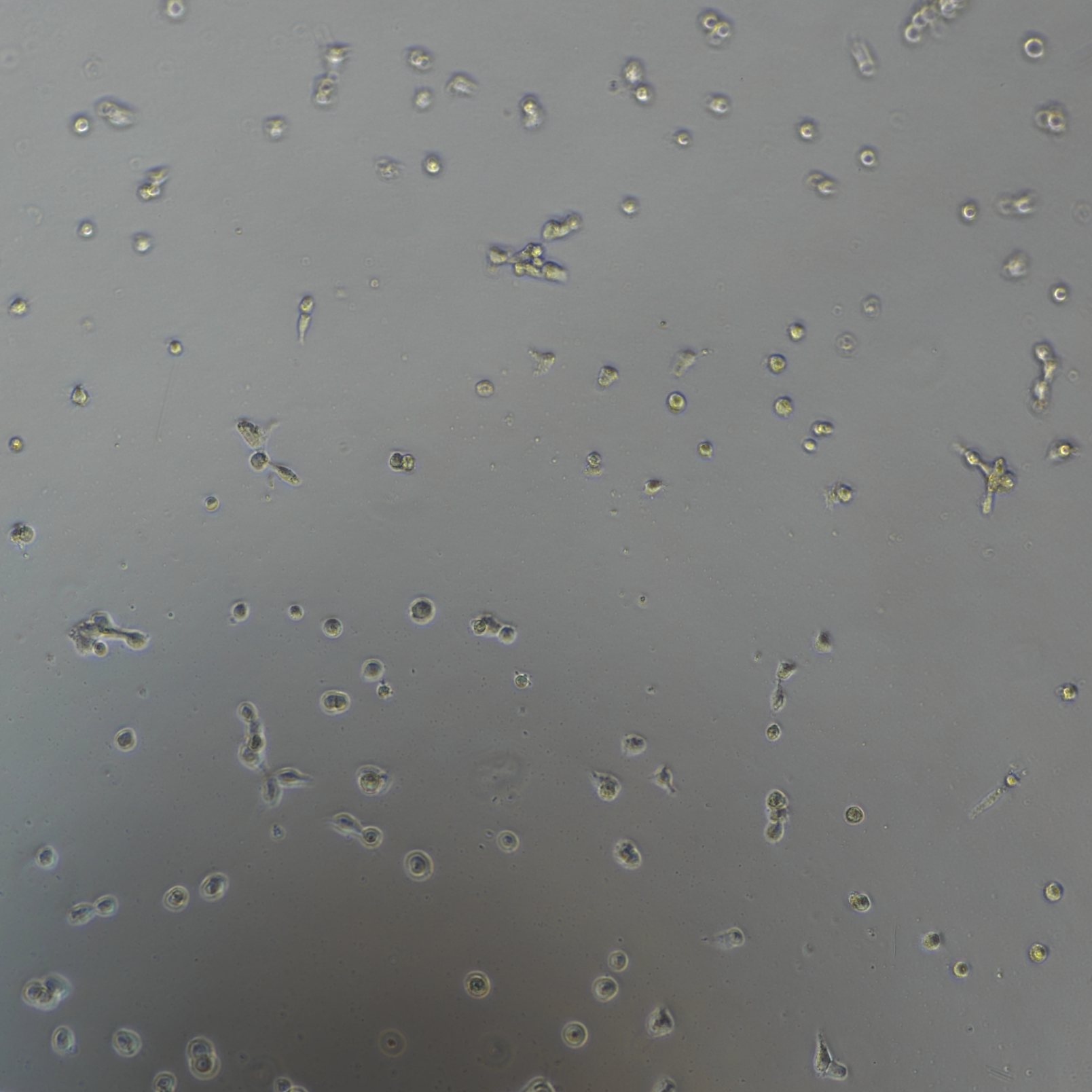

Supplement: Supplemental Information 97 [file peerj-10-13498-s097.tif]

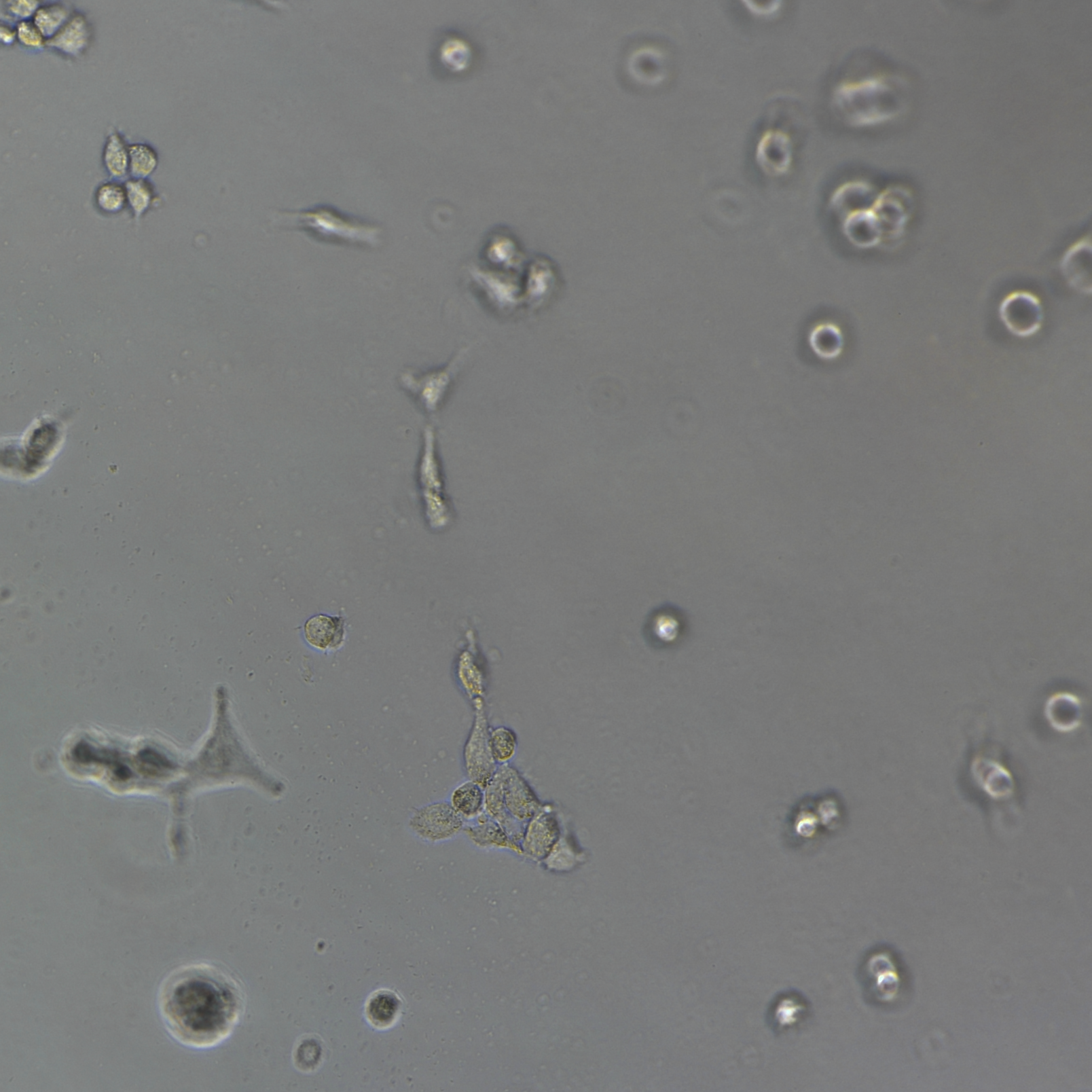

Supplement: Supplemental Information 98 [file peerj-10-13498-s098.tif]

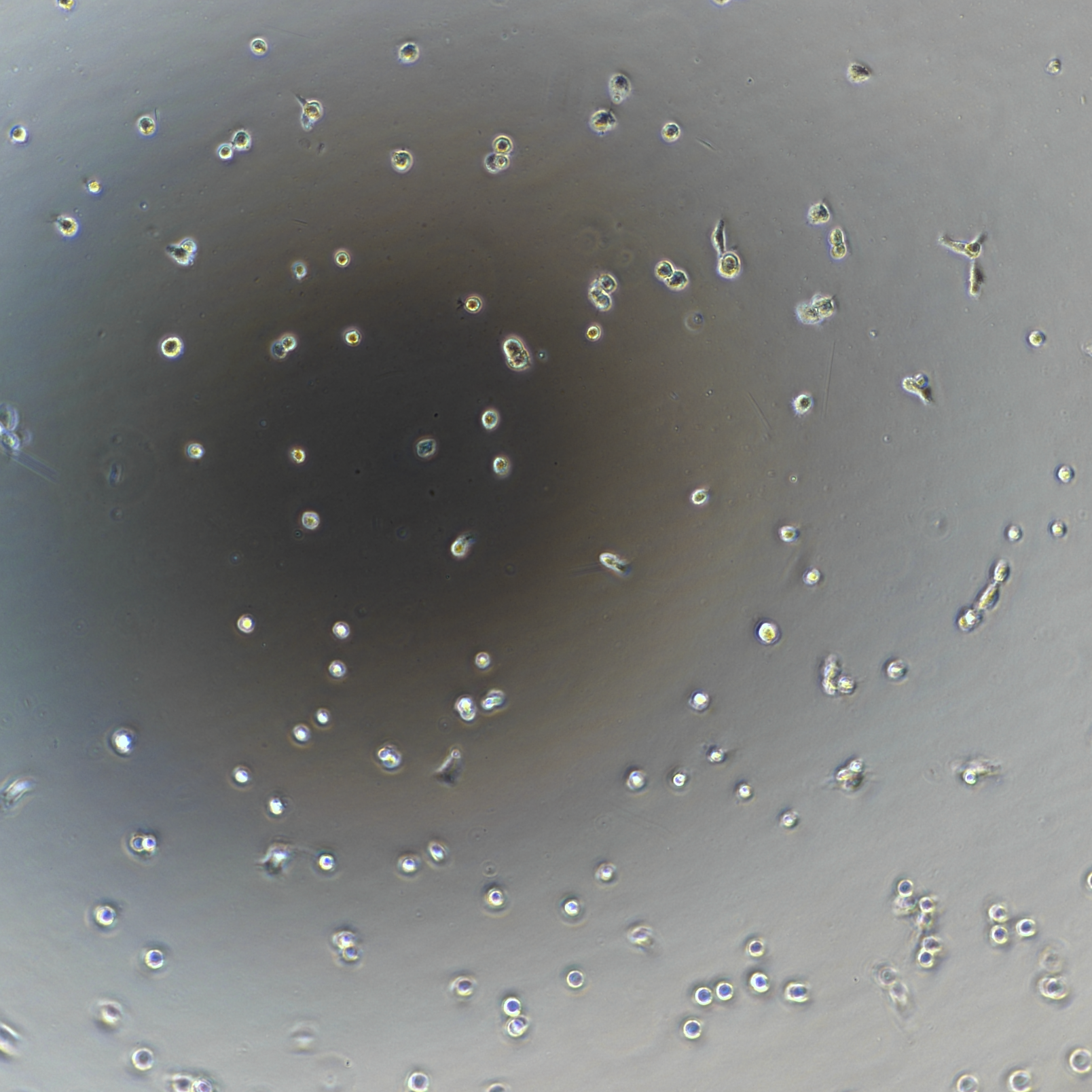

Supplement: Supplemental Information 99 [file peerj-10-13498-s099.tif]

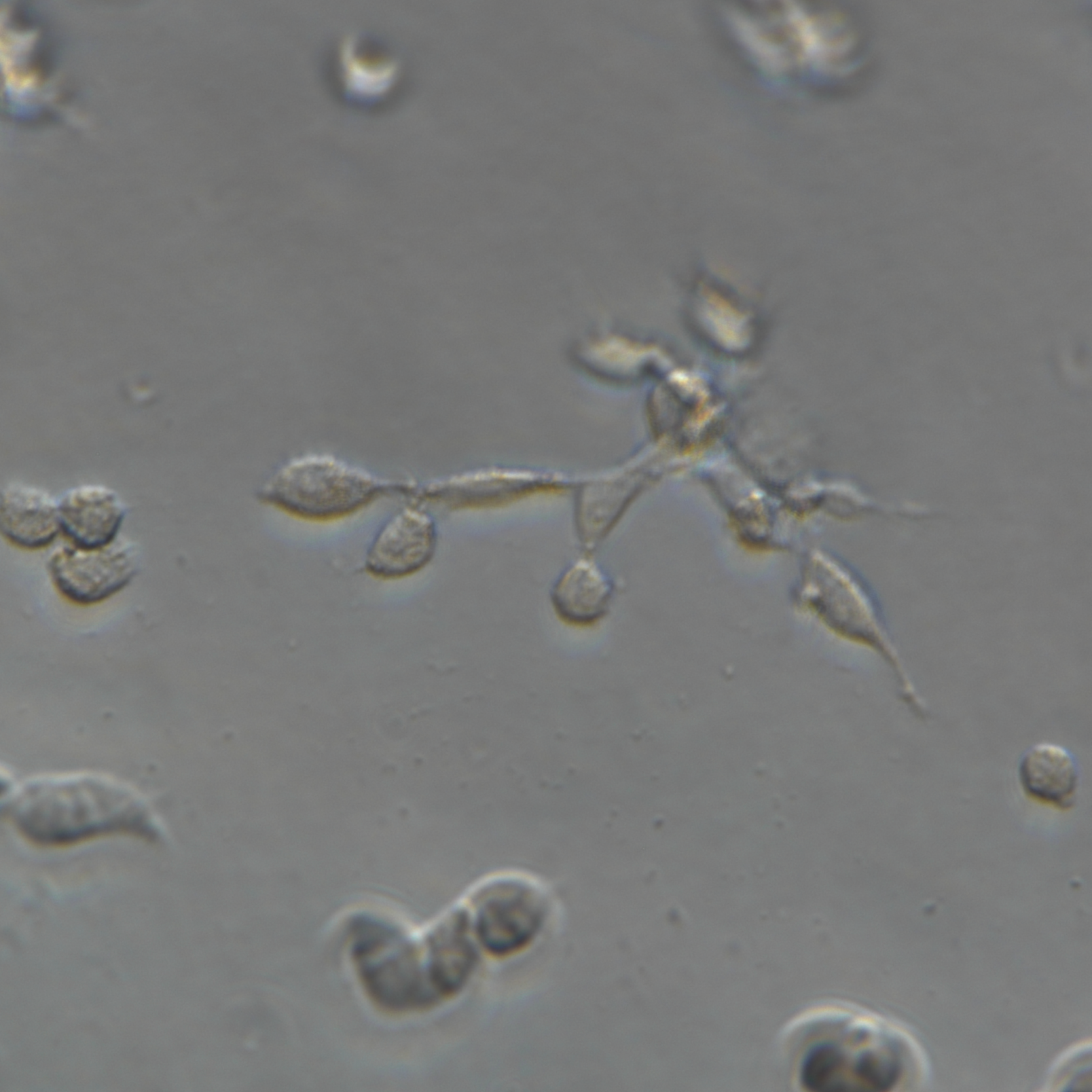

Supplement: Supplemental Information 100 [file peerj-10-13498-s100.tif]

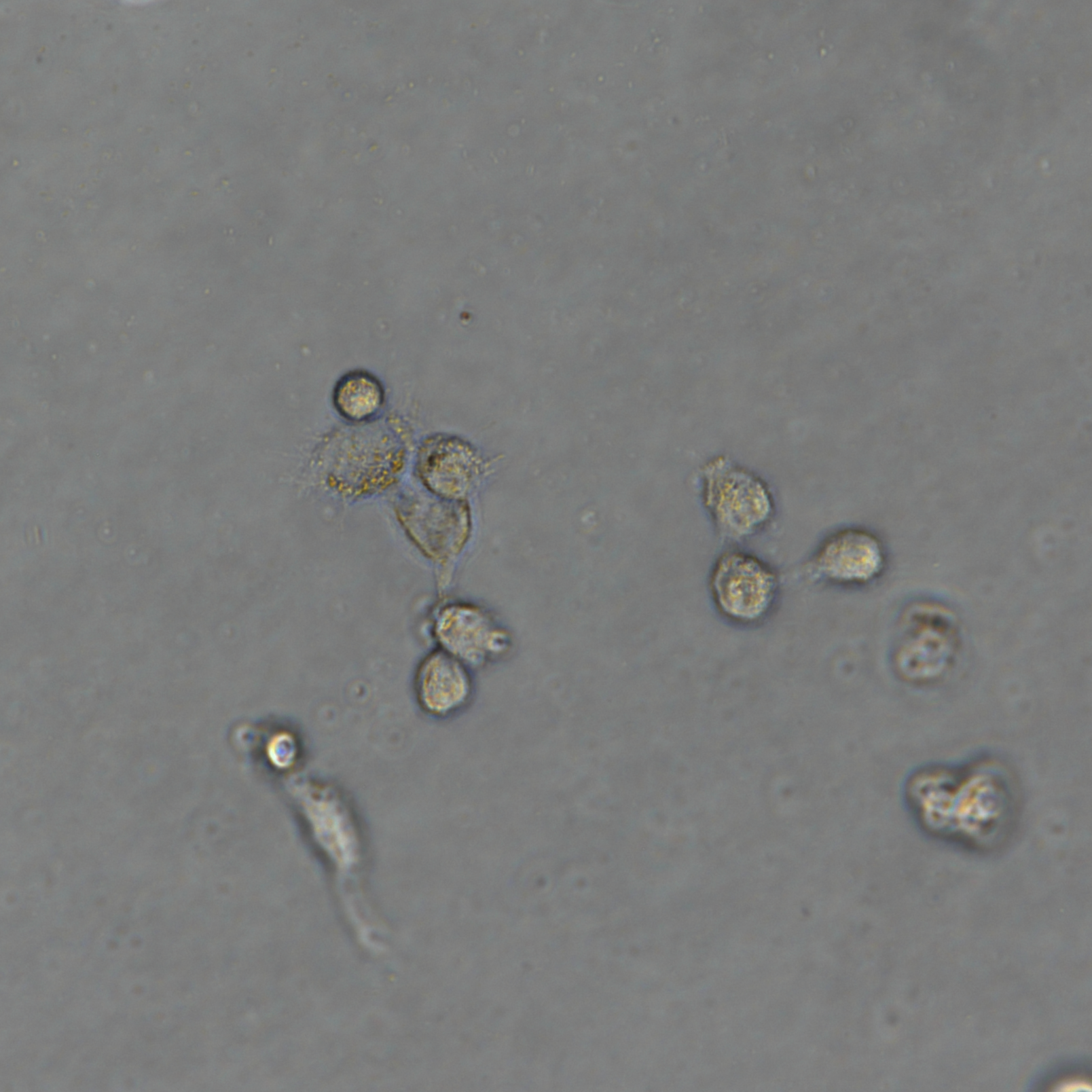

Supplement: Supplemental Information 101 [file peerj-10-13498-s101.tif]

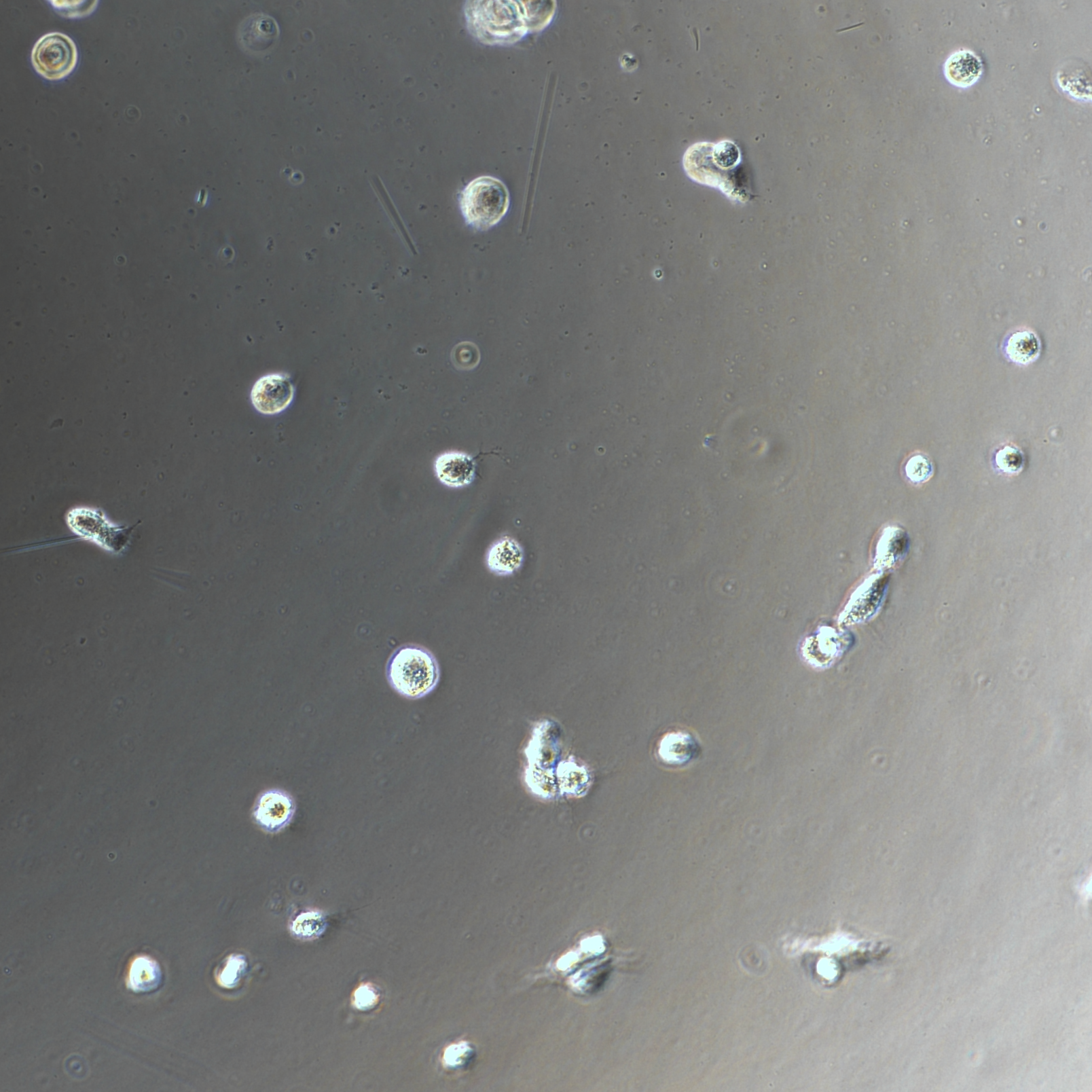

Supplement: Supplemental Information 102 [file peerj-10-13498-s102.tif]

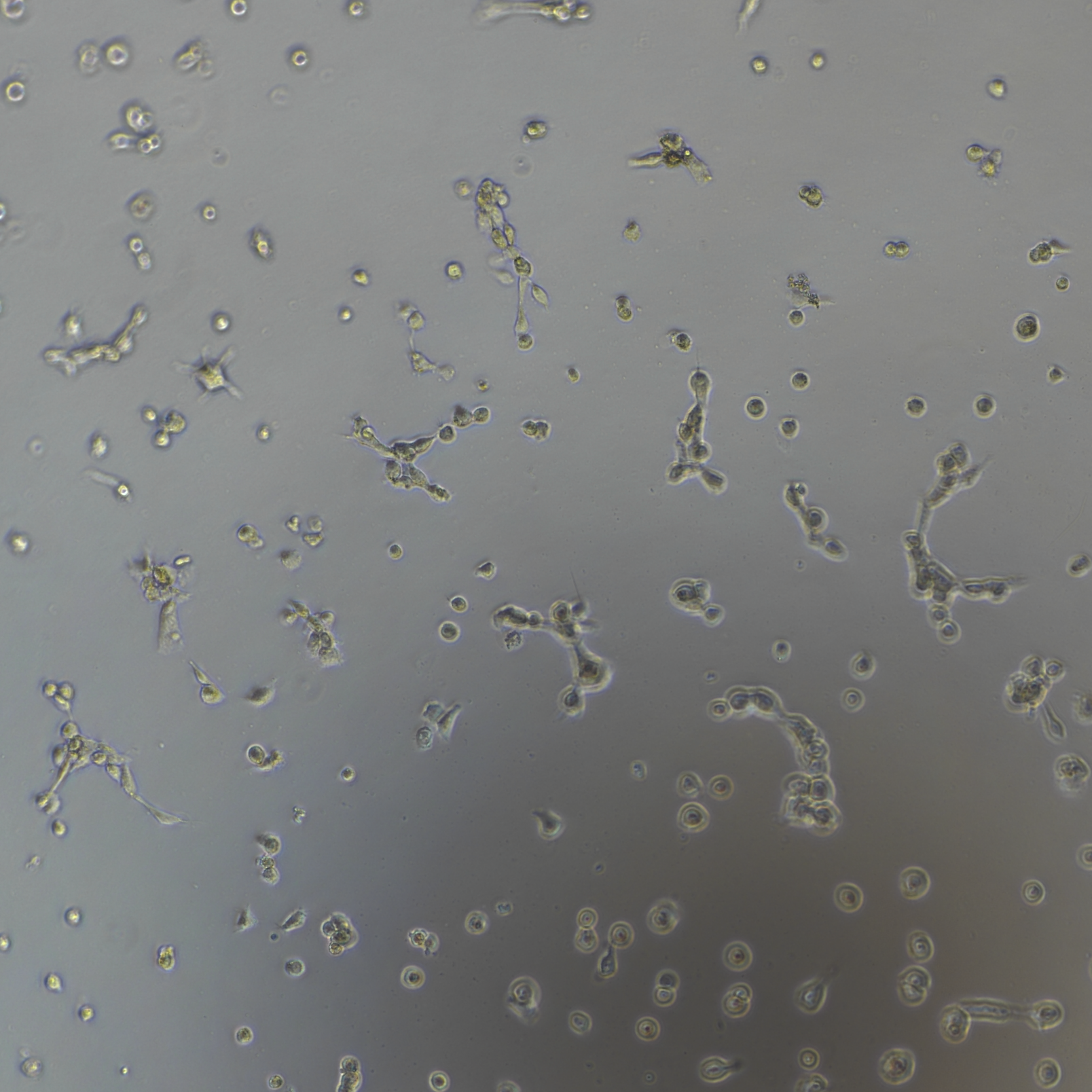

Supplement: Supplemental Information 103 [file peerj-10-13498-s103.tif]

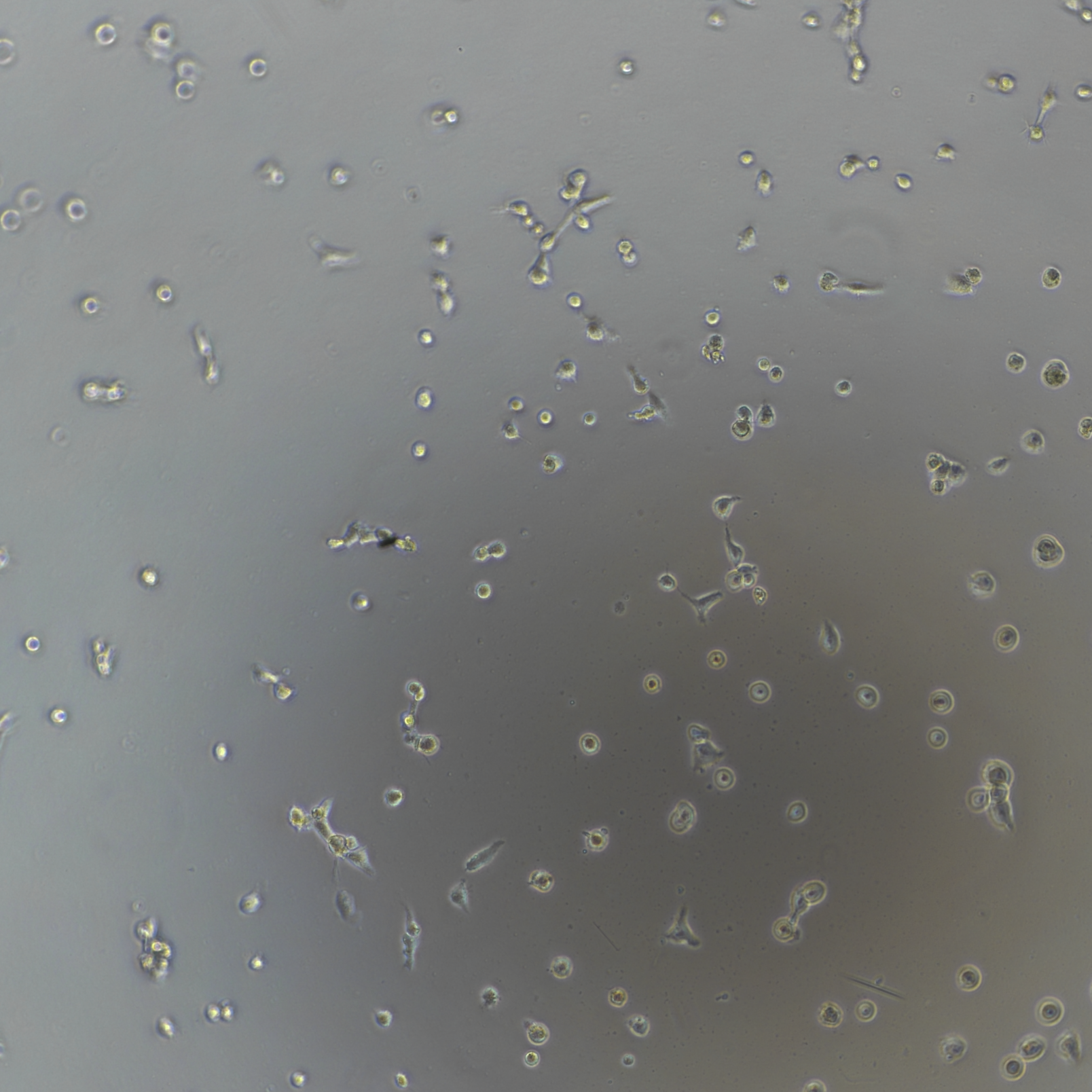

Supplement: Supplemental Information 105 [file peerj-10-13498-s105.tif]

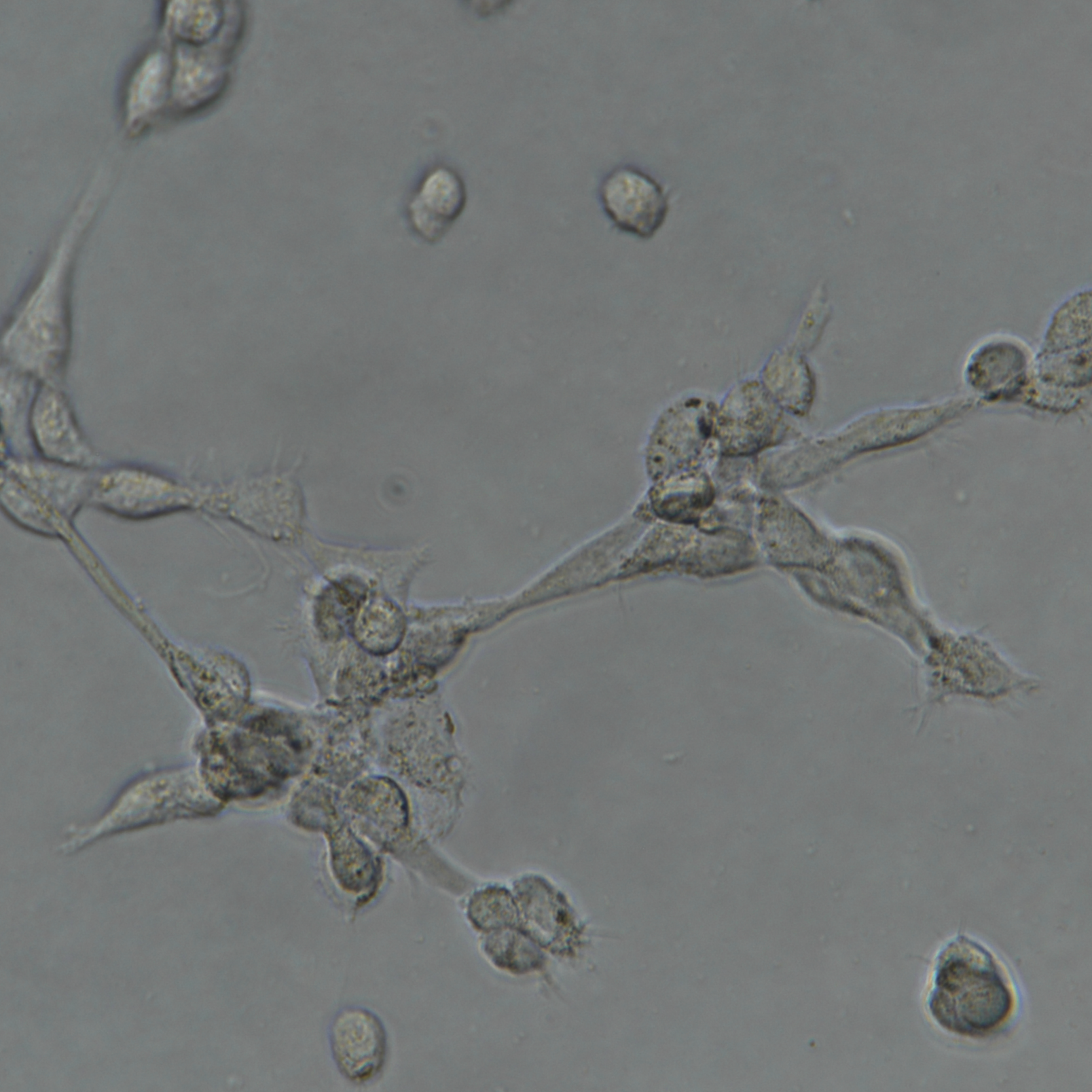

Supplement: Supplemental Information 106 [file peerj-10-13498-s106.tif]

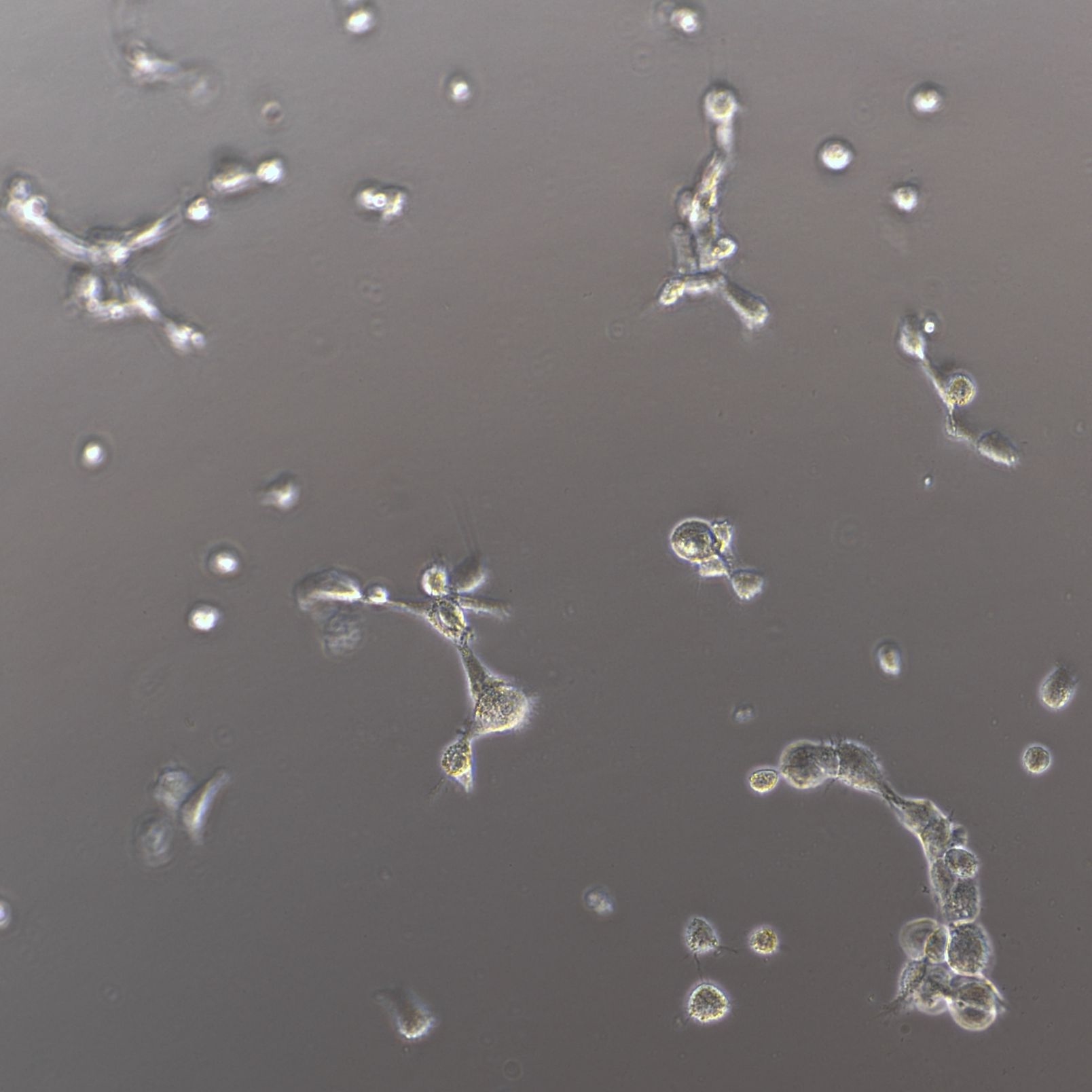

Supplement: Supplemental Information 107 [file peerj-10-13498-s107.tif]

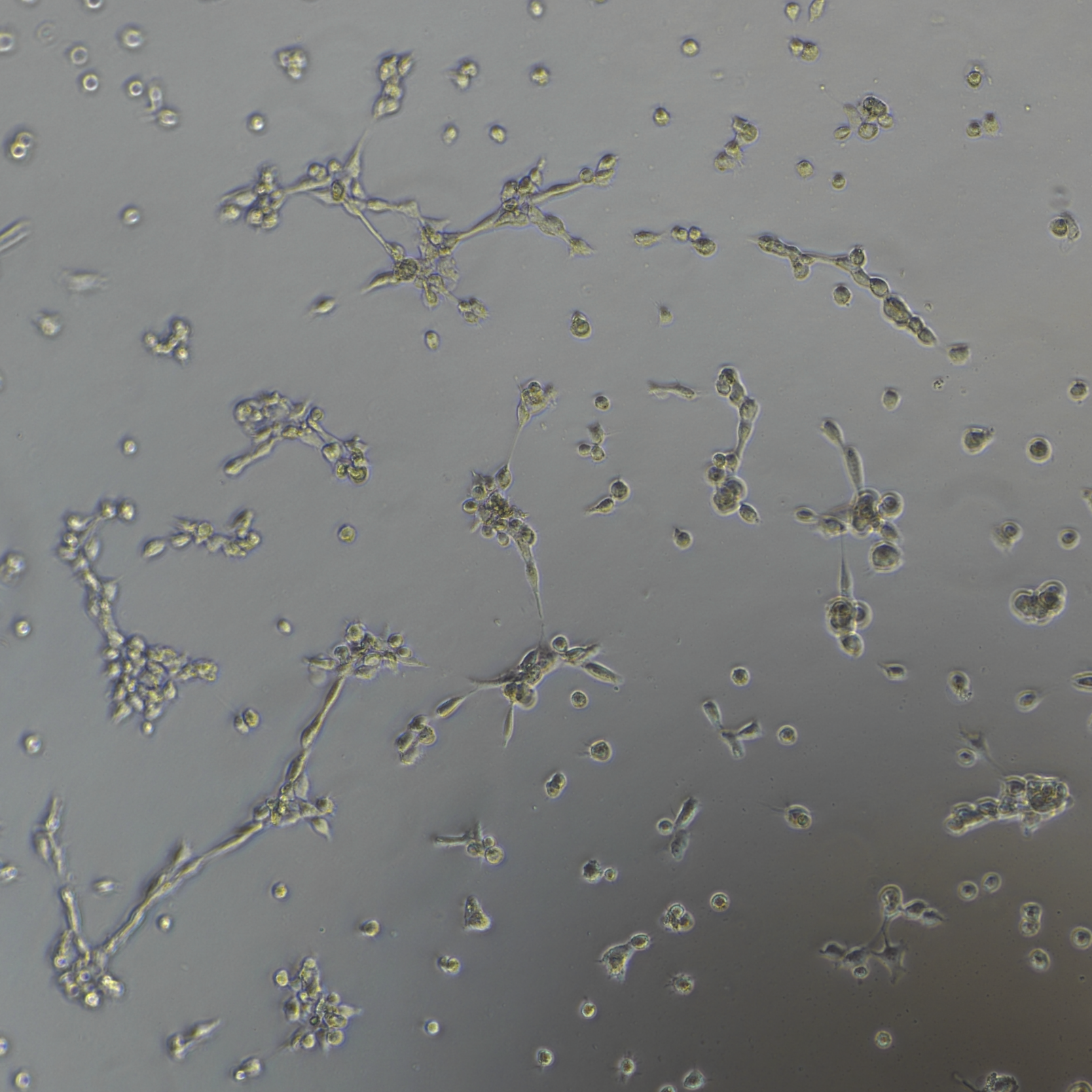

Supplement: Supplemental Information 108 [file peerj-10-13498-s108.tif]

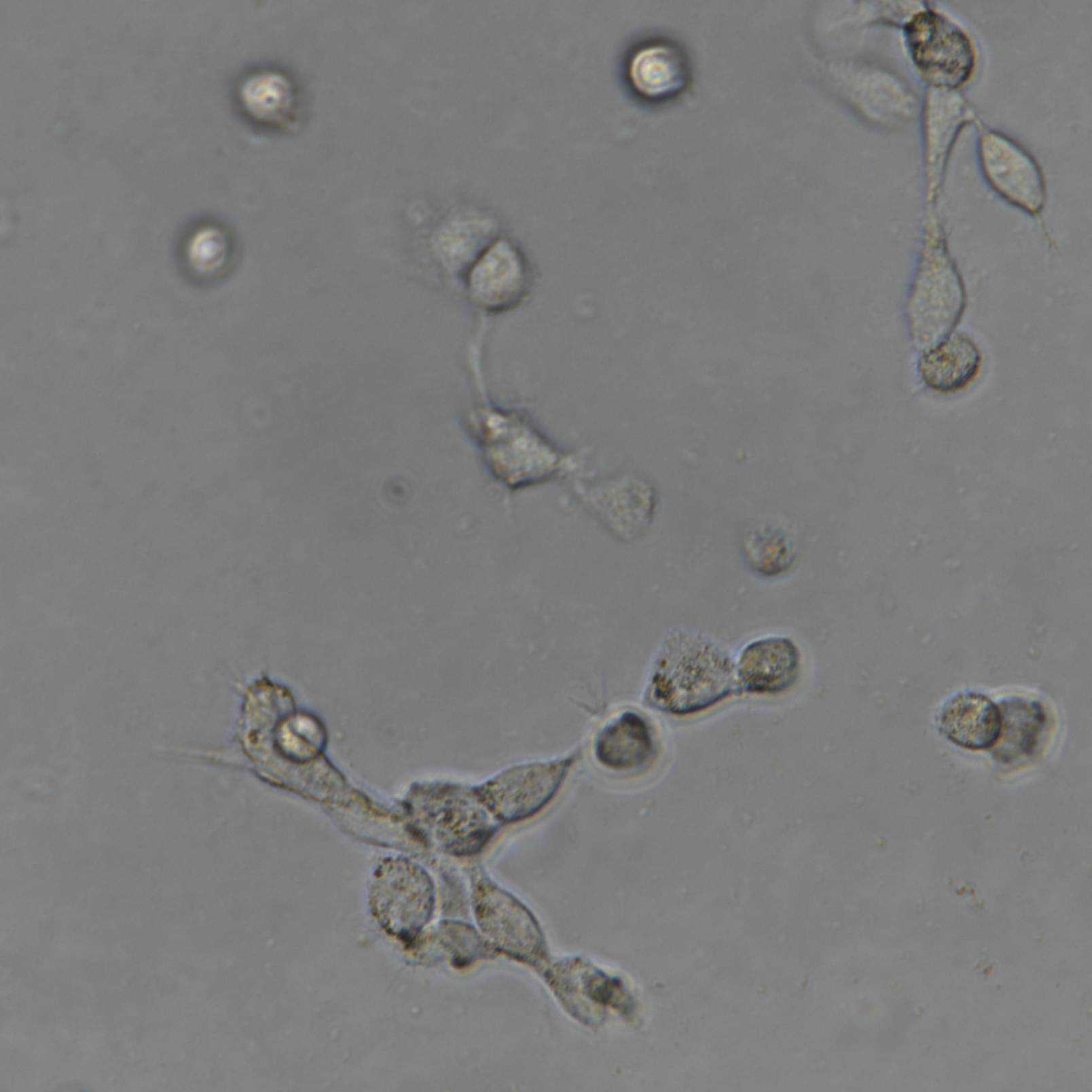

Supplement: Supplemental Information 109 [file peerj-10-13498-s109.tif]
