# Supplementary material for: Analysis of microRNA expression profiles in exosomes derived from acute myeloid leukemia by p62 knockdown and effect on angiogenesis
Source: PeerJ. 2022 Jul 22;10:e13498. doi: 10.7717/peerj.13498 (PMC9310811; doi:10.7717/peerj.13498)
Supplement: Supplemental Information 110 [file peerj-10-13498-s110.doc]

Miame Checklist

Part 1 Experiment description

None

Part 2 Array design./ Part 4 Hybridizations

**1, RNA extraction**

Total RNA was isolated using TRIzol (Invitrogen) . RNA quality and quantity was measured by using nanodrop spectrophotometer (ND-1000, Nanodrop Technologies) and RNA Integrity was determined by gel electrophoresis.

**2, miRNA labeling and array hybridization**

RNA labeling and array hybridization was according to Exiqon's manual.

1), After quality control, the miRCURY™ Hy3™/Hy5™ Power labeling kit (Exiqon, Vedbaek, Denmark) was used according to the manufacturer’s guideline for miRNA labelling by following steps:

a, 1μLRNA in 2.0 μL of water was combined with 1.0 μL of CIP buffer and CIP (Exiqon). The mixture was incubated for 30 min at 37°C.

b, The Reaction was terminated by incubation for 5 min at 95°C. Then 3.0 μL of labeling buffer, 1.5 μL of fluorescent label (Hy3TM), 2.0 μL of DMSO, 2.0 μL of labeling enzyme were added into the mixture.The labeling reaction was incubated for 1 h at 16°C

c, Terminated by incubation for 15 min at 65°C.

2), After stopping the labeling procedure, the Hy3™-labeled samples were hybridized on the miRCURYTM LNA Array (v.19.0) (Exiqon) according to array manual.

a, The total 25 μL mixture from Hy3™-labeled samples with 25 μL hybridization buffer were first denatured for 2 min at 95°C, incubated on ice for 2 min

b, Then hybridized to the microarray for 16–20 h at 56°C in a 12-Bay Hybridization Systems (Hybridization System - Nimblegen Systems, Inc., Madison, WI, USA)

c, Following hybridization, the slides were achieved, washed several times using Wash buffer kit (Exiqon)

3, Then the slides were scanned using the Axon GenePix 4000B microarray scanner (Axon Instruments, Foster City, CA).

Part 5 Measurements

Yes

Part 6 Normalization controls

Yes
